# A histological survey of avian post-natal skeletal ontogeny

**DOI:** 10.7717/peerj.12160

**Published:** 2021-10-01

**Authors:** Jessie Atterholt, Holly N. Woodward

**Affiliations:** 1 Graduate College of Biomedical Sciences, Western University of Health Sciences, Pomona, California, United States; 2 Department of Integrative Biology, University of California, Berkeley, California, United States; 3 Department of Anatomy and Cell Biology, Oklahoma State University Center for Health Sciences, Tulsa, Oklahoma, United States

**Keywords:** Post-natal development, Aves, Osteohistology, Altricial-precocial spectrum, Bone growth

## Abstract

Bone histology of crown-group birds is a research topic of great interest, permitting insight into the evolution of remarkably high growth rates in this clade and variation across the altricial-precocial spectrum. In this study, we describe microanatomical characteristics of the humerus and femur in partial growth series from 14 crown group birds representing ten major clades (Struthioniformes, Galliformes, Apodiformes, Columbiformes, Charadriiformes, Accipitriformes, Strigiformes, Psittaciformes, Falconiformes, and Passeriformes). Our goals were to: (1) describe the microanatomy of each individual; (2) make inter-and intra-taxonomic comparisons; (3) assess patterns that correspond with developmental mode; and (4) to further parse out phylogenetic, developmental, and functional constraints on avian osteological development. Across taxa, the femoral and humeral tissue of neonates can be broadly characterized as highly-vascularized, disorganized woven bone with great variation in cortical thickness (inter-and intrataxonomically, within an individual specimen, and within a single section). The tissue of precocial chicks is relatively more mature at hatching than in altricial, but other categories along the developmental spectrum were less easy to distinguish, thus we were unable to identify a definitive histological proxy for developmental mode. We did not find evidence to support hypotheses that precocial chicks exclusively have thicker cortices and more mature bone in the femur than the humerus at time of hatching; instead, this is a characteristic of nearly all taxa (regardless of developmental mode), suggesting deep evolutionary origins and the effects of developmental channeling. Bone tissue in adults exhibited unexpected variation, corresponding to differences in body size. Large-bodied birds have cortices of fibrolamellar bone, but organization of tissue increases and vascularity decreases with diminishing body size. The outer circumferential layer (OCL) also appears at earlier growth stages in small-bodied taxa. Thus, while the OCL is indicative of a cessation of appositional growth it is not always indicative of cortical maturity (that is, maximum organization of bony tissue for a given taxon). Small size is achieved by truncating the period of fast growth; manipulation of the timing of offset of bone growth is therefore an important factor in changing growth trajectories to alter adult body size.

## Introduction

The description and evolution of osteohistological structures and characteristics of avian adult bone have been a subject of scientific investigation for decades, particularly following the discovery of the dinosaurian ancestry of birds. Modern birds are extremely fast-growing animals (de Ricqlès et al., 1991; Starck & Ricklefs, 1998; Erickson et al., 2009; Wilson & Chin, 2014), as were many non-avialan dinosaurs. However, many initial studies of bone histology in Mesozoic avialans concluded that stem-group birds were either moderately more slow-growing (as in ornithuromorphs (Chinsamy, Chiappe & Dodson, 1995; Bell et al., 2010)), or drastically slower (as in the case of enantiornithines and *Archaeopteryx* (Chinsamy, Chiappe & Dodson, 1994; Cambra-Moo et al., 2006; Erickson et al., 2009; O’Connor et al., 2014)). Subsequent work has begun to reveal a greater diversity and complexity of life history strategies among these Mesozoic clades than previously understood. In *Archaeopteryx*, Voeten et al. (2018) demonstrated that immature ontogenetic stages of this taxon have vascular areas comparable to some extant taxa, and therefore likely a higher rate of bone growth than previously known (at least at certain growth stages). O’Connor et al. (2015) show evidence of a growth strategy similar to modern birds in the derived ornithuromorph *Iteravis huchzermeyeri*. Wang et al. (2019) conclude the same for the ornithuromorph *Yanornis*. In an ontogenetic study of *Confuciusornis*, Chinsamy et al. (2020b) report that this taxon likely experienced rates of growth comparable to some modern birds for a time in early-to mid-ontogeny, and furthermore present evidence that *Confuciusornis* retained a degree of developmental plasticity that allowed this taxon to phenotypically respond to environmental cues. Atterholt et al. (2021) report on fibrolamellar and incipient fibrolamellar bone in at least some skeletal elements of a Late Cretaceous skeletally-mature enantiornithine. A growing body of evidence supports the idea that fast, yet intermittent growth is the plesiomorphic condition for Avialae, and that a complex evolutionary pattern of both losses and amplifications of these features appears in Mesozoic and extant members of this clade (Prondvai et al., 2018; Wang et al., 2019; Chinsamy et al., 2020b; Wang et al., 2020).

Amid an increasing abundance of paleohistological studies on avialan and nonavialan dinosaurs, still relatively little is known about how bone develops in Aves, their closest living relatives. Bird bones are very thin-walled because resorption is a highly active process in avian bone growth, erasing most ‘history’ of development, so adult osteohistological features reveal very little about earlier growth stages. We instead must rely on ontogenetic studies of bone histology, which fortunately are increasingly common. Post-natal histology of bird bone has been examined in several taxa, however, the vast majority of studies focus on paleognathous birds or galloanseriforms (Castanet et al., 1996; Rath et al., 1999; Castanet et al., 2000; de Margerie, Cubo & Castanet, 2002; Skedros & Hunt, 2004; Turvey & Holdaway, 2005; Kuehn et al., 2019). The former are of particular interest likely because they are the early-branching clade of Aves, and thus are often perceived as being more representative of dinosaurian growth. Galloanseriforms understandably draw the attention of researchers because of their importance in the poultry industry, and because specimens are readily available since they are domestically bred and farmed. Comparatively few studies have investigated histological characteristics outside of these early-diverging clades (de Margerie et al., 2004; Watanabe, 2018; McGuire et al., 2020).

**Figure 1:**
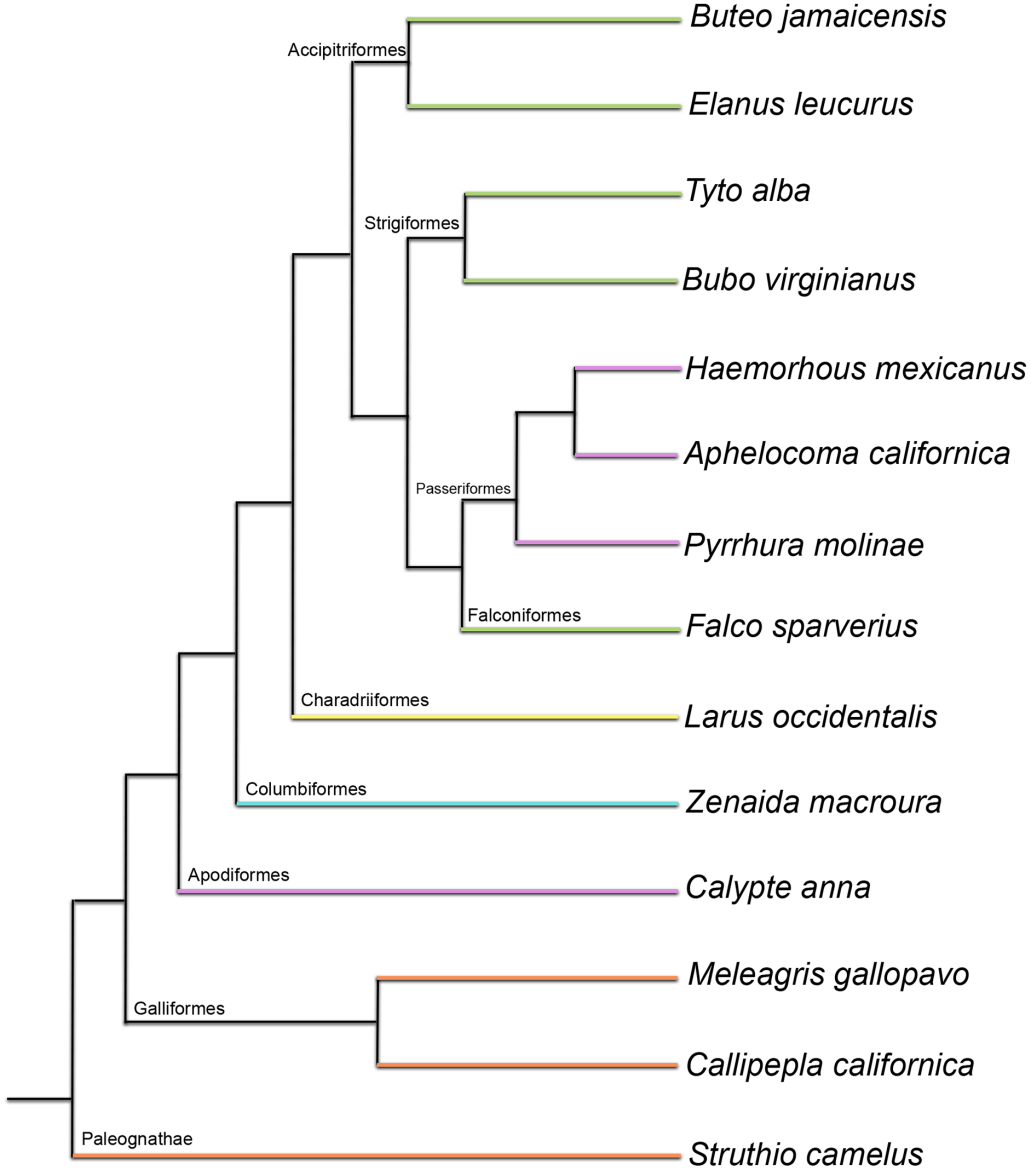
Phylogenetic tree used as evolutionary context in this study (topology based on Prum et al. (2015); branch lengths shown here do not reflect time calibration of the original tree). Colors represent developmental mode of each taxon: orange, precocial; yellow, semi-precocial; green, semi-altricial 1; blue, semi-altricial 2; purple, altricial. See Supplemental Table 2 for a break-down of traits used to define each category.

Here, we present a histological description of post-natal skeletal development of the humerus and femur in 14 taxa representing a phylogenetically diverse sampling of ten major clades: Struthioniformes, Galliformes, Apodiformes, Columbiformes, Charadriiformes, Accipitriformes, Strigiformes, Psittaciformes, Falconiformes, and Passeriformes (Fig. 1; Table S1). Specifically, we describe periosteal growth that occurs after a chick hatches, assessing patterns qualitatively and quantitatively, and making inter-and intrataxonomic comparisons. Bone is a highly complex tissue, and its growth and development are influenced by a variety of complicated functional and phylogenetic factors. In the case of birds, this includes the challenge of adapting a skeleton simultaneously to aerial locomotion and terrestrial locomotion (or at least the demands of weight-bearing when perched). This is further complicated by shifting demands on bones throughout ontogeny, as degree of locomotion and locomotion type generally shift dramatically through development as chicks fledge and take to flight. Previous studies began identifying the specific ways in which phylogeny and function influence bone histology (Cubo et al., 2005; Montes, Castanet & Cubo, 2007; Cubo et al., 2008; Montes, Castanet & Cubo, 2010; Legendre et al., 2013; Padian & Lamm, 2013; Legendre et al., 2014; Padian & de Ricqlès, 2020). A major goal of this study is to further identify aspects of bone microstructure affected by these two channeling mechanisms, as well as by developmental constraint.

**Table 1:** Summary of sampling across growth stages for each taxon (number of specimens per growth stage), and timing of the first appearance of an incipient OCL (light red) and a distinctly-formed OCL (dark red).

	Neonate	Downy	Pin-feathered	Pre-fledgling	Fledgling	Sub-adult	Adult
California^1^ Quail	8	—	2	—	—	—	2
Wild Turkey^1^	1	—	—	1	—	—	1
Western Gull^2^	2	—	—	—	—	—	2
American Kestrel^3^	2	—	—	—	1	—	2
Red-tailed Hawk^3^	—	1	—	1	—	—	2
White-tailed Kite^3^	—	—	1	2	3	—	1
Barn Owl^3^	—	1	2	2	1	1	2
Great-horned Owl^3^	—	—	1	1	1	—	1
Mourning Dove^4^	—	—	1	2	2	2	1
Anna’s Hummingbird^5^	—	—	1	—	1	3	1
Green-cheeked conure^5^	1	1	1	1	1	—	1
Western Scrub-Jay^5^	1	—	1	3	2	1	—
House Finch^5^	1	—	1	2	1	—	1

**Note:**

This indicates the first appearance of this structure in each taxon over all, either in the humerus and/or the femur. Developmental mode is indicated by color number superscript: orange^1^, precocial; yellow^2^, semi-precocial; green^3^, semi-altricial 1; blue^4^, semi-altricial 2; purple^5^, altricial. Six ostrich chicks also comprise part of the dataset, but are not included here because (beyond neonate an adult) they do not fit into the growth stages used for other taxa.

The final major objective of this investigation is to study ontogenetic histological patterns across the altricial-precocial spectrum. Birds, like mammals, are notable for the range of morphologies and behaviors they display at hatching (or birth), described as the altricial-precocial spectrum (Starck & Ricklefs, 1998). Neonatal chicks and their parents exhibit a full range of intermediate behavioral and morphological characteristics. At the precocial extreme of the spectrum, neonates are independent of their parents at hatching and even possess contour feathers and flight capabilities; here, there is no parental care after the eggs hatch. At the opposite end of the spectrum, the most altricial chicks hatch naked and with closed eyes. They remain nest-bound for the first few weeks of life, and are entirely dependent upon their parents for food, protection, etc. Various discretizations of the spectrum exist, with minor differences in the names and number of groups used to break it down into various developmental modes (*e.g*., Portmann, 1935; Nice, 1962; Skutch, 1976; Starck, 1993), but all rely on traits such as whether neonate eyes are open or closed, if chicks hatch naked or with feathers (and, in the latter case, what type of feathers), locomotor activity of the chick, and degree of parental care (*e.g*., showing to food, direct feeding, brooding, etc.) Here, we adopt the categories of developmental mode outlined by Starck & Ricklefs (1998), summarized in Table S2.

Of particular relevance to this investigation is evidence from previous studies showing that precocial chicks have growth rates much lower than those observed in altricial chicks (Ricklefs, 1968; Ricklefs, 1973; Starck, 1989; Starck, 1993; Starck & Ricklefs, 1998). Because growth rates, along with functional and physiological demands, vary so greatly across this spectrum, it is reasonable to expect substantial osteohistological variation in neonates of different developmental modes. Furthermore, identification of histological features that correlate with different developmental modes could be used as proxies for developmental mode in extinct taxa.

In studies of bone growth in the chicks of California gulls Carrier & Leon (1990) provided evidence that weak skeletal tissue (which they define to include a woven matrix) in very young individuals can be partially compensated for by increasing cortical thickness. Therefore, relative to other birds, gull chicks have very thick cortical walls in the femur because they begin locomoting terrestrially very soon after hatching, but the humerus has a thinner cortical wall until fledging, when the wing is finally used in flight. These data suggest certain predictions about cortical wall thickness of the two major limb elements in chicks at hatching, given information on developmental mode (whether or not the chicks are active at hatching) and primary locomotor module (whether they will ultimately use primarily their wings, legs, or both to locomote), and the correlation between the two. Dial (2003) identified the primary locomotor module of adults of more altricial taxa as the pectoral limb, and of precocial taxa as the pelvic limb. Additionally, chicks on the altricial end of the spectrum do not locomote at all until weeks after hatching, while precocial chicks can generally walk independently very soon post-hatching. Therefore, altricial and semi-altricial chicks should have thin-walled bones in both the humerus and the femur or a slightly thicker-walled humerus (if investment in the elements of their primary locomotor module begins very early), and precocial and semi-precocial chicks (such as the gull) should have a thicker-walled femur.

Similarly, a difference in bone maturity between pelvic and pectoral limbs was reported by Dial & Carrier (2010) in Mallard ducks, who found that the functional maturity of the hindlimb was much greater than that of the forelimb through most of post-natal ontogeny, up until the time of fledging. Prondvai et al. (2020) also report a difference in cortical apposition rates related to developmental timing of the bone of limb elements in ducks. Evidence from such studies suggests that a difference in bone maturity is linked to the precocial and semi-precocial developmental mode of these taxa; such chicks locomote using the pelvic limb from the time of hatching, but do not require the pectoral limb for flight until much later. It follows, therefore, that these earlier functional demands on the pelvic limb have led to selection for this locomotor module maturing at an earlier time.

Therefore, we have a clear hypothesis regarding a histological signal of developmental mode: more precocial chicks should have more mature femoral bone, defined here as possessing a mosaic of various features indicating relatively more growth has occurred (a thicker cortex, more organized tissue, smaller vascular openings, and/or thicker woven bone trabeculae). In contrast, chicks closer to the altricial end of the spectrum should have more mature humeral bone than femoral (or at least of equal maturity and thickness, if delayed locomotion is not enough to select for a functional difference in neonate limb bones). The range of developmental modes covered by the taxa in this dataset present an excellent opportunity for testing this hypothesis.

## Materials & methods

### Acquisition of specimens

Taxa for which complete or partial post-natal growth series were collected were included in the study. A post-natal growth series was defined as ranging from neonate chick to somatically mature adult (*i.e.*, final size, after which little or no morphological change takes place); in some instances, individuals of intermediate ages were also available. Ultimately, 14 taxa from 10 avian families were used (Fig. 1; Table 1; Table S1), comprising 89 specimens and generating 356 histological thin sections. Because a major goal of this study was to acquire specimens representing a phylogenetically broad sampling of Aves, specimen acquisition was opportunistic and dependent upon donations from the OK Corral Ostrich Farm in Oro Grande, CA, USA (the source of the ostriches); Avian Resources in San Dimas, CA, USA (the source of the green-cheeked conures); the Lindsay Wildlife Museum in Walnut Creek, CA and the Society for Prevention of Animal Abuse in Monterey Co., CA, USA for all other taxa. Beyond the broad phylogenetic sampling that this method of specimen acquisition allows, another strong benefit is that most of the birds in the dataset (the native Californian taxa accumulated from local wildlife hospitals) were all wild birds that hatched and lived in their natural habitat and conditions, where they would have been subject to normal behavioral and biomechanical impacts on the growing skeleton; only the two taxa not endemic to California (ostriches and green-cheeked conures) were hatched and raised by the breeder/farmer.

All specimens were dissected and skeletonized at the Museum of Vertebrate Zoology (MVZ) at the University of California, Berkeley. Skeletons, histological slides (plus residual tissue blocks), and frozen soft-tissue samples all are housed in the MVZ.

### Tissue processing and osteohistological slide preparation

This study examines bone as a mineralized tissue and is not an investigation of the process of ossification. Therefore, tissue samples were harvested from the youngest individuals with ossified diaphyses; in some cases, neonate bone was suitable, but in several altricial taxa neonate chicks were too small and had bones still too cartilaginous to be appropriate for the methods employed. By the time of hatching, most neonates have fully ossified bony diaphyses of the femur and humerus with cartilaginous proximal and distal ends, so tissue samples were taken at the mid-shaft portion of each element.

The humerus and femur were obtained from skeletonized individuals, and the mid-shaft regions harvested. Whenever possible, the left elements were used, though breakage or missing bones sometimes necessitated use of right elements. This investigation limited focus to the humerus and femur because these are two bones that, at their midshafts, undergo minimal secondary growth and remodeling, and therefore preserve a clear ontogenetic record (Padian & Lamm, 2013).

Tissue samples were fixed in 10% neutral buffered formalin for 48 h (with one change of solution after the first 24), then transferred to a solution of 70% ethyl alcohol for 48 h (with one change of solution after the first 24), subsequently placed in a solution of 85% ethyl alcohol for 48 h (with one change of solution after the first 24), and finally cleared in Histo-Clear (National Diagnostics) for 4–8 h, contingent upon the size of the tissue sample. Bone samples were then embedded in Epothin and sectioned to one mm wafers using a diamond-embedded saw. Wafers were mounted on glass slides and ground to approximately 100 µm thickness using a lap grinder and grit paper. Two sections from each bone were retained, and one of these mounted sections was stained in a 0.7% solution of toluidine blue. Finally, coverslips were applied using Permount (Fisher Chemical, Waltham, MA, USA). Slides were photographed using a Nikon digital sight camera and petrographic microscope (DS-U3 and DS-Fi2), and captured using the computer program NIS-Elements (F4.00.00). Sections were visualized under regular light and with cross-polarized light (XPL). Measurements were taken using ImageJ (1.48v).

### Experimental design & terminology

Precise ages of most individuals at time of death are unknown due to the method of specimen collection. Therefore, in cases where exact numerical age was missing, growth series were divided into the following qualitative growth stages, modified from identifications made by the wildlife hospitals and based on body size and general external morphology (primarily the condition of the feathers): neonate, downy chick, pin-feathered chick, pre-fledgling chick, fledgling chick, sub-adult, and adult. These categories were used to describe all taxa except for the ostrich, because these birds do not neatly fit into these categories. We note that, though these are artificial categories that fall along a spectrum, they roughly approximate the stages of feather development described by Prum & Brush (2002). Comparisons among intermediate growth stages are generally limited, and intertaxonomic comparisons are focused on adults and neonates (the two most ‘equal’ growth stages).

Due to decomposition and/or immaturity of specimens, sex was indeterminable by dissection for many specimens in the dataset. However, this is not considered a serious impediment to the study. The only major recorded histological difference in bone between male and female birds is medullary bone, variably present in adult females (Bonucci & Gherardi, 1975; Miller & Bowman, 1981; Van de Velde, Vermeiden & Bloot, 1985; Dacke et al., 1993).

This project is primarily descriptive. Each growth stage is described qualitatively in terms of general bone type, cross-sectional shape, variability of cortical thickness, density and orientation of blood vessels, density and shape of osteocyte lacunae, and presence of primary and secondary osteons. Osteocyte lacunal volume and density is known to be extremely variable intrataxonomically, among skeletal elements, and even regionally within a single bone, and is most accurately estimated when using multiple thin sections (D’Emic & Benson, 2013); therefore while a qualitative description of osteocyte lacunae is included in this study, we do not quantify this feature or infer relative rates of growth and metabolism.

The terminology used to qualitatively classify and describe bone tissues follows the precedent of Francillon-Vieillot et al. (1990) and de Ricqlès (1975), as updated by Huttenlocker, Woodward & Hall (2013). Bone is described both in terms of matrix classification (based on fiber organization), and vascular classification (based on type and orientation of vascular channels).

The altricial-precocial spectrum has been divided into a number of categories based on morphological and behavioral features by multiple authors (see Starck & Ricklefs, 1998); here, we used the Starck & Ricklefs (1998) reorganization of Nice’s (Nice, 1962) developmental classes. Starck and Ricklefs’ categories are summarized in Table S2.

Bone histology was also analyzed quantitatively; all statistical tests were run using the program R (v4.0.3). For each section, average cortical thickness and average total cross-sectional diameter were measured in µm. The cortical thickness was an average of eight measurements evenly distributed across the cortex (midpoints of the cranial, caudal, medial, and lateral regions, and positions equidistant between these). Average total diameter of each sample was taken as an average of four measurements of the total diameter of the cortex, along the craniocaudal and mediolateral axes. and the two axes at the mid points between these. These data were used to calculate ratios for comparisons of relative cortical thickness as 2 (cortical thickness)/total diameter.

Cortical thickness is analyzed and discussed in two ways: absolute cortical thickness (ACT) and relative cortical thickness (RCT). These ratios together with average measures of cortical thickness and cortical diameter can be found in Table S1. A log10 transformation was applied to measures of absolute cortical thickness (to correct for strong allometry as associated with body size) and to relative cortical thickness (to normalize the data, which had an exponential distribution). General patterns of variation and changes in cortical width (by growth stage and intertaxonomically) are visualized and described using boxplots. Phylogenetic signal was tested for in ACT and RCT of the humerus and femur of adults and neonates, as well as in developmental mode, using Blomberg’s K test statistic (Blomberg, Garland & Ives, 2003). The phylogenetic topology used for this test is based on the tree in Prum et al. (2015), using the same calibrations as the original published phylogeny. Finally, regression tests were implemented to assess intertaxonomic relationships (with relevant metrics from all individuals pooled into a common dataset) between cortical thickness of the adult humerus and femur, cortical thickness of the neonate humerus and femur, and developmental mode with cortical thickness of each element for neonates. Specific models (and results for each) are described in Tables 2 and 3. For these tests, the developmental spectrum was treated as a pseudo-continuous ordinal predictor (Symonds & Blomberg, 2014) and numerically discretized (based on the categorization of Starck & Ricklefs (1998) as follows: 1, precocial; 2, semi-precocial; 3, semi-altricial 1; 4, semi-altricial 2; 5, altricial. A phylogenetic generalized least squares regression (PGLS) was used in cases when model residuals had phylogenetic signal; in all other cases, an ordinary least squares regression (OLS) was used.

**Table 2:** Results of tests for phylogenetic signal in developmental mode and cortical thickness of humeri and femora of adults and neonates, using Blomberg’s *K* test-statistic.

Parameter tested	*K* Statistic	*P-*value
Adults, Humerus (ACT)	0.804	0.591
Adults, Femur (ACT)	1.060	0.197
Neonates, Humerus (ACT)	0.804	0.613
Neonate, Femur (ACT)	1.061	0.207
Adults, Humerus (RCT)	0.860	0.433
Adults, Femur (RCT)	1.101	0.089
Neonates, Humerus (RCT)	0.813	0.689
Neonate, Femur (RCT)	1.235	0.043*
Developmental Mode	1.317	0.005*
log10(NHACT) ~ log10(NFACT) + log10(NBM)	1.286	0.028*

**Note:**

ACT, absolute cortical thickness; RCT, relative cortical thickness; measured as the ratio of average cortical thickness to average total cross-sectional diameter. The final row shows results of a test for phylogenetic signal in the residuals from a PGLS analysis of the model described (NHACT, neonate humerus ACT; NFACT, neonate femur ACT; NBM, neonate body mass). Significant *P-*values are indicated by asterisks.

**Table 3:** Results of regression analyses between adult and neonate humerus and femur cortical thickness, and developmental mode.

Regression Model	*F* Ftatistic	*P*-value (*F* Statistic)	Adjusted R^2^	*P*-value (Adjusted *R*^*2*^)
log10(NHACT) ~ log10(NFACT) + log10(NBM)	0.81; 0.96	0.466; 0.392	0.65	0.054
log10(AHACT) ~ log10(AFACT) + log10(ABM)	5.53; −0.40	0.007 × 10^−4*^; 0.692	0.91	0.002 × 10^−5*^
log10(AHRCT) ~ log10(AFRCT)	—	—	0.23	0.029
log10(NHRCT) ~ log10(NFRCT)	—	—	0.15	0.095
DM ~ log10(NHACT) + log10(NBM)	0.45; −0.75	0.660; 0.468	−0.11	0.709
DM ~ log10(NFACT) + log10(NBM)	3.06; −3.15	0.051; 0.023*	0.39	0.050
DM ~ log10(NHRCT)	—	—	−0.03	0.442
DM ~ log10(NFRCT)	—	—	−0.06	0.657

**Note:**

The first model was assessed using PGLS. All other models were assessed using OLS. ACT, absolute cortical thickness; RCT, relative cortical thickness; measured as the ratio of average cortical thickness to average total cross-sectional diameter. Significant *p*-values are indicated by asterisks. Abbreviations used in models: NHACT, neonate humerus ACT; NFACT, neonate femur ACT; NBM, neonate body mass; AHACT, adult humerus ACT; AFACT, adult femur ACT; ABM, adult body mass; AHRCT, adult humerus RCT; AFRCT, adult femur RCT; NHRCT, neonate humerus RCT; NFRCT, neonate femur RCT; DM, developmental mode.

## Results

Broad intertaxonomic patterns and comparisons are reported here; for a detailed description of each individual and intrataxonomic comparisons, please see Supplementary Material.

### General observations of variation in cortical thickness

**Figure 2:**
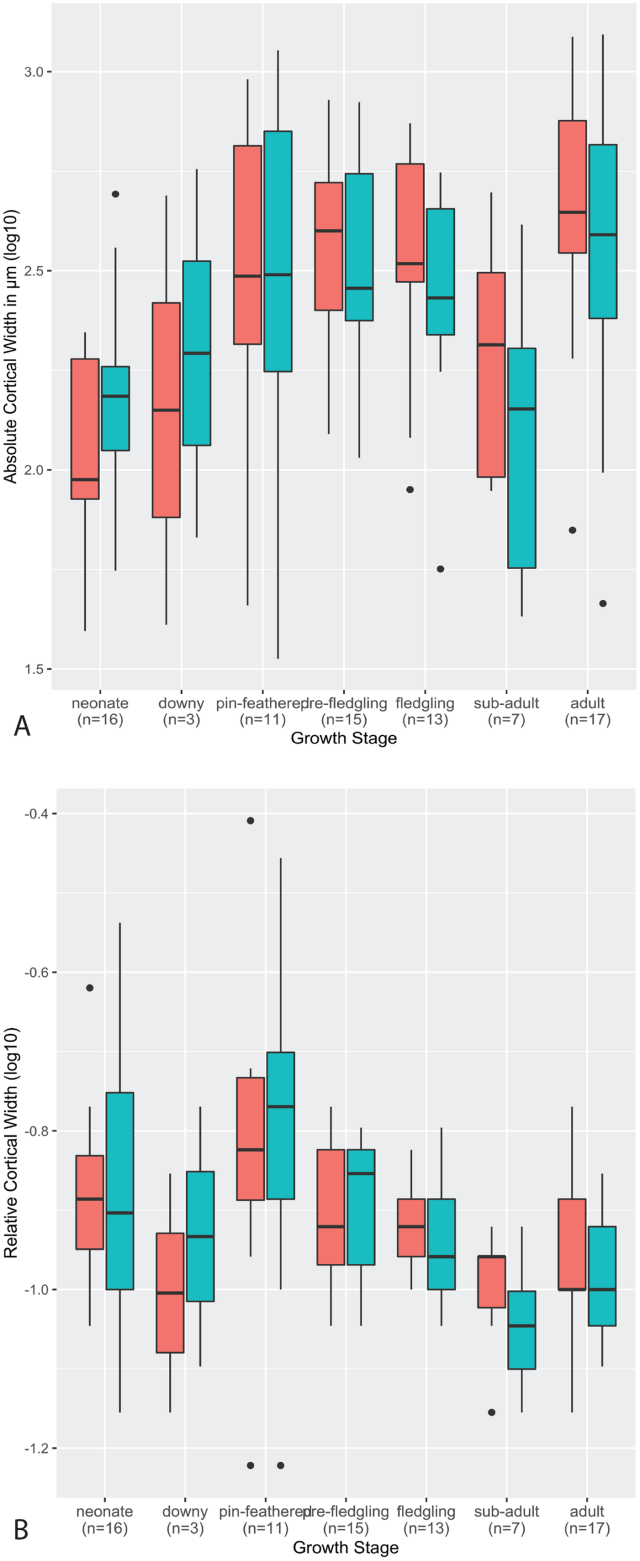
Boxplots showing the variation of cortical thickness by growth stage, where red represents measurements of the humerus and green measurements of the femur. (A) Absolute cortical thickness (ACT). (B) Relative cortical thickness (RCT). Within each growth stage, all samples (from the humerus and femur) across all taxa are included, excepting ostriches (which were not categorized using these growth stages). Boxes = interquartile range, or values between the 25th and 75th percentiles; horizontal black bars = median values; whiskers = minimum and maximum values, not including outliers; circles = outliers.

**Figure 3:**
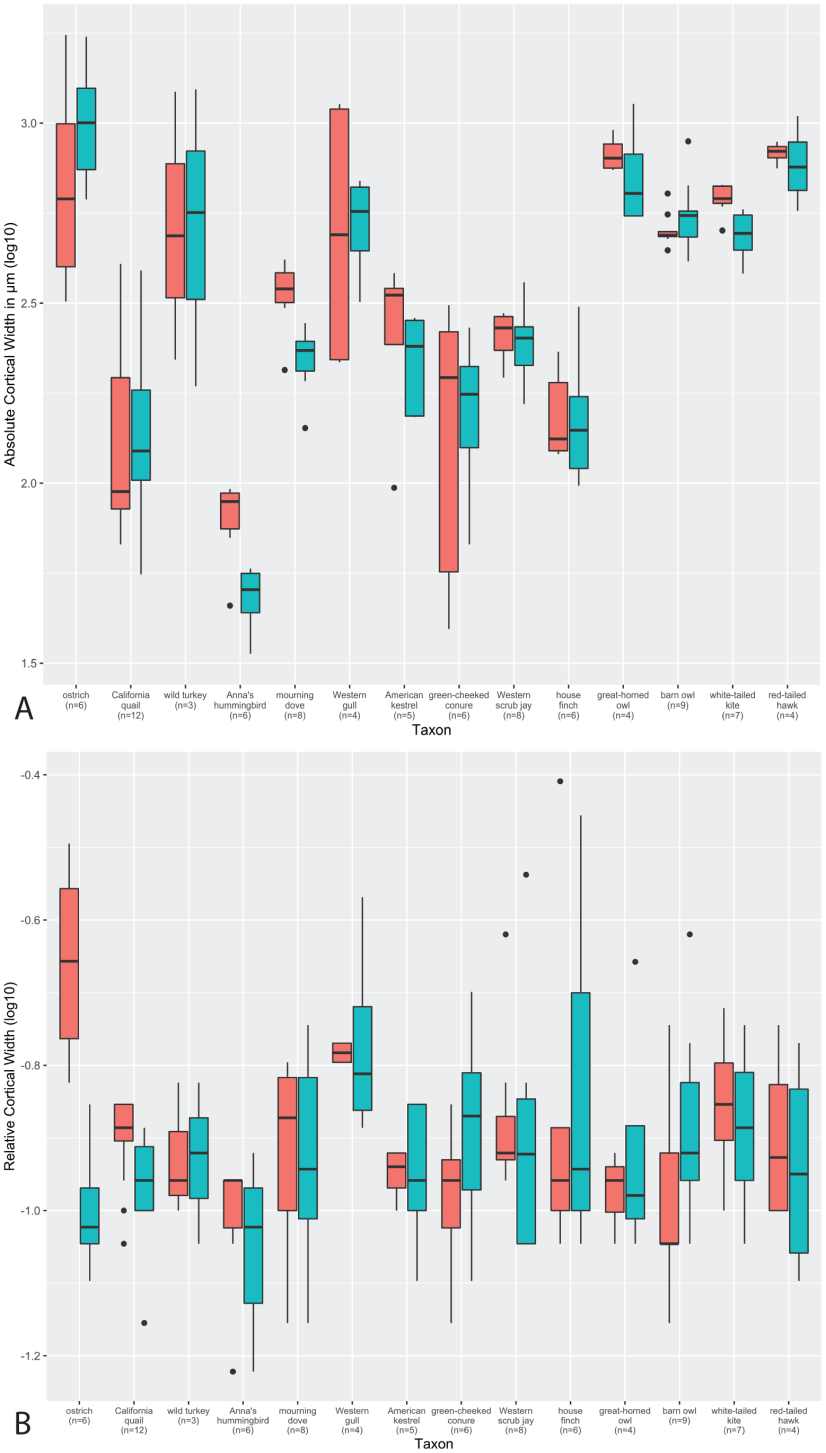
Boxplots showing variation in cortical thickness by taxon, where red represents measurements of the humerus and green measurements of the femur. (A) Absolute cortical thickness (ACT). (B) Relative cortical thickness (RCT). For each taxon, all specimens at all growth stages are represented. Boxes = interquartile range, or values between the 25th and 75th percentiles; horizontal black bars = median values; whiskers = minimum and maximum values, not including outliers; circles = outliers.

**Figure 4:**
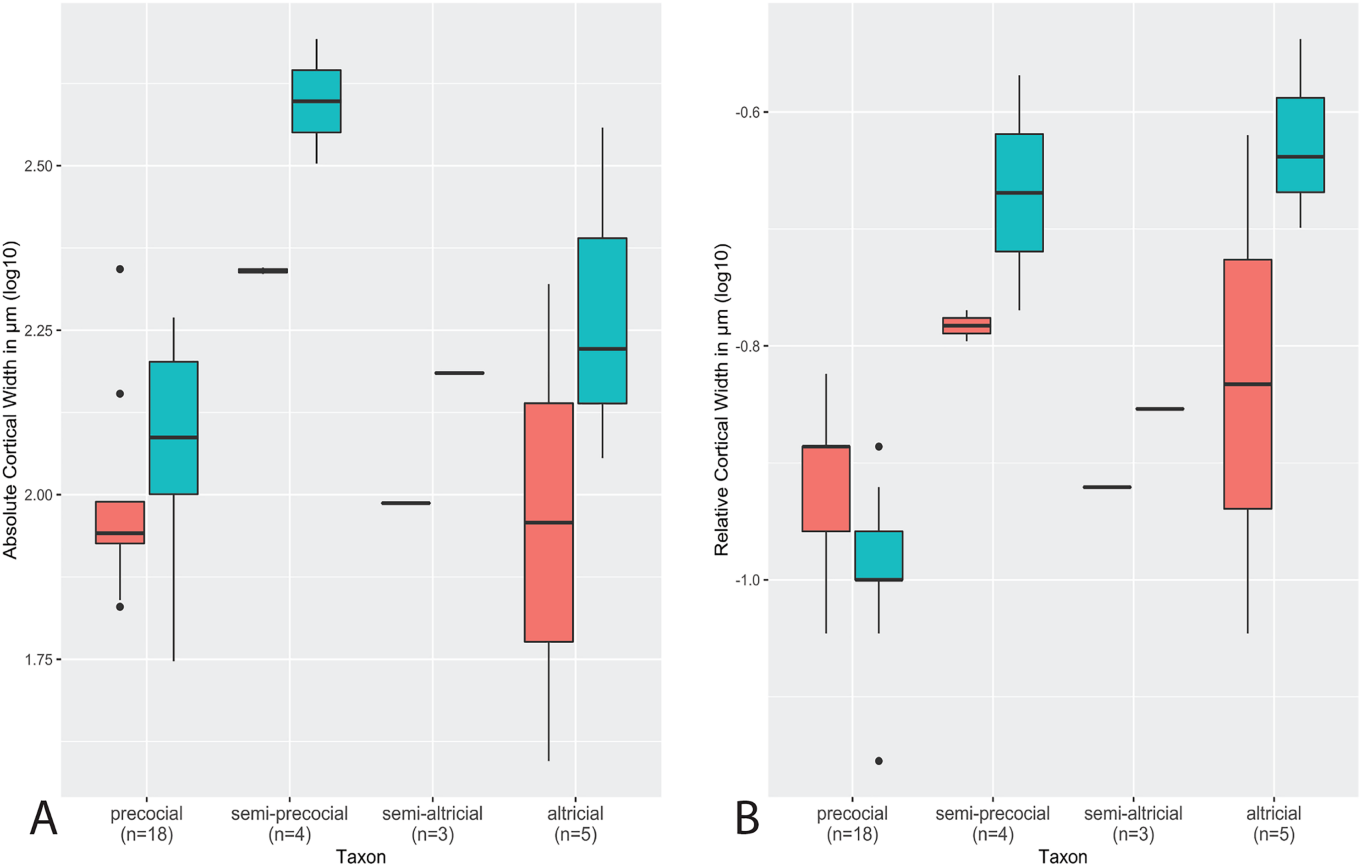
Boxplots showing variation in cortical thickness by developmental mode, where red represents measurements of the humerus and green measurements of the femur. (A) Absolute cortical thickness (ACT). (B) Relative cortical thickness (RCT). For each group, the humerus and femur of all neonate specimens within a particular developmental mode are represented. Additionally, “semi-altricial” here refers to “semi-altricial 1;” the elements of the semi-altricial 2 neonate were not ossified and thus not examined in this study. Boxes = interquartile range, or values between the 25th and 75th percentiles; horizontal black bars = median values; whiskers = minimum and maximum values, not including outliers; circles = outliers.

The thickness of the cortex, both on an absolute and relative scale, was highly variable across growth stages, taxa, and developmental mode (Figs. 2–4). Analysis of both RCT and ACT is included in the interest of conducting a thorough study, and because in some instances each metric reveals interesting patterns that the other does not. Absolute cortical thickness appears to undergo an overall trend of increase in early ontogeny to a maximum thickness around the pin-feathered stage, a subsequent decrease from the fledgling to sub-adult stages, and final moderate increase to adult cortical thickness (Fig. 2A). Interestingly, such a trend was also frequently observed at the scale of individual taxa (see Supplementary Material), though this more nuanced and detailed description also revealed a frequent pattern of a secondary increase at the fledgling stage (rather than a uniformly high ACT from pin-feather to fledgling stages). The pin-feathered stage exhibits the highest variance in both elements, both overall and for the interquartile range. In most growth stages, variance of the humerus and femur are similar in magnitude. Most measures of ACT fall within a narrower range of values for the femur than the humerus in neonates and adults. In pre-fledgling chicks, the humerus appears to have a more restricted range of ACT. In neonates, ACT of the femur is generally greater than in the humerus; this relationship is inverted in adults, where ACT of the humerus is generally higher.

Ontogenetic changes in RCT reflect the overall patterns observed in ACT (Fig. 2B): an increase in early ontogeny peaking at the pin-feathered stage, followed by a decrease, and final moderate increase from sub-adult to adult. However, the early increase is disrupted by a reduction in RCT from the neonate to downy stages before reaching a maximum at the pin-feathered stage. At hatching, neonate RCT is more variable in the femur than the humerus. Neonate median values of RCT are higher in the humerus than femur, but the medians are equal at the adult stage. Highest RCT is reached at the pin-feathered chick stage. The greatest individual value is seen in the humerus of the pin-feathered house finch chick (RCT = 0.78). However, this stage also has the highest variance, so certainly not all pin-feathered chicks have a very thick cortex. Anna’s hummingbird pin-feathered chicks had the lowest RCT in the dataset (RCT = 0.12 in the humerus and femur). Overall variance and interquartile variance generally tend to decrease through ontogeny.

Predictably, absolute cortical thickness is highest in ostriches, and lowest in Anna’s hummingbird, reflecting the body size extremes represented in this dataset (Figure 3A). However, the wild turkey, Western gull, great-horned owl, red-tailed hawk, barn owl, and white-tailed kite all have high ACT, within range of the values observed in the ostriches (though the dataset notably is lacking samples from an adult ostrich). Other taxa appear to roughly group according to medium values of ACT (California quail, mourning dove, American Kestrel, green-cheeked conure, and house finch). These general groupings also parallel body size differences, in spite of the log transformation of the data.

In many taxa (great-horned owl, red-tailed hawks, Anna’s hummingbird, white-tailed kites, American kestrels, barn owls, scrub jays, and house finches), the femur exhibits higher variance overall in ACT than the humerus. Variance in ACT is greater in the humerus than the femur in Western gulls, green-cheeked conures, and ostriches, and roughly equal between the two elements in California quail, wild turkeys, and mourning doves. Highest levels of variance in ACT are seen in the California quail, Western gull humerus, wild turkey, green-cheeked conure, and ostrich humerus (Fig. 3A). It is striking that these taxa span a wide ranges of body sizes, primary locomotor module, and developmental mode. Variance of ACT is low in great-horned owls, barn owls, white-tailed kites, red-tailed hawks, mourning doves, and scrub jays, though the degree of difference in variation is quite possibly due to a lack of available samples representing the extremes of the growth stages (no adult for scrub jays, and no neonates for the rest).

Intertaxonomically, the highest values of RCT were observed in the humerus and femur of the house finch pin-feathered chick, where the cortex comprises 78% and 70% of total cross-sectional diameter, respectively. Taxa represented by more specimens at the pin-feathered and pre-fledgling stage exhibit higher variance than others, further underscoring the major increase in cortical thickness during these early growth stages (Fig. 3). Interquartile range and total variance for each element are close and usually overlapping in nearly all taxa. However, the RCT of ostrich chick humeri is much higher than that of the femora, reflecting the sharp differences in cortical maturity observed between the two elements in this taxon. The predominant pattern across nearly all other taxa is a higher variance of RCT in the femur, either drastically (in Anna’s hummingbirds, Western gulls, American kestrels, Western scrub jays, and house finches) or moderately (as in California quail, wild turkeys, mourning doves, green-cheeked conures, white-tailed kites, and red-tailed hawks). The single exception to this pattern is the barn owl, in which the humerus exhibits higher variance. We are cautious about interpreting these observations since intrataxonomic sample metrics would be altered by the addition of more specimens representing more growth stages in most if not all cases. However, the consistency of this pattern across a range of samples sizes, phylogenetic differences, and various developmental modes, is notable. Overall, however, the interspecific interquartile range of RCT falls within a relatively narrow span of values across taxa (especially compared to ACT); only the ostrich deviates from this trend, and only then with respect to the humerus. In the femur, this pattern is sustained when making intraspecific observations, but in notable contrast, intraspecific variation is generally greater in the RCT of the humerus than in ACT.

Distributions of ACT and RCT by developmental mode are presented in Fig. 4. The semi-precocial developmental mode appears to have the highest values of ACT for both elements (based on our limited sampling) (Fig. 4A). The greatest variance of ACT is observed in altricial neonates, which represent both the lowest and some of the highest values of ACT (excepting semi-precocial ACT). ACT of semi-altricial chicks appears to fall within the same range as that of altricial neonates. Interestingly, there is relatively little difference in ACT between the two extremes of the spectrum. ACT of the humerus in precocial chicks falls also within the range of measurements of humeral ACT for altricial neonates (though with a variance of a much lesser magnitude). Femoral ACT of precocial neonates also overlaps with the range observed in altricial chicks, though values are generally a bit higher in the latter group. Additionally, femoral ACT is higher than humeral ACT across the altricial precocial spectrum (in terms of variance, interquartile range, and median values), though when considering taxa on an individual basis this pattern is not born out in the wild turkey neonate.

In terms of RCT (Fig. 4B), altricial neonates have the highest values for both the humerus and femur, though precocial neonates fall within a very similar range. Once again, this developmental mode also has a variance of the greatest magnitude, for both elements. Notably, the RCT of precocial chicks is generally quite a bit lower than all other developmental modes. This is also the only group in which RCT is higher in the humerus than in the femur. Furthermore, values of RCT for semi-altricial chicks are closer to those for precocial neonates than altricial neonates.

### Phylogenetic tests

We tested for phylogenetic signal in average cortical width of the humerus and femur in the adult and neonate, as well as in developmental mode (Table 2); cortical width was analyzed both as an absolute measurement (ACT), and relative (RCT). Phylogenetic signal was not significant in ACT of either element for either growth stage. Significant signal was recovered in the RCT of the neonate femur, but not the neonate humerus. No significant phylogenetic signal was detected in RCT of either bone in adults. Developmental mode has significant phylogenetic signal, a result that has been reported by previous authors (Starck & Ricklefs, 1998; Atterholt, 2011).

Regression analyses were used to assess relationships between RCT and ACT in adult and neonate humeri and femora, comparing element to element within the same growth stage, and comparing each neonate metric individually to developmental mode (see results in Tables 2 and 3). The only model with significant phylogenetic signal was that assessing the relationship between ACT of the neonate humerus and femur (*K* = 1.286; *P* = 0.028). The PGLS results for this regression, however, were not indicative of a significant effect of femoral ACT or body mass on humeral ACT in neonates, either individually or together (Table 3). In contrast, ACT of the adult humerus and femur samples is strongly correlated (*R* = 0.91; *P* = 0.002 × 10^−5^). Furthermore, results indicate that body mass is not significantly related to humerus ACT when the effects of femur ACT is accounted for (*F* = −0.40; *P* = 0.692). However, RCT of the humerus is not significantly related to that of the femur in neonates or adults (Table 3).

None of the various measures of cortical thickness in neonates exhibit significant correlations with developmental mode, though we note this may be due to the relatively small sample size of neonates in this study. However, body mass has an effect even at the neonate growth stage in ACT of the femur, with larger-bodied chicks having thicker femoral cortices.

### Histology of neonates & other immature developmental stages

The wild turkey and Western gull neonates have the thickest humeral cortices as an absolute measure (220.28 and 218.98 µm respectively), and the conure the thinnest (39.38 µm) (Table 4). In terms of RCT (Table 5), the Western scrub-jay has the thickest humeral cortex at 48% total cross-sectional diameter; the conure has thinnest at 18% total cortical diameter. Other taxa fall within a range of 24–34%. Qualitative characteristics of neonate humeri are summarized in Table 6.

**Table 4:** Average absolute cortical thickness (ACT) of the humerus and femur for all adults and neonates used in this study.

Taxon	Neonate humeral cortical thickness	Neonate femoral cortical thickness	Adult humeral cortical thickness	Adult femoral cortical thickness
California Quail^1^ **(*Callipepla californica*)**	90.67	117.99	395.36	380.76
Wild Turkey^1^ **(*Meleagris gallopavo)***	220.28	185.92	1223.12	1240.14
Western Gull^2^ **(*Larus occidentalis*)**	218.98	405.71	1105.81	632.64
American Kestrel^3^ **(*Falco sparverius*)**	124.86	153.60	359.75	261.49
Red-tailed Hawk^3^ **(*Buteo jamaicensis*)**	no sample	no sample	800.49	625.18
White-tailed Kite^3^ **(*Elanus leucurus*)**	no sample	no sample	503.06	382.01
**Barn Owl** ^**3**^ **(*Tyto*** ***alba*)**	no sample	no sample	463.79	545.27
Great-horned Owl^3^ **(*Bubo virginianus*)**	no sample	no sample	752.79	736.66
Mourning Dove^4^ **(*Zenaida macroura*)**	unossified	unossified	350.61	229.60
Anna’s Hummingbird^5^ **(*Calypte anna*)**	unossified	unossified	70.55	46.18
**Green-cheeked conure** ^**5**^ **(*Pyrrhura molinae*)**	39.38	113.68	266.38	184.81
Western Scrub-Jay^5^ **(*Aphelocoma californica*)**	208.99	361.33	no sample	no sample
**House Finch** ^**5**^ **(*Haemorhous mexicanus*)**	unossified	166.63	190.34	98.44

**Note:**

Developmental mode is indicated by color number superscript: orange^1^, precocial; yellow^2^, semi-precocial; green^3^, semi-altricial 1; blue^4^, semi-altricial 2; purple^5^, altricial. All measurements are given in µm.

**Table 5:** Average relative cortical thickness as compared to the average total cross-sectional diameter (RCT) of the humerus and femur for all adults and neonates used in this study.

Taxon	Neonate humeral cortical thickness	Neonate femoral cortical thickness	Adult humeral cortical thickness	Adult femoral cortical thickness
California Quail^1^ **(*Callipepla californica*)**	0.24	0.22	0.28	0.26
Wild Turkey^1^ **(*Meleagris gallopavo)***	0.30	0.18	0.20	0.24
Western Gull^2^ **(*Larus occidentalis*)**	0.34	0.44	0.34	0.28
American Kestrel^3^ **(*Falco sparverius*)**	0.24	0.28	0.22	0.18
Red-tailed Hawk^3^ **(*Buteo jamaicensis*)**	no sample	no sample	0.20	0.18
White-tailed Kite^3^ **(*Elanus leucurus*)**	no sample	no sample	0.20	0.18
**Barn Owl** ^**3**^ **(*Tyto*** ***alba*)**	no sample	no sample	0.20	0.30
Great-horned Owl^3^ **(*Bubo virginianus*)**	no sample	no sample	0.18	0.22
Mourning Dove^4^ **(*Zenaida macroura*)**	unossified	unossified	0.20	0.20
Anna’s Hummingbird^5^ **(*Calypte anna*)**	unossified	unossified	0.18	0.18
**Green-cheeked conure** ^**5**^ **(*Pyrrhura molinae*)**	0.18	0.40	0.22	0.20
Western Scrub-Jay^5^ **(*Aphelocoma californica*)**	0.48	0.58	no sample	no sample
**House Finch** ^**5**^ **(*Haemorhous mexicanus*)**	unossified	0.46	0.26	0.18

**Note:**

Developmental mode is indicated by color number superscript: orange^1^, precocial; yellow^2^, semi-precocial; green^3^, semi-altricial 1; blue^4^, semi-altricial 2; purple^5^, altricial.

**Table 6:** Qualitative comparisons among neonate humeri.

Taxon	Cross-sectional shape	Bone composition & porosity	Primary vascular orientation	Other notable features
California Quail^1^ (*Callipepla californica*)	elliptical, flattened lateral margin	moderate, canals relatively small; bone predominates cortex	longitudinal	lateral edge thinner and with fewer vascular canals
Wild Turkey^1^ (*Meleagris gallopavo)*	rounded triangle	moderate and very uneven porosity	longitudinal	medial cortex much thicker than lateral, canals much more numerous in thicker regions
Western Gull^2^ (*Larus occidentalis*)	circular	high porosity, but woven bone still substantial	longitudinal/ irregular	some variation in cortical thickness, canals larger in thicker regions
American Kestrel^3^ (*Falco sparverius*)	circular	moderate porosity, substantial bone present	longitudinal/ irregular	very thin cortex; few vascular canals
Green-cheeked conure^4^ (*Pyrrhura molinae*)	circular	moderate porisity, substantial bone present	all orientations	extremely thin cortex; few vascular canals
Western Scrub-Jay^4^ (*Aphelocoma californica*)	circular	high; bony bone very thin	all orientations	solid endosteal circlet of bone present; highly asymmetrical cortical thickness

**Note:**

Note that vascular density was high almost ubiquitously among these individuals, therefore porosity is compared among neonates; nearly all have high porosity relative to adults. Results are shown for taxa for which neonate specimens were available, and in which the humerus was ossified and viable for this study (the humeri of the mourning dove, Anna’s hummingbird, and house finch were not included for the latter reason). Developmental mode is indicated by color number superscript: orange^1^, precocial; yellow^2^, semi-precocial; green^3^, semi-altricial 1; purple^4^, altricial.

**Figure 5:**
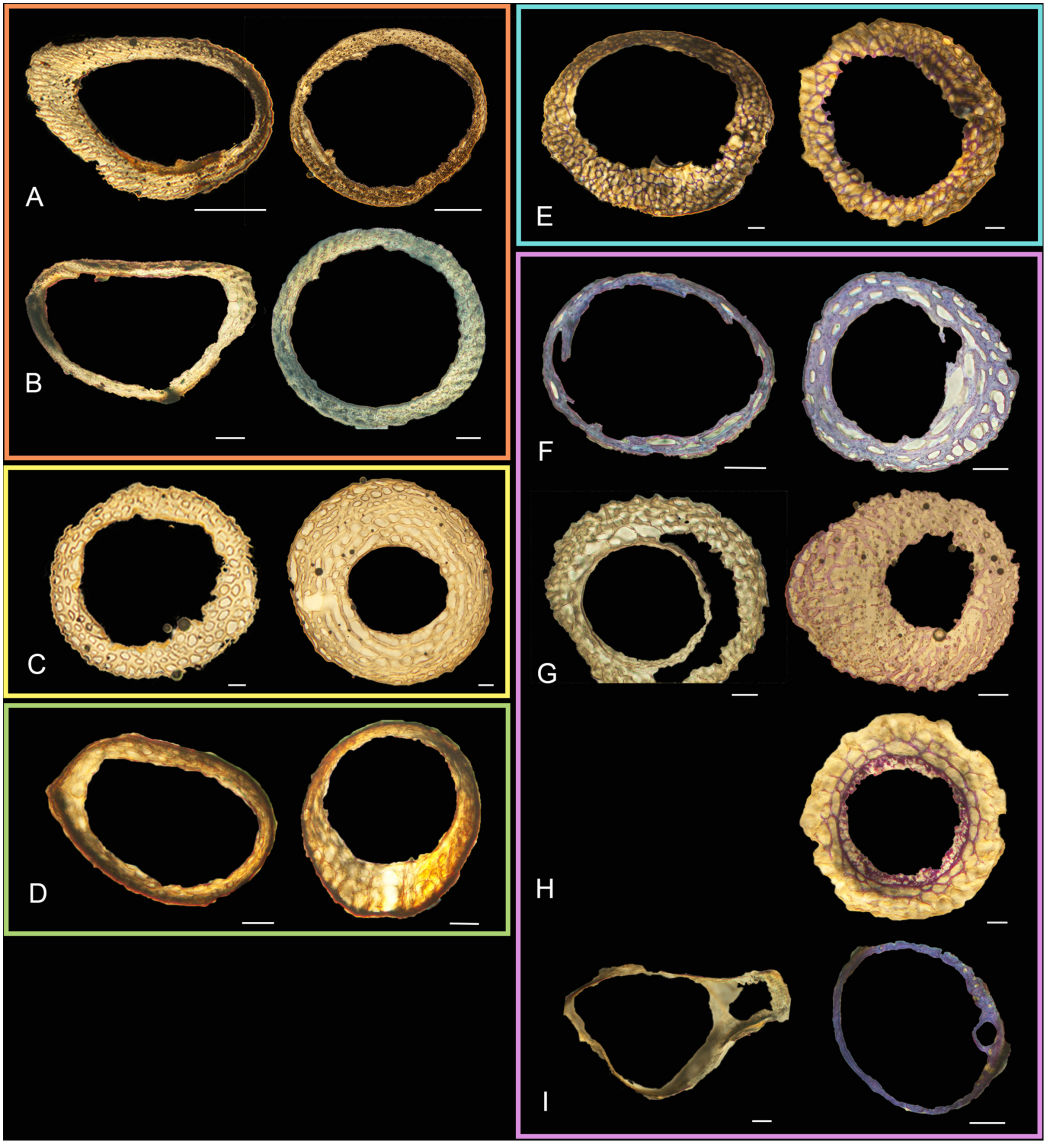
Diaphyseal cross-sections of youngest individuals with ossified humeral and femoral diaphyses included in this study, showing general cross-sectional geometry and variation in cortical thickness. All are neonates, except in the case of Anna’s hummingbird and mourning dove, shown here at the pin-feathered stage. Only femur of the neonate house finch was ossified, and therefore included in this study. For each pair, the humeral section is on the left and the femoral on the right. In all images cranial is up and lateral is left. Sections are grouped by developmental mode. Precocial (orange): (A) Wild turkey (*Meleagris gallopavo*; MVZ190764); (B) California quail (*Callipepla californica*; MVZ190745). Semi-precocial (yellow): (C) Western gull (*Larus occidentalis*; JAA264). Semi-precocial 1 (green): (D) American kestrel (*Falco sparverius*; MVZ190890). Semi-precocial 2 (blue): (E) mourning dove (*Zenaida macroura*; MVZ190778). Altricial (purple): (F) green-cheeked conure (*Pyrrhura molinae*; MVZ190895); (G) Western scrub jay (*Aphelocoma californica*; MVZ190927); (H) house finch (*Haemorhous mexicanus*; MVZ190969); (I) Anna’s hummingbird (*Calypte anna*; MVZ190799). Scale bars for the turkey sections = 500 µm. All other scale bars = 100 µm. For cross-sections of all ages, see figures in Supplemental Material.

**Figure 6:**
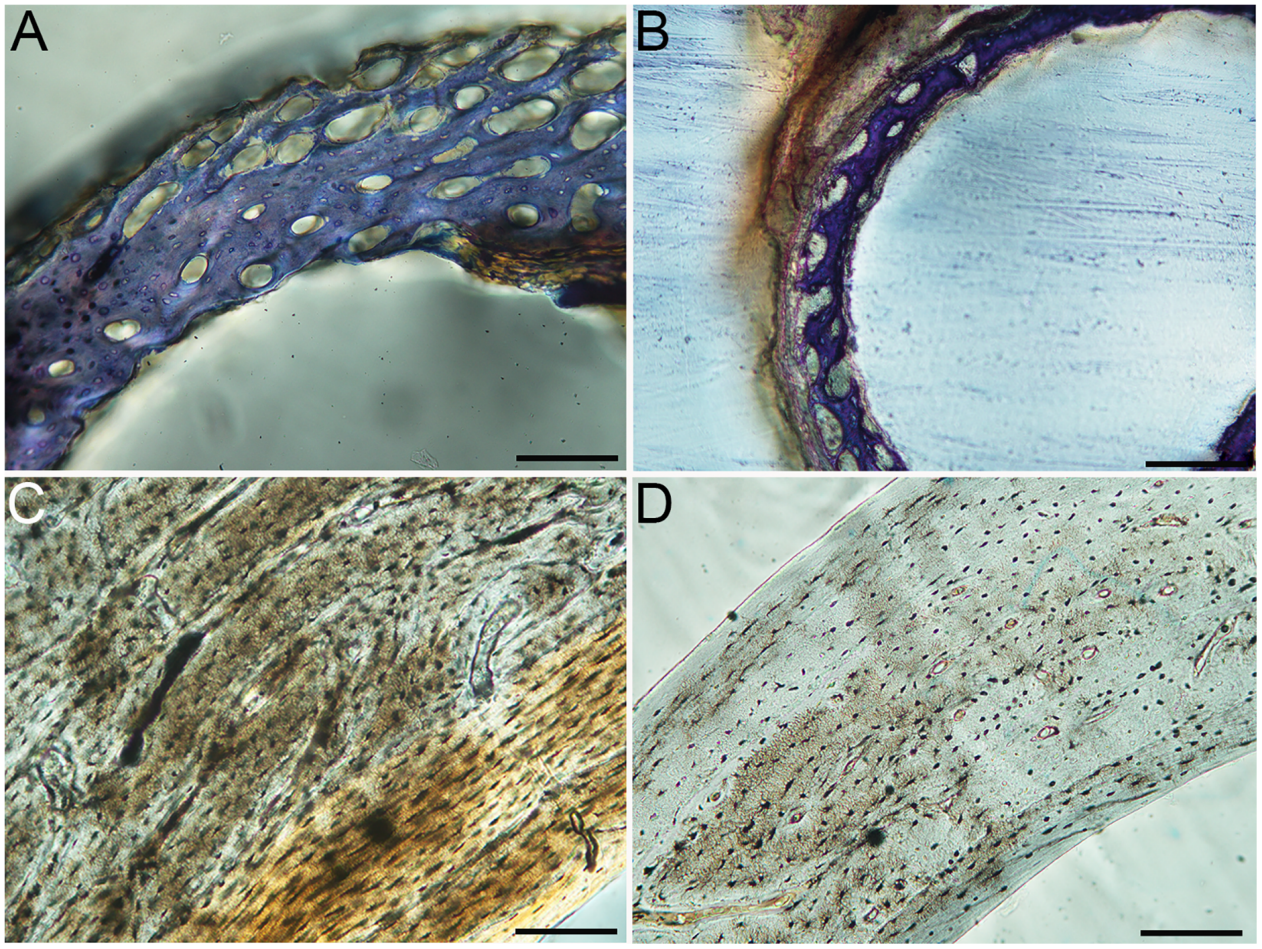
Close-up photographs of microanatomical details in the humeri of precocial birds, sections from neonates shown in the top row and from adults in the bottom row. (A) Wild turkey neonate (MVZ190764); (B) California quail neonate (MVZ190751); (C) wild turkey adult (MVZ190763), showing fibrolamellar bone of the middle cortical layer where it meets the ICL; (D) California quail adult (MVZ190762). In all images the periosteal surface is oriented to the upper left and the endosteal to the lower right. Scale bar = 100 µm.

**Figure 7:**
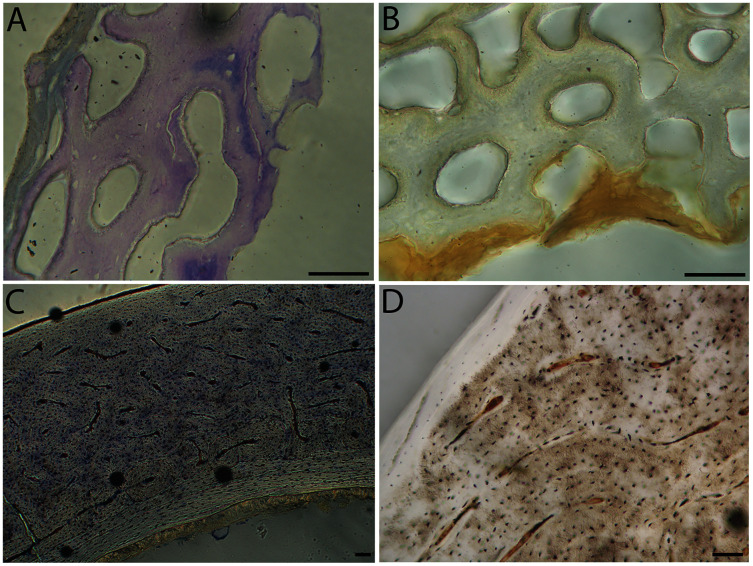
Close-up photographs of microanatomical details in the humeri of semi-precocial birds, sections from neonates shown in the top row and from adults in the bottom row. (A) Western gull neonate (JAA64); (B) Western gull neonate (MVZ190822); (C) Western gull adult (MVZ190829); (D) Western gull adult (MVZ190831). In all images the periosteal surface is oriented to the upper left and the endosteal to the lower right. Scale bar = 100 µm.

**Figure 8:**
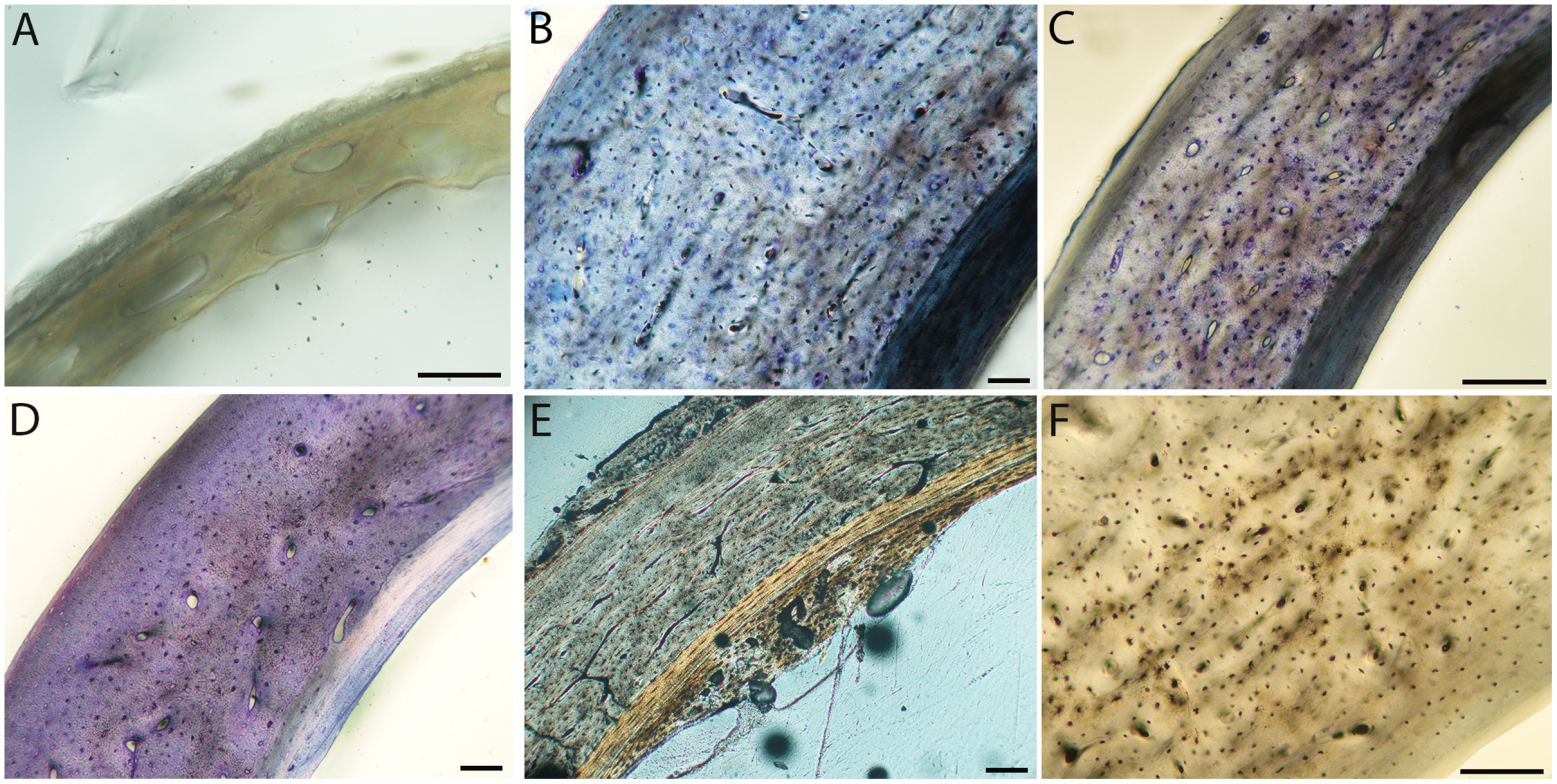
Close-up photographs of microanatomical details in the humeri of semi-altricial 1 birds. For this developmental mode, a neonate sample was only acquired for one taxon (the American kestrel), therefore this figure shows mainly adult histology. For descriptions and images of immature stages between neonate and adult, please see Supplemental Material. On the far left of the figure are samples of a neonate (MVZ190890) (A) and adult (MVZ190892) (D) American kestrel. Other panels show adult microanatomy: (B) red-tailed hawk (MVZ190855); (C) white-tailed kite (MVZ190861); (E) barn owl (MVZ190872); (F) great-horned owl (MVZ190883). In all images the periosteal surface is oriented to the upper left and the endosteal to the lower right. Scale bar = 100 µm.

**Figure 9:**
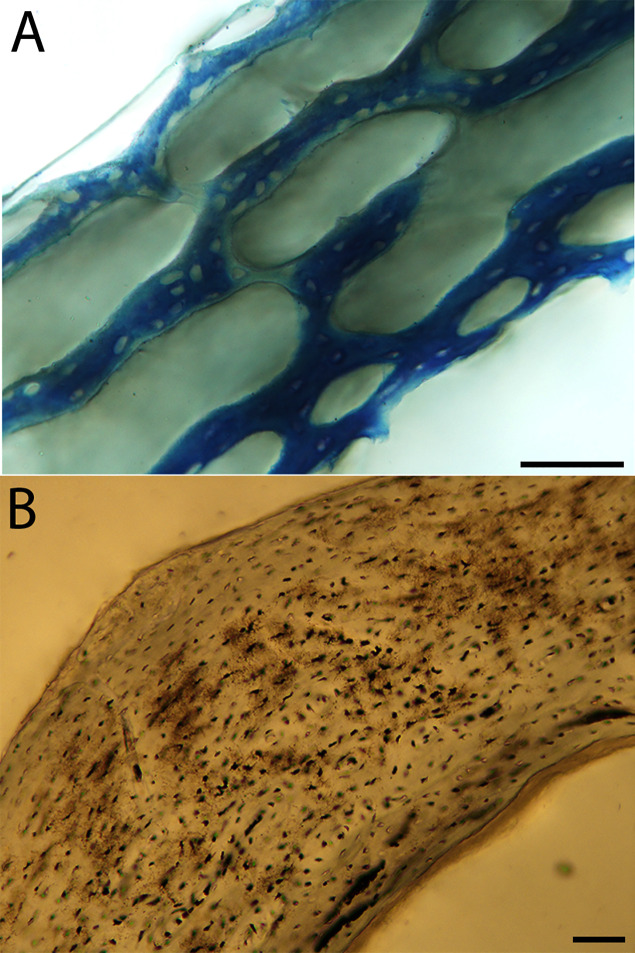
Close-up photographs of microanatomical details in the humeri of semi-altricial 2 birds, in this case a single taxon: the mourning dove (*Zenaida macroura*). For this taxon, neonates do not have ossified long bones. Therefore, this figure includes an image from a pin-feathered chick, the earliest stage at which there was actual bone to section. (A) Mourning dove pin-feathered chick (MVZ190778); (B) mourning dove adult (MVZ190775). In both images the periosteal surface is oriented to the upper left and the endosteal to the lower right. Scale bar = 100 µm.

**Figure 10:**
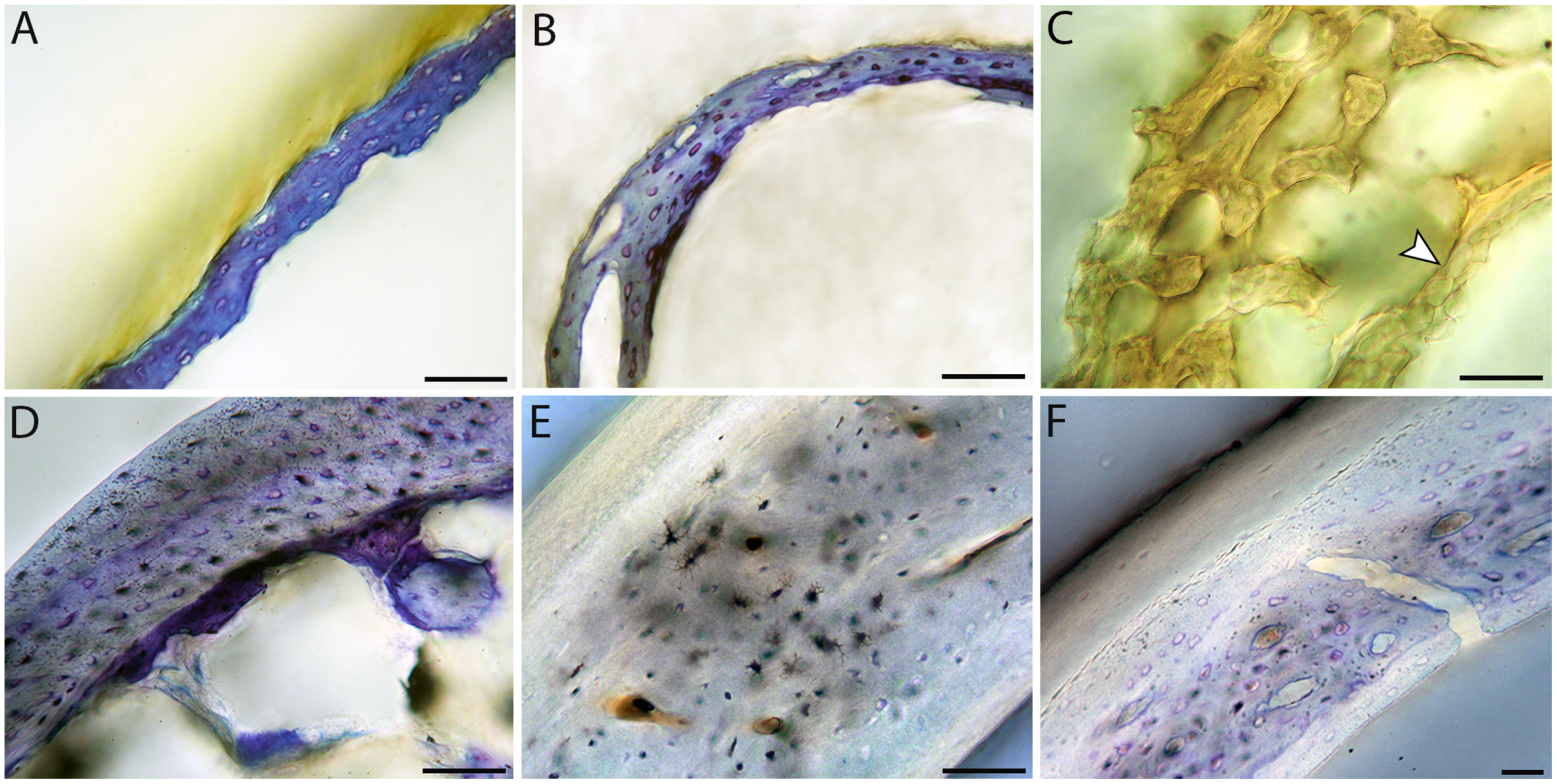
Close-up photographs of microanatomical details in the humeri of altricial birds. Sections from youngest individuals with ossified diaphyses shown in the top row, and sections from adults in the bottom row. (A) Anna’s hummingbird pin-feathered chick (MVZ190799); (B) green-cheeked conure neonate (MVZ190895); (C) Western scrub jay neonate (MVZ190927), arrow indicates endosteal bone that separated from the periosteal bone at a Kastschenko line; (D) Anna’s hummingbird adult (MVZ190807); (E) green-cheeked conure adult (MVZ190917); (F) house finch adult (MVZ190993). In all images the periosteal surface is oriented to the upper left and the endosteal to the lower right. Scale bar = 100 µm.

Humeri of neonates are almost uniformly circular in cross-section (Fig. 5); the two exceptions are the turkey, which has a rounded-triangle cross-sectional shape, and the quail, in which it is elliptical with a flat lateral margin. Vascular density is very high in all individuals, with many canals perforating a disorganized cortex of woven bone (Figs. 6–10). However, the thickness of the developing cortical trabeculae of the bone and the size of the channels (*i.e*., porosity) differ among taxa. Precocial chicks have more ‘mature’ humeri with relatively lower vascular porosity and thicker struts of woven bone (Figs. 6A & 6B). This is particularly noticeable in the quail and turkey, which have humeral bone of the most mature appearance out of all neonates examined. Apart from porosity, neonate humeri are all remarkably similar, consisting of woven bone not only with collagen fibers arranged in a disorganized way, but also with vascular channels displaying much irregularity in size, shape, and orientation. Most humeri can be characterized no more specifically than as having many longitudinal and circumferential channels of greatly variable shapes. Cortical thickness is often asymmetrical in the neonate humerus, to greater or lesser degrees, always showing variation in porosity in correlation with cortical width; narrow cortical areas have denser bone, while vascular channels are larger and more numerous in the thicker regions. This variation did not occur consistently in any particular directional region.

The thickness around the cortex of neonate femora was less variable than in the humerus, displaying patterns of variation more similar to that observed in adults (Fig. 5); qualitative features of neonate femora are summarized in Table 7. The femur therefore appears less variable and subject to change through ontogeny in terms of these macroscopic characteristics, possibly a reflection of the microanatomical evidence that this element is relatively further along in development than the humerus (as observed in most taxa in this dataset—see below for detailed description). In terms of ACT, the Western gull has the thickest cortex (405.71 µm) and the conure has the thinnest (113.68 µm), though the quail is nearly as thin (117.99 µm). The relatively thickest cortex was observed in the scrub jay (29%), and the thinnest in the wild turkey (9%), though the California quail has a nearly equivalent cortex at 11% total cross-sectional diameter (Table 5).

**Table 7:** Qualitative comparisons among neonate femora.

Taxon	Cross-sectional shape	Bone composition & porosity	Primary porosity orientation	Other notable features
California Quail^1^ (*Callipepla californica*)	circular	moderate porosity	longitudinal	uniform thickness w/evenly-distributed vascular canals
Wild Turkey^1^ (*Meleagris gallopavo)*	circular	moderate-low porosity, smaller canals than in humerus	longitudinal	some incipient primary osteons already present; slightly uneven cortical thickness w/lower porosity in thin regions
Western Gull^2^ (*Larus occidentalis*)	circular	high porosity, but woven trabeculae thick and well-developed	longitudinal/irregular	some variation in cortical thickness, canals larger in thicker regions
American Kestrel^3^ (*Falco sparverius*)	circular	high porosity; woven trabeculae very thin	longitudinal/irregular	highly asymmetrical cortical thickness with larger canals in thicker regions
Green-cheeked conure^4^ (*Pyrrhura molinae*)	circular	high porosity, but trabeculae thick and well-developed	all orientations	cortex not of uniform thickness; canals larger in thicker regions
Western Scrub-Jay^4^ (*Aphelocoma californica*)	circular	high porosity; very thin trabeculae	all orientations	solid endosteal circlet of bone present; highly asymmetrical cortical thickness
House Finch^4^ (*Haemorhous mexicanus*)	circular	high porosity; very thin trabeculae	longitudinal/irregular	solid circlet of endosteal bone present

**Note:**

Note that vascular density was high almost ubiquitously among these individuals, therefore porosity is compared among neonates. Results are shown for taxa for which neonate specimens were available, and in which the femur was ossified and viable for this study (the femora of the mourning dove and Anna’s hummingbird were not included for the latter reason). Developmental mode is indicated by color number superscript: orange^1^, precocial; yellow^2^, semi-precocial; green^3^, semi-altricial 1; purple^4^, altricial.

**Figure 11:**
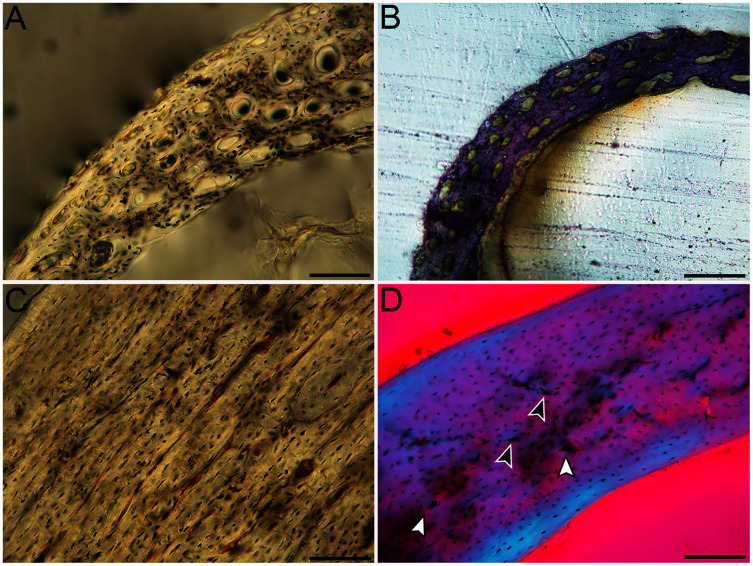
Close-up photographs of microanatomical details in the femora of precocial birds, sections from neonates shown in the top row and from adults in the bottom row. (A) Wild turkey neonate (MVZ190764); (B) California quail neonate (MVZ190751); (C) wild turkey adult (MVZ190763); (D) California quail adult (MVZ190762), showing a region of woven bone with incipient primary osteons (white arrows) and simple vascular canals (black arrows). In all images the periosteal surface is oriented to the upper left and the endosteal to the lower right. Scale bar = 100 µm.

**Figure 12:**
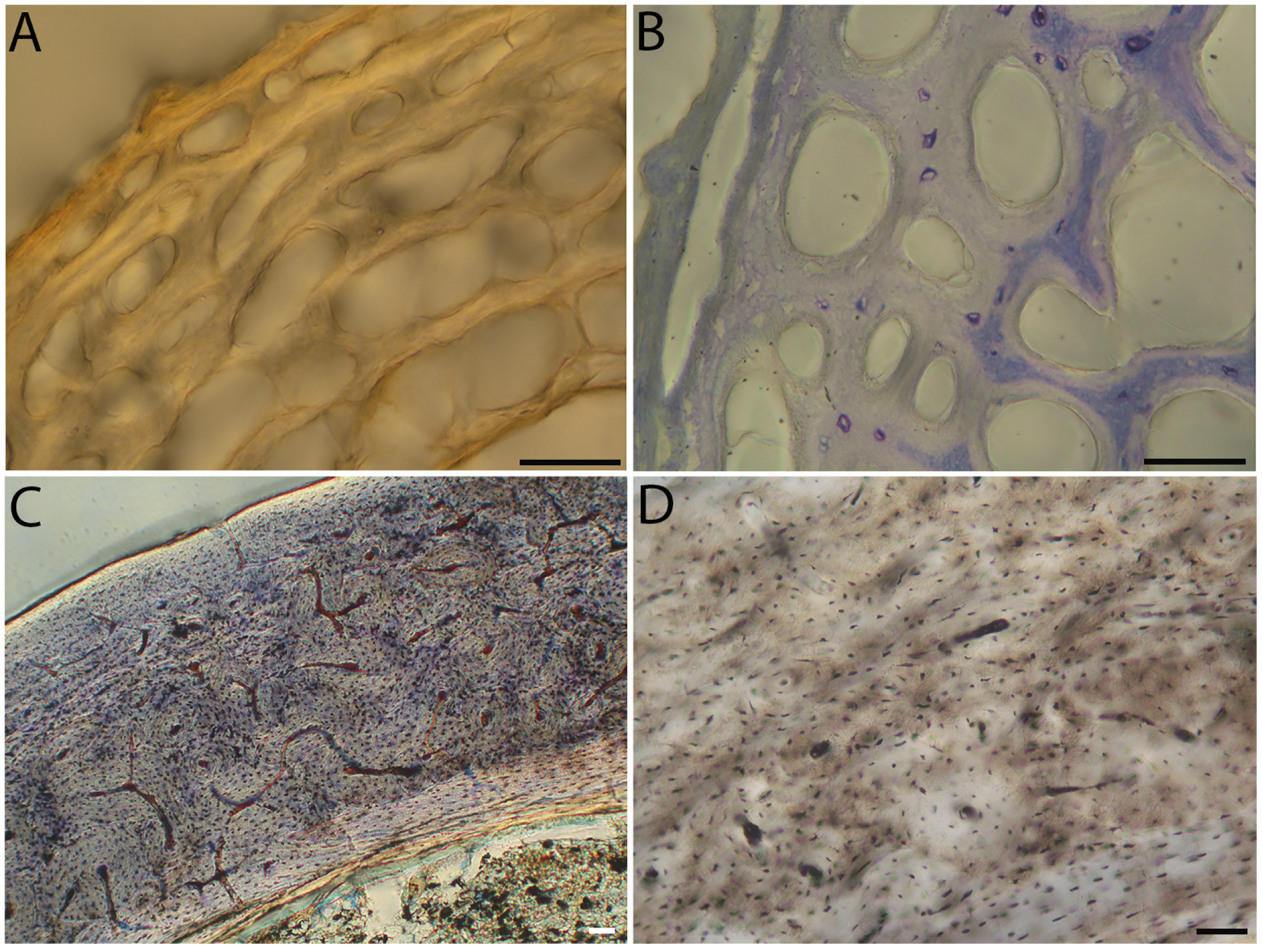
Close-up photographs of microanatomical details in the femora of semi-precocial birds, sections from neonates shown in the top row and from adults in the bottom row. (A) Western gull neonate (JAA64); (B) Western gull neonate (MVZ190822); (C) Western gull adult (MVZ190829); (D) Western gull adult (MVZ190831). In all images the periosteal surface is oriented to the upper left and the endosteal to the lower right. Scale bar = 100 µm.

**Figure 13:**
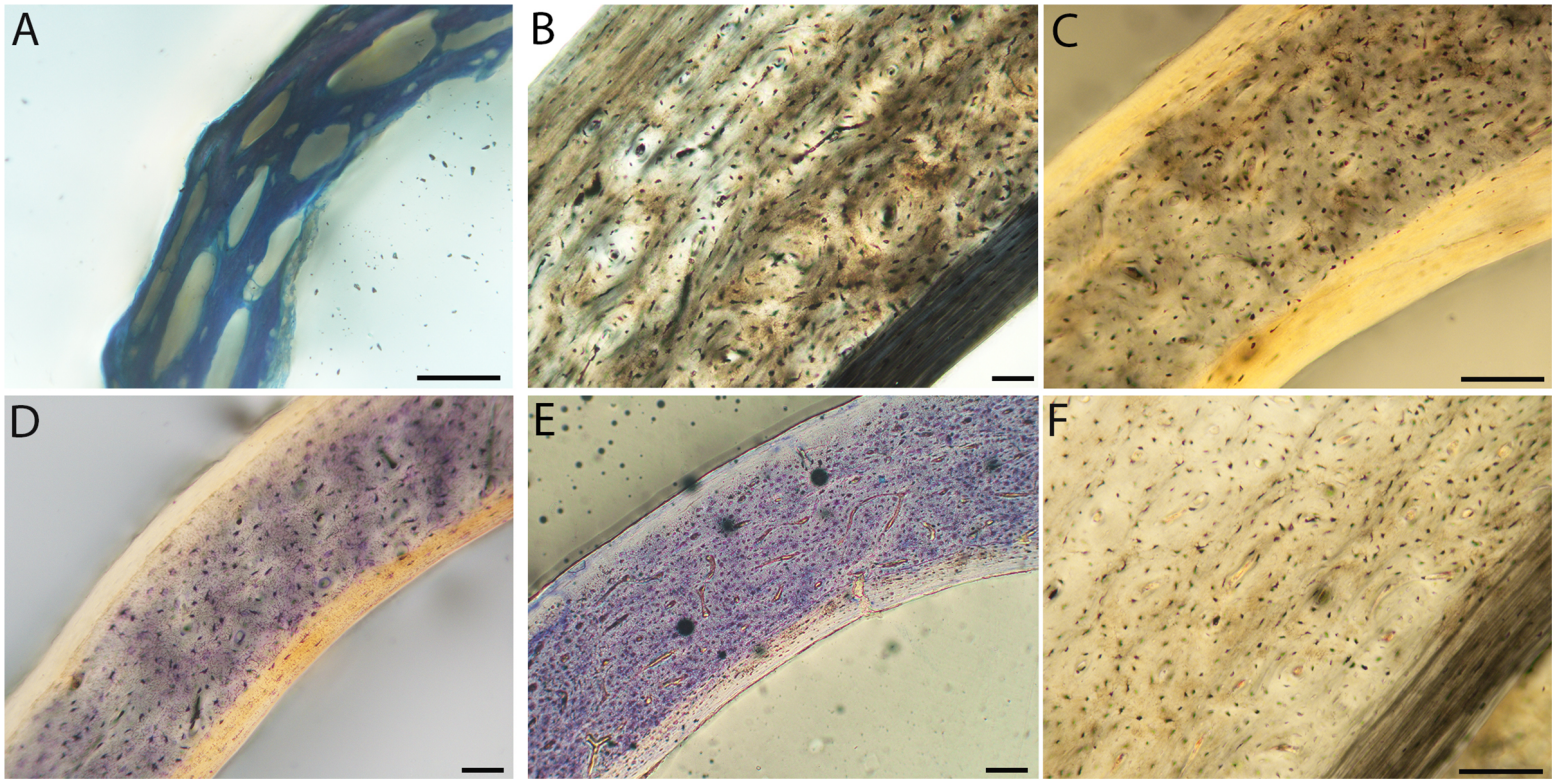
Close-up photographs of microanatomical details in the femora of semi-altricial 1 birds. For this developmental mode, a neonate sample was only acquired for one taxon (the American kestrel), therefore this figure shows mainly adult histology. For descriptions and images of immature stages between neonate and adult, please see Supplemental Material. On the far left of the figure are samples of a neonate (MVZ190890); (A) and adult (MVZ190885); (D) American kestrel. Other panels show adult microanatomy: (B) red-tailed hawk (MVZ190855); (C) white-tailed kite (MVZ190861); (E) barn owl (MVZ190877); (F) great-horned owl (MVZ190883). In all images the periosteal surface is oriented to the upper left and the endosteal to the lower right. Scale bar = 100 µm.

**Figure 14:**
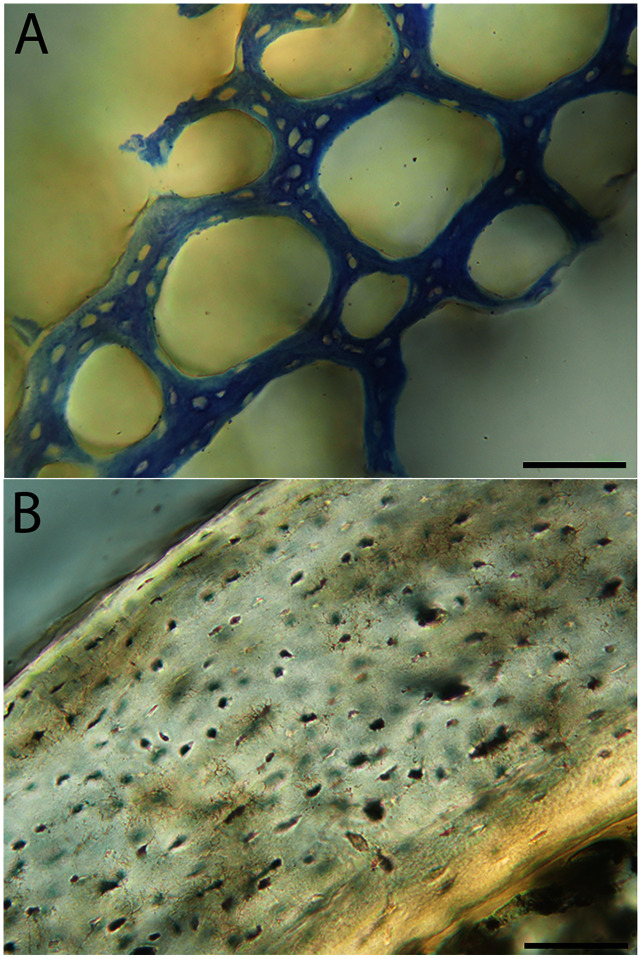
Close-up photographs of microanatomical details in the femora of semi-altricial 2 birds, in this case a single taxon: the mourning dove (*Zenaida macroura*). For this taxon, neonates do not have ossified long bones. Therefore, this figure includes an image from a pin-feathered chick, the earliest stage at which there was actual bone to section. (A) Mourning dove pin-feathered chick (MVZ190778); (B) mourning dove adult (MVZ190775). In both images the periosteal surface is oriented to the upper left and the endosteal to the lower right. Scale bar = 100 µm.

**Figure 15:**
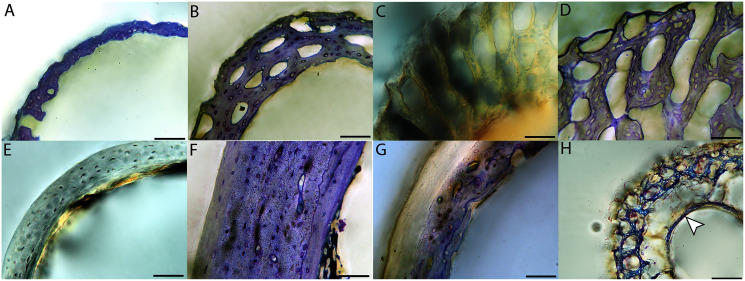
Close-up photographs of microanatomical details in the femora of altricial birds. (A) Anna’s hummingbird pin-feathered chick (MVZ190799); (B) green-cheeked conure neonate (MVZ190895); (C) house finch neonate (MVZ190969); (D) Western scrub jay neonate (MVZ190927); (E) Anna’s hummingbird adult (MVZ190807); (F) green-cheeked conure adult (MVZ190917); (G) house finch adult (MVZ190993). (H) house finch pin-feathered chick showing inner circle of “floating” bone interpreted as evidence of a Kastschenko line. In all images the periosteal surface is oriented to the upper left and the endosteal to the lower right. Scale bar = 100 µm.

Neonate femora are all circular in cross-section (Fig. 5). The bone of the neonate femur shares some features with the humerus. It is composed of a woven bone matrix of disorganized fibers perforated by numerous vascular channels that are highly irregular in shape and orientation. The relative size of these channels, and thus the porosity of the bone, is one of the characteristics that differs most among taxa. The California quail and wild turkey have the lowest porosity, *i.e*., canals of the smallest relative size. Vascular channels in the femora of these two taxa are also most organized of all the neonates, and can be unambiguously classified as longitudinal. In the gull, porosity is high, but the trabeculae of woven bone are quite thick. In the conure, scrub-jay, house finch, and kestrel, porosity is highest and the trabeculae are thinnest. In all of these taxa, it is difficult to classify predominant vascular orientation because there is such variation in the shape of channels and (Figs. 11–15; Table 7). Overall, these differences appear to reflect differences in maturity of the bone at hatching. Precocial quail and turkeys have the most mature bone, followed by semi-precocial gulls, while the most immature bone is seen in the altricial and semi-altricial chicks. Also as in the humerus, neonate femora display a hugely varying asymmetry in cortical thickness, and corresponding differences in density/porosity.

Chicks in the pin-feathered through fledgling stages frequently are distinguished by the presence of a thick, prominent endosteum that was seen only as a very thin layer in adults and neonates (when visible at all). Though we did not prepare our sections for study of soft tissues, when inadvertently preserved in a section, the soft tissue endosteum was consistently thick and prominent in the pin-feathered through fledgling stages, and only observed as a thin layer in neonates and adults. Indeed, a thickened endosteum was of one of the only distinguishing features between sub-adult and adult birds. Of particular importance to growing chicks, the endosteal tissue functions to house osteoprogenitor cells and providing an important environment for production of hematopoetic stem cells and multipotent cells (Hall, 2005; Eroschenko, 2008; Cordeiro-Spinetti, Taichman & Balduino, 2015), all of which would be particularly important in growing chicks.

In the two passerines included in this study, a unique feature was observed in early growth stages: a circlet of bone in the endosteal cavity almost completely detached from the rest of the developing cortex (Figs. 5H, 10C, 15H). In Western scrub jays, this was present in the humerus and femur of the neonate. In the house finch, this was observed in both the humerus and femur of the pin-feathered stage. We interpret this intracortical gap as evidence of a Kastschenko line (Francillon, 1980), a thin layer of osteoid tissue that occurs between growing endosteal and periosteal bone, degraded and no longer present in these thin sections but leaving behind a space where it used to be.

Chondroid bone has been described as abundantly present in the bones of developing Rouen ducks by Prondvai et al. (2020). Similarly, chondroid bone was very common in many growth stages of most taxa observed here (*e.g*., Figs. 6A, 14A, 15D). It is present in large areas of bone in younger chicks, and disappears approximately by the fledgling stage in most taxa, though was observed up to the subadult stage in Anna’s hummingbird. This discovery of wide-spread chondroid bone across avian taxa supports the results and predictions of Prondvai et al. (2020), and will be described and addressed in greater detail in a future publication.

### Adult histology

**Figure 16:**
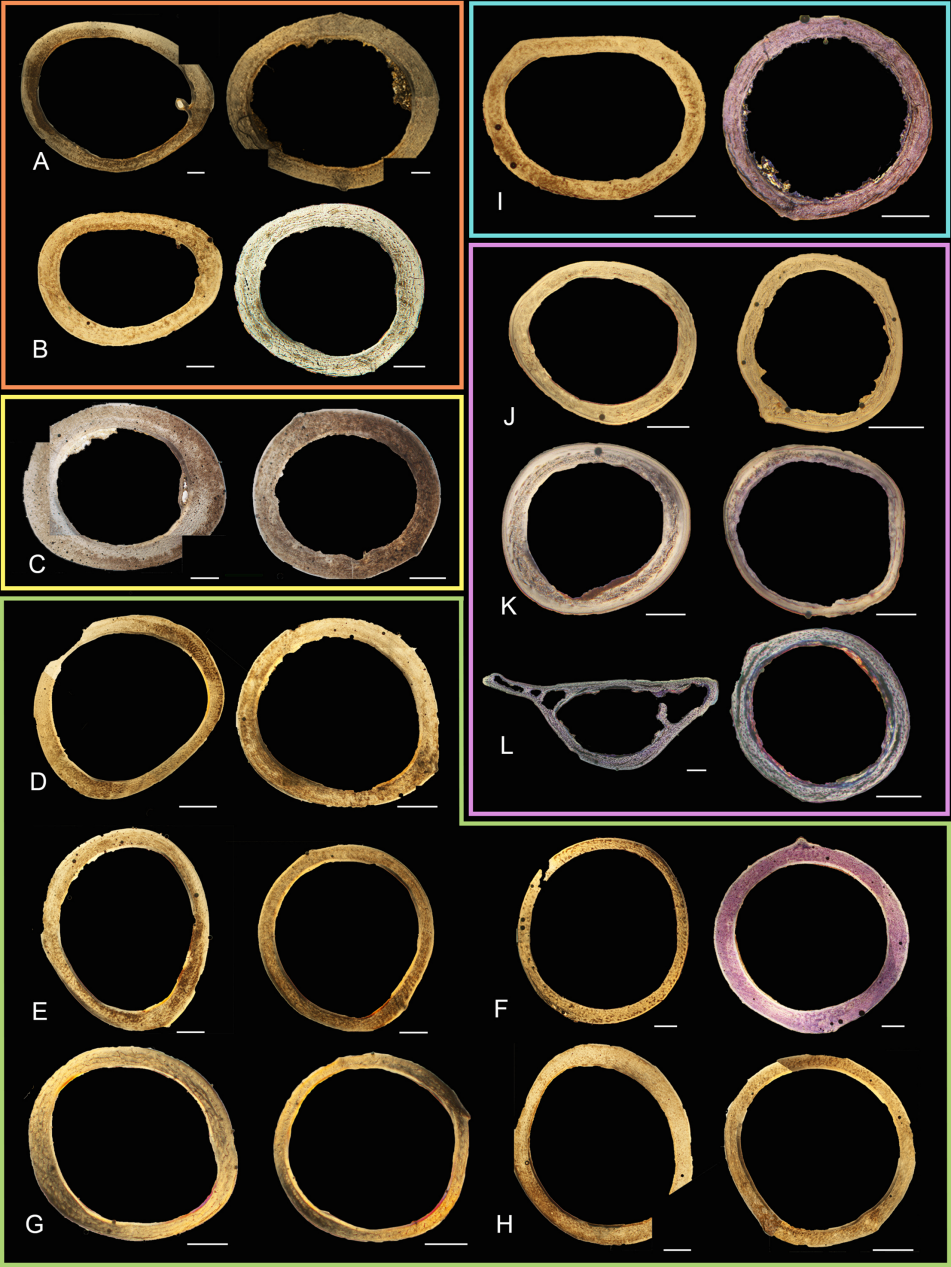
Diaphyseal cross-sections of all adults included in this study, showing general cross-sectional geometry and variation in cortical thickness. For each pair, the humeral section is on the left and the femoral on the right. In all images cranial is up and lateral is left. Sections are grouped by developmental mode. Precocial (orange): (A) Wild turkey (*Meleagris gallopavo*; MVZ190763), scale bar = 1,000 µm. (B) California quail (*Callipepla californica*; MVZ190762), scale bar = 500 µm. Semi-precocial (yellow): (C), Western gull (*Larus occidentalis*; MVZ190831), scale bar = 1,000 µm. Semi-altricial 1 (green): (D) great-horned owl (*Bubo virginianus*; MVZ190883), scale bar = 1,000 µm; (E) white-tailed kite (*Elanus leucurus*; MVZ190861), scale bar = 500 µm; (F) barn owl (*Tyto alba*; MVZ190877), scale bar = 500 µm; (G) American kestrel (*Falco sparverius*; MVZ190892), scale bar = 500 µm; (H) red-tailed hawk (*Buteo jamaicensis*; MVZ190855), scale bar = 1,000 µm. Semi-altricial 2 (blue): (I) mourning dove (*Zenaida macroura*; MVZ190775), scale bar = 500 µm. Altricial (purple): (J) green-cheeked conure(*Pyrrhura molinae*, MVZ190917), scale bar = 500 µm; (K) house finch (*Haemorhous mexicana*; MVZ190993), scale bar = 250 µm; L, Anna’s hummingbird (*Calypte anna*; MVZ190807), scale bar = 100 µm. For cross-sections of all ages, see figures in Supplemental Material.

Across somatically mature adults, the humerus varied in cross-sectional shape from roughly triangular (turkey) to ovate (quail, dove, hummingbird) to circular (all other taxa); many humeri of adult individuals are moderately flattened along one margin (Fig. 16); qualitative features of adult humeri, including cross-sectional shape, are summarized in Table 8. Some of this variation may reflect which portion of the shaft the tissue sample is from (the midshaft region was sampled generally, but some sections are marginally more proximal or distal), though it is notable that the femur, sampled in the same way, does not show such variation. This developmental stage displayed the least variance in cortical thickness throughout the circumference of the bone, with all cortices of uniform or nearly uniform breadth. In many taxa ACT reaches a maximum at adult body size, but RCT of the humerus was generally thinnest in adults (though sometimes very early growth stages were equally thin, see Table S1). Western gulls have the highest RCT (0.34). In all other birds, cortical width ranged from 18–28% of cortical diameter, a consistency remarkable considering the phylogenetic breadth and locomotor diversity of the sample size.

**Table 8:** Qualitative comparisons among adult humeri.

Taxon	Cross-sectional shape	Presence of ICL and OCL	Composition of middle cortical layer	Relative vascular density	Primary vascular orientation
California Quail^1^ (*Callipepla californica*)	circular to ovate	yes; OCL thicker (~1/3 of cortex) but ICL more distinct	woven/incipient fibrolamellar	moderate	longitudinal
Wild Turkey^1^ (*Meleagris gallopavo)*	rounded triangle	yes; ICL thicker and more distinct	fibrolamellar	high	circumferential/ plexiform
Western Gull^2^ (*Larus occidentalis*)	circular	yes; ICL thicker and more distinct	fibrolamellar	high	reticular
American Kestrel^3^ (*Falco sparverius*)	circular	yes; equal thickness, ICL more distinct	woven w/simple canals and regional fibrolamellar	moderate	longitudinal/ reticular
Red-tailed Hawk^3^ (*Buteo jamaicensis*)	circular	yes; ICL thicker and more distinct	fibrolamellar	high	longitudinal
White-tailed Kite^3^ (*Elanus leucurus*)	circular	yes; OCL thicker, ICL more distinct	fibrolamellar bone with regional woven bone w/simple canals	moderate	longitudinal
Barn Owl^3^ (*Tyto alba*)	circular	yes; equal thickness, ICL more distinct	fibrolamellar	high	reticular/ circumferential
Great-horned Owl^3^ (*Bubo virginianus*)	circular	yes; ICL thicker and more distinct	fibrolamellar	high	longitudinal
Mourning Dove^4^ (*Zenaida macroura*)	ovate	yes; ICL incipient, OCL 1/3-1/2 cortex	weakly woven with primary osteons (middle layer is of equal thickness to OCL in some areas)	moderate	longitudinal
Anna’s Hummingbird^5^ (*Calypte anna*)	ovate (main medullary cavity)	yes; OCL dominates cortex	none	avascular	—
Green-cheeked conure^5^ (*Pyrrhura molinae*)	circular	yes (but ICL thin/irregular); OCL thicker	woven bone with primary vascular canals; patches of fibrolamellar	moderate-low	longitudinal
House Finch^5^ (*Haemorhous mexicanus*)	circular	yes; OCL is thicker, ~1/3-1/2 of cortex	woven bone with primary vascular canals; patches of fibrolamellar	moderate-low	longitudinal

**Note:**

Vascular density in adults was ubiquitously lower than in younger growth stages, thus *relative* vascular density among adults only is reported here. “Middle cortical layer” refers to the portion of the cortex located between the OCL and the ICL. Descriptions of the ICL as “more distinct” refer to instances in which the ICL is more advanced in development than the OCL, and to the very high level of organization of tissue in this layer (which appears strongly lamellated and has a distinct, high birefringence). Developmental mode is indicated by color number superscript: orange^1^, precocial; yellow^2^, semi-precocial; green^3^, semi-altricial 1; blue^4^, semi-altricial 2; purple^5^, altricial.

High levels of variation were also observed in the density of vascular canals relative to anatomical location within the cortex (particularly traversing from inner to outer cortex), orientation of these channels, and relative thinness of the ICL and OCL. The size of vascular channels was small in all individuals, but qualitative data suggest porosity may be correlated with body size: canals occurred in very low densities in hummingbirds, and moderate numbers in doves, conures, finches, and kestrels; in all other taxa, vascular density was high. All humeri showed almost the full range of vascular canal orientations, but in the majority a reticular or longitudinal pattern was dominant; only the wild turkey and barn owl displayed a laminar arrangement. At the microstructural level, adult bone was very similar across taxa in having small, elliptical or compressed osteocyte lacunae.

Avian adult femora display even less variation than the humeri; qualitative characteristics of these elements are summarized in Table 9. Almost all are perfectly circular in cross-section (Fig. 16), with the exception of one of the adult quail. In nearly all taxa, the ACT of the femur is less than that of the humerus (Table 4). The thickest femoral cortices are found in the turkey (1240.14 µm) and the great-horned owl (736.66 µm). The thinnest is in the hummingbird (70.55 µm). In terms of RCT, the barn owl has the greatest cortical thickness in the femur (0.30). The RCT of all other taxa falls in a range from 0.18–28; notably, five of these taxa (representing a wide range of body sizes and flight styles) all have an average adult femoral RCT of 0.18 (Table 5).

**Table 9:** Qualitative comparisons among adult femora.

Taxon	Cross-sectional shape	ICL and OCL present	Composition of Middle Cortical Layer	Relative vascular density	Primary vascular orientation
California Quail^1^ (*Callipepla californica*)	circular to ovate	yes; OCL thicker (~1/4-1/3 cortex) but ICL more distinct	woven with simple canals/regional fibrolamellar	moderate	longitudinal
Wild Turkey^1^ (*Meleagris gallopavo)*	circular to ovate	yes; ICL thicker and more distinct	fibrolamellar	high	circumferential/plexiform
Western Gull^2^ (*Larus occidentalis*)	circular	yes; equal thickness, ICL more distinct	fibrolamellar	high	reticular
American Kestrel^3^ (*Falco sparverius*)	circular	yes; equal thickness, ICL more distinct	parallel-fibered with regional fibrolamellar	moderate	longitudinal
Red-tailed Hawk^3^ (*Buteo jamaicensis*)	circular	yes; ICL thicker and more distinct	fibrolamellar	high	reticular-longitudinal
White-tailed Kite^3^ (*Elanus leucurus*)	circular	yes; equal, ICL more distinct	fibrolamellar bone with regional woven bone w/simple canals	moderate	longitudinal
Barn Owl^3^ (*Tyto alba*)	circular	yes; equal thickness, ICL more distinct	fibrolamellar	high	reticular-longitudinal
Great-horned Owl^3^ (*Bubo virginianus*)	circular	yes; ICL thicker and more distinct	fibrolamellar	high	longitudinal
Mourning Dove^4^ (*Zenaida macroura*)	circular	yes; OCL thicker (comprises almost entirety of cortex)	small patches of weakly woven with sparse simple canals	low	longitudinal
Anna’s Hummingbird^5^ (*Calypte anna*)	circular	yes; ICL incipient(?), OCL dominates cortex	none	avascular	—
Green-cheeked conure^5^ (*Pyrrhura molinae*)	circular	yes; OCL thicker, about 1/3 of cortex	woven bone with simple vascular canals; patches of fibrolamellar bone	low	longitudinal
House Finch^5^ (*Haemorhous mexicanus*)	circular	ICL absent; OCL ~1/2 of cortex	parallel-fibered with regional woven bone	low	longitudinal

**Note:**

Vascular density in adults was ubiquitously lower than in younger growth stages, thus *relative* density among adults only is reported here. “Middle cortical layer” refers to the portion of the cortex located between the OCL and the ICL. Descriptions of the ICL as “more distinct” refer to the very high level of organization of tissue in this layer, which appears strongly lamellated and has a high birefringence. Developmental mode is indicated by color number superscript: orange^1^, precocial; yellow^2^, semi-precocial; green^3^, semi-altricial 1; blue^4^, semi-altricial 2; purple^5^, altricial.

Vascular channels were very small in size but present in large numbers in most taxa, as in the humerus. A low vascular density was only observed in the house finch, and the hummingbird femur is avascular. Notably, these are the two smallest adults included in this study. These observations are consistent with results reported by Cubo et al. (2014), who discovered a significant correlation between vascular density and body size in a number of adult birds and lepidosaurs. This is a reminder that, in mature bone, metabolic requirements and method of oxygen and nutrient receipt are related to size of an element, which should be considered when making paleohistological interpretations. A predominantly laminar orientation of vascular channels was observed only in the wild turkey and one barn owl specimen. In all other taxa, canals were arranged longitudinally, in an anastomosing network, or some variation between these two.

Overall, there is a fair amount of variance in the humeral and femoral histology of adults, with cortices ranging from predominantly fibrolamellar to avascular parallel-fibered bone (Figs. 6, 11C & 11D; Figs. 7,12C, 12D; Figs. 8, 13B–13F; Figs. 9 & 14B; Figs. 10D–10F; Figs. 15E–15G) . The wild turkey, Western gull, red-tailed hawk, barn owl, and great-horned owl have humeral cortices composed of a thick layer of fibrolamellar bone between a clear OCL and ICL. In California quail, American kestrels, white-tailed kites, and mourning doves, the cortex is mainly composed of a woven or parallel-fibered matrix with regional fibrolamellar bone or incipient primary osteons. In the house finch and green-cheeked conure the humerus is composed of a thin, middle layer of woven matrix with simple vascular canals, between a strongly parallel-fibered OCL and ICL. Finally, in Anna’s hummingbird the cortex of the humerus is nearly avascular and composed entirely of parallel-fibered bone.

All individuals classified here as “adults” did indeed have an OCL in the humerus and femur. However, in some taxa an OCL appeared in some individuals assigned to other growth stages (summarized in Table 1). In mourning doves, an incipient OCL is present at the subadult stage in both the humerus and femur (Figs. S18F & S18G, S20G). In green-cheeked conures, an incipient OCL is visible by the fledgling stage in the humerus, and the pre-fledging stage in the femur (Fig. S32D). The femur of the fledgling has a thick, prominent OCL, and has achieved a cross-sectional size nearly equivalent to that of the adult. In Western scrub jays, the humerus of the subadult individual shows signs of early OCL formation (Fig. S34G), and the femur of the fledgling and subadult stages both exhibit incipient OCLs (Figs. S36F & S36G). In house finches, the OCL first starts to develop in the fledgling stage in the humerus (Fig. S38D), and the pre-fledgling stage in the femur (Figs. S40C & S40D). By the fledgling stage, the OCL is thick and clearly developed, and cross-sectional size of this element is within the range of the adult (in fact, slightly larger in the two individuals compared here). In the great-horned owl, OCL formation begins in the fledgling chick in the humerus and femur (though it is in very early stages of development; Figs. S42D and S44C). In barn owls, an incipient OCL is also present in the femur as early as the fledgling stage (Fig. S48F), and by the subadult stage appears well-developed and has achieved the same thickness as that of the adult stage. The difference, instead, is lack of an ICL in the subadult individual. The humerus of the barn owl also has an incipient OCL by the subadult stage (Fig. S46G).

Anna’s hummingbird is the most outstanding exception to an OCL correlating with “adulthood” based on plumage. An OCL was present in the humerus as early as the fledgling growth stage (Fig. S14B), and persists through the subadult stage. In the adult, the OCL is no longer visible as a distinct layer (Fig. 10D). Rather, it appears to have widened so much that, in combination with endosteal resorption, it now comprises nearly all the bone of the cortex. Furthermore, the size of the fledgling humerus is comparable to that of the adult.

An ICL was also present in the femur and humerus of nearly all adult birds in this dataset. This feature often appeared as a very distinctive layer of highly organized tissue, parallel-fibered bone bordering on lamellar in some instances. It appeared to be only in early stages of formation in the adult hummingbird (Figs. 10D and 15E), and was entirely absent in femur of the adult house finch, which instead had a scalloped endosteal margin indicative of active resorption (Fig. 15G). Such variation may be linked to differing stages of the life-cycle in which endosteal resorption and deposition of bone are activated. Additionally, of the two adult barn owls sectioned in this study, one lacked an ICL in the humerus, indicating that tissue growth and/or resorption along the endosteal margin was still active, and suggesting this individual had either not yet achieved the same level of cortical maturity as the other, or had resorbed the endosteal surface for mineral mobilization (however, both individuals have an ICL and OCL in the femora).

Relatively little remodeling in humeri and femora has been described in previous studies of the limb bones of adult birds (Enlow & Brown, 1957; Currey, 2003; Simons & O’connor, 2012), with the exception of extremely dense Haversian systems present in penguin bones (related to the aquatic lifestyle of these animals (Meister, 1962) and paleognaths (Bourdon et al., 2009; de Ricqlès et al., 2016; Chinsamy et al., 2020a). With respect to the mid-shaft of the humerus and femur, the results of this investigation concur with these studies, and only rare, small, localized areas of osteonal remodeling were observed (*e.g*., in the wild turkey humerus).

## Discussion

Several osteohistological attributes characterize Aves as a whole, both in terms of general ontogenetic trends and similarities among specific growth stages. In all neonates, the humerus and femur are composed of a disorganized matrix of woven bone with many large, irregular vascular channels, and a high osteocyte density (with round, large osteocyte lacunae) (Figs. 6–15). In many hatchling chicks, it was difficult to identify a primary orientation of vascular canals because their arrangement appeared so haphazard. This is unsurprising, and corroborates with both previous reports on bone histology of avian chicks (Castanet et al., 2000; de Margerie, Cubo & Castanet, 2002; de Margerie et al., 2004), and data available on high growth rates in birds (Starck, 1989; Starck, 1993; Starck, 1994; Starck & Ricklefs, 1998) as bone with such a lack of organization is known to be very fast-growing. Additionally, nearly all taxa hatch with a femoral cortex that is thicker than that of the humerus (as an absolute measure), but this relationship becomes inverted by the adult stage, when the bone of the humerus is almost invariably thicker than the femur. Absolute cortical thickness generally increases through ontogeny, reaching highest values in adults. Conversely, relative cortical thickness often decreases between the neonate and adult stages. Below we discuss further patterns related to phylogeny, biomechanics, and ontogeny.

### Phylogenetic patterns

In this study, only RCT of the neonate femur has significant phylogenetic signal (*K* = 1.235; *P* = 0.043). Phylogenetic signal has been reported in other histological characteristics of adult birds by Houde & Olson (1986) and Houde (1987), in ostriches and the extinct bird *Hesperornis*. Other examples include signal in the density of Haversion bone (Ponton et al., 2007); relative cortical thickness of sauropsid femora, including a diverse sampling of extant birds (Cubo et al., 2005); and a wide range of features (*e.g*., vascular density and vascular orientation) across paleognathous birds (Legendre et al., 2014). It is perhaps surprising, then that significant phylogenetic signal was not detected in more traits in the present investigation. However, we note that this may be because low sample sizes, such as in this study, predispose these tests to type II error, and others urge the use of large datasets with thorough phylogenetic coverage to rigorously support a hypothesis of phylogenetic signal (Legendre et al., 2014). Future studies incorporating more taxa may yield different results.

Some features observed appear to be clade-specific. Very young chicks of both passerine taxa included in this study (the house finch and scrub-jay) have a unique characteristic in the humerus and femur: a thin, solid ring of bone that lines the endosteal edge of the woven bone comprising the cortex, making the medullary cavity very distinct (Figs. 5H, 10C, 15H). This circlet of bone appears to detach and ‘float’ into the medullary cavity later in development, where it is presumably resorbed as it appears in no later stages of ontogeny. We interpret this as evidence of a Kastschenko line, a thin layer of tissue that is a remnant of the cartilaginous precursor that forms earlier in bone development, located between the growing periosteal and endosteal bone (Kastschenko, 1880; Francillon, 1980; Castanet & Smirina, 1990). In this case, the space observed between the inner circlet of bone and the rest of the cortex is all that remains of the osteoid layer. During the specimen preparation process, periosteal and endosteal layers have separated along this region of thin, delicate tissue, forming a space between the two layers of bone. This is a very common osteohistological feature in frogs and salamanders (*e.g*., Rozenblut & Ogielska, 2005; Song et al., 2017) , but is relatively rare in other groups. To date, the only amniotes in which a Kastschenko line has been identified are elasmosaurid and polycotylid plesiosaurs (O’gorman, Talevi & Fernández, 2016; O’Keefe et al., 2019), early-diverging sauropodomorphs (Cerda, Pol & Chinsamy, 2014), a titanosaur sauropod (González et al., 2020), and theropods (de Ricqlès et al., 2001). This study therefore presents the first evidence of this structure in Avialae. Furthermore, both taxa in which it was identified belong to the clade Passeriformes, suggesting that the Kastschenko line is indicative of a growth strategy specific to members of this clade, however, sampling of additional passerine and non-passerine birds will be necessary to confirm the biological reality of this pattern.

### Histological variation

RCT is remarkably similar across adults of all taxa. This is particularly the case for the humerus, likely because the bones of flighted taxa thin out to a relatively restricted range of RCT values to simultaneously support the strains of active flight and reduce body weight. Both RCT and ACT are some of the most highly variable traits in growing chicks. Across taxa, birds of the same growth stage often exhibited very different cortical thicknesses, and within a given element great asymmetry in width of the cortical bone was also frequently observed, indicating that growth stage based on external, gross anatomical features (such as plumage) are unsurprisingly not “equivalent” between taxa in terms of bone development. Alternatively, it may reflect varying mechanical forces on the bone through growth, though the lack of pattern for a given element among individuals of the same growth stage and taxon suggests that this characteristic is non-adaptive, or that a greater sample size is needed for the adaptive pattern to become apparent. These differences among birds of the same growth stage later become highly reduced as elements converge to similar thicknesses among adults of the same taxon. This was observed across clades and implies that selective forces affecting cortical thickness may be less strong at earlier stages of development. It also suggests that environmental factors, differences in parental care, and quality/amount of food available from clutch to clutch may have a strong effect on the thickness of bone in chicks.

Overall, regional differences consistently related to anatomical direction within a section (that is, differences among cranial, caudal, medial, and lateral quadrants) were minimal across growth stages. Other studies have also reported a lack of regional histovariability. For instance, Simons & O’connor (2012) examined laminarity in adult birds of a range of flight styles, and found no significant variation among the four cortical quadrants. Here, the only major regional variation observed within a given bone cross-section was in the seemingly arbitrary thickness of cortical bone discussed above. Most of this variation was seen in the humerus, and primarily at younger developmental stages where it was very common.

### Mechanical signal

Almost invariably, a trend of decreasing RCT is observed through post-natal ontogeny, with adults having, on average, lower RCT than chicks (Table 5). However, it must be noted that this trend is not always a constant, unidirectional decrease, and that there are some exceptions to this pattern. In the turkey and quail, adult RCT of the femur is greater than in the neonate. Both taxa are precocial, and both are members of Galliformes, an early-diverging clade, so this characteristic may be related to life history or inherited from early common ancestors. Alternatively, this could reflect functional demands; both birds rely primarily on the pelvic limb for locomoting and are not strong fliers.

When considering more complete growth series (that is, not only the end points), other trends in cortical growth become apparent. In many taxa, there is an increase in RCT from the neonate to pin-feathered stage followed by a decrease, and an increase from pre-fledgling to fledgling stage followed by a decrease. Similarly, some taxa also undergo an increase in ACT at the pin-feathered or fledgling stage, followed by a decrease in later ontogenetic stages. However, cortical thickness within and between individual growth stages exhibited such great variability that it is impossible to comment on the biological reality of such trends without a greater sample size. Nonetheless, this does suggest a general trend of highly dynamic osteoblast and osteoclast activity, with rates of endosteal resorption and periosteal deposition fluctuating through ontogeny. Patterns of periosteal growth in birds are therefore complex, and the relationship between rates of periosteal deposition and endosteal resorption is ever-shifting.

Some trends in cortical thickness are clearly explicable. In adults, ACT is typically at a peak because individuals have reached maximum body size and thus have larger bones than earlier stages. Also, adults tend to have measures of lowest RCT because thin-walled bones are an adaptation to flight. However, proximate functional causes of other ontogenetic changes in the cortical thickness of the humerus and femur are more ambiguous.

A previous study suggests (Carrier & Leon, 1990) that the structural weaknesses of the immature bone of young birds may be compensated for by a greater cortical thickness. Time of fledging, when a bird begins to fly, places especially high demand on the wing bones, potentially explaining the secondary increase in relative cortical thickness often observed in fledgling chicks. Carrier & Auriemma (1992) note that time of fledging is limited by relative length of certain limb elements (birds with proportionally longer humeri and ulnae take longer to reach the fledgling stage). These authors infer that longitudinal growth may be a developmental constraint imposing a minimum duration to the fledgling stage. This critical stage of avian ontogeny is strongly affected by the dynamics of bone growth, and diametric thickening of the bone may be a way to offset the limitations of longitudinal growth and/or weaknesses of the immature tissue.

Other histological features have also been suggested as adaptations to locomotion, and to resisting the various strains that it imposes. de Margerie, Cubo & Castanet (2002) and de Margerie (2006) proposed that circular cross-sectional shape, a thin cortical wall, collagen fibers arranged spirally around the bone shaft at a 45° angle, and bone laminarity (*i.e*., many circumferential vascular canals) are all functional adaptations for torsion resistance. They further report that degree of laminarity varies across skeletal elements, and is highest in the humerus, radius, ulna, and femur.

If true, we expect laminar vascularity will be found in the major pectoral elements across volant avian taxa. The range of taxa explored in this study allows a test of this hypothesis, which does not appear to hold true across the avian phylogeny. Laminar vascularity was in fact quite rare in the samples studied here. In a few adult birds, laminar bone did dominate the humerus (the wild turkey, one of the barn owls, and weak lamination in some regions of the red-tailed hawk cortex), but it was absent in the vast majority of specimens. Certainly, a larger sample size and additional sectioning of each element would provide a more rigorous test, but preliminary results indicate that laminar vascularity does not dominate the humerus in a majority, or even many, birds. This corroborates recent findings in homing pigeons (McGuire et al., 2020) and bats (Lee & Simons, 2015). Of particular note, McGuire et al. (2020) observed a higher degree in laminarity in younger individuals, with vascular orientation becoming increasingly longitudinal with maturity. Though additional quantitative studies of data presented here are necessary, current qualitative observations suggest this ontogenetic pattern is likely common across Aves.

Interestingly, the femur of the wild turkey was also predominantly laminar, as were the femora of the older ostrich chicks. These results are in keeping with the idea that this orientation of vascular canals is an adaptation to torsion, because the avian femur, which is obliquely oriented in the cranio-caudal plane, is subject to high torsion, particularly in large terrestrial birds such as the turkey, ostrich, and emu (de Margerie, Cubo & Castanet, 2002; de Margerie et al., 2005; Kuehn et al., 2019).

### Developmental mode & onset of bone growth

At the start of this investigation, we hypothesized that a histological signal of developmental mode would be present in the form of differing cortical thickness in pectoral *versus* pelvic limb elements. Precocial chicks locomote earlier, and with the pelvic limb, and should have an absolutely thicker and more mature femoral cortex than humeral; in contrast, altricial chicks will ultimately locomote primarily with the pectoral limb, and should have an absolutely thicker and more mature humeral cortex relative to that of the femur (or at least of equal maturity and thickness, if delayed locomotion is not enough to select for a functional difference in neonate limb bones). However, the data here do not bear out these predictions, instead indicating that the functional relationship between cortical thickness and maturity of neonates is more ambiguous.

With respect to thickness of the cortical bone, neonates of some taxa do fit the model described above. The femur of the semi-precocial Western gull chick is thicker than the humerus (by absolute and relative measures), matching Carrier & Leon’s (1990) observations in the California gull. The precocial California quail neonate also has a thicker femoral than humeral cortex by absolute measure. However, in the wild turkey chick, the humerus is absolutely thicker than the femur, and in the altricial Western scrub-jay and green-cheeked conure, the femur is absolutely and relatively thicker than the humerus. Additionally, the highest values of relative cortical thickness of both elements are predominantly seen in the altricial birds. Instead, precocial birds appear to compensate for greater functional demands on the pelvic limb simply by having more mature bone in the femur than the humerus (though this is not unique to precocial chicks, as discussed in greater detail below).

The idea of cortical thickness compensating for a lack of structural strength is born out by three of the four precocial taxa represented in this dataset, but this does not fully explain why the pattern of the femoral neonate cortex being thicker than the humeral does not appear in the precocial wild turkey, or why it does appear in altricial taxa. These differences in cortical thickness may not be a functional adaptation at all, instead representing a phylogenetic constraint as implied by how common this trait is in the diverse array of birds sampled in this study. On the other hand, Carril, Tambussi & Rasskin-Gutman, 2021 purport that the early ossification of the pelvic limb relative to the pectoral limb in altricial monk parakeet chicks is adaptive because chicks engage in moderate walking movements while still in the nest and before fledging (Aramburú, 1997). If such behavior is also present in other altricial chicks, this would potentially account for the patterns of bone maturity observed here. Future studies of locomotory behavior in chicks may help reveal whether or not it is adaptive to have a thicker femoral cortex than humeral at hatching.

Ultimately, it is striking that no distinct pattern of differences in either measure of cortical thickness was observed across the altricial-precocial spectrum. RCT of precocial and semi-altricial chicks falls within a similar range, as does RCT of semi-precocial and altricial chicks (Fig. 5B). Results for ACT across developmental modes are similar, though the differences not as great (Fig. 5A). Whatever contributes to variation in cortical thickness, it does not appear related to place along the altricial-precocial spectrum.

In contrast to cortical thickness, relative maturity of the bone at hatching does appear related to developmental mode. In precocial taxa, the bone of the humerus and femur was more mature (as inferred from the relatively smaller vascular channels and more advanced state of fibrolamellar development), while the most immature bone was observed in more altricial neonates (with very thin cortices, large vascular openings, thin trabeculae of woven bone, and in some cases totally cartilaginous elements at hatching). Again, this very likely reflects differences in functional demands and growth rates. Starck & Ricklefs (1998) have reported higher overall rates of growth at the altricial end of the developmental spectrum and lower rates at the precocial end. Ricklefs (1979) specifically identified an inverse relationship between growth rate and maturity of a tissue. Kirkwood et al. (1989) demonstrate that an increase in tibiotarsal length occurs at an average rate three times greater in altricial hatchlings than in precocial neonates. The more immature bone of altricial chicks indicates a relatively higher growth rate of the humerus and the femur than in precocial chicks. The more mature bone of precocial chicks is evidence of the higher functional demands placed on neonates that must locomote, find food (in some cases), and maintain at least some degree of independence very soon after hatching.

However, when comparing relative maturity of the femur and humerus of the same individual, another pattern becomes apparent: in all of the birds in this dataset for which a neonate specimen was available, the tissue of femur at hatching is comparatively more mature than the humerus, having relatively smaller vascular channels and a more organized bone matrix. In the case of the house finch, this difference is manifest as only the femur being ossified enough to be included in this study, while the humerus was still entirely cartilaginous. At a proximate level, this difference in maturity is indicative of varying growth rates between these two major limb bones. Ricklefs (1979) reported that degree of functional maturity of a tissue is inversely related to rate of growth, a hypothesis that has been substantiated by subsequent studies (*e.g*., Montes et al., 2005). Others describe a an inverse, negative relationship between high growth rates and strength of ossified tissues (Carrier & Leon, 1990; de Margerie et al., 2004), indicating a functional constraint on the relationship between growth rate and maturity, an idea further supported by differences in bone maturity of altricial and precocial neonates observed here. Therefore, the more immature bone of the humerus suggests that this element grows at a faster rate than the femur. It is likely that the difference in bone maturity between the humerus and femur is partly due to early functional demands placed on the femora of precocial taxa (both bones are more mature in precocial than altricial hatchlings); however, the presence of this characteristic in all birds across the altricial-precocial spectrum indicates it may also be the result of ontogenetic (wherein possible phenotypes are constrained by the developmental program) or phylogenetic channeling.

Precocial and semi-precocial birds may have, through selection, exapted and exaggerated this pre-existing characteristic to facilitate the chicks’ lifestyle, but the data presented here suggest this difference in maturity may initially (and in other developmental modes) represent a non-adaptive trait. Alternatively, as suggested by Carril, Tambussi & Rasskin-Gutman (2021), this may in fact be adaptive in even altricial chicks contingent upon the degree of in-nest locomotion that they exhibit. In either case, it is notable that a growing body of histological studies of Mesozoic birds and non-avialan theropods hints that a greater functional maturity of the hindlimb relative to the forelimb may have evolutionary origins even deeper than crown group birds (*e.g*., Prondvai et al., 2018).

Ultimately, the only consistent histological patterns related to developmental mode is the maturity of femoral and humeral bone together at the time of hatching (precocial chicks have more mature bone than altricial). However, because this characteristic is continuous and not discrete, it is difficult to define a precise way of predicting developmental mode from qualitative assessment of neonate histology alone, perhaps outside of the two most extreme ends of the altricial-precocial spectrum. The bone of precocial taxa appears more mature at hatching than that of altricial chicks, however, altricial chicks pass through stages later in their long bone ontogeny where they resemble the bone of precocial chicks, suggesting that this difference results from changes to pre-natal onset of bone growth. More altricial chicks have accomplished less growth in the egg, but their post-natal growth trajectory is still very similar to that of precocial chicks, only with a different post-hatching ‘starting point.’ That is, altricial chicks will undergo ontogenetic stages where their bone resembles that of precocial chicks, but have to first experience early stages of post-natal growth that have already been achieved by more precocial chicks at the time of hatching.

### Body size and offset of bone growth

The presence of the OCL is conventionally interpreted as a signal of reduction or cessation of growth, an indicator that skeletal maturity has been achieved (Chinsamy & Elzanowski, 2001; de Ricqlès et al., 2003; Chinsamy et al., 2013). In this study, where the “adult” growth stage was based on plumage and body size, we were able to test the relationship between “adult” external morphology and skeletal maturity.

An OCL was observed in both the humerus and femur of all taxa in this dataset for which an adult sample was available. However, in almost all instances, the OCL was also observed at earlier growth stages in both elements. In eight of the nine taxa for which mid-to late-growth stages were available, the OCL first appears at the pre-fledgling (house finch), fledging (white-tailed kite, barn owl, great-horned owl, Anna’s hummingbird, green-cheeked conure, Western scrub jay) or subadult (mourning dove) stage (Table 1). In nearly all first appearances, the OCL is incipient, comprising a very thin portion of the periosteal cortex that is parallel-fibered and avascular. However, in some instances a fully-developed, ‘mature’ OCL was present before the adult stage. Anna’s hummingbird represents the most drastic example of this, with a very thick OCL in the cortex of the fledgling humerus. In all such cases, the diametric size of the humerus or femur is comparable to that of the adult. Therefore, these examples suggest that appositional growth ceases at a relatively early developmental stage, while the bone continues to change and mature with a parallel-fibered matrix becoming more prevalent than a woven matrix or chondroid tissue, and endosteal resorption still active. Not only is an OCL decisively not a sign of senescence (as also argued by Woodward et al., 2020), but the presence of an OCL does not always even imply that an individual has achieved cortical maturity and does not necessarily correlate with the presence of “adult” gross morphology, further underscoring the complexity of defining “adulthood.” In short, skeletal maturity cannot be equated with the cessation of appositional growth.

Additionally, the microanatomical features of the middle cortical layer (adjacent to the OCL) of “adults” in this study was distinct relative to all earlier growth stages. Prior studies describe the cortical bone of modern birds as generally composed of a layer of highly-vascularized fibrolamellar bone (de Ricqlès, 1975; de Ricqlès et al., 1991; Castanet et al., 1996), though de Margerie, Cubo & Castanet (2002) do describe avascular to poorly-vascularized parallel-fibered bone in the humerus of a mallard. A cortex mainly composed of fibrolamellar bone was observed in many skeletally mature individuals in this dataset, but not all. Specifically, this best describes the larger-bodied birds (the wild turkey, Western gull, red-tailed hawk, barn owl, and great-horned owl). The adult histology of these taxa corroborates the known description of crown-group birds growing very quickly until adult size is achieved, then rapidly slowing down, resulting in the familiar pattern of a fibrolamellar cortex (reflecting early fast growth) and a parallel-fibered OCL and ICL (reflecting the later short stage of slower growth).

However, this pattern becomes less clear as body size decreases. In birds of medium body-size (quail, American kestrels, white-tailed kites, and mourning doves), the middle cortical layer is composed of a woven or parallel-fibered matrix with regional fibrolamellar bone or incipient primary osteons. The fast growth of earlier developmental stages is not as clearly reflected in these adults. A true fibrolamellar complex never quite fully forms in these birds and seems to only ever achieve early stages of development with, at most, regional and/or incipient fibrolamellar bone present.

In small birds (house finch and green-cheeked conure), the long bones are composed of a thin, middle layer of a woven matrix with simple vascular canals, between a strongly parallel-fibered OCL and ICL. Vascularity is also reduced, with relatively sparse longitudinal canals present. Finally, in the smallest bird in this dataset (Anna’s hummingbird), the cortices of the humerus and femur are avascular and composed entirely of parallel-fibered bone. In short, the spectrum of body sizes parallels the spectrum of structural organization of bone, ranging from fibrolamellar to parallel-fibered with a variety of intermediates between the two. In smaller birds, the OCL appears to grow so thick that, combined with endosteal resorption, it ultimately comprises the majority or the entirety of the cortex.

This trend of apparently slower growth in small-bodied taxa that belong to clades including large, fast-growing taxa (*e.g*., dinosaurs, pterosaurs, and mammals) has also been reported in other groups and is not unique to crown-group birds (Case, 1978; Padian, de Ricqles & Horner, 2001; Padian, Horner & de Ricqlès, 2004; Lehman & Woodward, 2008). These patterns provide evidence that most birds in this dataset follow an ontogenetic trajectory of very fast growth in early stages (neonate to pre-fledgling), which abruptly slows or even stops at later stages (pre-fledgling to subadult), followed by a period dominated by bone maturation with little new appositional growth. This supports the idea suggested by Padian, Horner & de Ricqlès (2004) that avialans evolved a relatively small body size by truncating the early period of fast growth. Furthermore, this appears to be a growth strategy that has been manipulated and exaggerated repeatedly in the evolution of increasingly smaller birds. Histological traits of small-bodied taxa studied here indicate that the period of fast growth is much shorter than in large-bodied taxa. This pattern is most extreme in Anna’s hummingbird, in which a fully-formed OCL is present by the fledgling stage in the humerus, and the adult cortex is completely parallel-fibered. However, these small birds still ultimately complete their growth fast enough *not* to lay down any LAGs or other growth marks, as none were observed in the adults of this dataset.

What is notable is that this ontogenetic trend seems entirely driven by body size, and independent of developmental mode; birds of medium to small body size across the altricial-precocial spectrum follow this model. This is not to say that these taxa share similar growth rates, however. It has been well-documented that birds on the precocial end of the spectrum grow at lower rates than those at the altricial end (Ricklefs, 1968; Ricklefs, 1973; Starck, 1989; Starck, 1993; Starck & Ricklefs, 1998). Instead, this seems to result from a manipulation in the timing of off-set of bone growth (a truncated period of fast growth followed by a relatively longer period of slowed growth and bone maturation) in small-bodied birds across the altricial-precocial spectrum. While absolute growth rates of more altricial taxa are undoubtedly higher during the fast growth phase, any taxon that only has to achieve small body size still employs the strategy of truncating this part of the growth trajectory. In parallel to this and as described above, data presented here also suggest that change in the timing of onset of bone growth *is* related to place along the altricial-precocial spectrum.

## Conclusions

We tested hypotheses that cortical thickness is used to compensate for biomechanical weakness of woven bone in the hindlimbs of precocial chicks, and that precocial chicks exclusively have more mature bone in the femur than the humerus at the time of hatching. No evidence was found that unequivocally supports either prediction. While most precocial taxa in this dataset have thicker femoral cortices than humeral at hatching, this relationship was also observed in most other taxa, and irrespective of place along the altricial-precocial spectrum, the femoral bone of most neonates was comparatively more mature than that of the humerus. Once again, this indicates a difference in relative growth rates, and is a somewhat surprising result given the different functional demands on altricial and precocial chicks based on behavior and primary locomotor module. This may instead represent developmental channeling in avian growth, a feature with such deep evolutionary history that it is a nearly indelible part of their ontogeny. While maturity of these two limb bones later becomes coincident, as reported here and by others (*e.g*., Starck & Ricklefs, 1998), the initial difference appears to be common to all or most avians. Furthermore, differences in cortical thicknesses across growth stages and within single elements suggest that width of the cortex may represent a non-adaptive feature of immature bird bones. Ultimately, there are in fact fewer histological differences along the altricial-precocial spectrum than anticipated, and it is difficult based on qualitative descriptions to identify a clear histological signal of developmental mode beyond relative maturity of both skeletal elements at the extreme ends of the spectrum. However, it is very possible that quantitative analyses of histological attributes would be more successful in parsing out finer categories along the altricial-precocial spectrum.

If relative growth rates can be inferred from tissue maturity as many previous studies suggest (*e.g*., Ricklefs, 1979; Kirkwood et al., 1989; Carrier & Leon, 1990; Montes et al., 2005) this research does support the conclusion of lower growth rates in precocial neonates, and higher growth rates in altricial neonates. The humerus and femur of chicks at the precocial end of the developmental spectrum were observed to be more mature than that of the humerus and femur respectively in chicks along the altricial end of the spectrum. This not only reflects the faster growth of altricial chicks, but also the higher functional demands on precocial chicks, which need to locomote and be relatively independent at an earlier age.

Bone growth appears to be a balance between growth rate, structural weakness/strength, and functional demands. This aspect of life history is a compromise between functional and developmental channeling, and it is the interplay of these two constraints that limits viable growth trajectories. However, within these constraints, different taxa alter their developmental trajectories by changing the timing of the onset and offset of bone growth. The bone of precocial chicks is more functionally mature than that of altricial neonates because of the biomechanical demands that come with precociality. The onset of bone growth is affected by developmental mode, with precocial chicks accomplishing more growth in the egg than altricial. Altricial chicks ultimately follow a similar trajectory of bone development, but in a shorter span of time (hatching to adult). On the other hand, timing of the offset of bone growth is independent of developmental mode, and instead changes with adult body size. Smaller birds achieve smaller body size by truncating their period of fast growth, parallel to the strategy used by avialans to evolve a small body size relative to their dinosaurian ancestors. As a result, small taxa achieve appositional “adult” size by the pre-fledgling to subadult stage when the OCL appears. However, this does not correlate with complete skeletal maturity. Instead, the tissue continues to change and mature through a combination of endosteal resorption, increased organization of bone texture (including a thickening of the OCL), and a reduction in vascularity.

Avian osteological development is clearly influenced by the canonical triad of constraints: functional, phylogenetic, and developmental (Seilacher, 1970; Gould, 2002). Functionally, the demands of locomotion (to varying degrees across the developmental spectrum) and strain on bone are high but are necessarily compromised by the need for fast growth which results in weaker bone. Phylogenetically, several histological characteristics observed are correlated with particular clades, including cortical thickness and unique features (*e.g*., intercortical gap left by the Kastschenko line in passeriforms). And developmentally, all birds are constrained by high growth rates and consequently weak bones, as well as ontogenetically channeled attributes, such as relatively more mature bone in the femur compared to the humerus at hatching.

This research lays the groundwork for future studies, such as longitudinal sections of the samples described here, sections from other skeletal elements, a detailed description of the presence of chondroid bone, quantification of additional microstructural features (*e.g*., vascular porosity and size and shape of osteocyte lacunae), inclusion of more specimens and taxa, and further investigation into the functional and phylogenetic differences between developmental modes and avian clades to gain greater insight into the influence of these factors on bone growth and evolution.

## Supplemental Information

10.7717/peerj.12160/supp-1 Supplemental Information 1 Supplemental material and descriptions of histological changes through post-natal ontogeny of individual taxa.

10.7717/peerj.12160/supp-2 Supplemental Information 2 A list of specimens for each taxon sampled in this study, along with growth stage classifications, and measurements taken from histological sections. All measurements reported are averages for each specimen; units are micrometers. Top value for each parameter is for the humerus, and bottom is for the femur. 2C:T = relative cortical thickness, calculated as two (average cortical thickness)/average cross-sectional diameter.

10.7717/peerj.12160/supp-3 Supplemental Information 3 The classification system of developmental mode used in this study, based on differences in chick morphology and behavior, and parent behavior. Note that this is simply a helpful discretization of a continuous spectrum. Reproduced from Starck & Ricklefs (1998).

10.7717/peerj.12160/supp-4 Supplemental Information 4 Body mass of the neonate and adult specimens used in this study, along with sex (in cases where gonads were identifiable during specimen preparation).

10.7717/peerj.12160/supp-5 Supplemental Information 5 Macroscopic view of ostrich growth series, transverse sections of humeri. ~2w = approximately 2 weeks old, MVZ190729; other specimens from smallest to largest: MVZ190727, MVZ190731, MVZ190732. Cr, cranial; L, Lateral. Scale bar equals 1,000 µm

10.7717/peerj.12160/supp-6 Supplemental Information 6 Growth series of ostrich humeri. (A) Humerus of approximately 2-week-old chick (MVZ190729) showing vascular canals nearer the endosteal surface are larger and more irregularly shaped than those towards the periosteal surface. The latter were in the early stages of primary osteon formation. There is an abundance of mature osteocytes within the neonate cortex. Plane light, 200×. (B) Humerus of MVZ190733 with a cortex of woven bone similar to the neonate, but with a thicker cortex and a periosteal vascular canals fully closed-off from to the outside. Plane light, 200×. (C) Humerus of MVZ190727 with characteristics similar to B. Plane light, 200×. (D) Humerus of MVZ190731 showing densely-packed, early-stage longitudinal primary osteons within a woven matrix, forming incipient fibrolamellar bone. This is especially concentrated in the endosteal half of the cortex. Plane light, 100×. (E) Humerus of MVZ190734 with characteristics similar to D. Plane light, 100×. (F) Humerus of MVZ190732,with an incipient inner circumferential layer and sparsely scattered secondary osteons (arrow). Plane light, toluidine blue stain, 100×. In all images the periosteal surface is oriented to top/left and the endosteal to the bottom/right. Scale bare equals 100 µm.

10.7717/peerj.12160/supp-7 Supplemental Information 7 Macroscopic view of ostrich growth series, transverse sections of femora. ~2w = approximately 2 weeks old, MVZ190729; other specimens from smallest to largest: JAA74, MVZ190731, MVZ190732. Cr, cranial; L, Lateral. Scale bar equals 1,000 µm.

10.7717/peerj.12160/supp-8 Supplemental Information 8 Growth series of ostrich femora. (A) Femur of approximately 2-week-old chick (MVZ190733) composed of disorganized woven bone with large vascular canals, though mature enough that those at the periosteal border no longer communicate with the exterior. Plane light, toluidine blue stain, 200×. (B) Femur of MVZ190729. Plane light, toluidine blue stain, 200×. (C) Femur of MVZ190734 showing increased organization of the developing bone with the incipient fibrolamellar bone concentrated in periosteal half of the cortex. Plane light, toluidine blue stain, 100×. (D) Femur of MVZ190727 composed of woven bone with newly-formed primary osteons and incipient primary osteons. Plane light, 200×. (E) Femur of MVZ190731 showing increasingly mature bone with substantially lower vascular area than earlier growth stages and numerous primary osteons. Plane light, 40×. (F) Femur of MVZ190732 with a mature fibrolamellar complex, charachterized by a predominance of circumferential canals in the endosteal half of the cortex. Plane light, 100×. In all images the periosteal surface is oriented to top/left and the endosteal to the bottom/right. Scale bare equals 100 µm.

10.7717/peerj.12160/supp-9 Supplemental Information 9 Macroscopic view of California quail growth series, transverse sections of humeri. n = neonate, MVZ190743; p = pin-feathered chick, MVZ190761; a = adult, MVZ190749. Cr, cranial; L, Lateral. Scale bar equals 500µm.

10.7717/peerj.12160/supp-10 Supplemental Information 10 Growth series of California quail humeri. (A) Neonate humerus (MVZ190751) composed of woven bone with irregulary-oriented vascular canals. Plane light, toluidine blue stain, 200×; (B) Neonate humerus (MVZ190750) of woven bone with large vascular canals. Plane light, 200×. (C) Neonate humerus (MVZ190745), showing thicker cortex of woven bone and irregularly-oriented canals. Plane light, toluidine blue stain, 200×. (D) Humerus of a pin-feathered chick (MVZ190761) showing woven bone with a smooth periosteal edge and organized orientation of vascular canals (mostly longitudinal). Plane light, 200×. (E) Adult humerus (MVZ190762) with a weakly-formed fibrolamellar complex. Plane light, 100×. (F) Adult humerus (MVZ190749) showing a thick OCL of parallel-fibered bone adjacent to a mid-cortical layer of woven bone with simple vascular canals. Polarized light, 100×. In all images the periosteal surface is oriented to top/left and the endosteal to the bottom/right. Scale bare equals 100 µm.

10.7717/peerj.12160/supp-11 Supplemental Information 11 Macroscopic view of California quail growth series, transverse sections of femora. n = neonate, MVZ190745; p = pin-feathered chick, MVZ190761; a = adult, MVZ190762. Cr, cranial; L, Lateral. Scale bar equals 250 µm.

10.7717/peerj.12160/supp-12 Supplemental Information 12 Growth series of California quail femora. (A) Neonate femur (MVZ190743) composed of incipient fibrolamellar bone. Plane light, 200×. (B) Neonate femur (MVZ190751) showing woven bone and variously oriented vascular canals. Plane light, toluidine blue, 200×. (C) Neonate femur (MVZ190750) with a very thin cortex composed of woven bone. Plane light, toluidine blue stain, 200×. (D) Femur of a pin-feathered chick (MVZ190761) with woven bone and incipient osteons. Plane light, 200×. (E) Adult femur (MVZ190762) showing a strongly-developted ICL adjacent to a region of weakly-fibrolamellar bone; a thick parallel-fibered region is located periosteally. Plane light, 100×. (F) Adult femur (MVZ190749) with characteristics similar to E but showing a mid-cortical region of woven bone with simple vascular canals instead of fibrolamellar bone (though both individuals had regions of both in the femur). Plane light, 100×. In all images the periosteal surface is oriented to top/left and the endosteal to the bottom/right. Scale bare equals 50 µm.

10.7717/peerj.12160/supp-13 Supplemental Information 13 Macroscopic view of wild turkey growth series, transverse sections of humeri. n = neonate, MVZ190764; 4–6 = 4-to 6-week-old chick, MVZ190765; a = adult, MVZ190763. Arrow indicates loop of ICL, which housed the vessel penetrating the bone in *via* nutrient formation (see Fig. 10E). Cr, cranial; L, Lateral. Scale bar equals 1,000 µm.

10.7717/peerj.12160/supp-14 Supplemental Information 14 Growth series of wild turkey humeri. (A) Neonate humerus (MVZ190764) showing disorganized woven bone with large, irregular vascular canals. Plane light, toluidine blue stain, 200x. (B) Humerus of a 4-to 6-week-old chick (MVZ190765), composed of woven bone with developing primary osteons; levels of vascularity remain high. Plane light, 200×. (C) Adult humerus (MVZ190763), showing OCL and ICL and a predominance of laminar vascular spaces. Plane light, 40×. (D) Adult humerus (MVZ190763) with mature fibrolamellar bone; arrow indicates the parallel-fibered ICL. Plane light, 200×. (E) Adult humerus (MVZ190763) showing the mediocaudal margin of the cortex with a predominance of longitudinally-oriented vascular canals; arrow indicates the nutrient foramen penetrating the bone. Plane light, toluidine blue stain, 40×. In all images the periosteal surface is oriented to top/left and the endosteal to the bottom/right. In all images the periosteal surface is oriented to top/left and the endosteal to the bottom/right. Scale bare equals 100 µm.

10.7717/peerj.12160/supp-15 Supplemental Information 15 Macroscopic view of wild turkey growth series, transverse sections of femora. n = neonate, MVZ190764; 4–6 = 4-to 6-week-old chick, MVZ190765; a = adult, MVZ190763. Note medullary bone lining endosteal surface of the adult femur (white arrow). Cr, cranial; L, Lateral. Scale bar equals 1,000 µm.

10.7717/peerj.12160/supp-16 Supplemental Information 16 Growth series of wild turkey femora. (A) Neonate femur (MVZ190764) of woven bone with large, longitudinally-oriented vascular canals (some, visible here, are incipient primary osteons); arrow indicates part of the endosteum seen in the endosteal cavity of this specimen. Plane light, 200×. (B) Femur of a 4-to 6-week-old chick (MVZ190765) showing the development of a fibrolamellar complex, with woven bone with incipient primary osteons. Plane light, toluidine blue stain, 200×. (C) Adult femur (MVZ190763). Plane light, 200×. (D) Adult femur (MVZ190763), showing circumferentially-oriented vascular canals and medullary bone (arrow). Plane light, 40×. (E) Adult femur (MVZ190763), close-up of medullary bone (right) and parallel-fibered bone of the ICL (left). Note localized Haversian system in the adult femur; this was one of very few areas of secondary growth observed in this study. Plane light, 200×. In all images the periosteal surface is oriented to top/left and the endosteal to the bottom/right. Scale bare equals 100 µm.

10.7717/peerj.12160/supp-17 Supplemental Information 17 Macroscopic view of Anna’s hummingbird growth series, transverse sections of humeri. p = pin-feathered chick, MVZ190799; f = fledgling, MVZ190802; a = adult, MVZ190807. Cr, cranial; L, lateral. Scale bar equals 100 µm.

10.7717/peerj.12160/supp-18 Supplemental Information 18 Growth series of Anna’s hummingbird humeri (shown at 200–400 times actual size). (A) Humerus of a pin-feathered chick (MVZ190799) showing a very thin cortex of woven bone. Almost no vascular canals were fully enclosed with bone. Plane light, toluidine blue stain, 400×. (B) Humerus of fledgling chick (MVZ190802) showing cortex composed periosteally of parallel-fibered bone and endosteally of woven bone. Note scalloped endosteal margin, indicative of the presence of vascular structures. Plane light, toluidine blue stain, 400×. (C) Humerus of a sub-adult (MVZ190803) showing the cortex of the developing bicipital crest with a very thick endosteal layer of woven adjacent to the parallel-fibered bone of the cortex. Plane light, toluidine blue stain, 400×. (D) Humerus of a sub-adult (MVZ190800) showing a transitional region of the cortex with a clear outer layer of parallel-fibered bone (with low osteocyte lacuna density) and an inner layer of woven bone transitioning to parallel-fibered. Plane light, toluidine blue stain, 200×. (E) Humerus of a sub-adult (MVZ190808) along the diaphyseal cortex, composed of parallel-fibered bone with low-cellularity in the periosteal portion and woven bone transitioning to parallel-fibered endosteally. There appears to be an outer layer of highly organized bone that resembles an OCL, but disappears in the adult. Plane light, toluidine blue stain, 200×. F) Adult humerus (MVZ190807) composed of parallel-fibered bone with higher numbers of osteocyte lacunae than observed in sub-adult individuals. In all images the periosteal surface is oriented to top/left and the endosteal to the bottom/right. Plane light, toluidine blue stain, 200×. Scale bare equals 50 µm.

10.7717/peerj.12160/supp-19 Supplemental Information 19 Macroscopic view of Anna’s hummingbird growth series, transverse sections of femora. p = pin-feathered chick, MVZ190799; f = fledgling, MVZ190802; a = adult, MVZ190807. Cr, cranial; L, lateral. Scale bar equals 100 µm.

10.7717/peerj.12160/supp-20 Supplemental Information 20 Growth series of Anna’s hummingbird femora. (A) Femur of a pin-feathered chick (MVZ190799) composed of a thin layer of woven bone. Fully-developed vascular canals are sparse (only two are visible here), but the scalloped peri-and endosteal margins are indicative of the presence of vascular structures not fully enclosed in bone. Plane light, toluidine blue stain, 400×. (B) Femur of a fledgling chick (MVZ190802) showing parallel-fibered bone lined by a thin, irregular layer of chondroid bone (arrow). Plane light, toluidine blue stain, 400×. (C) Femur of a sub-adult (MVZ190803) composed of parallel-fibered bone with sparse osteocyte lacunae. An incipient ICL is present in this individual (arrow). Plane light, toluidine blue stain, 400×. (D) Femur of a sub-adult (MVZ190800) showing parallel-fibered bone with sparse osteocyte lacunae. Plane light, toluidine blue stain, 400×. (E) Femur of a sub-adult (MVZ190808) with characteristics similar to D. Plane light, toluidine blue stain, 400×. (F) Femur of an adult (MVZ190807) composed of highly-cellularized, avascular, parallel-fibered bone. An ICL is also visible (arrow). Plane light, toluidine blue stain, 400×. In all images the periosteal surface is oriented to top/left and the endosteal to the bottom/right. Scale bare equals 50 µm.

10.7717/peerj.12160/supp-21 Supplemental Information 21 Macroscopic view of mourning dove growth series, transverse sections of humeri. p = pin-feathered chick, MVZ190778; pf = pre-fledgling chick, MVZ190774; f = fledgling chick, MVZ190779; sa = sub-adult, MVZ190783; a = adult, MVZ190775. Cr, cranial; L, lateral. Scale bar equals 500 µm.

10.7717/peerj.12160/supp-22 Supplemental Information 22 Growth series of mourning dove humeri. (A) Humerus of a pin-feathered chick (MVZ190778) with highly porous woven bone. Plain light, toluidine blue stain, 400×. (B) Humerus of a pre-fledgling chick (MVZ190784) showing a thick cortext of woven bone with large, irregular vascular canals and a thick layer of endosteum. Plain light, toluidine blue stain, 200×. (C) Humerus of a pre-fledgling chick (MVZ190774) showing woven bone with irregular vascular canals; these spaces are still large but are becoming smaller as the bone separating them expands. Plain light, 400×. (D) Humerus of a fledgling chick (MVZ190785) showing bone maturing into a fibrolamellar complex with many incipient osteons in a matrix of woven bone. Plane light, toluidine blue stain, 400×. (E) Humerus of a fledgling chick (MVZ190779) with characteristics similar to D. Plane light, toluidine blue stain, 400×. (F) Humerus of a sub-adult (MVZ190780), composed of mature fibrolamellar bone with an incipient an OCL. Plane light, toluidine blue stain, 200x. G) Humerus of a sub-adult (MVZ190783) showing fibrolamellar bone with an incipient OCL; there is also a layer of endosteum. Plane light, toluidine blue stain, 100×. (H) Humerus of an adult (MVZ190775) composed of fibrolamellar bone with very small vascular canals, within a parallel-fibered OCL. Plane light, 100×. In all images the periosteal surface is oriented to top/left and the endosteal to the bottom/right. Scale bare equals 50 µm.

10.7717/peerj.12160/supp-23 Supplemental Information 23 Macroscopic view of mourning dove growth series, transverse sections of femora. p = pin-feathered chick, MVZ190778; pf = pre-fledgling chick, MVZ190784; f = fledgling chick, MVZ190779; sa = sub-adult, MVZ190783; a = adult, MVZ190775. Cr, cranial; L, lateral. Scale bar equals 500 µm.

10.7717/peerj.12160/supp-24 Supplemental Information 24 Growth series of mourning dove femora. (A) Femur of a pin-feathered chick (MVZ190778), composed of very thin woven bone demarcating large, irregular vascular canals; most are longitudinal in orientation . Plane light, toluidine blue stain, 200×. (B) Femur of a pre-fledgling chick (MVZ190784), also composed of woven bone but with increased bone deposition and smaller vascular spaces as a consequence. Plane light, toluidine blue stain, 200×. (C) Femur of a pre-fledgling chick (MVZ190774) showing features similar to B. Plane light, toluidine blue stain, 200×. (D) Femur of a fledgling chick (MVZ190785) showing woven bone with incipient primary osteons and smaller vascular canals than the pre-fledgling stage. Plane light, toluidine blue, 200×. (E) Femur of a fledgling chick (MVZ190779) showing a region of fibrolamellar bone with weakly-formed primary osteons. Plane light, toluidine blue, 200×. (F) Femur of the less mature individual classified as “sub-adult” (MVZ190780) showing a thinning cortext composed of woven bone with weakly-formed primary osteons. Plane light, toluidine blue, 200×. (G) Femur of the more mature individual classified as “sub-adult” (MVZ190783) with vascular canals that are small but still numerus; a thin, outer layer resembling an OCL is visible. Plane light, toluidine blue stain, 200×. (H) Femur of an adult (MVZ190775) showing a cortex of parallel-fibered/weakly-woven bone with very few, small, primary vascular canals. An ICL is also visible, as well as a very thin layer possibly representing the OCL. Polarized light, 200×. In all images the periosteal surface is oriented to top/left and the endosteal to the bottom/right. Scale bare equals 50 µm.

10.7717/peerj.12160/supp-25 Supplemental Information 25 Macroscopic view of Western gull growth series, transverse sections of humeri. n = neonate, MVZ190822; a = adult, MVZ190831. Arrow indicates trabecular loop, which likely enclosed structures from a nutrient foramen. Cr, cranial; L, lateral. Scale bar equals 500 µm.

10.7717/peerj.12160/supp-26 Supplemental Information 26 Growth series of Western gull humeri. (A) Humerus of a neonate (MVZ190824), composed of woven bone with large, irregular vascular canals. Arrow indicates periosteum. Plane light, toluidine blue stain, 400×. (B) Humerus of a neonate (MVZ190822), also composed of woven bone with large, irregular vascular openings. Arrow indicates endosteum. Plane light, 400×. (C) Humerus of an adult (MVZ190829) showing a cortex of fibrolamellar bone between an OCL and ICL of avascular, parallel-fibered bone. Several secondary osteons are present near the ICL (one is indicated by an arrow). Plane light, toluidine blue stain, 100×. (D) Humerus of an adult (MVZ190831) with remnants of medullary bone (arrows). Plane light, toluidine blue stain, 40×. In all images the periosteal surface is oriented to top/left and the endosteal to the bottom/right. Scale bare equals 50 µm.

10.7717/peerj.12160/supp-27 Supplemental Information 27 Macroscopic view of Western gull growth series, transverse sections of femora. n = neonate, MVZ190824; a = adult, MVZ190829. Cr, cranial; L, lateral. Scale bar equals 500 µm.

10.7717/peerj.12160/supp-28 Supplemental Information 28 Growth series of Western gull femora. (A) Femur of a neonate (MVZ190824), composed of woven bone with very large vascular canals of variable shape. Plane light, 400×. (B) Femur of a neonate (MVZ190822) showing a relatively low number of osteocyte lacunae, which are rounded and lack cannaliculi. Plane light, toluidine blue stain, 400×. (C) Femur of an adult (MVZ190829) composed of fibrolamellar bone with numerous primary and secondary osteons (one example of the latter is indicated by an arrow). Plane light, toluidine blue stain, 100×. (D) Femur of an adult (MVZ190831). Plane light, toluidine blue stain, 200×. (E) Adult femur (MVZ190831), close-up of bone marrow (yellow tissue above) and delicate medullary bone (purple tissue below). Plane light, toluidine blue stain, 400×. In all images the periosteal surface is oriented to top/left and the endosteal to the bottom/right. Scale bare equals 50 µm.

10.7717/peerj.12160/supp-29 Supplemental Information 29 Macroscopic view of American kestrel humeri growth series, transverse sections of humeri. n = neonate, MVZ190890; f = fledgling, MVZ190888; a = adult, MVZ190892. Cr = cranial; L = lateral. Scale bar equals 1,000 µm.

10.7717/peerj.12160/supp-30 Supplemental Information 30 Growth series of American kestrel humeri. (A) Neonate humerus (MVZ190890) of woven bone with large vascular spaces and a thin layer of dense tissue surrounding it (arrow). Plane light, 400×. (B) Fledgling humerus (MVZ190888) showing a cortex composed of fibrolamellar bone endosteally (with longitdinal primary osteons) and incipient fibrolamellar bone periosteally (with incipient primary osteons in a variety of orientations). Plane light, toluidine blue stain, 200×. (C) Adult humerus (MVZ190892) of the more mature individual, showing an ICL and an OCL bordering a region of parallel-fibered bone with low levels of vascularity. Plane light, toluidine blue stain, 200×. (D) Adult humerus (MVZ190885) of the less mature individual, showing one of the larger regions of fibrolamellar bone; also notable is the incipient OCL, thinner than that seen in C. Plane light, 200×. In all images the periosteal surface is oriented to top/left and the endosteal to the bottom/right. Scale bare equals 50 µm.

10.7717/peerj.12160/supp-31 Supplemental Information 31 Macroscopic view of American kestrel growth series, transverse sections of femora. n = neonate, MVZ190890; f = fledgling, MVZ190888; a = adult, MVZ190892. Cr, cranial; L, lateral. Scale bar equals 1,000 µm.

10.7717/peerj.12160/supp-32 Supplemental Information 32 Growth series of American kestrel femora. (A) Neonate femur (MVZ190887) showing the thinner region of the cortex, with relatively small, elongate vascular canals. Plane light, toluidine blue stain, 400×. (B) Neonate femur (MVZ190890) showing the thicker cortical bone with larger, more rounded vascular spaces and thinner struts of bone between them. Plane light, toluidine blue stain, 400×. (C) Fledgling femur (MVZ190888) showing an incipient fibrolamellar complex, with developing primary osteons and fairly large vascular spaces. Plane light, toluidine blue, 200×. D) Adult femur (MVZ190892) with few, small vascular canals; this part of the femur has fibrolamellar bone interspersed with parallel-fibered bone in the middle layer of the cortex. This individual appears to be a less-mature “adult,” with a more weakly-formed OCL. Plane light, 200×. (E) Adult femur (MVZ190885) mostly composed of parallel-fibered bone in this region of the cortex. This more mature adult has less fibrolamellar bone and a thicker OCL than the other “adult”. Plane light, toluidine blue stain, 200×. In all images the periosteal surface is oriented to top/left and the endosteal to the bottom/right. Scale bare equals 50 µm.

10.7717/peerj.12160/supp-33 Supplemental Information 33 Macroscopic view of green-cheeked conure growth series, transverse sections of humeri. n = neonate, MVZ190895; 2w6d = 2 weeks and 6 days old, MVA190898; 4w4d = 4 weeks and 4 days old, MVZ190903; 5w3d = 5 weeks and 3 days old, MVZ190904; a = adult, MVZ190917. Cr, cranial; L, lateral. Scale bar equals 250 µm.

10.7717/peerj.12160/supp-34 Supplemental Information 34 Growth series of green-cheeked conure humeri. (A) Neonate humerus (MVZ190895) composed of woven bone with few large vascular chanells and moderate scalloping along the endosteal and periosteal margins. Plane light, toluidine blue stain, 400×. (B) Humerus of a 1-week-old chick (MVZ190896) showing characteristics similar to A but with increased vascular porosity. Plane light, toluidine blue stain, 200×. C) Humerus of a 2-week, 6-day-old chick (MVZ190898) with a thicker cortex still composed of woven bone; more endosteal canals are smaller and longitudinal, while channels in the periosteal region are larger and more immature. Plane light, toluidine blue stain, 400×. (D) Humerus of a 4-week, 4-day-old chick (MVZ190903) showing bone similar to C, but with smaller vascular channels and developing primary osteons. Plane light, toluidine blue stain, 400×. (E) Humerus of a 5-week, 3-day-old chick (MVZ190904) composed predominantly of fibrolamellar bone, but with several simple vascular canals; the endosteum lining the medullary cavity is also shown. Polarized light, 400×. (F) Adult humerus (MVZ190917), showing a prominent OCL and thin ICL surrounding a cortex of woven bone with primary vascular canals. Plane light, toluidine blue, 400×. In all images the periosteal surface is oriented to top/left and the endosteal to the bottom/right. Scale bare equals 50 µm.

10.7717/peerj.12160/supp-35 Supplemental Information 35 Macroscopic view of green-cheeked conure growth series, transverse sections of femora. n = neonate, MVZ190895; 2w6d = 2 weeks and 6 days old, MVA190898; 4w4d = 4 weeks and 4 days old, MVZ190903; 5w3d = 5 weeks and 3 days old, MVZ190904; a = adult, MVZ190917. Cr, cranial; L, lateral. Scale bar equals 250 µm.

10.7717/peerj.12160/supp-36 Supplemental Information 36 Growth series of green-cheeked femora. (A) Neonate femur (MVZ190895), showing a thick cortex of woven bone with large, irregular vascular channels with moderately-scalloped endosteal and periosteal margins. Plane light, toluidine blue stain, 400×. (B) Femur of a 1-week-old chick (MVZ190896), composed of woven bone with large vascular canals; this element shows a trend of larger, more immature vascular spaces in the endosteal half of the cortex. Plane light, 400×. (C) Femur of a 2-week, 6-day-old chick (MVZ190898), similar to B but with greater in-filling of vascular spaces. Plane light, 400×. (D) Femur of a 4-week, 4-day-old chick (MVZ190903) composed of fibrolamellar bone with mature and incipient primary osteons. Plane light, toluidine blue stain, 400×. (E) Femur of a 5-week, 3-day-old chick (MVZ190904) composed of fibrolamellar bone with an OCL and an actively-resorbing endosteal margin. Plane light, 400×. (F) Adult femur (MVZ190917) showing an OCL and ICL adjacent to a layer of weakly-woven bone with primary osteons and simple vascular canals. Plane light, toluidine blue stain, 400×. In all images the periosteal surface is oriented to top/left and the endosteal to the bottom/right. Scale bare equals 50 µm.

10.7717/peerj.12160/supp-37 Supplemental Information 37 Macroscopic view of Western scrub jay growth series, transverse sections of humeri. n = neonate, MVZ190927; p = pin-feathered chick, MVZ190929; pf = pre-fledgling, MVZ190921; f = fledgling, MVZ190931; sa = sub-adult, MVZ190923. Cr, cranial; L, lateral (anatomical directions cannot be applied to neonate). Scale bar equals 250 µm.

10.7717/peerj.12160/supp-38 Supplemental Information 38 Growth series of Western scrub-jays. (A) Neonate humerus (MVZ190927) composed of woven bone with very large, irregular vascular spaces. Plane light, toluidine blue stain, 400×. (B) Humerus of a pin-feathered chick (MVZ190929) showing a cortex of woven bone largely similar to the neonate, but with smaller vascular canals and, as a consequence, thicker bone between vascular spaces. Plane light, toluidine blue stain, 200×. (C) Humerus of a pre-fledgling chick (MVZ190928), showing a region of circumferentially-oriented vascular channels, one example of the variation in vascular orientation observed at this growth stage. Plane light, toluidine blue stain, 400×. (D) Humerus of a pre-fledgling chick (MVZ190921) composed of woven bone, showing additional examples of variation in vascular channel orientation (here both longitudinal and reticular are seen). Plane light, toluidine blue stain, 200×. (E) Humerus of a pre-fledgling chick (MVZ190922) showing an incipient fibrolamellar complex, characteristic of this age group. Plane light, toluidine blue stain, 400×. (F) Humerus of a fledgling chick (MVZ190930) showing a region of parallel-fibered bone with low vascularity in the fibrolamellar cortex. Plane light, toluidine blue stain, 400×. (G) Humerus of a fledgling chick (MVZ190931) showing a cortex composed of fibrolamellar bone with small, longitudinal canals and an incipient OCL along the periosteal margin. Plane light, toluidine blue stain, 200×. (H) Humerus of a sub-adult (MVZ190923) showing microanatomical characteristics very similar to F and G. Plane light, 200×. In all images the periosteal surface is oriented to top/left and the endosteal to the bottom/right. Scale bare equals 50 µm.

10.7717/peerj.12160/supp-39 Supplemental Information 39 Macroscopic view of Western scrub jay growth series, transverse sections of femora. n = neonate, MVZ190927; p = pin-feathered chick, MVZ190929; pf = pre-fledgling, MVZ190921; f = fledgling, MVZ190931; sa = sub-adult, MVZ190923. Cr, cranial; L, lateral (anatomical directions cannot be applied to neonate). Scale bar equals 250 µm.

10.7717/peerj.12160/supp-40 Supplemental Information 40 Growth series of Western scrub-jay femora. (A) Neonate femur (MVZ190927) showing a thick cortex of woven bone with high vascular porosity. Plane light, toluidine blue stain, 400×. (B) Femur of a pin-feathered chick (MVZ190929) showing immature bone generally similar to A but with a thinner cortical wall. Plane light, toluidine blue stain, 200×. (C) Femur of a pre-fledgling chick (MVZ190928) composed of woven bone with numerous incipient primary osteons, predominantly longitudinal in orientation in this cortical region of this individual. Plane light, toluidine blue stain, 400×. (D) Femur of a pre-fledgling chick (MVZ190921), showing diversity of orientation of vascular canals at this growth phase; here, there are anastomosing and circumferential channels. Plane light, toluidine blue stain, 400×. (E) Femur of a pre-fledgling chick (MVZ190922) showing a region of more immature bone (woven but without incipient primary osteons), highlighting osteohistological variation in and among individuals at this growth stage. Plane light, 400×. (F) Femur of a fledgling chick (MVZ190930) showing a patch of parallel-fibered bone, and primary osteons clustered near the endosteal margin. Plane light, toluidine blue stain, 400×. (G) Femur of a fledgling chick (MVZ190931) showing fibrolamellar bone . Plane light, toluidine blue stain, 200×. (H) Femur of a sub-adult (MVZ190923) showing characteristics broadly similar to F and G, with a marginally thinner cortex. Plane light, toluidine blue stain, 200×. In all images the periosteal surface is oriented to top/left and the endosteal to the bottom/right. Scale bare equals 50 µm.

10.7717/peerj.12160/supp-41 Supplemental Information 41 Macroscopic view of house finch growth series, transverse sections of humeri. p = pin-feathered chick, MVZ190984; pf = pre-fledgling, MVZ190994; f = fledgling, MVZ190991; a = adult, MVZ190993. Cr, cranial; L, Lateral. Scale bar equals 250 µm.

10.7717/peerj.12160/supp-42 Supplemental Information 42 Growth series of house finch humeri. (A) Humerus of a pin-feathered chick (MVZ190984) composed of highly-porous woven bone, lined by a thin circlet of endosteal bone. Plane light, toluidine blue stain, 200×. (B) Humerus of a pre-fledgling chick (MVZ190967) showing a woven matrix with large vascular canals; these spaces are much smaller than in the pin-feathered stage. Plane light, toluidine blue stain, 400×. (C) Humerus of a pre-fledgling chick (MVZ190994) composed of woven bone with simple vascular canals and incipient primary osteons. Plane light, 200×. (D) Humerus of a fledgling chick (MVZ190991), composed of fibrolamellar bone and with a developing ICL. Polarized light, 400×. (E) Adult humerus (MVZ190993), showing a thick OCL, middle layer of fibrolamellar bone, and ICL. Plane light, toluidine blue, 400×. In all images the periosteal surface is oriented to top/left and the endosteal to the bottom/right. Scale bare equals 50 µm.

10.7717/peerj.12160/supp-43 Supplemental Information 43 Macroscopic view of house finch growth series, transverse sections of femora. (n = neonate, MVZ190969; p = pin-feathered chick, MVZ190984; pf = pre-fledgling, MVZ190994; f = fledgling, MVZ190991; a = adult, MVZ190993. Cr, cranial; L, Lateral. Scale bar equals 250 µm.

10.7717/peerj.12160/supp-44 Supplemental Information 44 Growth series of house finch femora. (A) Neonate femur (MVZ190969) showing a thick cortex of woven bone with large vascular canals and thin struts of bone separating them. Plane light, 400×. (B) Femur of a pin-feathered chick (MVZ190984) showing features similar to A, but with thicker bony struts separating smaller vascular spaces. Plane light, 200×. (C) Femur of a pre-fledgling chick (MVZ190994) composed of a developing fibrolamellar complex with an incipient OCL. Plane light, toluidine blue stain, 400×. (D) Femur of a pre-fledgling chick (MVZ190967) showing features similar to C; some incipient primary osteons are visible, though some simple vascular canals also remain. Plane light, toluidine blue stain, 400×. (E) Femur of a fledgling chick (MVZ190991) showing a fibrolamellar cortex that is more mature than in the pre-fledgling chicks, but still not finished developing. (F) Adult femur (MVZ190993) showing an avascular region of parallel-fibered bone; other regions of the cortex have woven bone and simple vascular canals. In all images the periosteal surface is oriented to top/left and the endosteal to the bottom/right. Scale bare equals 50 µm.

10.7717/peerj.12160/supp-45 Supplemental Information 45 Macroscopic view of great-horned owl growth series, transverse sections of humeri. p = pin-feathered, MVZ190881; pf = pre-fledgling, MVZ190882; f = fledgling, MVZ190879; a = adult, MVZ190883. Cr, cranial; M, medial. Scale bar equals 1,000 µm.

10.7717/peerj.12160/supp-46 Supplemental Information 46 Growth series of great-horned owl humeri. (A) Humerus of a pin-feathered chick (MVZ190881) showing a thick cortex of woven bone with high vascular porosity and some incipient primary osteons. Plane light, 400×. (B) Humerus of a pre-fledgling chick (MVZ190882). with charactiristics broadly similar to A, but with more matury primary osteons and smaller vascular canals. Plane light, toluidine blue, 400×. (C) Humerus of a fledgling chick (MVZ190879) showing mature fibrolamellar bone in the cortex. Plane light, toluidine blue stain, 400×. (D) Humerus of a fledgling chick (MVZ190879) showing variation in orientation of vascular canals, as well as an incipient OCL and ICL. Plane light, 40×. (E) Adult humerus (MVZ190883) of mature fibrolamellar bone. Plane light, 400×. (F) Adult humerus (MVZ190883) showing moderate level of porosity, strongly-developed ICL, and clear OCL. Plane light, toluidine blue stain, 40×. In all images the periosteal surface is oriented to top/left and the endosteal to the bottom/right. Scale bare equals 100 µm.

10.7717/peerj.12160/supp-47 Supplemental Information 47 Macroscopic view of great-horned owl growth series, transverse sections of femora. p = pin-feathered, MVZ190881; pf = pre-fledgling, MVZ190882; f = fledgling, MVZ190879; a = adult, MVZ190883. Cr, cranial; L, Lateral. Scale bar equals 1,000 µm.

10.7717/peerj.12160/supp-48 Supplemental Information 48 Growth series of great-horned owl femora. (A) Femur of pin-feathered chick (MVZ190881), composed of woven bone large vascular channels that are irregular in shape and size. Plane light, toluidine blue, 400×. (B) Pre-fledgling chick (MVZ190882). Plane light, 400×. (C) Fledgling femur (MVZ190879) composed of mature fibrolamellar bone. Plane light, toluidine blue stain, 400×. (D) Adult femur (MVZ190883) showing a mature fibrolamellar complex; a prominent ICL is also visible. Plane light, 400×. In all images the periosteal surface is oriented to top/left and the endosteal to the bottom/right. Scale bare equals 100 µm.

10.7717/peerj.12160/supp-49 Supplemental Information 49 Macroscopic view of barn owl growth series, transverse sections of humeri. d = downy, MVZ190875; p = pin-feathered chick, MVZ190868; pf = pre-fledgling, MVZ190866, f = fledgling, MVZ190867; sa = sub-adult, MVZ190869; a = adult, MVZ190872. Cr, cranial; L, Lateral. Scale bar equals 500 µm.

10.7717/peerj.12160/supp-50 Supplemental Information 50 Growth series of barn owl humeri. (A) Humerus of a downy chick (MVZ190875), composed of woven bone with large, irregular vascular canals separated by thick struts of bone. Plane light, toluidine blue stain, 200×. (B) Humerus of a pin-feathered chick (MVZ190876) showing disorganized, porous woven bone similar to the downy chick. Plane light, 200×. (C) Humerus of a pin-feathered chick (MVZ190868) with characteristics similar to B, and also showing a region with smaller vascular spaces in the periosteal region of the cortex combined with larger spaces in the endosteal portion. Plane light, toluidine blue stain, 100×. (D) Humerus of a pre-fledgling (MVZ190866) showing developing fibrolamellar bone; there are many incipient primary osteons in this region. Plane light, 200×. (E) Humerus of a pre-fledgling (MVZ190870) composed of incipient fibrolamellar bone. Plane light, toluidine blue stain, 100×. (F) Humerus of a fledgling (MVZ190867), showing a mature fibrolamellar complex in most of the cortical bone, and developing primary osteons with large vascular spaces in the periosteal region of the cortex. (G) Humerus of a sub-adult (MVZ190869) showing a cortex dominated by mature fibrolamellar bone with very small vascular openings; the thin, incipient OCL is also visible. Arrow indicates a structure resembling a growth line, which separates the more organized and less organized regions of the middle cortical layer. Plane light, 200×. Adult humerus (MVZ190872) composed of fibrolamellar bone with a dominant circumferential/anastomosing vascular pattern, between a thin OCL and ICL of parallel-fibered bone. Plane light, 40×. In all images the periosteal surface is oriented to top/left and the endosteal to the bottom/right. Scale bare equals 100 µm.

10.7717/peerj.12160/supp-51 Supplemental Information 51 Macroscopic view of barn owl growth series, transverse sections of femora. d = downy, MVZ190875; p = pin-feathered chick, MVZ190868; pf = pre-fledgling, MVZ190870, f = fledgling, MVZ190867; sa = sub-adult, MVZ190869; a = adult, MVZ190877. Cr, cranial; L, Lateral. Scale bar equals 500 µm.

10.7717/peerj.12160/supp-52 Supplemental Information 52 Growth series of barn owl femora. (A) Femur of a downy chick (MVZ190875) composed of woven bone with large vascular canals; the endosteal two-thirds of the cortex is more mature, with slightly smaller vascular spaces and thicker struts of bone separating them. Plane light, toluidine blue stain 200×. (B) Femur of the pin-feathered chick with a thicker cortex (MVZ190876), showing woven bone a concentration of more mature osteocyte lacunae in the endosteal two-thirds of the cortex. Plane light, 200×. (C) Femur of the pin-feathered chick with the thinner cortex (MVZ190868), composed of woven bone with several incipient primary osteons. Plane light, toluidine blue stain, 100×. (D) Femur of a pre-fledgling (MVZ190866) showing a fibrolamellar complex with larger, simple vascular channels near the periosteal edge. Plane light, 200×. (E) Femur of a pre-fledgling (MVZ190870) showing characteristics similar to D. Plane light, 100×. (F) Femur of a fledgling (MVZ190867) composed of fibrolamellar bone with lower vascular porosity than the pre-fledgling stage, with an incipient OCL of parallel-fibered bone developing along the periosteal margin. Plane light, toluidine blue stain, 200×. (G) Femur of a sub-adult (MVZ190869) showing fibrolamellar bone with many narrow circumferential and anastomosing vascular canals and a distinct OCL. Plane light, 100×. (H) Adult femur (MVZ190877), showing characteristics similar to G but with a mature ICL in addition to an OCL. Plane light, toluidine blue stain, 100×. In all images the periosteal surface is oriented to top/left and the endosteal to the bottom/right. Scale bare equals 100 µm.

10.7717/peerj.12160/supp-53 Supplemental Information 53 Macroscopic view of white-tailed kite growth series, transverse sections of humeri. p = pin-feathered chick, MVZ190862; pf = pre-fledging, MVZ190858; f = fledgling, MVZ190860; a = adult, MVZ190861. Cr, cranial; L, Lateral. Scale bar equals 500 µm.

10.7717/peerj.12160/supp-54 Supplemental Information 54 Growth series of white-tailed kite humeri. (A) Humerus of a pin-feathered chick (MVZ190862) showing a porous cortext of woven bone with a high density of osteocyte lacunae. Plane light, toluidine blue stain, 200×. (B) Humerus of a pre-fledgling chick (MVZ190858) showing a thin region of the cortex, with some incipient primary osteons (toward the endosteal surface) and many large, simple vascular canals. Plane light, 200×. (C) Humerus of a pre-fledling chick (MVZ190864) showing a thinner section of the cortex with a higher concentration of incipient primary osteons and lower vascular porosity. Plane light, toluidine blue stain, 200×. (D) Humerus of a fledgling chick (MVZ190860) showing a thicker region of the cortex still with fairly large vascular channel. Plane light, toluidine blue stain, 200×. (E) Humerus of a fledgling chick (MVZ190859) in a thin region of the cortex; the inner two-thirds are mature fibrolamellar bone with low vascular porosity, while the periosteal region is incipient fibrolamelar with larger vascular spaces. Plane light, 200×. (F) Humerus of a fledgling chick (MVZ191134) with characteristics similar to E. Plane light, 200×. (G) Adult humerus (MVZ190861) showing a region composed of fibrolamellar bone between a distinct OCL and ICL; in other parts of the cortex this middle layer consisted of woven bone with simple vascular canals. Plane light, toluidine blue stain, 200×. In all images the periosteal surface is oriented to top/left and the endosteal to the bottom/right. Scale bare equals 100 µm.

10.7717/peerj.12160/supp-55 Supplemental Information 55 Macroscopic view of white-tailed kite growth series, transverse sections of femora. p = pin-feathered chick, MVZ190862; pf = pre-fledging, MVZ190858; f = fledgling, MVZ190860; a = adult, MVZ190861. Cr, cranial; L, Lateral. Scale bar equals 500 µm.

10.7717/peerj.12160/supp-56 Supplemental Information 56 Growth series of white-tailed kite femora. (A) Femur of a pin-feathered chick (MVZ190862) showing woven bone with high vascular porosity and a high density of osteocyte lacunae. Plane light, 200×. (B) Femur of a pre-fledgling chick (MVZ190858) composed of incipient fibrolamellar bone with smaller vascular openings than the pin-feathered stage. Plane light, toluidine blue stain, 200×. (C) Femur of a pre-fledgling chick (MVZ190864) with characteristics similar to B. Plane light, 200×. (D) Femur of a fledgling chick (MVZ190860) with an irregular endosteal border and thin periosteal layer of woven bone on either side of fibrolamellar bone. Plane light, toluidine blue stain, 200×. (E) Femur of a fledgling chick (MVZ190859) composed predominantly of fibrolamellar bone, with vascular porosity highly reduced relative to the pre-fledgling stage. Plane light, toluidine blue stain, 200×. (F) Femur of a fledgling chick (MVZ191134) showing characteristics similar to D and E. Plane light, toluidine blue stain, 200×. (G) Adult femur (MVZ190861) showing fibrolamellar bone between a prominent OCL and a thick ICL. Plane light, 200×. In all images the periosteal surface is oriented to top/left and the endosteal to the bottom/right. Scale bare equals 100 µm.

10.7717/peerj.12160/supp-57 Supplemental Information 57 Macroscopic view of red-tailed hawk growth series, transverse sections of humeri. d = downy, MVZ190852; pf = pre-fledgling, MVZ190853; a = adult, MVZ190855. Cau, caudal; M, medial. Scale bar equals 500 µm.

10.7717/peerj.12160/supp-58 Supplemental Information 58 Growth series of red-tailed hawk humeri. (A) Humerus of a downy chick (MVZ190852) composed of woven bone with large, irregularly shaped vascular channels. Plane light, 200×. (B) Humerus of a pre-fledgling chick (MVZ190853) with characteristics similar to A, but thicker struts of bone separate vascular channels (which, as a consequence, are smaller in comparison). Plane light, 200×. (C) Adult humerus (MVZ190855) showing the thick middle layer of fibrolamellar bone between and OCL and ICL that characterizes the adult red-tailed hawk. Plane light, toluidine blue stain, 200×. (D) Adult humerus (MVZ190854) showing a close-up on a region of fibrolamellar bone with secondary osteons. Plane light, 200×. In all images the periosteal surface is oriented to top/left and the endosteal to the bottom/right. Scale bare equals 50 µm.

10.7717/peerj.12160/supp-59 Supplemental Information 59 Macroscopic view of red-tailed hawk growth series, transverse sections of femora. d = downy, MVZ190852; pf = pre-fledgling, MVZ190853; a = adult, MVZ190854. Scale bar equals 500 µm.

10.7717/peerj.12160/supp-60 Supplemental Information 60 Growth series of red-tailed hawk femora. (A) Femur of a downy chick (MVZ190852) showing woven bone with large vascular channels and a high density of osteocyte lacunae. Plane light, 200×. (B) Femur of a pre-fledgling chick (MVZ190853) with a cortex of woven bone in which vascular canals are smaller than the downy stage; here they are seen oriented in many directions. Plane light, 200×. (C) Adult femur (MVZ190855) showing fibrolamellar bone between a thin OCL and a thicker, more fully developed ICL. Plane light, 200×. (D) Adult femur (MVZ190854) showing characteristics similar to C. Plane light, 200×. In all images the periosteal surface is oriented to top/left and the endosteal to the bottom/right. Scale bare equals 50 µm.

## Additional Information and Declarations

### Data Availability

The following information was supplied regarding data availability: Raw data (measurements of histological structures) are available in the Supplemental Tables.
