# Supplementary material for: A histological survey of avian post-natal skeletal ontogeny
Source: PeerJ. 2021 Oct 1;9:e12160. doi: 10.7717/peerj.12160 (PMC8489414; doi:10.7717/peerj.12160)
Supplement: Supplemental Information 1 [file peerj-09-12160-s001.docx]

**Supplemental Material**

*Struthio camelus* - Ostrich (Paleognathae)

Ostriches for this study were received as donations from an ostrich farm. Because accumulation of specimens was opportunistic, and mature birds live long lives, no adult or subadult individuals were obtained for the study. The youngest chick obtained is estimated to be approximately two weeks old. Specific ages for other specimens are unknown, though it is estimated that the sample presented here represents the first several months of growth. These specimens form a continuum of increasing cortical diameter and bone maturity, which we interpret as representative of increasingly older individuals. Specimens are discussed here from smallest and most immature, to largest and most mature.

The humerus of the flightless ostrich is a thin, dense rod of bone, very much unlike that of the other birds in terms of function and therefore structure. This element is circular in cross-section in all individuals, and has a very thick cortex relative to total cross-sectional diameter persisting into older developmental stages (Supplementary Fig. 1; Supplementary Table 1). Through the first few months of ontogeny at least, the ostrich humerus exhibits a steady trend of thickening cortical bone, both in ACT and RCT; during these stages, it is inferred that periosteal deposition is proceeding more quickly than endosteal resorption. This increase in RCT of the humerus of the ostrich is in contrast to the study of Castanet et al. (2000) who report that growth of these elements seemed to be characterized by a balance between periosteal bone deposition and endosteal bone resorption, thus resulting in a relatively constant cortical thickness. It is possible this discrepancy highlights variation in ostrich bone growth, or environmental differences (ostriches used in this study were donated from a farm that raises the birds as economically as possible; ostriches for the other study were from a reserve where birds are raised and bred in conditions presumably closer to what is experienced in the wild). However, it is notable that an early increase in ACT was also observed in nearly all other taxa in this study.

The humerus of the two-week-old ostrich is characterized by a thick cortical wall of mostly woven bone, perforated by a dense network of longitudinally-oriented vascular canals with a low amount of anastomoses visible (Supplementary Fig. 2, A); this is consistent with results reported by Castanet et al. (2000) in both ostrich and emu chicks. Canals toward the endosteal surface of the bone are relatively larger and more irregularly shaped; canals toward the periosteal surface have begun bone in-filling, representing early stages of osteon formation, and are more circular in shape. The cortical bone is initially of near-uniform thickness but becomes slightly thinner in the medial and dorsolateral regions with age and forms a particularly wide region along the craniomedial border (Supplementary Fig. 1). A thin lattice of endosteal bone is visible in some hatchlings but is quickly resorbed, and replaced by a thick, irregular layer of endosteum lining the endosteal cortex and occasionally extending into the cavity in large patches. An abundance of osteocytes are present in the neonate humerus.

For a time after about two weeks of post-natal growth, a number of oblique vascular canals appear in the periosteal half of the cortex, with a network of numerous longitudinal canals and incipient osteons in the endosteal half. The number of osteocyte lacunae with distinct canaliculi also appears to increase through at least portion of the growth trajectory documented in this study (Supplementary Fig. 2). Another interesting feature of the oldest chick examined here is the presence of parallel-fibered bone in a thin layer forming around the endosteal edge of the craniolateral cortex (Supplementary Fig. 2, F). The endosteal edge of the cortex retains a somewhat wavy appearance, characteristic of immature individuals, but this may represent the early stages of formation of the ICL. Longitudinal vascular canals fill in with lamellar tissue to form close-packed osteons present through most of the cortex by about two months. Therefore, by this stage of growth the porosity of the bone has decreased substantially as it transforms from woven to fibrolamellar.

The pelvic limb is the dominant locomotor module in the ostrich, and the femur is a large, robust element adapted for weight-bearing and locomotion, in contrast to the relatively minimalistic humerus. Also unlike the humerus, the two-week-old cross-sectional shape already bears a close resemblance to the rounded-triangle cross-sectional shape of more mature individuals (Figure 2; Supplementary Fig. 3). As precocial birds, chicks locomote relatively soon after hatching, so this asymmetrical shape may reflect the early weight-bearing and kinetic demands on this element. In contrast to the trend of increasing RCT through ontogeny observed in the humerus, RTC of the femur remains relatively constant across the growth stages represented here.

In the youngest chick (about two weeks old), the femoral cortex is composed of woven bone with a high level of porosity (Supplementary Fig. 4, A). There are numerous large canals, but most no longer communicate with the periosteal edge, forming a relatively smooth edge for such a young individual. This is also indicative of the greater maturity of this bone at hatching relative to the humerus. Many channels are oriented circumferentially, but longitudinal and oblique canals are not uncommon. Several large, irregular erosional cavities are present near the endosteal margin of the cortex. Cortical thickness across the diameter is also relatively even in the hatchling chick.

Through early growth, the anastomosing network of vessel cavities remains elaborate, but the spaces become increasingly infilled with parallel-fibered bone, reducing porosity within the stages represented here (Supplementary Fig. 4, B&C). As in the humerus, vascular canals of the periosteal half of the cortex are becoming smaller as incipient osteons form, while the endosteal half retains a less mature appearance with large, irregular canals.

Older chicks have broadly similar histological characteristics: a thickening cortex with bone maturing into a fibrolamellar complex as primary osteons form within the woven matrix (Supplementary Fig. 4, D-F). Rates of bone deposition appear to be higher along the craniomedial margin, which exhibits the greatest cortical thickening. The density of osteocyte lacunae also greatly increases through at least the first several months of growth. A unique characteristic of the oldest chick in this study is an emerging pattern of predominantly longitudinal canals in the more endosteal half of the cortex, and predominantly circumferential canals in the more periosteal half (Supplementary Fig. 4, F).

*Callipepla californica* - California Quail (Galliformes)

Predominantly neonate quail chicks and quail adults were received for this study (from the Lindsay Wildlife Hospital), with very few individuals of intermediate maturity. The following results therefore focus on differences between hatchling and mature adult.

In cross-section, the neonate humerus is roughly elliptical with a flattened lateral edge and rounded medial, cranial, and caudal margins (Supplementary Fig. 5). In many of the chicks sectioned, bone deposition was asymmetrical resulting in a thick caudomedial margin and thin lateral cortex. The bone has a general spongy appearance, with the predominance of vessel cavities in the thick caudomedial cortex and relatively few in the thinner cortical regions, particularly in the cranial cortex (Supplementary Figs. 5&6, A-C). These areas are instead dominated by a high density of osteocytes. All observed vascular cavities are longitudinally oriented. In most individuals, a thick endosteum variably lines the medullary cavity, sometimes with thin, soft-tissue projections extending into the space; in some, thick portions of periosteum were also preserved. The three individuals identified as neonates based on gross anatomy exhibit some variation in cortical thickness (Supplementary Fig. 6, A-C); this is either evidence of the effect of environmental variables on growth (e.g., availability of food), different growth phases not discernable based on external morphology, or individual variation in development. Overall, while the bone is clearly of immature individuals, the relatively thick cortex, smooth endosteal and periosteal edges, and dense population of osteocytes, and small vessel canals indicate the bone is surprisingly mature for neonate individuals. Like the ostrich, the quail is a precocial bird with chicks highly motile soon after hatching; it is likely that this relative maturity of the bone is related to developmental mode.

The bone of the pin-feathered chick is very similar to that of the neonates (Supplementary Fig. 6, D), composed of woven bone with numerous large vascular channels. However, many of these channels are beginning to become encircled by organized osteocyte lacunae, and thus represent incipient osteons.

The adult humerus is much more circular in cross-section, with a cortex of generally more uniform thickness (Supplementary Fig. 6, E&F), though RCT within range of that observed in earlier growth stages (Supplementary Table 1). A clear ICL of parallel-fibered bone with minimal vascularization is present (Supplementary Figs. 5&6, E&F). A thick layer of avascular parallel-fibered bone comprises the outer 1/3 of the cortex, forming an OCL. The presence of this structure indicates that diametric growth has slowed substantially. The middle cortical layer has a woven matrix with a moderate amount of longitudinally oriented vascular canals visible (Supplementary Fig. 6); some show signs of developing into primary osteons, but these regions are at most incipiently fibrolamellar. Porosity decreased substantially over post-natal growth. While most canals are oriented longitudinally, sparse oblique canals, connecting the central canals of some osteons, are present. Primary osteons are concentrated mainly in the middle of the cortex and are weakly formed when present (Supplementary Fig. 6, D&E) with few concentric layers of osteocyte lacunae and lamellar bone not distinct. There is also a very high density of osteocyte lacunae throughout most of the cortex.

The femur of the neonate is spongy, and is composed of woven bone with numerous relatively large vascular canals. The overall appearance is similar to the humerus although the pelvic element is circular (as opposed to elliptical) in cross-sectional shape, with a uniform thickness and even distribution of vascular channels (Supplementary Figs. 7&8, A-C). Like the humerus, femora of several chicks identified as neonates based on gross anatomy exhibit a surprising range of histological maturity, varying in thickness of cortical bone and maturity and organization of vascular canals (Supplementary Fig. 8, A-C). Again, this may reflect environmental differences (e.g., availability of food), differences in individual developmental trajectories, or may suggest that chicks in the “neonate” age class vary in actual numerical age more than could be inferred from body size and state of feather development. Most of the canals are longitudinally oriented, though several circumferential channels are present. The shape and orientation of the canals is generally non-uniform. They range from spherical to elliptical to oblate to generally irregular. In orientation, many are longitudinal but others are circumferential or oblique; however, at this stage of growth there is almost no anastomosing of vessels. Also as in the humerus, a substantial endosteum is present in many individuals, often forming thin projections of soft tissue that extend into the medullary cavity.

As in the humerus, femur of the pin-feathered chick is very similar to that of the neonates (Supplementary Fig. 8, D), composed of woven bone with numerous large vascular channels. It shows increased signs of maturity in the form of incipient osteons.

The adult femur strongly resembles the adult humerus in general shape and histological characteristics, but with a higher ACT (Supplementary Figs. 6&7). However, as in the humerus, RCT is very similar between adults and neonates. This uniform rate of endosteal resorption and periosteal deposition is unusual among birds (see “Results” for other taxa in this dataset), and is potentially related to the precocial life history of the quail. However, it is impossible to comment with certainty, as all intermediate growth stages are not available for this study. In other taxa the pre-fledgling and fledgling chicks are often those with the thickest cortical walls, both stages that are missing from this growth series.

In the adult quail, the femur is nearly perfectly circular in cross-section, with a uniform cortical thickness. The ICL is thin and prominently formed of parallel-fibered bone bordering on lamellar. The OCL is much thicker, formed of parallel-fibered bone and occupying nearly half of the cortex. This region is also nearly avascular. As in the humerus, the OCL appears to be slightly more ‘mature’ in one individual than in the other. However, the femur MVZ190749 has a more-developed OCL than MVZ190762 (opposite the relationship seen in the humerus). Between the OCL and ICL is a region composed varyingly of weakly-formed fibrolamellar bone, and woven matrix with simple vascular canals (Supplementary Fig. 8 E&F), similar to the humerus. Most canals are oriented longitudinally, with a moderate degree of anastomoses between them formed by obliquely-oriented Volkmann’s canals. No trabeculae are present, and the bulky endosteum of chicks has been highly reduced.

*Meleagris gallopavo* - Wild Turkey (Galliformes)

Three wild turkeys were included in this study (all donated by the Lindsay Wildlife Hospital): one adult female, one pre-fledgling male chick estimated to be 4-6 weeks old, and one male neonate. In cross-section, the humerus of the wild turkey has the shape of a rounded triangle throughout post-natal growth, with a semi-flattened cranial margin and a relatively pointed medial margin (Supplementary Fig. 9); however, this shape becomes more subtle in the adult due to differential bone deposition that leads to a general rounding out of the cortex. RCT steadily decreases through ontogeny after hatching. ACT increases through ontogeny.

The neonate humerus is composed of woven bone with numerous large vascular canals (Supplementary Figure 10, A). Cortical thickness is very uneven, with the medial portion much thicker than the lateral (Supplementary Fig. 9). The distribution of vascular canals is similarly asymmetrical, with a much higher number present along the mediocranial, medial, and mediocaudal parts of the cortex. These canals are large and irregularly shaped, and overall the element has a high level of porosity. Many channels are oriented longitudinally, but circumferential and oblique canals are also observed (Supplementary Fig. 10, A). A moderate amount of relatively large, non-uniformly shaped osteocyte lacunae are evenly distributed throughout the matrix. A thick, variably present endosteum is visible in the medullary cavity, with extensions of soft tissue into the medullary cavity.

The humerus of the 4-6 week old chick is characterized primarily by woven bone that is transitioning to a fibrolamellar complex (Supplementary Fig. 10, B). The cortex is becoming relatively thinner and more even, and the lateral and medial margins are now equal in bone mass (Supplementary Fig. 9). Much of the vascular space has been infilled, and channels generally are smaller. Many longitudinally-oriented canals are present and represent early stages of primary osteon formation with osteocytes circularly arranged around them, though distinct lamellae are not yet present and the central canals remain larger than in mature osteons. This dense network of longitudinal canals and incipient osteons characterizes the whole of the bone cortex in the older chick. Along some portions of the periosteal margin, particularly the cranial edge, simple longitudinal canals are also present. Osteocytes are of a higher density throughout the matrix than in the neonate, and while still relatively large, overall have a more uniformly elliptical shape.

The adult humerus is a very large, robust element. By this stage of growth, the cortical wall is of nearly uniform thickness, with a slight thickening at the rounded point formed along the medial margin. The adult turkey humerus is further characterized by both an ICL and OCL. The former is generally more easily distinguishable, though both very clearly form layers of parallel-fibered bone with very few osteocyte lacunae along the endosteal and periosteal edges of the cortex (Supplementary Fig. 10, C&D). Overall, this bone is primarily fibrolamellar with a laminar vascular network (most canals are oriented circumferentially); in some regions, there are connections between these circumferential vessels, forming a network of plexiform canals (Supplementary Fig. 10, C). However, the medial and caudomedial regions of the cortex are dominated instead by longitudinal canals and osteons, both primary and secondary. This area also shows a nutrient foramen perforating the medial surface of the mid-shaft of the humerus (Supplementary Fig. 10, E). In the next serial thin section and in the same position along the diameter of the cortex, there is a loop of parallel-fibered bone that extends into the medullary cavity, interpreted as a large neurovascular channel continuous with the nutrient foramen (Supplementary Fig. 9). Finally, there is a high density of thin, small osteocyte lacunae throughout the adult humerus.

Unlike the humerus, the femur generally increases in RCT during post-natal growth, but only moderately (Supplementary Fig. 11; Supplementary Table 1). The cross-sectional shape of this element is circular in the neonate and 4-6 week old chick, and circular with a slight craniocaudal compression in the adult. In the neonate there is a delicate mass of endosteum, with projections that interweave in the medullary cavity (Supplementary Fig. 12, A).

The femur of the neonate is a thin-walled element composed of woven bone with vascular canals that are large enough to signal immaturity, but smaller than those observed in the neonate humerus (Supplementary Figs. 10, A & 12, A). Many of these longitudinally-oriented canals already seem to be developing into primary osteons. Several circumferential Volkmann’s canals are also present. In general, the canals are larger and more irregularly shaped closer to the endosteum. Already there is a dense population of osteocyte lacunae present, although at this early stage of development they remain relatively large and ‘plump’ (Supplementary Fig. 12, A).

In the 4-6 week old pre-fledgling chick femur, large pieces of endosteum are visibly peeling away from the endosteal cortex. Fibrolamellar bone is well under development within the cortex. An abundance of incipient osteons and mature osteons is present throughout the cortical matrix (Supplementary Fig. 12, B). Most of these are oriented longitudinally, though numerous oblique Volkmann’s canals give the bone a moderately reticulated appearance in some regions of the cortex. Osteocyte lacunae with fully-developed canalicular systems appear even more abundant than in the neonate, and are relatively smaller in size and more compressed in shape, though they remain relatively ‘plump’ compared to lacunae in the adult bone.

The adult femur is also composed of fibrolamellar bone, but with a predominance of circumferentially-oriented vessels rather than longitudinal (Supplementary Fig. 12, D). The two regional exceptions are localized areas on the cranial and caudal margins where slight, narrow, raised areas represent the formation of inter-muscular lines down the shaft of the femur. Here, the pattern of vascularity is much better described as plexiform rather than laminar (though even in the predominantly laminar regions of the tissue anastomoses of vessels and the presence of longitudinal osteons break up the pattern). Primary osteons, and even some secondary osteons, are prevalent in the femoral cortex (Supplementary Fig. 12, C). As in the humerus, an ICL is easily distinguishable, and a clear OCL is also present (although the bone of this layer is less distinct and well-organized than that of the ICL). A spire of parallel-fibered bone continuous with the ICL protrudes into the medullary cavity at the craniolateral cortex. The medullary cavity of the femur is further characterized by a thick infilling of bone and residual red marrow (Supplementary Fig. 12, D&E). This tissue appears to comprise medullary bone in the femur, implying that this female was at some stage of the egg-laying period at the time she died.

*Calypte anna* - Anna’s Hummingbird (Trochilidae)

The histological analysis of hummingbird ontogeny presented here is limited in scope relative to that for other taxa. Most individuals collected represent older developmental stages (fledgling chick to adult with one pin-feathered chick). One neonate was obtained, but the specimen was so small (~1cm total body length), and the long bones so minimally ossified, that histological sectioning of the pectoral and pelvic limb was impractical using the methods applied in this study. All hummingbird specimens were received from the Lindsay Wildlife Museum.

The humerus of the hummingbird has a unique morphology unlike that of any other bird included in this study, because it is specially adapted to hovering. Rather than having an elongate, rounded shaft with most bony landmarks (e.g., attachment points for muscles and tendons) at the proximal and distal ends, the humerus of the hummingbird is extremely short, flattened, and wide with the deltopectoral crest, bicipital crest, and other bony landmarks extending nearly the full length of the very short element. The shape in cross section therefore deviates greatly from the circular and elliptical humeri of other taxa (Supplementary Fig. 13). While there is a main medullary cavity, generally rounded caudally and flat cranially, the diaphysis expands medially and laterally into two narrow projections also enclosing medullary space. The lateral projection traces the development of the deltopectoral crest, and the medial projection that of the bicipital crest. This general shape is present as early as the pin-feathered stage of development, though the lateral and medial projections are much smaller and the medial is not yet separated from the main medullary cavity (Supplementary Fig. 13).

The cortical wall of the pin-feathered chick humerus is a very thin layer of woven bone with numerous osteocyte lacunae (Supplementary Fig. 14, A). Unlike many other very young chicks, the mid-shaft of the hummingbird chick humerus has very few vascular perforations. Most that are present are not fully enclosed within the cortex, instead meeting with the endosteal edge of the developing bone. The elaborate pneumatic cavities seen in older individuals have not yet developed in the medial and lateral projections, both of which are relatively reduced (Supplementary Fig. 13).

The humerus of the fledgling chick has a thin cortical wall mostly composed primarily of parallel-fibered bone, with a layer of woven bone and patches of chondroid bone within the more endosteal portion of the cortex. (Supplementary Fig. 14, B). This bone, too, has no vascular canals proper, but the scalloped shape of the endosteal margin indicates the presence of a number of vascular canals within this space. In this individual, osteocyte lacunae are particularly concentrated along the areas adjacent to the medullary cavities, especially at the ‘corners’ of the lateral and medial projections and along the bars of bone separating off the accessory medullary spaces in these projections from the main medullary space. An ICL and OCL are present in subadult and adult birds. The ICL appears as a thin irregular layer and as though it is the early stages of developing. The OCL also varies in width, but in some is so thick that the parallel-fibered bone of which it consists dominates most of the cortex, particularly along the lateral margin.

Subadult individuals maintain a similar pattern of an outer layer of parallel-fibered bone forming an OCL, and an inner layer of chondroid bone (Supplementary Fig. 14, C-E). However, the former shows increased signs of maturity: it is thinner, with flattened osteocyte lacunae generally oriented in the same direction. Near the periosteal margin, the bone is nearly acellular, populated by very few lacunae. This is the dominant layer in the cortex of the main diaphysis, with a thin-to-nonexistent woven bone layer. However, the developing deltopectoral and bicipital crests have a very prominent, thick layer of chondroid bone (Supplementary Fig. 14, C). In one individual there was a small pocket of fibrolamellar bone consisting of a single primary osteon in a woven matrix. In these regions, there are many osteocyte lacunae. Here, they are large, rounded in shape, and irregular in orientation. This element remains nearly avascular, with sparsely distributed simple, longitudinal vascular canals.

The adult humerus is composed almost entirely of parallel-fibered bone (Supplementary Fig. 14, F). The degree of organization of the bone parallels patterns observed at the subadult stage. In the cortex of the diaphysis, osteocyte lacunae are very thin and flattened, all in a similar orientation. In the bone of the bicipital crest and deltopectoral crest, and the bony struts between pneumatic spaces, lacunae are mature but appear plump by comparison. Their orientation is also slightly more irregular. Sparse, small patches of woven matrix remain in the areas of thicker bone in the bicipital and deltopectoral crests. A thin, irregular ICL is present. It is difficult to discern the OCL as a distinct layer, as almost the entire cortex now has the same degree of organization and is mostly composed of parallel-fibered bone. It seems that the OCL is so thick as to dominate the cortex of the adult.

The femur of Anna’s hummingbird, on the other hand, follows a more typical growth pattern. Throughout post-natal ontogeny, it is circular in cross-sectional shape, and steadily maintains a cortex of uniform thickness along all margins (Supplementary Fig. 15). Both ACT and RCT increase only moderately through growth of the stages represented here.

The femur of the pin-feathered chick has an extremely thin cortex composed of woven bone (Supplementary Fig. 16, A). There are very few, tiny vascular canals and a single very large canal located at the endosteal margin of the cortex (visible in Supplementary Fig. 15); all are oriented longitudinally. By the fledgling stage, the femur has grown to adult thickness. The bone is mostly parallel-fibered with sparse osteocyte lacunae. This layer is lined endosteally by a thin and variably-present layer of chondroid bone. Lacunae generally become smaller and take on the more flattened appearance of mature osteocyte lacunae. Under polarized light, in the caudal part of the cortex the endosteal margin of the parallel-fibered layer has the appearance of an even greater degree of organization, with bands of collagen fibers arranged in parallel. Vascular canals are even more rare than in the younger individual.

Subadult individuals are much the same, but with even fewer osteocyte lacunae (Supplementary Fig. 16, C-E). Additionally, by this time in development bony landmarks such as an inter-muscular line (likely the linea internus cranialis) are clearly formed (Supplementary Fig. 15). A definite ICL is also present in one individual, but not all (Supplementary Fig. 16, C-E), indicating that not all individuals in this morphologically-defined age class have achieved the same level of skeletal maturity. This ICL is of very thin, and variable in width throughout the endosteal circumference, appearing to be in early stages of formation.

In the adult hummingbird, the cortex is formed entirely of parallel-fibered bone. Small, elliptical osteocyte lacunae are widely dispersed and have well-developed canaliculi. An ICL is present, but no OCL is apparent. This feature is variable in appearance around the circumference of the diaphysis, alternately appearing very clearly and at times very weakly developed even under polarized light. It bears a resemblance to the incipient ICL observed in one subadult , and does not appear to be substantially more mature. Once again, endosteum clearly lines the medullary cavity, but no projections were observed in the sections examined. Notably, a relatively thick endosteal lining is present even in the adult individual. This bone is avascular in the sections studied here.

*Zenaida macroura* - Mourning Dove (Columbiformes)

The mourning dove growth series received from the Lindsay Wildlife Hospital is complete (in terms of the growth stage break-down used in this study), with every stage represented from neonate to adult. However, the neonate chick was not described here due to its extremely small size and unossified limb bones. The following description therefore pertains to the pin-feathered through adult stages.

The humerus of pin-feathered chicks is elliptical in cross-section, with the mediolateral axis longer than the craniocaudal (Supplementary Fig. 17). The cranial region of the cortex is thinnest, and a notable thickening occurs along the lateral and medial curves leading to a widest point along the caudal portion. This bone is highly porous and spongy. Most vascular canals are oriented longitudinally, though varying greatly in size and shape, forming almost a honey-comb of bone (Supplementary Fig. 18, A). Osteocyte lacunae are common, and similarly variable in size and shape.

In the pre-fledgling chicks, ACT of the humerus increases, while the porosity decreases moderately, though the bone retains a strongly spongy appearance (Supplementary Fig. 18, B&C). Many vascular canals are longitudinal, but size, shape and orientation remain highly irregular at this stage of growth. A thick endosteum lines the medullary cavity of these individuals. Osteocyte density has increased relative to the pin-feathered chick, and lacunae are predominantly large and rounded.

ACT and RCT remain fairly constant as chicks transition into the fledgling stage, and becomes nearly uniform in all sub-regions of the cortex (Supplementary Fig. 17). The thick endosteum seen in the pre-fledglings persists. At this stage of growth, much in-filling of vascular channels has occurred, forming an incipient fibrolamellar complex with a reticular, highly-variable network of canals and developing primary osteons (Supplementary Fig. 18, D&E). Though porosity is considerably reduced since hatching, vascular canals are still relatively large. Osteocyte lacunae also retain the relatively larger, more circular morphology.

At the subadult stage, adult RCT of the humerus is achieved, indicating a moderate decrease from observed values in fledgling birds (Supplementary Fig. 17). A substantial endosteum is present at this growth stage, but is reduced relative to that observed in younger birds (Supplementary Fig. 18, G). In both the subadult bird and the adult, cortical width across each element remains uniform, and humeral cross-section is still oval, with a longer mediolateral than craniocaudal axis, and the cranial margin has become subtly flattened. In both, osteocyte lacunae are very numerous, and have acquired the reduced size and flattened shape of more mature individuals. Overall, the appearance is very similar to the adult humerus (Supplementary Figs. 17 & 18, F-H). However, vascular canals are still very numerous, if mostly reduced in size. Some primary osteons are well developed, while others are still incipient. Most channels are longitudinally oriented, with several wide Volkmann’s canals present. Both subadults have an incipient OCL of parallel-fibered bone with very few vascular canals; no evidence of an ICL is apparent, though one specimen classified in this group has a thinner incipient OCL and retains scalloped periosteal and endosteal margins, thus appearing more immature (18, F). This is an important reminder that development is a spectrum, and the discretization applied in this study is ultimately based on subjective categories.

In contrast to the subadults, the adult has relatively few canals in the cortex, and nearly all are present as the small central canals of longitudinally-oriented primary osteons embedded in a weakly woven matrix. An OCL is present as a thick layer of avascular, parallel-fibered bone, occupying anywhere from one-third up to one-half of total cortical thickness (Fig. 18, H). An ICL is not readily apparent, but photographs of the adult specimen under polarized light show an organized, circular pattern of collagen fiber orientation at the endosteal edge, indicating the ICL may either have begun formation or is less distinctly developed in this taxon. Toluidine blue staining also reveals a resorption line along the margin of this incipient ICL where it meets the middle cortical layer.

The femur of the mourning dove undergoes a more uniform change in RCT, decreasing consistently from pin-feathered chick to adult. ACT, in contrast, appears to remain about the same across age classes. Additionally, the femur retains a circular cross-sectional shape through nearly all stages of post-natal ontogeny, with the exception of some mediolateral compression observed in several pre-fledgling chicks (Supplementary Fig.19).

In the pin-feathered chick, the femur is composed of a thick layer of spongy bone. Vascular channels are arranged longitudinally, and vary greatly in size and shape (Supplementary Fig. 20, A). As in the humerus, osteocyte lacunae are present in moderate amounts and also exhibit great irregularity in size and shape. The femur of the pre-fledgling chick is overall similar in appearance, but with a slightly lower RCT. Also, bony struts between vascular channels are thicker, resulting in a relative decrease in porosity and size of the channels (Supplementary Fig. 20, B&C). Osteocyte lacunae are generally ‘plump’ and relatively large. A thick endosteum with some extensions into the medullary cavity is also present in the femur at this phase, as in the humerus.

This layer of endosteum persists in fledgling doves, though substantial changes occur in the cortex. The femur is now predominantly composed of regions of fibrolamellar bone and incipient fibrolamellar bone (with developing primary osteons), with a high frequency of longitudinal vascular canals (Supplementary Fig. 20, D&E). Many of these are incipient osteons, while others are still at earlier stages of development as growing bone subdivides what were larger spaces. Though porosity is clearly decreasing, it remains relatively high in the femora of fledgling birds. Also as in the humerus, osteocyte lacunae are still large and circular.

At a macro-scale, the subadult and adult femora are very similar in terms of cortical thickness and shape (Supplementary Fig. 19). However, a closer look at the microanatomy reveals substantial differences (Supplementary Fig. 20, F-H). In the subadults, a low number of vascular canals are present, many oriented longitudinally but some are also circumferential or oblique. Some are still primary osteons, but appear very weakly-formed. Some simple vascular canals are also present. The matrix of the cortex is still mostly woven, but a thin, periosteal layer of parallel-fibered bone is present in one individual, possibly representing the beginnings of OCL formation (Supplementary Fig. 20, G). The femora, like the humeri, bear evidence of a difference in bone maturity between the two individuals classified as “immature,” highlighting developmental variability.

The cortex of the adult is dominated by osteocyte lacunae with exceedingly sparse vasculature. Under polarized light, an ICL is clearly visible. An OCL appears to dominate the cortex, as most of the bone is formed of parallel-fibered tissue. (Supplementary Fig. 20, H). Several small regions of weakly woven matrix with sparse simple vascular canals are present. In life, while this bone was highly populated with osteocytes, it was also nearly avascular (at least at along the mid-shaft). The few channels that are present are longitudinally oriented simple canals. The osteocyte lacunae have become small and compressed.

*Larus occidentalis* - Western Gull (Charadriiformes)

Only neonate chicks and adult gulls were available for this study; adults came from the SPCA of Monterey County, while the chicks had been donated to the MVZ from an unknown source. The following comparisons will thus be restricted to these two end points in the post-natal growth series. The gull humerus and femur are circular in cross-section in both age classes (Supplementary Figs. 21&23). RCT of the humerus and femur falls in the same range for adults and neonates, though ACT of both elements is much higher in adults. The humerus of adult gulls had the highest RCT of all taxa in this study.

The humerus of the neonate is made up of spongy bone with numerous large vascular cavities distributed evenly throughout the cortex (Supplementary Fig. 22, A&B). The predominant orientation of the vascular canals is longitudinal. Because they are still in early stages of development, the shape of these channels is very roughly circular with much irregularity. Vascular channels along the periosteal perimeter tend to be slightly smaller and more oblong in shape, perhaps in the process of forming circumferentially-oriented canals. Both the periosteal and endosteal edges of the cortex have the characteristic scalloped appearance of immature bone, where blood vessels were in contact with the cortex but not fully-enclosed in bone. Osteocyte lacunae have a relatively sparse distribution in the gull chick humeri. Lacunae that are present are relatively small and lack well-developed canaliculi. A thick peri- and endosteum were present in both chicks included in the study.

In the adult humerus, the cortex is formed almost exclusively by fibrolamellar bone with an anastomosing, with reticular and longitudinal primary osteons, except for parallel-fibered bone present in the ICL and OCL (Supplementary Fig. 22, C&D). The ICL is the much thicker, more prominent layer of the two. Both layers are very clearly composed of lamellar bone. The presence of both these layers signals a substantial slowing in rates of periosteal growth and endosteal resorption. The ICL of the second adult branches to form a short trabecula or bony bridge along the medial endosteal surface, not unlike the bony loop and rod seen in the medullary cavity of the turkey humerus and femur, and probably enclosed the structures perforating a nutrient foramen to reach the medullary cavity. Vascular canals are small, but numerous. The density of primary osteons is low, and several secondary osteons were observed near the ICL (Supplementary Fig. 22, C). In contrast, osteocyte lacuna density is very high; small, elliptical lacunae are present throughout the cortex, with a highly elaborate and well-developed system of canaliculi. Both specimens are female, and one individual preserves small remnants of delicate medullary bone lining medullary cavity in uneven patches (Supplementary Fig. 22, D).

Histologically, the femur of the gull neonate is nearly indistinguishable from the humerus, but with a higher ACT, indicative of a slightly greater maturity (Supplementary Fig. 23). The spongy bone of the cortex has many large, longitudinally-oriented vascular canals. Not uncommon are circumferentially-oriented canals (Supplementary Fig. 24, A&B); often the longitudinal channels anastomose with one another to form a circumferential channel. Once again, the density of osteocyte lacunae is low. The peri- and endosteal edges of the cortex are rough and wavy, and the shape of the vascular channels is non-uniform and highly variable.

The adult femur is very similar to the humerus in terms of histological and macroscopic features. The cross-sectional shape of both is circular, though the cortex of the femur is marginally smaller as compared to the endosteal diameter in the humerus. The cortex is composed of fibrolamellar bone dominated by a reticular vascular network. Secondary osteons are present, as in the humerus, but in higher numbers than in the pectoral element, demonstrating that the femur underwent more remodeling at the midshaft. Osteocyte lacuna density is high, and these structures are elliptical and oblate in shape. A layer of parallel-fibered bone was deposited on the end- and periosteal edges, forming an OCL and an ICL. The ICL is very distinct, and the OCL is only faintly visible. Interestingly, numerous branches of the Volkmann’s canals extend down into the ICL.

Tissue is present in the medullary cavities of both adult gull femora, both of which were identified as female based on the presence of ovaries. One contains broad patches of marrow (this individual also had marrow in the humerus). The other contains a moderate amount of fine medullary bone (this individual also has traces of medullary bone in the humerus) (Supplementary Fig. 24, E); this histological information suggests that this individual died at a stage of its reproductive cycle close to egg-laying.

*Falco sparverius* - American Kestrel (Falconiformes)

An abundance of kestrel specimens was not available to this study; the few that were received came from the Lindsay Wildlife Museum. The following comparisons are primarily between neonates and adults, with one representative of an intermediate growth stage.

Based on the growth stages represented, the RCT humerus of the American kestrel remains remarkably constant (Supplementary Fig. 25). The femur, in contrast, appears to show the more typical trend of decreasing RCT through post-natal ontogeny (Supplementary Fig. 27). Both the femur and humerus show an increase in ACT of both elements between the neonate and fledgling stage, at which point ACT is comparable to that observed in adults.

The humerus of the neonate kestrel is thin-walled woven bone (Supplementary Fig. 26, A). Vascular canals are numerous, large, and highly irregular in shape. Many are oriented longitudinally, but circumferential canals are not uncommon. Osteocyte lacunae are visible throughout the matrix, and are relatively large. These structures also exhibit great variation in shape. Another notable feature of the hatchling humerus is the denser tissue around the periosteal perimeter, smoothing out what would otherwise be a strongly scalloped periosteal margin (Supplementary Fig. 26, A). This could be periosteum that survived the process of dehydration and clearing.

In the fledgling chick, the humerus is primarily fibrolamellar in the endosteal portion, and incipient fibrolamellar in the periosteal portion (Supplementary Fig. 26, B). The peri- and endosteal edges are very scalloped, particularly the former, indicating that bone was still actively growing in life. Vascular canals are fairly large, and primarily longitudinal in orientation but with moderate anastomoses between them. Smaller, longitudinal primary osteons are concentrated nearer the endosteal surface; large, stretched and elongated incipient primary osteons are present in higher numbers near the periosteal edge. This further supports the hypothesis that major bone deposition is still occurring along the periosteal margin.

The bone is composed of a woven matrix with patches of fibrolamellar bone in the adult kestrel. This element also has relatively low porosity, mainly in the form of simple primary canals (Supplementary Fig. 26, C&D). Few primary osteons remain, and there is no evidence of secondary osteons. This bone is dominated by longitudinal vascular channels with occasional anastomosing between them. A thick, unambiguous ICL now comprises the endosteal margin of the cortex. A clear OCL is visible in one adult individual (MVZ190892), present as a substantial periosteal layer of parallel-fibered bone that is continuous with the woven/fibrolamellar layer. The OCL in the second adult individual (MVZ190885) is thinner and not as clearly developed. Notably, this individual also retains larger patches of fibrolamellar bone, which also indicates it may be less mature in spite of the fact that both appear can be classified as “adults” based on size and plumage. In both individuals, the osteocyte lacunae are numerous and extremely small and flattened.

The femur of the neonate has a cortex that is highly asymmetrical in terms of thickness; unfortunately this element lacked bony landmarks in this age group that would aid identification anatomical orientations (Supplementary Fig. 27). In spite of this asymmetry, it still has a very circular cross-sectional shape. The cortex is made of highly porous spongy woven bone. Vascular canals thus dominate the cortex, but vary greatly in shape and size with the thickness of the bone. Around the narrow edge, channels are small, elongate, and relatively few in number (Supplementary Fig. 28, A). As the bone grades into the thick portion of the cortex, channels greatly increase in size and become more circular or oval in shape, and more numerous (Supplementary Fig. 28, B). Additionally, in the thicker part of cortex, the struts of woven bone between canals are very thin, but becomes more substantial along the thinner cortical margin. Osteocyte lacunae are rounded, irregular in orientation, and present ubiquitously throughout the matrix.

In the fledgling chick, the femoral bone is an incipient fibrolamellar complex with a reticulated network of vascular channels (Supplementary Fig. 28, C). While these canals are partly infilled with bone, the femur remains an element with high porosity. Osteons and incipient osteons are common throughout the cortex. Osteocyte lacunae are present in high concentrations throughout the matrix, and are very small as in the adults. No evidence of an OCL or ICL is yet apparent. The thickness of the cortex is only slightly asymmetrical, exhibiting a thicker region in the medial and mediocaudal cortical regions.

The adult femur is composed of parallel-fibered bone with regional fibrolamellar bone, between two layers of avascular parallel-fibered bone. These layers are the OCL and ICL, and are of equal thickness in the kestrel femur. The middle region of the cortex is highly populated with tiny osteocyte lacunae, but relatively few vascular channels. Those that are present are mainly central canals of weakly-formed primary osteons and simple vascular canals, with few oblique Volkmann’s canals connecting them. As in the humerus, one individual had a more strongly-developed OCL and smaller fibrolamellar regions than the other (Supplementary Fig. 28 E and D respectively). The cortex is of uniform thickness in the adult kestrel, and no medullary bone or thick endosteum was present in the sections analyzed in this study.

*Pyrrhura molinae* - Green-cheeked Conure (Psittaciformes)

Green-cheeked conure specimens were donated by a breeder who kept careful records of each individual. For this taxon, precise age is known, though specimens were also classified in the qualitative growth stages used for other taxa in this study. A nearly complete growth series was acquired, ranging from two-day-old neonates to somatically mature adults with many intermediate growth stages.

The RCT of the humerus is low in neonates, increasing through post-natal ontogeny to about the time of fledging (five weeks, three days old), then decreasing again by the adult stage (Supplementary Fig. 29; Supplementary Table 1). Cross sectional shape of the humerus is a bit irregular in very early chicks, shifting to elliptical around the pin-feathered (two weeks, six days old) and pre-fledging (four weeks, four days old) stages (with a longer mediolateral axis), and finally rounding out to become circular in more mature individuals.

The humerus of the neonate consists of a very thin scaffolding of woven bone (Supplementary Fig. 30, A). Osteocyte lacunae are already numerous, and display a wide variety of shapes, sizes, and orientations. Vascular canals are relatively sparse, resembling the young hummingbird bones, likely because such an extremely small, thin bone does not require a great amount of vascularization, and because periosteal and endosteal vasculature has not been fully enclosed in bone. These channels are mainly longitudinal in orientation, and have a flattened, stretched appearance. They are also very large. The one week-old chick has a humerus with very similar characteristics (Supplementary Fig 30, B), but with a slightly more expansive network of vascular channels. At both of these early stages of development, cortical thickness is uniform around all areas of the humerus.

At around three weeks post-hatching (classified as a “pin-feathered” chick), the conure humerus has a thicker cortex and the distinct spongy bone appearance characteristic of immaturity (Supplementary Fig. 30C). Vascular channels are large and very numerous. Most appear longitudinally oriented; some are circular in shape but many are still compressed and elongate, or simply irregular. Vessels around the endosteal perimeter are much smaller, while those around the periosteal perimeter remain quite large and appear to represent more recent growth. This, combined with the increased RCT, indicates that periosteal deposition of new bone is proceeding at a higher rate than endosteal resorption at this stage of development.

By approximately four and a half weeks of post-natal growth (classified as a “pre-fledgling” chick), the humerus has continued to increase in cortical thickness, but not uniformly (Supplementary Fig. 29). The cranial and craniolateral regions of the cortex are thinner. The bone retains a similar spongy appearance to that observed in the three-week-old bird, but with vascular canals that are infilling and becoming smaller. At this stage, the cortex is composed of a woven matrix punctuated by numerous vascular canals of many orientations that would have supported a reticular network of blood vessels in life (Supplementary Fig. 30, D). Some of these have osteocyte lacunae beginning to arrange themselves circularly around them, and are therefore interpreted as incipient primary osteons. Lacunae are numerous and still highly variable in size and shape.

After five and a half weeks of growth after hatching (and classified as a “fledgling”), the conure humerus has evened out in bone growth, and now has a cortex of uniform thickness around its circumference (Supplementary Fig. 29). The appearance of the bone has changed drastically with one week of additional growth (Supplementary Fig. 30, E). The cortex is now distinctly fibrolamellar, with a more moderate number of vascular channels (Supplementary Figure 29). Most canals now run longitudinally and form the centers of primary osteons, though simple vascular canals are also present. Oblique Volkmann’s canals are also not uncommon. Lacunae are still relatively ‘plump.’ A thick layer of endosteum lines the medullary cavity, and extends several projections into this space. Notably, an incipient OCL is present at this stage of growth. The outer portion of the cortex is composed of this layer of parallel-fibered tissue, with little vascularization and few osteocytes; this gives the outer surface of the bone a very smooth appearance, and indicates that periosteal deposition has stopped or slowed substantially. In contrast, no sign of an ICL is present, and the endosteal edge remains wavy and irregular, indicating that bone deposition or resorption (likely the latter) is still actively occurring here.

In the adult conure humerus, cortical thickness is uniform and cross-sectional shape is almost perfectly circular (Supplementary Fig. 29); the cortex has decreased in RCT and ACT as compared to the previous developmental stage. As described here in other small-bodied birds, the mature cortex is mainly composed of a woven matrix with simple vascular canals and small, irregular patches of truly fibrolamellar bone with primary osteons (Supplementary Fig. 30, F). This resides between a distinct OCL of parallel-fibered bone around the periosteal perimeter and a thin ICL of irregular thickness also lines the endosteal surface. Neither medullary bone nor trabeculae are present in the sections studied. Vascular canals are relatively few, and predominantly longitudinal in orientation with some anastomosing branches.

The femur is relatively thick in neonates, but like the humerus shows an overall trend of thickening into the pre-fledgling and fledgling stages before thinning again in the adult (Supplementary Fig. 31). This element in the green-cheeked conure varies from circular to moderately ellipsoid in cross-section through post-natal ontogeny. Cortical thickness also varies somewhat around the circumference of the bone. However, most of this variation occurs in early stages of growth, and by the time the fledgling stage is achieved, the femur is circular with a uniform cortical thickness.

In the neonate, the femur appears to be more advanced in ontogeny than the humerus—the cortex is already a thick layer of spongy woven bone with numerous large vascular cavities (Supplementary Fig. 32, A). (In the humerus, this stage was not achieved until three and a half weeks of growth had occurred.) In the femur, the periosteal and endosteal edges are moderately scalloped, indicating active deposition and resorption of bone is occurring. Vascular channels are primarily arranged longitudinally; many are circular or elliptical in shape, with several very flattened and stretched.

The one-week-old chick has a femur of similar appearance, but relatively smaller vascular channels (Supplementary Fig. 32, B). It is clear that infilling of these openings has already begun. Notably, however, channels are larger around the endosteal perimeter than they are closer to the periosteal surface.

In the femur of the two and a half week old bird (pin-feathered), cortical wall is composed of woven bone. Porosity is still high, but reduced relative to that observed in the younger chicks (Supplementary Fig. 32, C). Vascular canals are predominantly longitudinal with an occasional circumferential or radial channel. Lacunae are still numerous, present in roughly the same density as earlier growth stages and still relatively large and irregular in shape.

After four and a half weeks of growth (pre-fledgling), the femur of the conure is defined by woven bone transitioning to a fibrolamellar complex (Supplementary Fig. 32., D). Porosity has decreased once more relative to previous growth stages. The majority of canals are still oriented longitudinally. Some now form central canals of osteons, others represent incipient osteons, and still others remain relatively large and irregular, resembling vascular channels in younger birds.

By five and a half weeks after hatching (fledgling), femoral bone is quite mature. It is mainly composed of fibrolamellar bone with a reticular vascular pattern (Supplementary Fig. 32, E), and a thick OCL of parallel-fibered bone is present. Still no ICL is visible, and the endosteal margin is still slightly scalloped; it is likely that endosteal resorption is still underway. A prominent endosteum lines the medullary surface, and several substantial incursions of this tissue into the medullary cavity are visible. Osteocyte lacunae are small and very numerous. Many are flattened in shape, but some remain circular. Canaliculi are well-developed. Porosity has continued to decrease, and relatively few canals remain. Those that are longitudinal mainly form the central canals of primary osteons, and these are connected with an anastomosing network of oblique vessels. Several larger vessels are still present along the endosteal surface, however, indicating endosteal resorption is ongoing.

In the adult conure, the femur is composed of patches of fibrolamellar bone and woven matrix with simple vascular canals. The OCL is thick (about one-third the entire width of the cortex), and a thin, irregular ICL is also present (Supplementary Fig. 32, F). Relative cortical thickness has decreased compared to the five and a half-week-old. Porosity also seems to have decreased, with fewer oblique canals anastomosing and connecting central canals of osteons. Osteocyte lacunae remain numerous, and do not seem to have increased or decreased in number substantially through post-natal growth.

*Aphelocoma californica* - Western Scrub-Jay (Passeriformes - Corvidae)

Western scrub-jays available for this portion of the study ranged from neonate to subadult with numerous individuals of intermediate age classes. All specimens were donated by the Lindsay Wildlife Hospital. RCT of the humerus is greatest in the neonate chick, and decreases substantially until the pre-fledgling stage, after which it stabilizes and remains constant (Supplementary Fig. 33; Supplementary Table 1).

The humerus of the neonate scrub-jay has a thick cortex of spongy woven bone (Supplementary Fig. 34, A). It is highly vascularized, and vascular channels are large and highly irregular and variable in shape. An unusual feature is a solid circle of bone enclosing the medullary cavity (Supplementary Figs. 33; 34A). This circlet of bone has no major vascular perforations, except along the edge where it contacts the spongy cortex; it has been separated from the spongy bone along one edge. We interpret this as the remaining evidence of a Kastschenko line, a space that formed between the endosteal and periosteal bone after a layer of osteoid between them (a remnant of embryonic cartilage, the Kastschenko line) was disturbed during the specimen preparation process. Osteocyte lacunal density is high throughout the section; these structures are relatively large and irregular in both shape and orientation. Cortical thickness is asymmetrical, appearing to gradually thin out along one edge of the cortex. Unfortunately, this specimen was too small to track anatomical orientations, so more specific regional differences are unidentifiable.

In the pin-feathered chick, the humerus is composed of highly vascular woven bone, and retains an asymmetrical appearance, with one half of the cortex much thinner than the other (Supplementary Fig. 33). Infilling of vascular channels from the neonate phase has occurred, but these spaces are still large and numerous (Supplementary Fig. 34, B). The bone is highly populated with ‘plump’ osteocyte lacunae, evenly distributed throughout the matrix. The solid medullary circlet of bone is no longer present.

The pre-fledgling chick demonstrates similar histological features, but in a slightly thinner RCT that is nearing uniform thickness throughout its diameter (Supplementary Fig. 33). Porosity is still high, but canals are infilling with bone, and appear relatively smaller (Supplementary Fig. 34, C-E). Within each individual, there is strong regional variation in canal orientation. Around the thinner part of the cortex the bone appears woven with reticular incipient osteons; in the thicker parts of the cortex, canals are predominantly longitudinal in orientation and are still very open, giving the bone a spongy appearance. In one individual there is even a region of circumferentially-oriented channels, giving it a distinct laminated appearance (34, C). However, the bone matrix has a uniformly dense arrangement of osteocyte lacunae. Some are beginning to become small and elongate, while others remain circular or irregularly-shaped. A thick, variable layer of endosteum also lines the medullary cavities of all pre-fledgling scrub-jays. Various small projections and incursions of this soft tissue into the medullary cavity are not uncommon.

By the time the fledgling stage is achieved, cortical thickness has become completely uniform throughout humeral circumference (Supplementary Fig. 33). The cortical bone has substantially matured, and is now fibrolamellar, interspersed with large regions of parallel-fibered bone and woven matrix with primary vascular canals. Vascular channels are much smaller than in the pre-fledgling stage and mainly longitudinal, though anastomosing between them is not uncommon (Supplementary Fig. 34, F&G). Both incipient and mature primary osteons and are observed. A thin, incipient OCL has developed in the form of a periosteal layer of parallel-fibered bone, but no ICL is apparent.

The humerus of the subadult is almost identical to the humeri of fledgling birds (Supplementary Fig. 34, H), evidence that external indications of maturity (e.g. body size and feathers) does not always correlate with histological characteristics. The subadult specimen sample in this study may simply be a developmental outlier, or may represent an unusual ontogenetic trend unique to scrub jays; future studies with a greater sample size will be able to address this question. The cortex is composed of both regional fibrolamellar bone and regional parallel-fibered with simple vascular canals. Some circumferential vessel channels are visible but most are longitudinal. A very thin layer of parallel-fibered bone is irregularly present around the periosteal margin of the bone, mainly along the cranial side; this may represent an OCL in the very early stages of formation. Osteocyte lacuna density is still high. While the orientation of osteocytes is still disorganized, most are small and elliptical at this stage of development. A prominent endosteum is again present, but in a much thinner layer; several delicate extensions of connective tissue project into the medullary cavity.

The femur follows a similar trend of cortical thickness compared to the medullary cavity as seen in the humerus (Supplementary Fig. 35), with a slight difference at the fledgling stage when the cortex acquires extra tissue before stabilizing to previous relative thickness in the subadult scrub-jay.

The femur of the neonate chick has a very thick cortex in terms of RCT and ACT, composed of highly porous spongy woven bone (Supplementary Fig. 36, A). The cortex is highly uneven, being very thin along the lateral border, thick along the medial margin, and intermediate in the cranial and caudal areas. Canals are irregular in shape and orientation oriented radially. There is also a solid, bony ring around at least a portion of the medullary cavity, similar to that seen in the humerus but more incompletely detached from the bone of the rest of the cortex (Supplementary Figs. 35; 36,A). Again, we interpret this as evidence that a Kastschenko line was present in the growing chick. Comparably, this feature is so thin that it lacks much vascularization, but still has many osteocyte lacunae. Many lacunae are generally present throughout the cortex of the hatchling femur, and display the irregular, ‘plump’ morphology characteristic of immature bone.

In the pin-feathered chick, the RCT and ACT of the femoral cortex has been reduced (Supplementary Fig. 35). This is the only major difference, however, because the bone is still predominantly spongy with a high porosity and large number of rounded osteocyte lacunae (Supplementary Fig. 36, B). There is an asymmetry in cortical thickness caused by a thickening along the medial margin, relative to other regions, which have now developed a more even width. No tissue is present in the medullary cavity, except one ‘floating,’ thin half-circle of bone located in the medullary cavity near the lateral cortex. This perhaps represents the remainder of the previously-mentioned bony ring observed in the neonate.

The cortex of the pre-fledgling chicks continues to reduce moderately in RCT, with some general variability observed in this age class. The cortical wall has now evened out to the point of near-uniform thickness. The matrix of the cortex is woven with many vascular canals (Supplementary Fig. 36 C-E). These large pores have started to be filled in with bone, and are considerably smaller than in the pin-feathered and neonate chicks. However, the shapes and orientations of the channels are still highly variable. Longitudinal and circumferential canals are common, and some oblique channels are also observed. Some are in the process of becoming osteons, as evidenced by the organization of fibers and lacunae circularly around them. A relatively thick layer of endosteum lines the medullary cavity, with some projections into this central space.

Fledgling individuals have femora composed variably of mostly parallel-fibered bone in one specimen (Supplementary Fig. 36, F) and fibrolamellar bone in another (Supplementary Fig. 36, G). In both, thick remnants of endosteum are also present. Vascular canals are now relatively smaller and fewer in number, and many are the central canals of primary osteons. A few are circumferential in orientation. Primary osteons appear in clusters through most of the cortex located nearer the endosteal surface than the periosteal one. In the specimen with a predominantly fibrolamellar cortext a thin OCL is forming, composed of parallel-fibered bone with no osteons and little to no vascular canals. In the other, the OCL is thicker are more well-developed. The endosteal edge remains irregular and wavy, indicating that bone deposition and resorption along this margin have not yet slowed down enough for the ICL to begin formation.

As in the humerus, the femur of the subadult specimen is very similar to that of the fledgling chicks, but with a relatively narrower cortex (both in terms of ACT and RCT) and even smaller vascular canals (Supplementary Fig. 36, H). The OCL still appears thin and incipient. Beginnings of an ICL are visible along some portions of the endosteal margin. This bone was densely cellular in life, and many small, mature osteocyte lacunae with full canalicular systems are present. A thick layer of endosteum remains present, likely an indication of the relative immaturity of the bone.

*Haemorhous mexicanus* - House Finch (Passeriformes)

House finches used in this study represent the full continuum of a post-natal growth series (neonate, pin-feathered chick, pre-fledgling chick, fledgling chick, and adult), received from the Lindsay Wildlife Hospital. The humerus of the naked chick was too cartilaginous to survive the skeletonization process. Therefore, analysis of humeral histology begins with the pin-feathered stage.

In the house finch, the humerus has a very high RCT and ACT in young chicks, and subsequently undergoes a rapid decrease in bone thickness, followed by a steady increase in relative cortical thickness to maturity (Supplementary Fig. 37). ACT is highest in the pin-feathered chick, and decreases in the fledgling and pre-fledgling stages, and finally increases moderately at the adult stage.

The humerus of the pin-feathered chick has a very thick, asymmetrical layer of spongy woven bone in the cortex surrounding a remarkably small medullary cavity (Supplementary Fig. 38, A). Like the scrub-jay chicks, a solid circlet of bone contacts portions of endosteal edge of this spongy tissue and lines the medullary cavity (Supplementary Figs. 37; 38,A). Therefore, this taxon also preserves evidence of the remnant of the cartilaginous precursor in long bone formation (i.e., a Kastschenko line), represented by the space where this soft tissue was present in the living organism. Vascular cavities in the spongy portion of the cortex are very large and irregular in shape, though primarily longitudinal in orientation. They are separated by very thin struts of woven bone with moderate numbers of osteocyte lacunae.

At the pre-fledgling stage, cortical thickness (ACT and RCT) decreased substantially (Supplementary Fig. 37), indicating that periosteal deposition is proceeding at a much lower rate than endosteal resorption. The cortex is primarily composed of chondroid bone. Considerable in-filling of vascular channels has occurred relative to the pin-feathered stage (Supplementary Fig. 38, B&C). Most channels are oriented longitudinally. A few incipient osteons are also present.

In the fledgling chick, the humeral cortex is of uniform thickness and composed primarily of chondroid bone with a relatively low porosity (Supplementary Fig. 38, D). The cranial margin is slightly flattened relative to other portions of the cortex. An ICL has started to form and is strongly visible under polarized light. The outermost portion of the cortex is nearly avascular, and appears to be in the early stages of forming the OCL. Vascular canals are distributed throughout the matrix between them, in the form of longitudinal central canals of primary osteons. These are relatively small compared to previous developmental stages.

The humeral cross-section of the adult house finch is a nearly-perfect circle of even cortex composed of thick OCL (comprising about one-third to one-half of the cortex), which borders a middle layer composed of chondroid bone with simple vascular canals and regional fibrolamellar bone. A thin ICL is also present (Supplementary Fig. 38, E). Osteons are slightly more numerous than in the fledgling chick (some of these are in the process of developing), and several Volkmann’s canals are present connecting them. However, overall porosity remains quite low and the predominant vascular pattern is longitudinal canals. Osteocyte lacunae are present in moderate numbers and most have a compressed shape, though some in the middle layer remain relatively rounded.

In the femur of young individuals (the neonate and pin-feathered chick) RCT is high. This decreases substantially as individuals grow but undergoes a notable relative increase at the fledgling stage before decreasing to its lowest relative thickness in the adult (Supplementary Fig. 39). Absolute thickness increases from neonate to the pin-feathered stage, where it is highest, then decreases through later stages; the adult has the thinnest absolute cortex.

The femur of the neonate is composed of spongy bone with a very high porosity (Supplementary Fig. 40, A). As in the subadult house finch humerus, and the humerus and femur of scrub-jay chicks, the medullary cavity is sharply defined by a solid circlet of bone with minimal vascular canals and osteocyte lacunae (Supplementary Fig. 39). Large vascular canals are the dominant feature of the cortex, highly variable in shape and orientation, ranging from oval to circular; only very thin struts of bone separate a channel from its neighbors. These thin deposits of bone have dense populations of osteocyte lacunae, also highly variable in shape and size.

In the pin-feathered chick, the femur has a very similar appearance to that of the neonate. The cortex is primarily spongy with huge vascular canals separated by narrow struts of bone (Supplementary Fig. 40, B). The endosteal circlet of bone is detached from the endosteal edge of the bone and now is visible ‘floating’ in the medullary cavity (Supplementary Figs. 39; 40,B). Once again, this feature and the details of its development are very similar to ontogenetic changes observed in the scrub-jay femur, which we interpret as another example of evidence of a Kastschenko line. The bony trabeculae between vascular channels are slightly thicker than in the neonate, but the size of channels remains very large.

More substantial change in histological characteristics occurs by the pre-fledgling stage. Here, ACT and RCT have decreased and evened out through the diameter of the femur (Supplementary Fig. 39). The bone is predominantly chondroid tissue (Supplementary Fig. 40, C&D). Much infilling between vascular canals has occurred relative to earlier growth stages, and the porosity of the bone is greatly decreased relative to earlier growth stages. Channels are still fairly numerous and large, and most are oriented longitudinally. Osteocyte lacunae are still present in dense populations, and are large and rounded.

In the fledgling individual, RCT has once again increased, as observed in multiple other taxa. The cortex is fibrolamellar, still with a moderate number of relatively large, longitudinally-oriented vascular canals, many of which have become or are becoming central canals of primary osteons (Supplementary Fig. 40, E). Some regions of the cortex are sparse in osteons, while others have many clustered together. The OCL is thicker and more clearly-developed than in the pre-fledglings. A thin layer of endosteum lines the medullary cavity.

The femur of the adult house finch is extremely thin-walled. The fibrolamellar layer of the fledgling stage appears to have been eroded away, in some regions present only as a thin endosteal layer and in others gone completely (Supplementary Fig. 40, F). The OCL now dominates the cortex, comprising on average half the thickness of the bone. Therefore, the adult femur is dominated by parallel-fibered bone with some regions of woven matrix with simple vascular canals. An ICL has not formed. Instead, the endosteal margin remains uneven in appearance, indicating that resorption along this border was still active (Supplementary Fig. 40, F) Osteocyte lacuna density remains moderately high; these structures have decreased in relative size, but many remain rounded.

*Bubo virginianus* - Great Horned Owl (Strigiformes)

The growth series of the great-horned owl was less complete than that of the barn owl. Only a few individuals, ranging in age from pin-feathered chick to adult, were procured. They also were donated by the Lindsay Wildlife Hospital. In the great-horned owl, RCT and ACT of the humerus are thickest in the pin-feathered chick, the youngest bird available to this study, and decrease through ontogeny (Supplementary Fig. 41). This decrease is initially very rapid going from pin-feathered to fledgling chick but slows as maturity is approached.

The humeral cross-section of the pin-feathered chick is circular with a highly uneven cortex (Supplementary Fig. 41); bone is thickest in the caudal and medial areas, and relatively thinner in the lateral and cranial cortex. As in asymmetrical cortices of other young chicks, the thinner regions appear denser by comparison, and have a lower porosity. The cortex is composed of woven bone with a generally spongy appearance and fairly high porosity (Supplementary Fig. 42, A). Many vascular canals are circular and longitudinal, but larger, irregular vessels are not uncommon. A few incipient osteons are already present, as are many osteocyte lacunae, complete with canaliculi. A thick layer of endosteum is present in the medullary cavity.

The tissue in the humerus of the pre-fledgling individual is generally very similar to that of the pin-feathered chick. It is composed of woven bone with high porosity, however, in this later developmental stage developing primary osteons are approaching maturity, and simple vascular canals are relatively smaller (Supplementary Fig. 42, B). Overall porosity has decreased.

In the fledgling chick, the cortex is evened out in thickness and is composed of fibrolamellar bone (Supplementary Fig. 42, C). Many vascular canals remain but are much smaller than in the pin-feathered chick, having been infilled with bone. Canals of all orientations are present, with an overall dominance of reticular to plexiform-laminar orientations (Supplementary Fig. 42, D). High numbers of longitudinal primary osteons are present, particularly concentrated in the endosteal half of the cortex. Regional areas of bone with parallel collagen fibers and no vascular channels along the peri- and endosteal edges indicate that the ICL and OCL have begun to form (Supplementary Fig. 42, D).

Mature bone in the adult great-horned owl humerus is formed of a fibrolamellar complex between a thin ICL and OCL (Supplementary Fig. 42, E&F). The cortex is of even thickness, and roughly circular except for the caudal edge, which is slightly flattened. The ICL is thicker on average than the OCL, and is composed of lamellar bone. The OCL is formed of parallel-fibered bone. Vascular canals are very small in size, yet numerous. They are predominantly longitudinal in orientation. Nearly all are the central canals of primary osteons. Osteocyte lacuna density is very high, though lacunae are quite reduced in size.

Similar to the humerus, the femur shows a trend in reduction in ACT through ontogeny, but with a slight increase in cortical width in the adult (Supplementary Fig. 43). RCT is very high in the pin-feathered stage, and decreases drastically by the pre-fledgling stage; it remains constant throughout the rest of ontogeny (Supplemental Table 1). Also as in the humerus, the femur of the pin-feathered chick has a cortex of highly asymmetrical thickness. It is much wider along the medial and caudomedial portion than elsewhere (Supplementary Fig. 43). Once again, porosity is relatively higher in the thicker regions, giving them an appearance of higher density. A very large erosional cavity is present in the caudomedial cortex. The overall appearance of the bone is woven, with much variation in the size and shape of vascular canals (Supplementary Fig. 44, A). However, the individual was old enough that a good number of vascular canals are infilled and relatively small, giving the femur a more mature appearance than the humerus. Canals are predominantly longitudinal in orientation. Many osteocyte lacunae are present. They are still relatively large, but many have taken on a more compressed shape. Cortical thickness is greatly evened out by the pre-fledgling stage (Supplementary Fig. 43). Vascular canals are greatly reduced in size, and most have become longitudinally-oriented osteons (Supplementary Fig. 44, B). The only remaining signs of immaturity are scalloped periosteal and endosteal margins, and vascular pores slightly larger than in later growth stages.

Cortical bone is fully fibrolamellar in the fledgling chicks (Supplementary Fig. 44, C). The size of most vascular canals is further reduced, though they are still distributed throughout the bone in high numbers. Most are now longitudinal and form the central canals of numerous primary osteons. Some regions of the peri- and endosteal margins show evidence of a developing OCL and ICL, though the bone remains primarily fibrolamellar, an indication it was still actively growing.

The femur of the adult is formed of a similar cortex of fibrolamellar bone, now between an ICL and OCL. The ICL is substantially thicker than the OCL in this element (Supplementary Fig. 43). The ICL is composed of lamellar bone. Vascular canals are highly reduced in size, but remain present in large numbers; once again, most form the central canals of the numerous primary osteons that populate the cortex (Supplementary Fig. 44, D). Volkmann’s canals are also common. Also as in the humerus, osteocyte lacunae are present in high numbers but are extremely small and flattened in appearance.

*Tyto alba* - Barn Owl (Strigiformes)

Barn owls are well-represented, with numerous specimens collected ranging in age from downy chick to mature adult; in this case, “downy” does not equate to neonate—the chick had not yet started growing pin feathers, but was of larger body size than neonate. All were donated by the Lindsay Wildlife Hospital. As with the other birds in this study, cortical wall thickness is dynamic through post-natal ontogeny (Supplementary Figs. 45 & 47; Supplementary Table 1). In the humerus of the barn owl, RCT is relatively high at the downy and pin-feathered stages, and gradually decreases through maturity. ACT varies relatively little, thickening moderately between the downy and adult stages, though these two end points are very similar in cortical thickness.

The humerus of the downy chick is composed of woven bone with a high porosity (Supplementary Fig. 46, A); however, areas of bone between vascular channels are much thicker than in chicks of other taxa (e.g., the red-tailed hawk). As seems typical of young avian chicks, most vascular canals are oriented longitudinally and range greatly in size and shape. Osteocyte lacunae are very large and ‘plump.’ The cortex is thickest along the cranial and caudal margins, and thins out in the lateral and medial areas.

Cortical thickness showed much variability in the pin-feathered chicks used in this study (46, B&C). One had a relatively thin cortex (less bony tissue than in the downy chick), while the other had a very thick cortex (more bony tissue that the downy chick). In the former individual, cortical thickness is essentially uniform, with a slight narrowing along the craniomedial and caudolateral margins. In the latter individual, a much greater asymmetry is observed: the cranial portion of the cortex is disproportionately thick relative to the other regions. However, they are otherwise similar in terms of other histological characteristics. As in the downy chick, the cortex is made of woven bone with a relatively high porosity (Supplementary Fig. 46, B&C). Vascular channels remain large and highly variable in shape, size, and orientation; many are longitudinal but all other orientations are also observed. Around some areas of the cortex, a trend of smaller, more circular canals nearer the periosteum and larger, more irregular canals nearer the endosteum is observed, but this pattern does not characterize the whole of the element. Endosteal tissue is present in the medullary cavity of both pin-feathered chicks, as incursions into the cavity of a variety of shapes and sizes (some bony struts, others amorphous patches of bone).

The cortex of the pre-fledgling chicks is discernibly thinner and less porous. A general asymmetry in cortical thickness persists, with the cranial and medial margins notably wider than the caudal and lateral (Supplementary Fig. 45). The bone is still primarily woven, but with a lower porosity than earlier developmental stages. The cortex was transitioning to fibrolamellar bone (Supplementary Fig. 46, D&E) with many incipient primary osteons present. Many of the vascular canals are now clearly circular and longitudinal (i.e., variation in channel orientation is reduced), although variation in size of these channels persists. Thicker regions of the cortex have a higher porosity, while the thinnest regions appear noticeably denser. Osteocyte lacunae are present in fairly high numbers and have, however, acquired the very small, compressed appearance of more mature bone.

In the fledgling barn owl, RCT has not decreased substantially, but it has evened out and is now uniform around the diameter of the humerus (Supplementary Fig. 45). The element at this stage is now almost completely fibrolamellar bone. In some regions, vascular canals remain fairly large and are still developing into primary osteons, with a clear trend of smaller canals and mature osteons located predominantly in the more endosteal portion of the cortex and larger, more irregular canals located primarily in the more periosteal portion of the cortex (Supplementary Fig. 46, F). The bone closer to the periosteal margin therefore has a much immature appearance, and was clearly still being actively deposited. In this outer immature region, vascular channels are primarily longitudinal and variable in shape. The inner, more mature region of the bone is characterized by numerous mature longitudinal osteons, connected by a reticulating network of vascular canals. This layer is thicker, giving the bone a general reticulated appearance in terms of predominant vascular orientation. No evidence of an ICL or OCL is yet apparent, and no endosteum was observed in the sections studied.

The cortex of the subadult barn owl is very thin (Supplementary Fig. 45), and is composed of dense fibrolamellar bone (Supplementary Fig. 46, G). Vascular canals are reduced in size relative to those of in the fledgling chick. Channels that are present are now so infilled that they are only marginally larger than the extremely numerous osteocyte lacunae. The general vascular pattern is best characterized as reticular, though some areas are dominated by circumferential canals. No ICL is yet present, but a thin, incipient OCL of varying thickness does circle the periosteal margin. Throughout much of the cortex, there is a subtle but discernable difference in tissue organization within the middle layer of the cortex: the more periosteal half of this layer is more organized with smaller vascular spaces, and the more endosteal half is often more disorganized with larger vascular spaces and larger patches of woven matrix (Fig. 46, G). Notably, in the craniolateral portion of the cortex, these two regions appear to be separated by a thin layer of parallel-fibered bone resembling a growth mark such as an annulus. However, this structure only spans this small region of the cortex and is not continuous around its diameter.

The humerus of the adult barn owl is mainly composed of very thin-walled fibrolamellar bone (Supplementary Fig. 46, H). The predominant vascular pattern is now circumferential in one specimen and reticular in another. These channels are extremely narrow. Primary osteons are dispersed throughout the fibrolamellar cortex, and are present in high densities. A distinct ICL of parallel-fibered/lamellar bone was present in one of the individuals classified as an adult (MVZ190872) but lacking in the other (MVZ190877). However, a parallel-fibered OCL was present in both individuals, so by that measure both had achieved final diametric size.

The femur, in contrast to the humerus, shows much greater variability in cortical thickness (Supplementary Fig. 47). Though RCT is highest in earlier developmental stages, and shows an overall trend of thinning out, there is much overlap in values between individuals of different ages. Average absolute thickness also appears change more dynamically than in the humerus, increasing dramatically at the pin-feathered stage, then decreasing and stabilizing in subsequent stages until a sudden decrease in the subadult stage before increasing again in the adult (Supplementary Table 1).

The femoral cortex of the downy chick is uneven in thickness. The medial region is thickest, and a substantial narrowing occurs along the caudolateral cortex (Supplementary Fig. 47). A large erosional cavity was also observed in the caudal cortex of one section. The bone is woven, with a moderately high porosity. The femur of the downy chick is relatively more mature than the humerus, with vascular canals more infilled with bone and more regular in shape (Supplementary Fig. 48, A). These channels are predominantly circular and run longitudinally. Regional differences in bone maturity are apparent in the downy chick femur. The endosteal two-thirds of the bone appear more mature, with smaller vascular channels of a more consistent shape. The periosteal one-third, in contrast, appears more immature, with wider vascular canals demonstrating greater variation in size and shape.

As in the pin-feathered chick humerus, there is enormous variation in cortical thickness of the pin-feathered chick femur. In one individual, the cortex is roughly of the same relative thickness as that of the downy chick, and appears uniform throughout its diameter. In the other pin-feathered barn owl, relative cortical thickness is much greater than in the downy chick, and shows a disproportionate thickening along the medial margin. This femur also has a large space where a neurovascular bundle perforated the bone. The pin-feathered stage for altricial birds is a time when the chicks stay in the nest and are fed as much as possible by the parents. Because this is a critical stage of nutrient acquisition and growth, this variation in cortical appearance may reflect differences in food resources available to each of these chicks.

Once again, however, microstructural characteristics remain generally constant (Supplementary Fig. 48, C). In both pin-feathered chicks, the femur is composed of woven bone of moderate porosity. Vascular channels are large and irregularly shaped, though primarily longitudinal in orientation. The regional differences in bone maturity of the downy chick are still present in some areas, manifesting mainly as a higher concentration of osteocyte lacunae in the endosteal portion of the cortex. These lacunae also appear more mature than those in the periosteal portion; they are beginning to acquire the small, flattened appearance of mature osteocytes and elaborate canaliculi have developed. Though vascular canals remain large, some have become primary osteons, evident from the circular arrangement of lacunae around them. Therefore, there are large patches of fibrolamellar bone at this growth stage.

In the pre-fledgling barn owl, the cortex remains relatively thick and has mainly evened out, except for a thinning in the cranial region (Supplementary Fig. 47). In this developmental stage, the bone has almost entirely transitioned to mature fibrolamellar (Supplementary Fig. 48, D&E), with only a few large, simple vascular canals near the periosteal margin. Many longitudinal primary osteons are present, generally concentrated in the endosteal portion of the cortex. Near the periosteal edge, canals are circumferential, larger, and more irregular in shape. Anastomosing vessels are common throughout. No sign of an OCL or ICL is apparent. Spongy woven bone crosses the medullary cavity in multiple irregular struts.

In the fledgling barn owl, vascular canals dispersed throughout the fibrolamellar cortex are smaller and less numerous than in younger individuals. This stage also exhibits early signs of OCL development, though precursors of an ICL are not yet visible (Supplementary Fig. 48, F). The endosteal margin still appears to be undergoing active change and resorption. Overall porosity of the element is decreased, except an opening representing a perforating foramen present in the craniomedial cortex. Osteocyte lacuna numbers are very high.

The femoral cortex of the subadult is composed of fibrolamellar bone with even smaller vascular canals than the fledgling stage. Many form the central canals of osteons, but circumferential and oblique canals are also common (Supplementary Fig. 48, G). The overall vascular appearance is reticulated. The cortex is thin-walled, circular, and even throughout its diameter. An OCL is clearly present, thicker and more developed than in the fledgling stage. An ICL, however, is still absent. Osteocyte lacunae are small and extremely numerous, with a highly elaborate canalicular network.

Adult barn owls have bone of essentially the same appearance as subadult owls, but with a distinct ICL along a mature endosteal margin (Supplementary Fig. 48, H). Interestingly, this relatively late-developing layer of parallel-fibered bone is thicker than the OCL. Longitudinal, circumferential, and oblique primary osteons were moderately numerous, but no secondary osteons were observed. Some of these vascular spaces are simple vascular channels, rather than osteons.

*Elanus leucurus* - White-tailed Kite (Accipitriformes)

Unfortunately, no extremely young kites were collected for this study, so analysis begins at the pin-feathered chick stage; these specimens were from the Lindsay Wildlife Hospital. In terms of RCT, the humeral cortex shows a trend of steady narrowing through post-natal development (Supplementary Fig. 49). ACT remains fairly constant across different growth stages, but shows a minor decreasing trend when comparing the earliest and latest age classes..

In pin-feathered kites, the thick humeral cortex is very asymmetrical, with thin cranial and medial regions relative to the rest of the diameter (Supplementary Fig. 49). The cortex is composed of highly porous woven bone. A very large cavity is present in the lateral cortex, likely where a perforating vessel passed through the bone. Vascular canals are large and range in shape from circular to elongate to irregular (Supplementary Fig. 50, A. This range of shape is paralleled by the variety of observed canal orientations: while longitudinal canals predominate, oblique and circumferential canals are also present.

In the pre-fledgling stage of life history the humerus has a rounded-triangular shape in cross section, emphasized in part by a continued asymmetry in cortical thickness: now the medial and lateral margins are relatively narrow, and the medial, caudomedial, and craniomedial areas are very wide (Supplementary Fig. 49). Differences in histological structure of the bone parallel these differences in cortical thickness. The bone appears much denser and closer to fibrolamellar in the narrow regions, and much more spongy and porous in the thicker regions (Supplementary Fig. 50, B&C). Throughout the cortex, primary and incipient osteons are visible, though in higher numbers in the denser, thinner regions. Longitudinal canals predominate, with smaller, rounder canals generally in the thinner cortex and larger, more irregular channels in the thick cortex. In both pre-fledglings studied, delicate masses of endosteum extend into the medullary canal.

The rounded-triangle cross-sectional shape persists into the fledgling stage. Cortical thickness becomes more even, though the lateral region remains distinctly narrow and the medial region distinctly wide (Supplementary Fig. 49). Cortical bone is now fibrolamellar with a reticular network of vascular channels dispersed throughout (Supplementary Fig. D, E, &F). Once again, the narrower region of cortex is denser. That is, fewer vascular canals are apparent in this region relative to the rest of the bone. While the endosteal two-thirds of the cortex is generally composed of a mature fibrolamellar complex, a thin yet distinct layer of a porous woven matrix lines the periosteal one-third of the element, indicating active bone deposition was still occurring at this growth stage. Most vascular canals are small and longitudinal, and an abundance of osteons is observed, except for the outer rim of the bone where channels in the newer bone are larger and more variable in shape and orientation. There is no sign of an incipient OCL as in the fledgling stage of some other birds.

The adult humerus is markedly different from the fledgling. The rounded-triangle shape is now very subtle, caused only by a slight flattening along the medial cortex and a subtle apex formed at the caudal area. Cortical thickness is very close to uniform, with only a slight thickening at the caudal apex and along the cranial cortex (Supplementary Fig. 49). The humerus is composed of a thick layer of fibrolamellar bone, with patches of woven matrix with simple vascular canals in between an OCL and ICL (Supplementary Fig. 50, G). In some places these two layers of parallel-fibered bone are of even thickness, but the ICL appears thicker than the OCL. The bone of the ICL is more highly organized, verging on lamellar, while the parallel-fibered bone of the OCL grades into the middle layer of the cortex. Vascular canals are few in number and primarily longitudinal in orientation. Most form the central canals of osteons, which have been so reduced in size they are hardly larger than the many osteocyte lacunae dispersed throughout the cortex.

The femur shows a similar pattern of ontogenetic reduction in RCT and ACT to the humerus (Supplementary Fig. 51). The femur of the pin-feathered kite chick has a thick, uneven cortex of woven bone, generally spongy in appearance. The cranial and craniolateral margins are thinnest, and the widest area is around the caudolateral region (Supplementary Fig. 51). Also, a large cavity is present in the caudal cortex, part of a perforating foramen. The cortical bone has a higher porosity in the thicker regions of the cortex, and appears relatively denser in the thin areas, though the periosteal-most bone has very large vascular canals even in these areas (Supplementary Fig. 52, A). The great variation in size, shape, and orientation of vascular canals is an indication of the very young age of the specimen. Some endosteum is present as projections into the medullary cavity.

Pre-fledgling chick femora still have a relatively thick cortical wall, but the overall thickness at this stage is much more even than in the downy chick (Supplementary Fig. 51). The cranial and lateral margins are still thinner, but only marginally so. Overall porosity of the bone is also more uniform. The large vascular cavities in woven bone are somewhat infilled and are now smaller in diameter, though still much larger and more numerous than in a mature individual (Supplementary Fig. 52, B&C). Some channels have become highly reduced in size and now form incipient osteons. The bone was clearly transitioning from woven to fibrolamellar. Delicate projections of endosteum are also present in the pre-fledgling chicks observed.

The femur of the fledgling kite is round in cross-section, with a moderately thicker ACT as compared to the pre-fledgling stage (Supplementary Fig. 51). This is unusual, as many of the other birds included in this study appear to undergo a secondary phase of cortical thickening during this developmental stage. In the kite fledgling femur, the cortex still has minor asymmetries in thickness, though now substantially different from the patterns observed in early stages: the cranial edge is now the widest part of the cortex, while the caudal area is now the thinnest. In the endosteal two-thirds to three-quarters of the cortex, bone is fully fibrolamellar. A thin periosteal layer of bone with a woven matrix with more open, simple vascular canals is present irregularly, indicating that periosteal bone deposition is still active (Supplementary Fig. 52, D, E, &F). Vascular channels are highly reduced in size, but remain numerous. Many longitudinal primary osteons and oblique Volkmann’s canals are present. The vascular pattern is best characterized as reticular. Osteocyte lacunae are also numerous, small in size, but still rounded in shape. No sign of an ICL is yet apparent, though an incipient OCL is present. The endosteal margin remains somewhat irregular, suggesting that resorption at this surface is still on-going.

The femur of the adult white-tailed kite is very thin-walled, and of close to even cortical thickness throughout its diameter. It is easily distinguishable as the bone of an adult individual by the presence of an ICL and OCL on either side of a layer of fibrolamellar bone/woven matrix with simple vascular canals (Supplementary Fig. 52, G). The ICL and OCL are similar in thickness, though the ICL is more strongly formed and organized. The fibrolamellar bone is much less porous than in the fledgling individuals. Vascular channels are smaller and few in number, mainly present as longitudinally-oriented central canals of primary osteons and simple vascular channels.

*Buteo jamaicensis* - Red-tailed Hawk (Falconiformes)

This taxon is represented by an incomplete growth series, ranging from late-stage downy chick to adult. Specimens were donated by the Lindsay Wildlife Hospital. RCT and ACT of both the humerus and femur decrease steadily with age through post-natal growth (Supplementary Figs. 53&55). However, this apparent trend may be biased by the age classes represented, which do not include pin-feathered chicks (the stage at which RCT is often highest) or fledgling chicks (the stage at which cortical thickness often undergoes a secondary increase before thinning out in the adult in many other taxa).

In the downy chick, the humerus is composed of a thick layer of spongy cortex that thins slightly along the lateral and caudolateral margins (Supplementary Fig. 53). A thick layer of endosteum is also present, lining portions of the medullary cavity and extending across the cavity in projections. The cortex is composed of woven bone with high porosity (Supplementary Fig. 54, A). On an absolute scale, the vascular canals are close in size to those of very young chicks of other taxa (e.g., kestrels, scrub-jays, wild-turkey), but appear relatively smaller because this larger bone in a bigger bird is composed of a greater number of canals. The bony trabeculae between them are thin, also similar to very young chicks of other taxa, though this is not a neonate. The size/orientation of the canals varies greatly, and the shapes ranges from oval to circular to tube-like. Osteocyte lacuna density is very high.

The humerus of the pre-fledgling chick is very similar to that of the downy hawk chick, but with a relatively thinner cortical wall (Supplementary Fig. 53). The cortical thickness remains asymmetrical, and is thinnest along the lateral margin. The bone is still composed of highly porous spongy bone, but vascular canals have begun to fill in with bone, and thus appear relatively smaller than the previous growth stage (Supplementary Fig. 54, B). Most are round and longitudinally oriented, though vessels of irregular shape and orientation are still present. The density of osteocyte lacunae is very high; lacunae have full canalicular systems by this growth stage.

In the adult red-tailed hawk, the cortex of the humerus is of approximately uniform thickness throughout its circumference (Supplementary Fig. 53), and composed of a thick middle layer of fibrolamellar bone (Supplementary Fig. 54, C&D). A distinct OCL and ICL are both present; the ICL is thicker and more prominently developed. In one of the adult individuals included in this study, a trabecular ‘loop’ of the ICL extends into the medullary cavity, which most likely contained neurovascular structures was continuous with a nutrient foramen at the surface of the element, similar to that observed in the wild turkey. Both these layers of parallel-fibered bone are relatively thin, and the fibrolamellar bone comprises the bulk of the cortex. Though the vascular canals are quite reduced in size, they remain numerous even in the adult. Most are oriented longitudinally and constitute central canals of primary osteons, though regional clusters of secondary osteons were also observed (Supplementary Fig. 54, D). Numerous small oblique and circumferential canals are also present. Osteocyte lacunae are extremely small and numerous.

The downy chick femur looks very much like the downy chick humerus; the cortex is composed entirely of highly porous spongy woven bone, though in this case only the endosteal margin is wavy, indicative of the greater maturity of this element (Supplementary Fig. 56, A). Another difference is greater infilling of the vascular canals, also evidence of the greater relative maturity of the femur compared to the humerus. In this sense, the femur is at a later developmental stage than the humerus at hatching. The predominant orientation of vascular canals is longitudinal, but the shape of these openings varies greatly. In cross-section, the femur appears circular, and has a cortex of near-uniform width except for a narrowing at the caudal margin (Supplementary Fig. 55). As in the humerus, a large amount of endosteum is present in the medullary cavity and appears to be forming several trabeculae-like extensions. Many small osteocyte lacunae are already present throughout the bone, and some canalicular systems have developed.

The pre-fledgling femur also is composed of spongy bone, but with vascular cavities much more in-filled, and a relatively thinner cortical wall. Cortical thickness remains asymmetrical, and appears to be labile in these early stages of growth; in contrast to the thin caudal margin observed in the downy chick, the caudal margin is the thickest in the pre-fledgling, while the cortex is thinnest in the lateral and cranial areas (Supplementary Fig. 55). Around the thinner margins of the cortex, the bone is denser, with smaller vascular pores. Most vascular channels run longitudinally, though short oblique, radial, and circumferential canals are also present; the overall pattern of orientation seems to be an amalgam of all directions. At this stage, some of the smaller longitudinal canals have become or are becoming primary osteons, (Supplementary Fig. 56, B). These are mainly found in the thinner, denser regions of the cortex. Osteocyte lacuna density remains very high, and many lacunae with canaliculi are now also observable.

In the adult red-tailed hawk, the femur is nearly perfectly circular, and of even cortical thickness throughout its diameter (Supplementary Fig. 55). Though the cortex is slightly thinner relative to the medullary diameter than in the humerus, the femur has the same histological traits: two layers of parallel-fibered bone encircling a region of fibrolamellar bone. The fibrolamellar layer is the thickest, followed by the ICL, and then an OCL (Supplementary Fig. 56, C&D). Osteocyte lacunae are extremely small but present in high numbers; their dense populations even extend into the OCL and ICL. The size of vascular canals has become so highly reduced that many are not much bigger than the osteocyte lacunae. Most form the longitudinally-oriented central canals of primary osteons, although reticulating osteons are also common. This fibrolamellar bone appears highly organized, verging on the parallel-fibered range of the bone-type spectrum; numerous areas of parallel bundles of collagen fibers are visible under polarized light.

Castanet J, Rogers K, Cubo J, and Boisard J. 2000. Periosteal bone growth rates in extant ratites (ostriche and emu). Implications for assessing growth in dinosaurs. *Cr Acad Sci Iii-Vie* 323:543-550.
