# Supplementary material for: A histological survey of avian post-natal skeletal ontogeny
Source: PeerJ. 2021 Oct 1;9:e12160. doi: 10.7717/peerj.12160 (PMC8489414; doi:10.7717/peerj.12160)
Supplement: Supplemental Information 3 — Note that this is simply a helpful discretization of a continuous spectrum. Reproduced from Starck & Ricklefs (1998). [file peerj-09-12160-s003.docx]

|  | Plumage | Eyes | Nest Attendance | Parental Care |
| --- | --- | --- | --- | --- |
| Precocial-1 | Contour feathers |  |  | None |
| Precocial-2 |  |  |  | Brooding |
| Precocial-3 |  | Open | Leave | Food showing |
| Precocial-4 |  |  |  |  |
| Semiprecocial | Down |  | Nest Area |  |
| Semialtricial-1 |  |  |  | Parental Feeding |
| Semialtricial-2 |  | closed | Stay |  |
| Altricial | None |  |  |  |
