# Supplementary material for: A histological survey of avian post-natal skeletal ontogeny
Source: PeerJ. 2021 Oct 1;9:e12160. doi: 10.7717/peerj.12160 (PMC8489414; doi:10.7717/peerj.12160)
Supplement: Supplemental Information 4 [file peerj-09-12160-s004.docx]

| **Taxon** | **Specimen Number** | **Growth Stage & Sex (if identifiable)** | **Mass** |
| --- | --- | --- | --- |
| Ostrich  (*Struthio camelus)* | MVZ190729 | neonate | 802 |
| California quail  (*Callipepla californica*) | MVZ190745 | neonate | 7.2 |
| California quail  (*Callipepla californica*) | MVZ190751 | neonate | 4.9 |
| California quail  (*Callipepla californica*) | MVZ190743 | neonate | 7.7 |
| California quail  (*Callipepla californica*) | MVZ190744 | neonate | 7.3 |
| California quail  (*Callipepla californica*) | MVZ190750 | neonate(F) | 10.3 |
| California quail  (*Callipepla californica*) | MVZ190753 | neonate | 6.1 |
| California quail  (*Callipepla californica*) | MVZ190757 | neonate(M) | 7.1 |
| California quail  (*Callipepla californica*) | MVZ190759 | neonate | 7.4 |
| California quail  (*Callipepla californica*) | MVZ190749 | adult(M) | 210 |
| California quail  (*Callipepla californica*) | MVZ190762 | adult(M) | 159.7 |
| Wild turkey  (*Meleagris gallopavo*) | MVZ190764 | neonate(M) | 42.5 |
| Wild turkey  (*Meleagris gallopavo*) | MVZ190763 | adult(F) | 4466 |
| Anna’s Hummingbird  (*Calypte anna*) | MVZ190807 | adult(F) | 3.7 |
| Mourning dove  (*Zenaida macroura*) | MVZ190775 | adult(M) | 188.8 |
| Western gull  (*Larus occidentalis*) | MVZ190824 | neonate | 45.4 |
| Western gull  (*Larus occidentalis*) | MVZ190822 | neonate | 37.1 |
| Western gull  (*Larus occidentalis*) | MVZ190829 | adult(F) | 695 |
| Western gull  (*Larus occidentalis*) | MVZ190831 | adult(F) | 814 |
| American kestrel  (*Falco sparverius*) | MVZ190887 | neonate | 7.0 |
| American kestrel  (*Falco sparverius*) | MVZ190890 | neonate(M) | 7.7 |
| American kestrel  (*Falco sparverius*) | MVZ190892 | adult(M) | 78 |
| American kestrel  (*Falco sparverius*) | MVZ190885 | adult(F) | 106.4 |
| Green-cheeked conure  (*Pyrrhura molinae*) | MVZ190895 | neonate | 2.6 |
| Green-cheeked conure  (*Pyrrhura molinae*) | MVZ190917 | adult | 65.7 |
| Western scrub jay  (*Aphelocoma californica*) | MVZ190927 | neonate(M) | 14.1 |
| House finch  (*Haemorhous mexicanus*) | MVZ190969 | neonate | 3.2 |
| House finch  (*Haemorhous mexicanus*) | MVZ190993 | adult(F) | 14.4 |
| Great-horned owl  (*Bubo virginianus*) | MVZ190883 | adult(F) | 1217 |
| Barn owl  (*Tyto alba*) | MVZ190877 | adult | 583 |
| Barn owl  (*Tyto alba*) | MVZ190872 | adult | 465 |
| White-tailed kite  (*Elanus leucurus*) | MVZ190861 | adult(M) | 315 |
| Red-tailed hawk  (*Buteo jamaicensis*) | MVZ190852 | adult(M) | 1072 |
| Red-tailed hawk  (*Buteo jamaicensis*) | MVZ190855 | adult(F) | 863 |
