# Supplementary figures and images for: A histological survey of avian post-natal skeletal ontogeny
Source: PeerJ. 2021 Oct 1;9:e12160. doi: 10.7717/peerj.12160 (PMC8489414; doi:10.7717/peerj.12160)

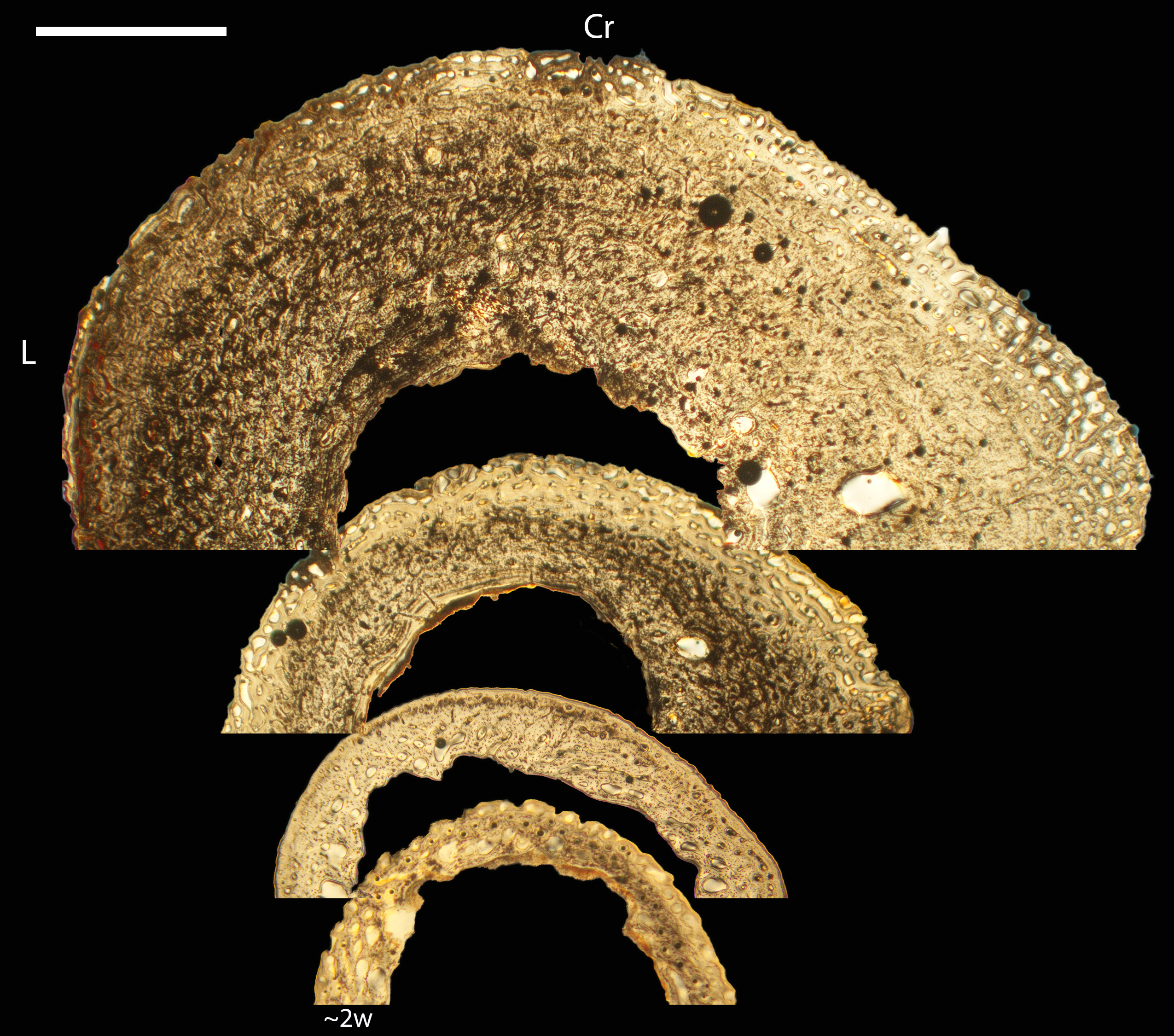

Supplement: Supplemental Information 5 — ~2w = approximately 2 weeks old, MVZ190729; other specimens from smallest to largest: MVZ190727, MVZ190731, MVZ190732. Cr, cranial; L, Lateral. Scale bar equals 1,000 µm [file peerj-09-12160-s005.jpg]

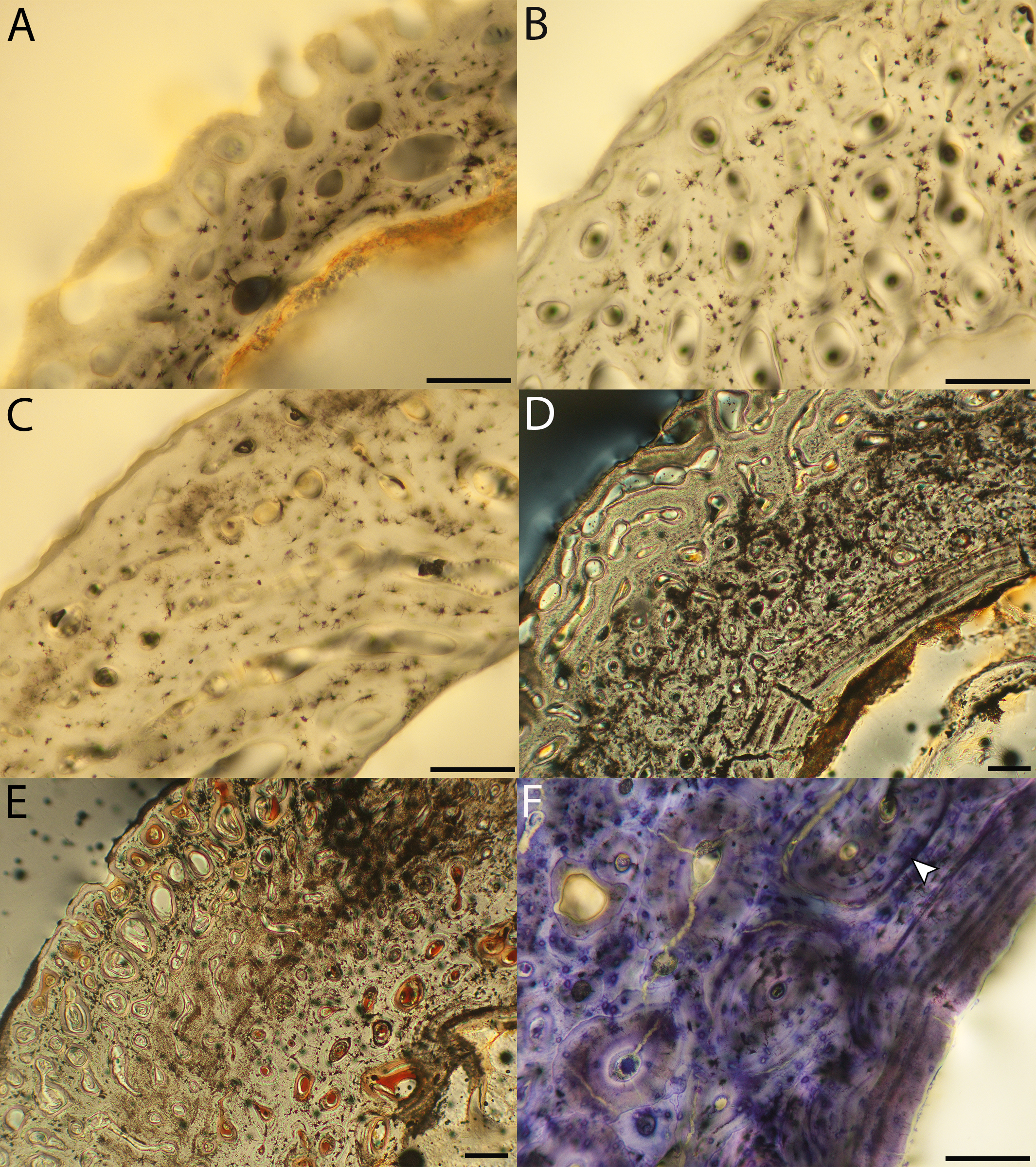

Supplement: Supplemental Information 6 — (A) Humerus of approximately 2-week-old chick (MVZ190729) showing vascular canals nearer the endosteal surface are larger and more irregularly shaped than those towards the periosteal surface. The latter were in the early stages of primary osteon formation. There is an abundance of mature osteocytes within the neonate cortex. Plane light, 200×. (B) Humerus of MVZ190733 with a cortex of woven bone similar to the neonate, but with a thicker cortex and a periosteal vascular canals fully closed-off from to the outside. Plane light, 200×. (C) Humerus of MVZ190727 with characteristics similar to B. Plane light, 200×. (D) Humerus of MVZ190731 showing densely-packed, early-stage longitudinal primary osteons within a woven matrix, forming incipient fibrolamellar bone. This is especially concentrated in the endosteal half of the cortex. Plane light, 100×. (E) Humerus of MVZ190734 with characteristics similar to D. Plane light, 100×. (F) Humerus of MVZ190732,with an incipient inner circumferential layer and sparsely scattered secondary osteons (arrow). Plane light, toluidine blue stain, 100×. In all images the periosteal surface is oriented to top/left and the endosteal to the bottom/right. Scale bare equals 100 µm. [file peerj-09-12160-s006.jpg]

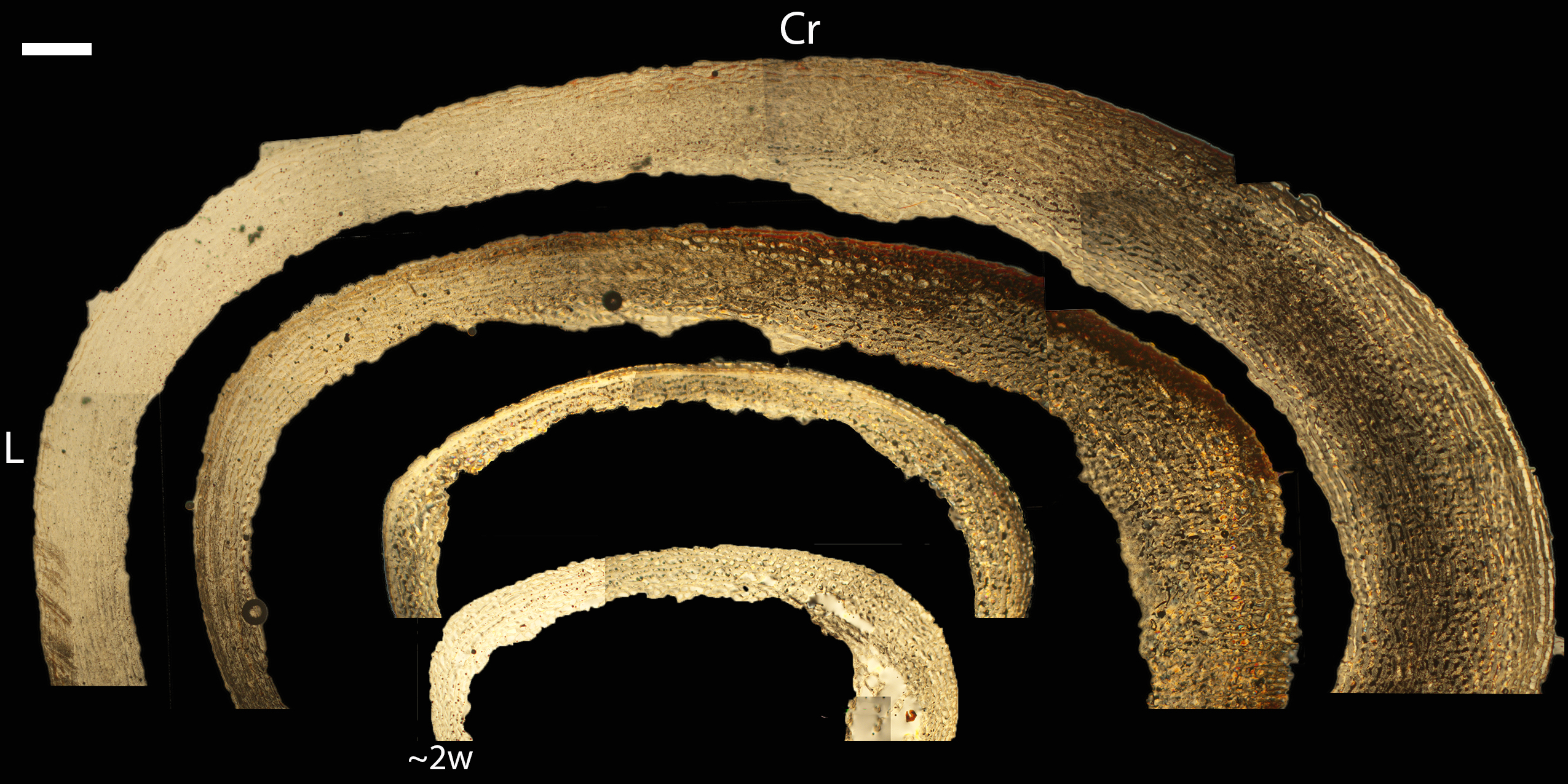

Supplement: Supplemental Information 7 — ~2w = approximately 2 weeks old, MVZ190729; other specimens from smallest to largest: JAA74, MVZ190731, MVZ190732. Cr, cranial; L, Lateral. Scale bar equals 1,000 µm. [file peerj-09-12160-s007.jpg]

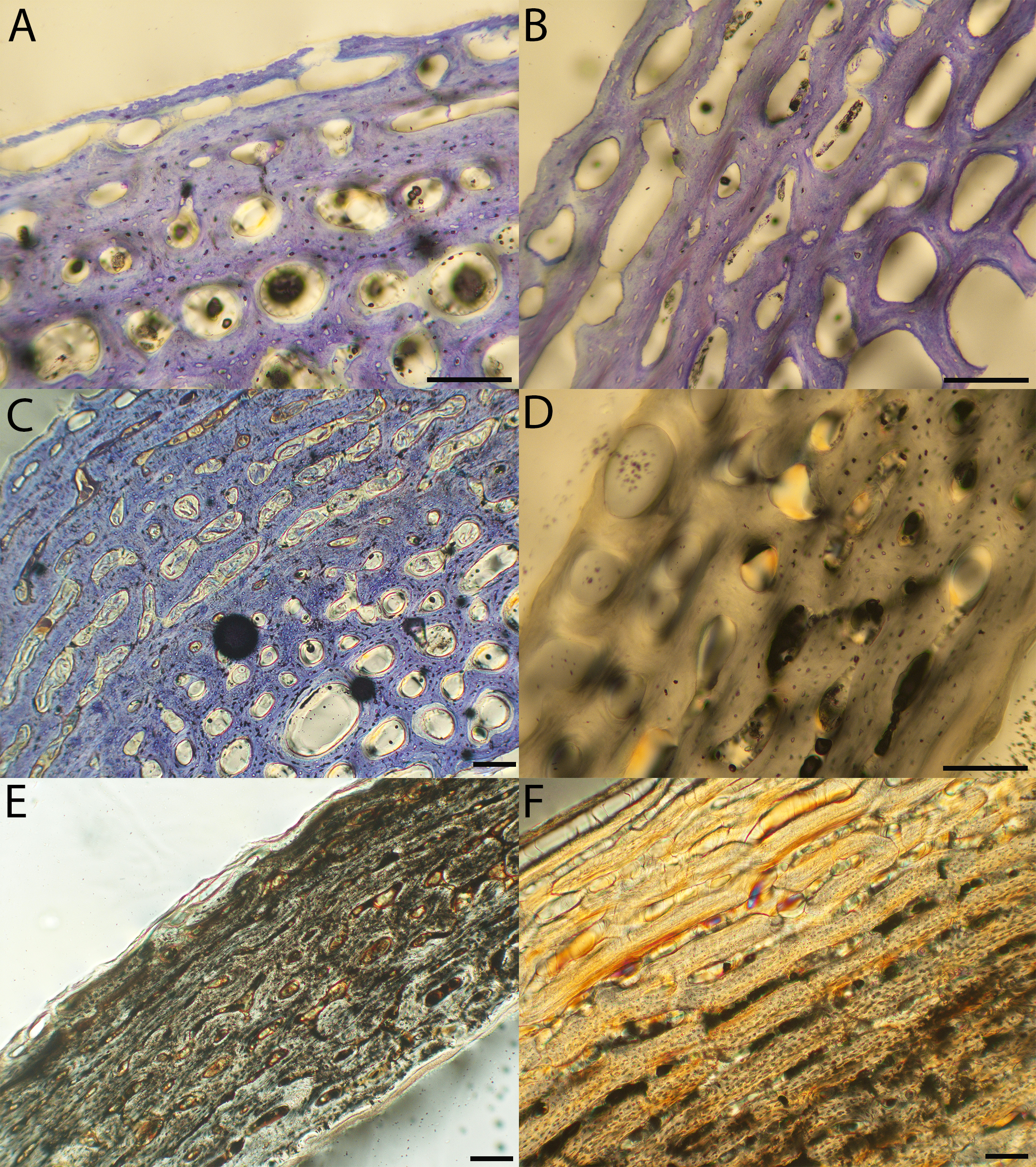

Supplement: Supplemental Information 8 — (A) Femur of approximately 2-week-old chick (MVZ190733) composed of disorganized woven bone with large vascular canals, though mature enough that those at the periosteal border no longer communicate with the exterior. Plane light, toluidine blue stain, 200×. (B) Femur of MVZ190729. Plane light, toluidine blue stain, 200×. (C) Femur of MVZ190734 showing increased organization of the developing bone with the incipient fibrolamellar bone concentrated in periosteal half of the cortex. Plane light, toluidine blue stain, 100×. (D) Femur of MVZ190727 composed of woven bone with newly-formed primary osteons and incipient primary osteons. Plane light, 200×. (E) Femur of MVZ190731 showing increasingly mature bone with substantially lower vascular area than earlier growth stages and numerous primary osteons. Plane light, 40×. (F) Femur of MVZ190732 with a mature fibrolamellar complex, charachterized by a predominance of circumferential canals in the endosteal half of the cortex. Plane light, 100×. In all images the periosteal surface is oriented to top/left and the endosteal to the bottom/right. Scale bare equals 100 µm. [file peerj-09-12160-s008.jpg]

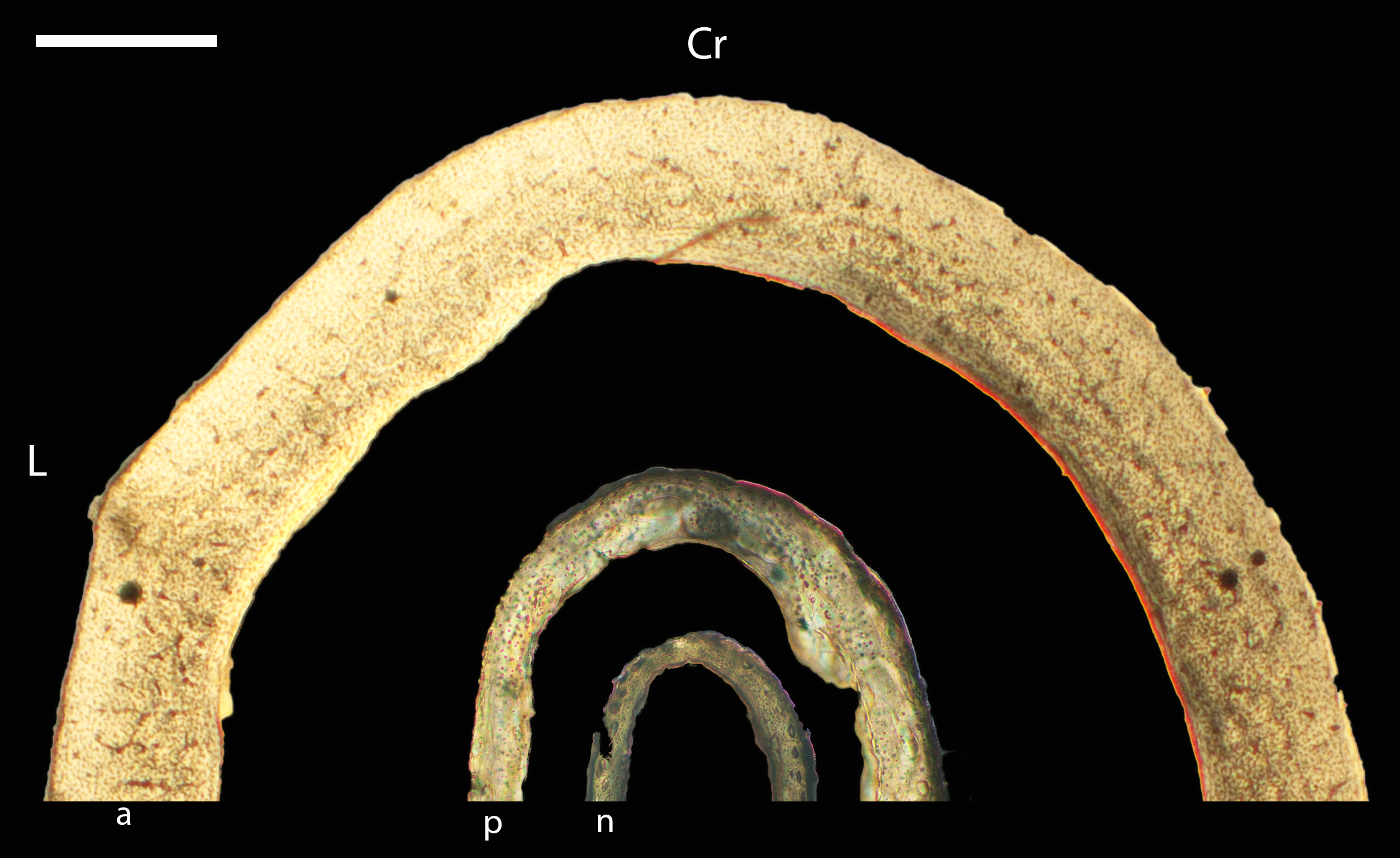

Supplement: Supplemental Information 9 — n = neonate, MVZ190743; p = pin-feathered chick, MVZ190761; a = adult, MVZ190749. Cr, cranial; L, Lateral. Scale bar equals 500µm. [file peerj-09-12160-s009.jpg]

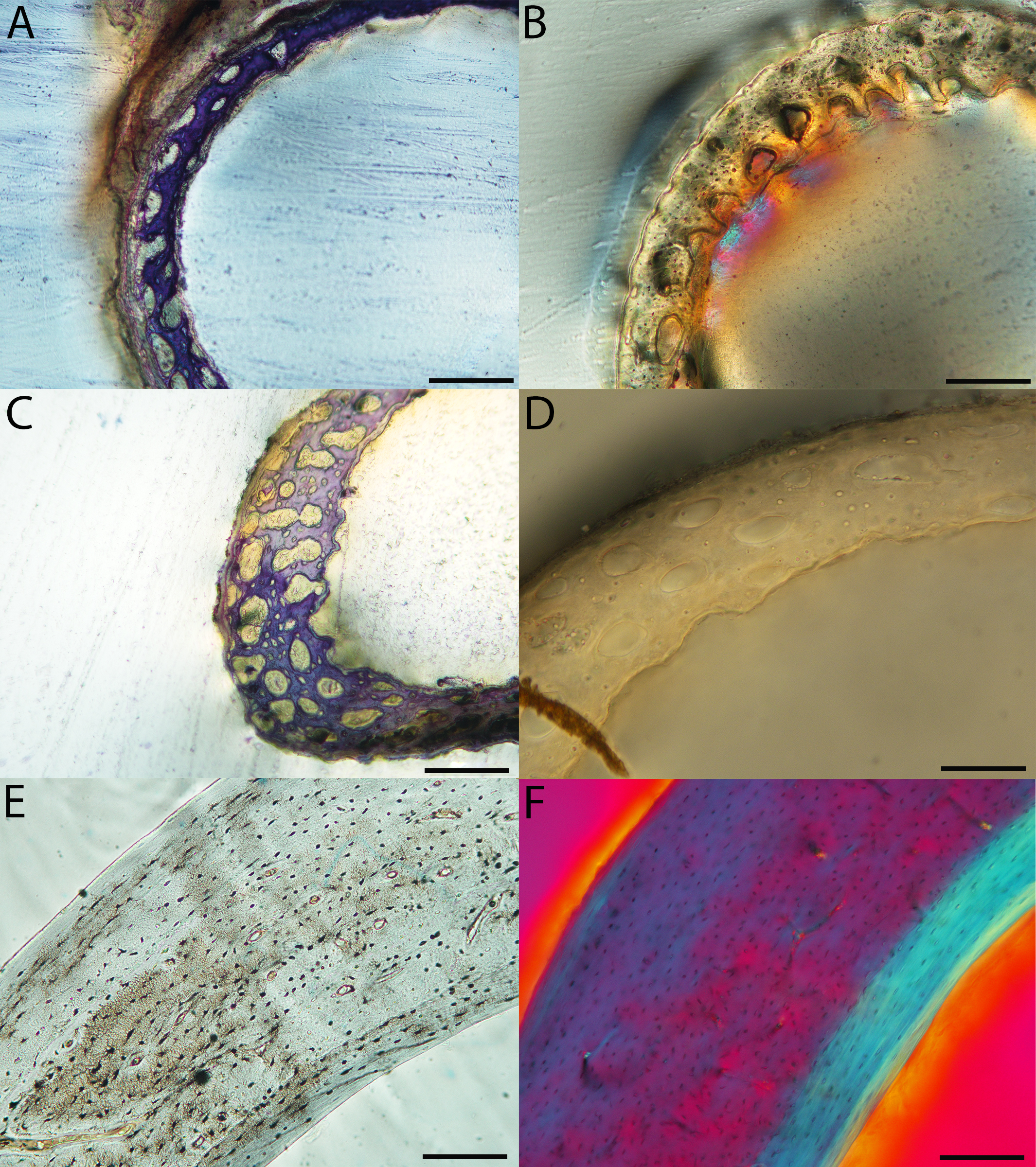

Supplement: Supplemental Information 10 — (A) Neonate humerus (MVZ190751) composed of woven bone with irregulary-oriented vascular canals. Plane light, toluidine blue stain, 200×; (B) Neonate humerus (MVZ190750) of woven bone with large vascular canals. Plane light, 200×. (C) Neonate humerus (MVZ190745), showing thicker cortex of woven bone and irregularly-oriented canals. Plane light, toluidine blue stain, 200×. (D) Humerus of a pin-feathered chick (MVZ190761) showing woven bone with a smooth periosteal edge and organized orientation of vascular canals (mostly longitudinal). Plane light, 200×. (E) Adult humerus (MVZ190762) with a weakly-formed fibrolamellar complex. Plane light, 100×. (F) Adult humerus (MVZ190749) showing a thick OCL of parallel-fibered bone adjacent to a mid-cortical layer of woven bone with simple vascular canals. Polarized light, 100×. In all images the periosteal surface is oriented to top/left and the endosteal to the bottom/right. Scale bare equals 100 µm. [file peerj-09-12160-s010.jpg]

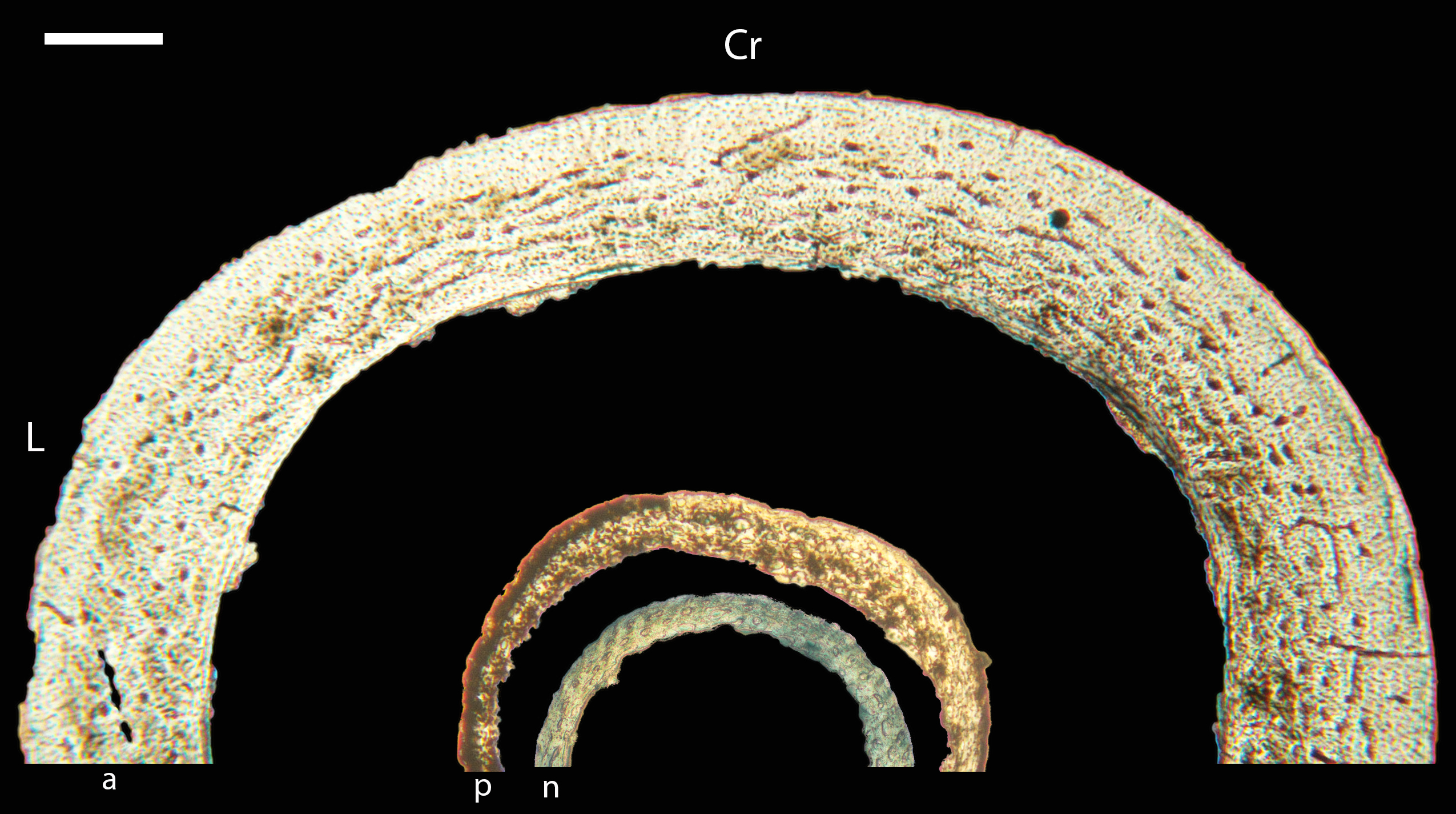

Supplement: Supplemental Information 11 — n = neonate, MVZ190745; p = pin-feathered chick, MVZ190761; a = adult, MVZ190762. Cr, cranial; L, Lateral. Scale bar equals 250 µm. [file peerj-09-12160-s011.jpg]

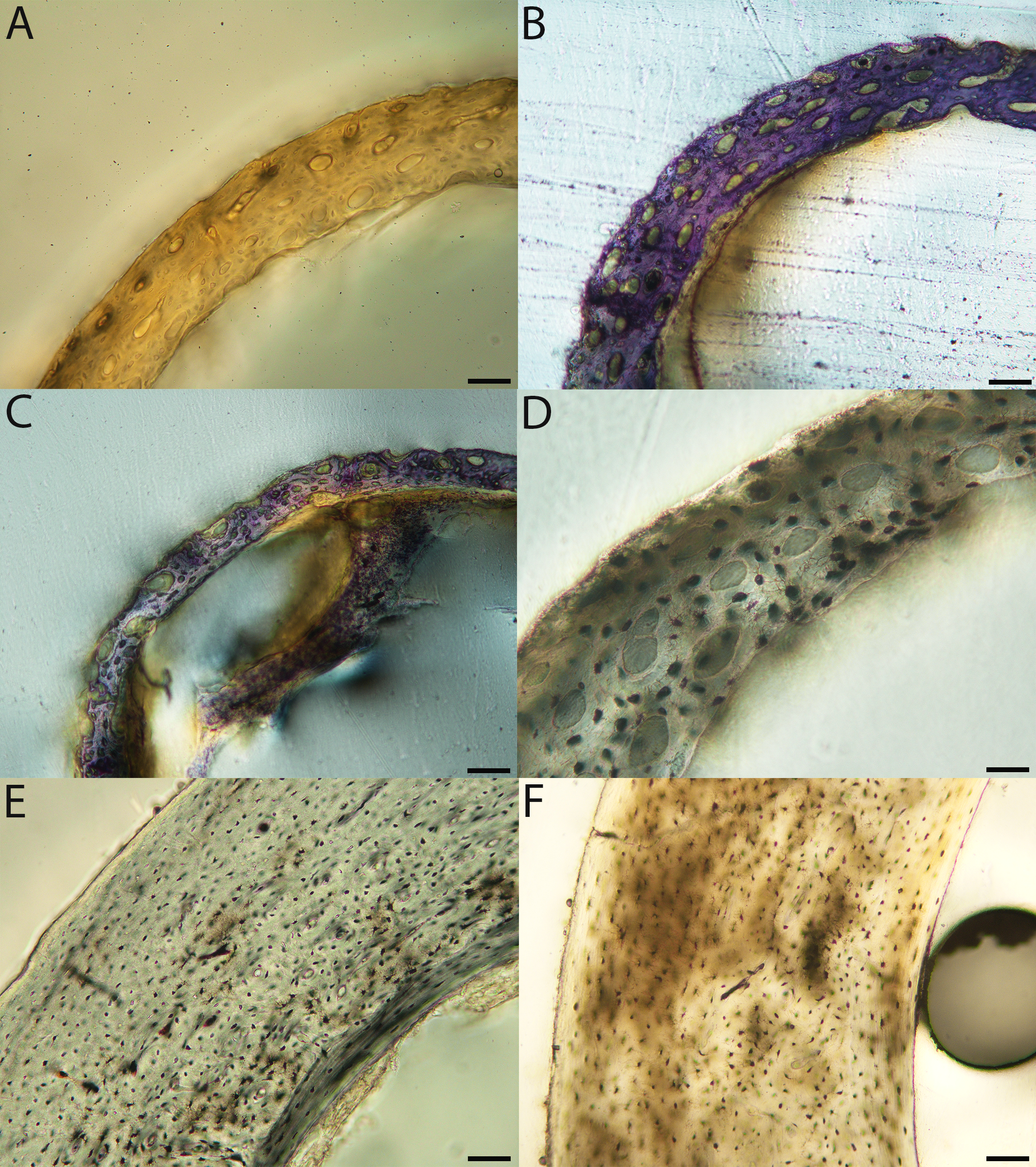

Supplement: Supplemental Information 12 — (A) Neonate femur (MVZ190743) composed of incipient fibrolamellar bone. Plane light, 200×. (B) Neonate femur (MVZ190751) showing woven bone and variously oriented vascular canals. Plane light, toluidine blue, 200×. (C) Neonate femur (MVZ190750) with a very thin cortex composed of woven bone. Plane light, toluidine blue stain, 200×. (D) Femur of a pin-feathered chick (MVZ190761) with woven bone and incipient osteons. Plane light, 200×. (E) Adult femur (MVZ190762) showing a strongly-developted ICL adjacent to a region of weakly-fibrolamellar bone; a thick parallel-fibered region is located periosteally. Plane light, 100×. (F) Adult femur (MVZ190749) with characteristics similar to E but showing a mid-cortical region of woven bone with simple vascular canals instead of fibrolamellar bone (though both individuals had regions of both in the femur). Plane light, 100×. In all images the periosteal surface is oriented to top/left and the endosteal to the bottom/right. Scale bare equals 50 µm. [file peerj-09-12160-s012.jpg]

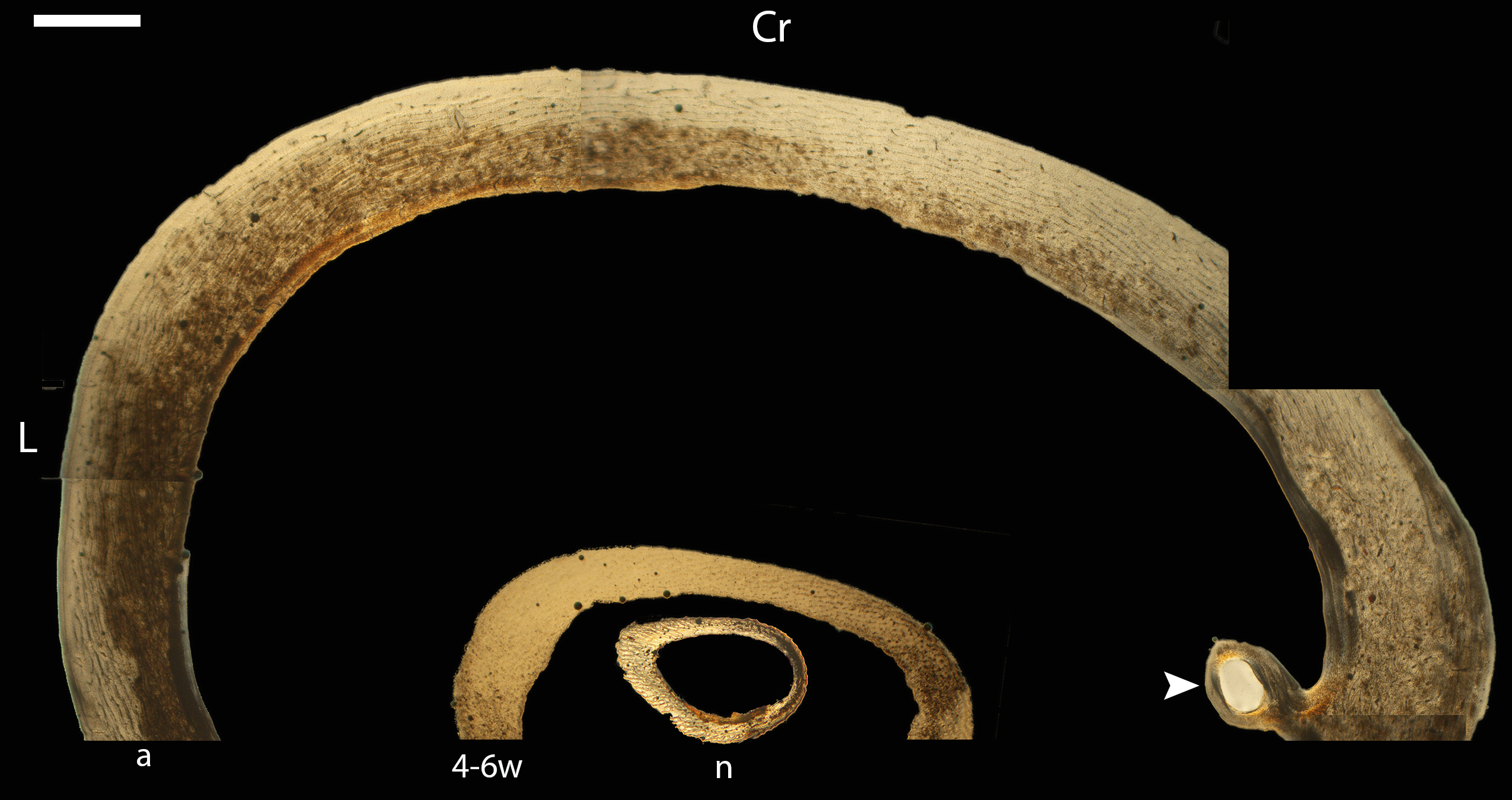

Supplement: Supplemental Information 13 — n = neonate, MVZ190764; 4–6 = 4-to 6-week-old chick, MVZ190765; a = adult, MVZ190763. Arrow indicates loop of ICL, which housed the vessel penetrating the bone in via nutrient formation (see Fig. 10E). Cr, cranial; L, Lateral. Scale bar equals 1,000 µm. [file peerj-09-12160-s013.jpg]

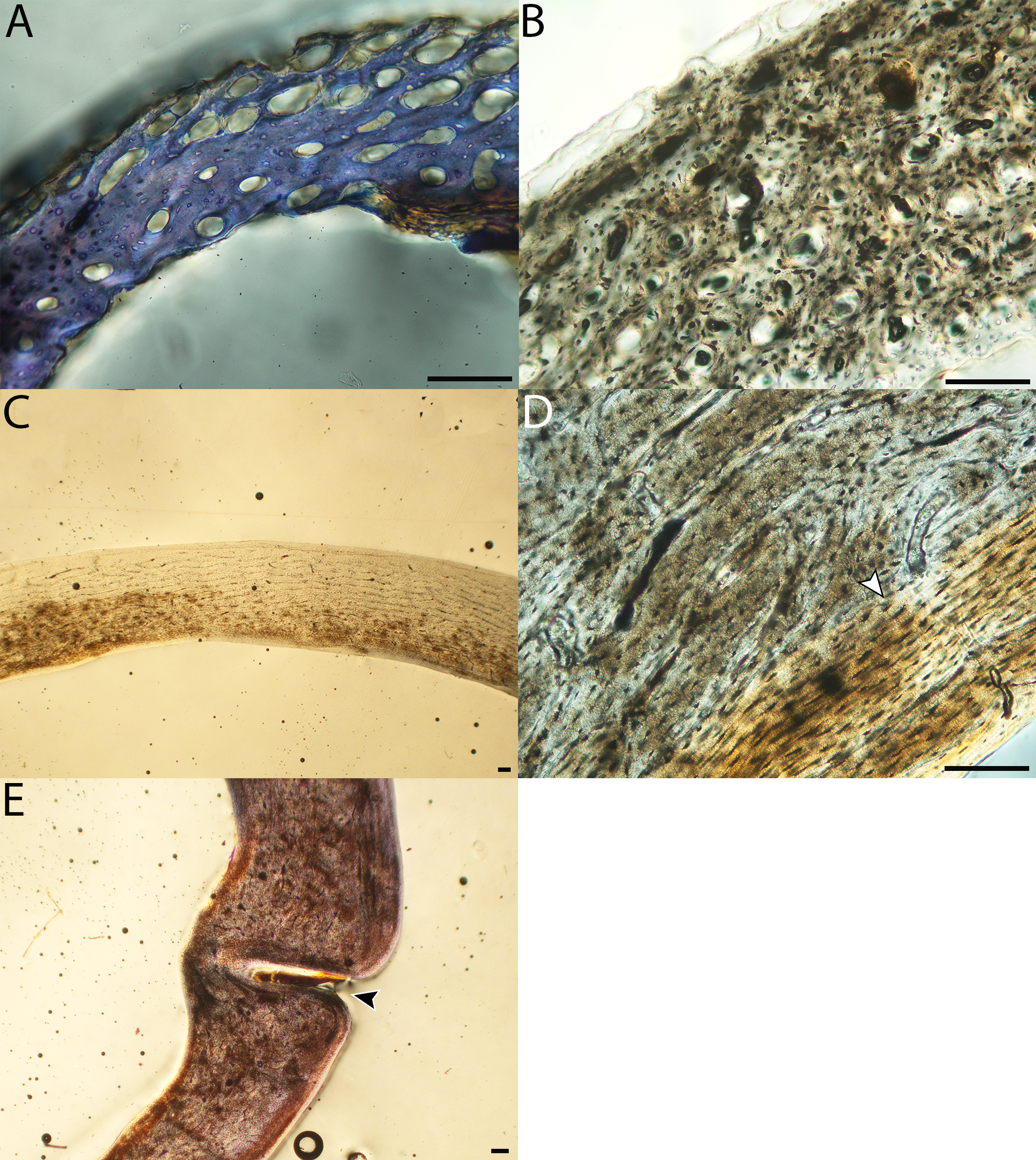

Supplement: Supplemental Information 14 — (A) Neonate humerus (MVZ190764) showing disorganized woven bone with large, irregular vascular canals. Plane light, toluidine blue stain, 200x. (B) Humerus of a 4-to 6-week-old chick (MVZ190765), composed of woven bone with developing primary osteons; levels of vascularity remain high. Plane light, 200×. (C) Adult humerus (MVZ190763), showing OCL and ICL and a predominance of laminar vascular spaces. Plane light, 40×. (D) Adult humerus (MVZ190763) with mature fibrolamellar bone; arrow indicates the parallel-fibered ICL. Plane light, 200×. (E) Adult humerus (MVZ190763) showing the mediocaudal margin of the cortex with a predominance of longitudinally-oriented vascular canals; arrow indicates the nutrient foramen penetrating the bone. Plane light, toluidine blue stain, 40×. In all images the periosteal surface is oriented to top/left and the endosteal to the bottom/right. In all images the periosteal surface is oriented to top/left and the endosteal to the bottom/right. Scale bare equals 100 µm. [file peerj-09-12160-s014.jpg]

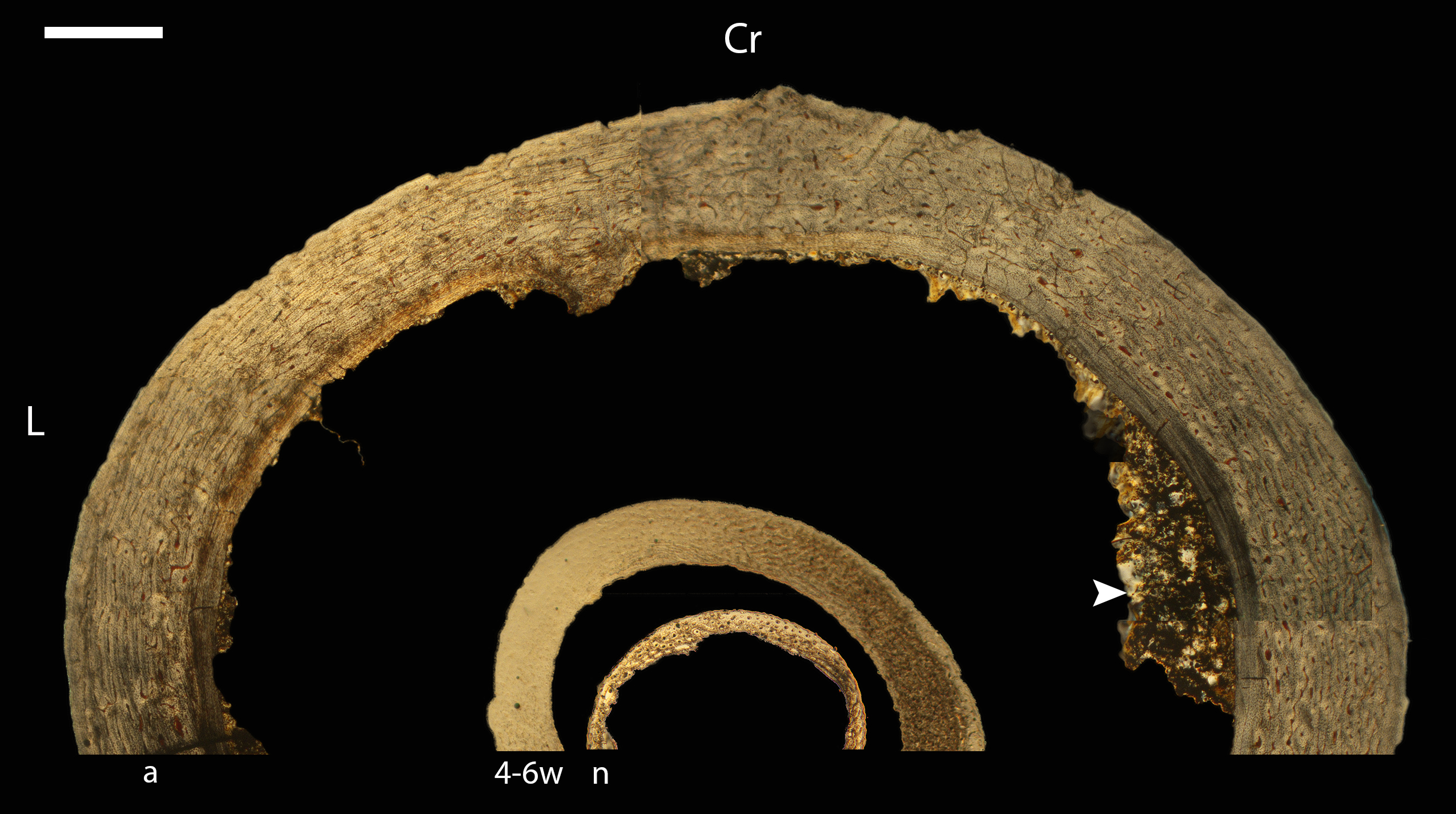

Supplement: Supplemental Information 15 — n = neonate, MVZ190764; 4–6 = 4-to 6-week-old chick, MVZ190765; a = adult, MVZ190763. Note medullary bone lining endosteal surface of the adult femur (white arrow). Cr, cranial; L, Lateral. Scale bar equals 1,000 µm. [file peerj-09-12160-s015.jpg]

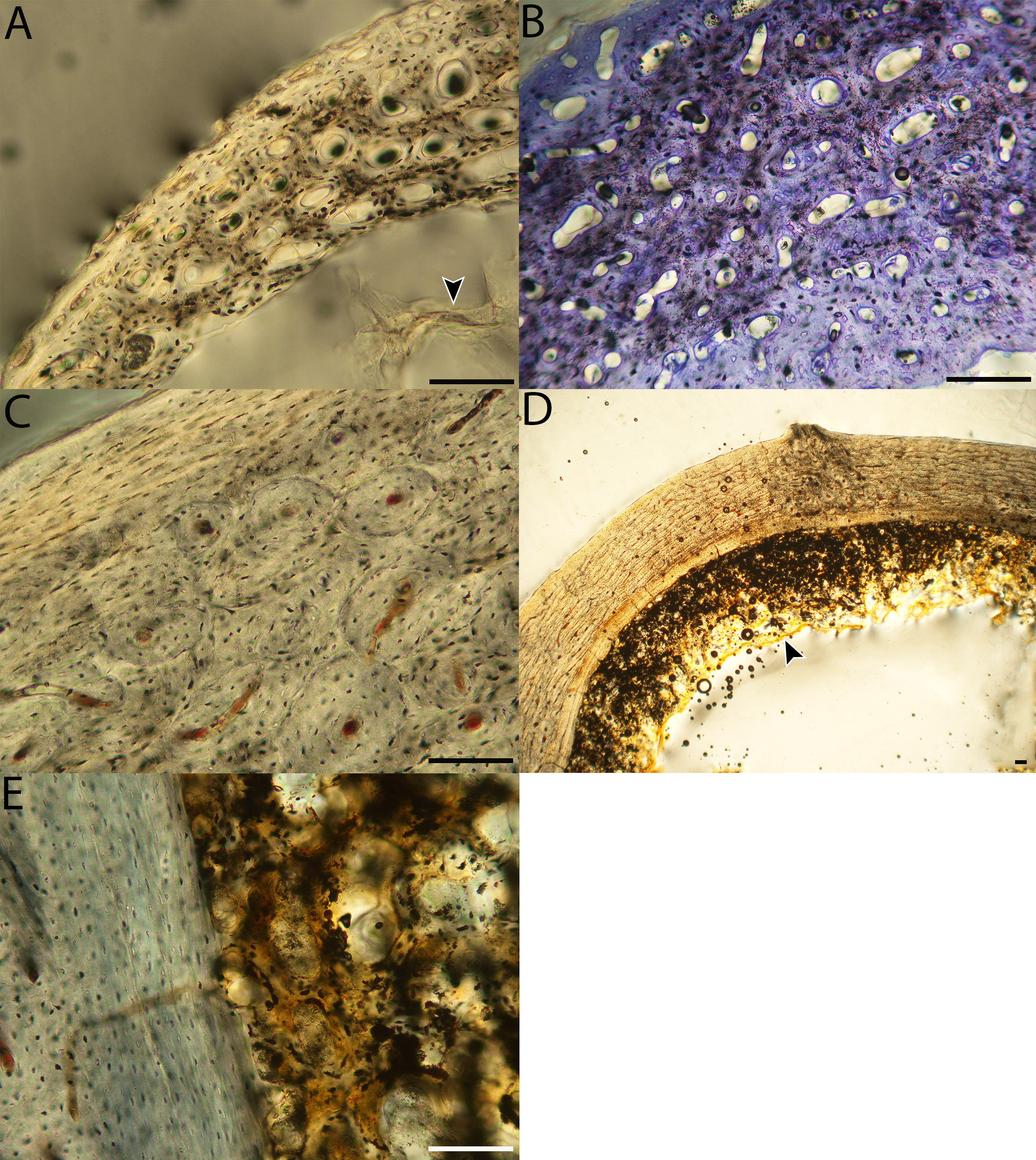

Supplement: Supplemental Information 16 — (A) Neonate femur (MVZ190764) of woven bone with large, longitudinally-oriented vascular canals (some, visible here, are incipient primary osteons); arrow indicates part of the endosteum seen in the endosteal cavity of this specimen. Plane light, 200×. (B) Femur of a 4-to 6-week-old chick (MVZ190765) showing the development of a fibrolamellar complex, with woven bone with incipient primary osteons. Plane light, toluidine blue stain, 200×. (C) Adult femur (MVZ190763). Plane light, 200×. (D) Adult femur (MVZ190763), showing circumferentially-oriented vascular canals and medullary bone (arrow). Plane light, 40×. (E) Adult femur (MVZ190763), close-up of medullary bone (right) and parallel-fibered bone of the ICL (left). Note localized Haversian system in the adult femur; this was one of very few areas of secondary growth observed in this study. Plane light, 200×. In all images the periosteal surface is oriented to top/left and the endosteal to the bottom/right. Scale bare equals 100 µm. [file peerj-09-12160-s016.jpg]

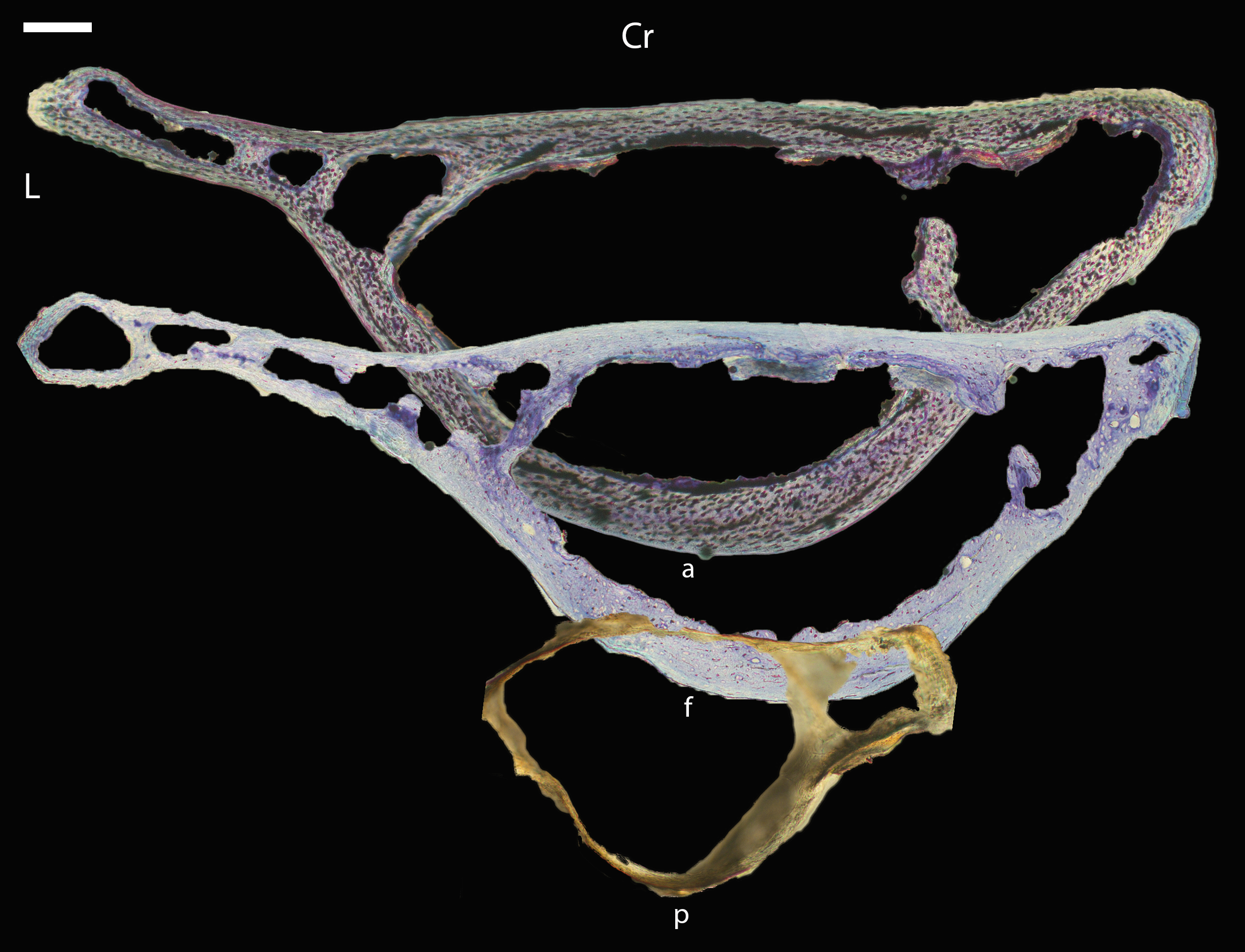

Supplement: Supplemental Information 17 — p = pin-feathered chick, MVZ190799; f = fledgling, MVZ190802; a = adult, MVZ190807. Cr, cranial; L, lateral. Scale bar equals 100 µm. [file peerj-09-12160-s017.jpg]

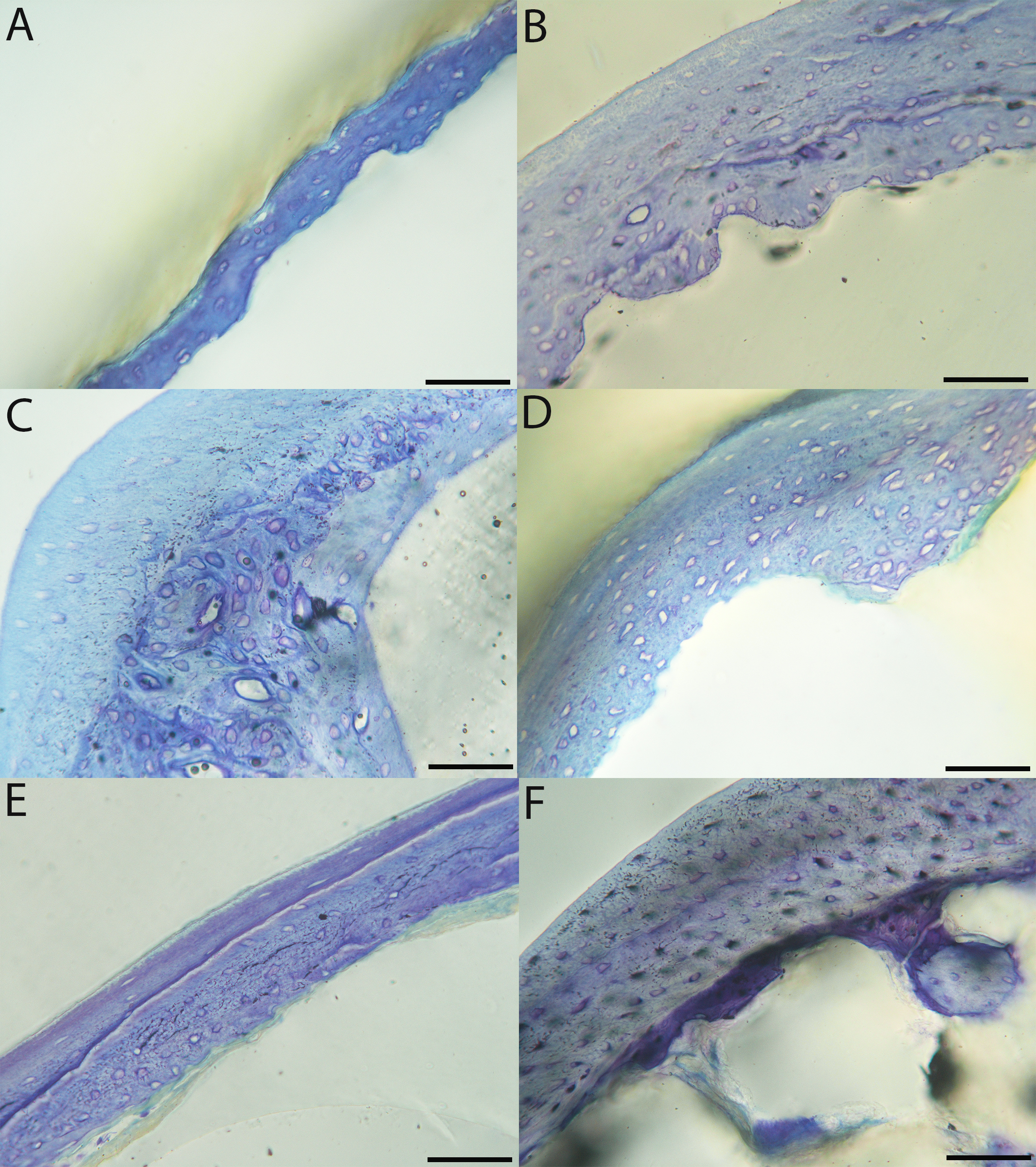

Supplement: Supplemental Information 18 — (A) Humerus of a pin-feathered chick (MVZ190799) showing a very thin cortex of woven bone. Almost no vascular canals were fully enclosed with bone. Plane light, toluidine blue stain, 400×. (B) Humerus of fledgling chick (MVZ190802) showing cortex composed periosteally of parallel-fibered bone and endosteally of woven bone. Note scalloped endosteal margin, indicative of the presence of vascular structures. Plane light, toluidine blue stain, 400×. (C) Humerus of a sub-adult (MVZ190803) showing the cortex of the developing bicipital crest with a very thick endosteal layer of woven adjacent to the parallel-fibered bone of the cortex. Plane light, toluidine blue stain, 400×. (D) Humerus of a sub-adult (MVZ190800) showing a transitional region of the cortex with a clear outer layer of parallel-fibered bone (with low osteocyte lacuna density) and an inner layer of woven bone transitioning to parallel-fibered. Plane light, toluidine blue stain, 200×. (E) Humerus of a sub-adult (MVZ190808) along the diaphyseal cortex, composed of parallel-fibered bone with low-cellularity in the periosteal portion and woven bone transitioning to parallel-fibered endosteally. There appears to be an outer layer of highly organized bone that resembles an OCL, but disappears in the adult. Plane light, toluidine blue stain, 200×. F) Adult humerus (MVZ190807) composed of parallel-fibered bone with higher numbers of osteocyte lacunae than observed in sub-adult individuals. In all images the periosteal surface is oriented to top/left and the endosteal to the bottom/right. Plane light, toluidine blue stain, 200×. Scale bare equals 50 µm. [file peerj-09-12160-s018.jpg]

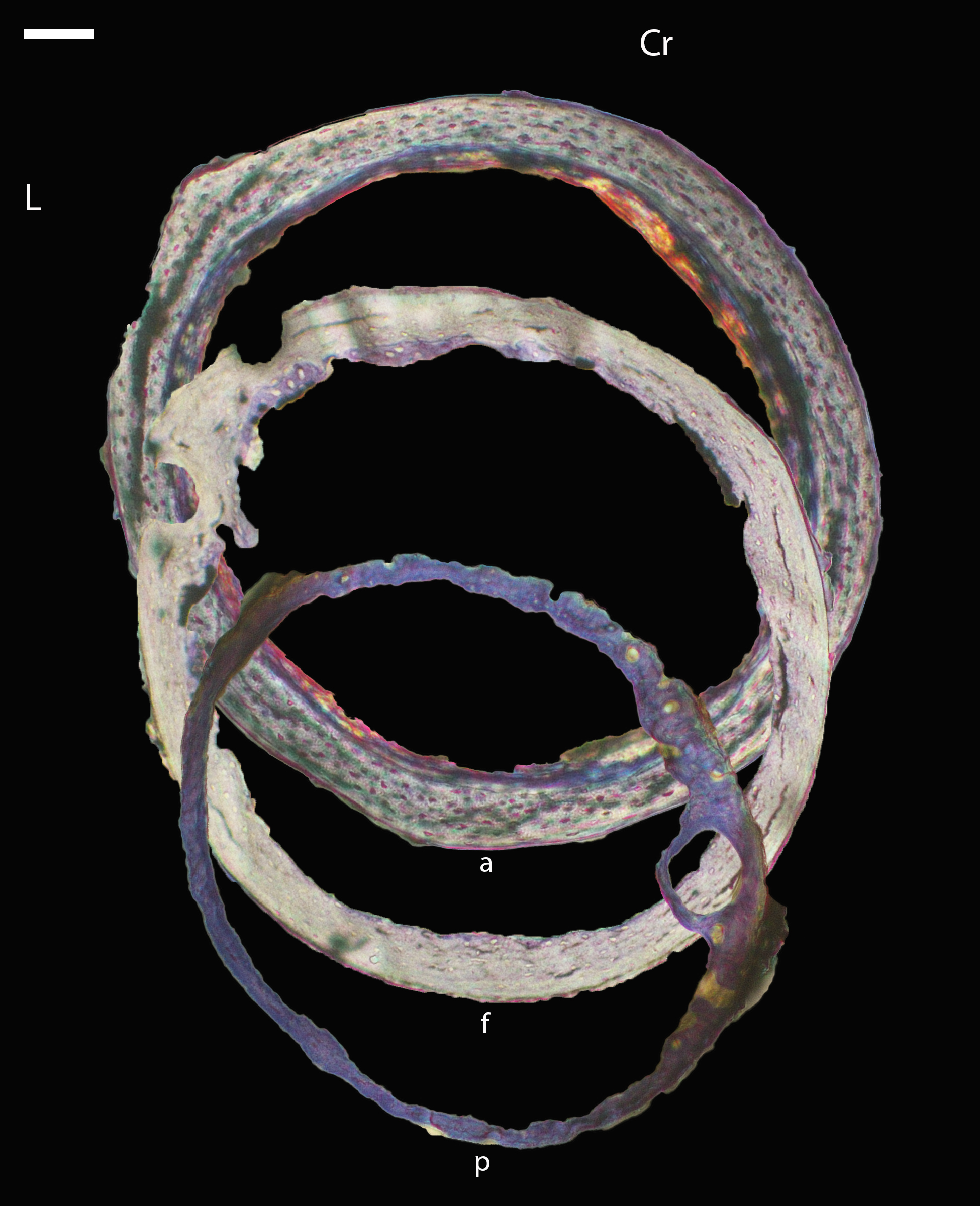

Supplement: Supplemental Information 19 — p = pin-feathered chick, MVZ190799; f = fledgling, MVZ190802; a = adult, MVZ190807. Cr, cranial; L, lateral. Scale bar equals 100 µm. [file peerj-09-12160-s019.jpg]

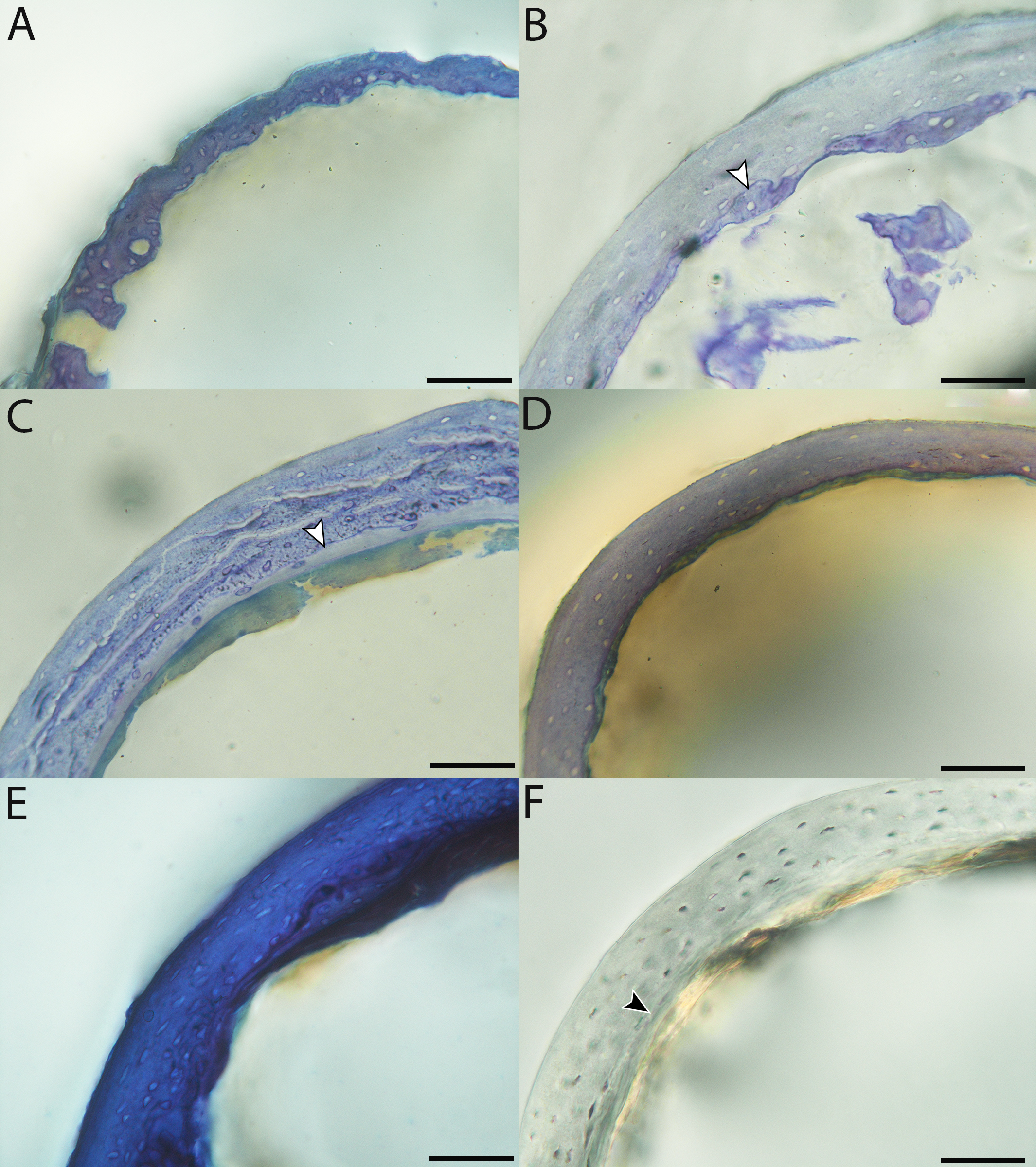

Supplement: Supplemental Information 20 — (A) Femur of a pin-feathered chick (MVZ190799) composed of a thin layer of woven bone. Fully-developed vascular canals are sparse (only two are visible here), but the scalloped peri-and endosteal margins are indicative of the presence of vascular structures not fully enclosed in bone. Plane light, toluidine blue stain, 400×. (B) Femur of a fledgling chick (MVZ190802) showing parallel-fibered bone lined by a thin, irregular layer of chondroid bone (arrow). Plane light, toluidine blue stain, 400×. (C) Femur of a sub-adult (MVZ190803) composed of parallel-fibered bone with sparse osteocyte lacunae. An incipient ICL is present in this individual (arrow). Plane light, toluidine blue stain, 400×. (D) Femur of a sub-adult (MVZ190800) showing parallel-fibered bone with sparse osteocyte lacunae. Plane light, toluidine blue stain, 400×. (E) Femur of a sub-adult (MVZ190808) with characteristics similar to D. Plane light, toluidine blue stain, 400×. (F) Femur of an adult (MVZ190807) composed of highly-cellularized, avascular, parallel-fibered bone. An ICL is also visible (arrow). Plane light, toluidine blue stain, 400×. In all images the periosteal surface is oriented to top/left and the endosteal to the bottom/right. Scale bare equals 50 µm. [file peerj-09-12160-s020.jpg]

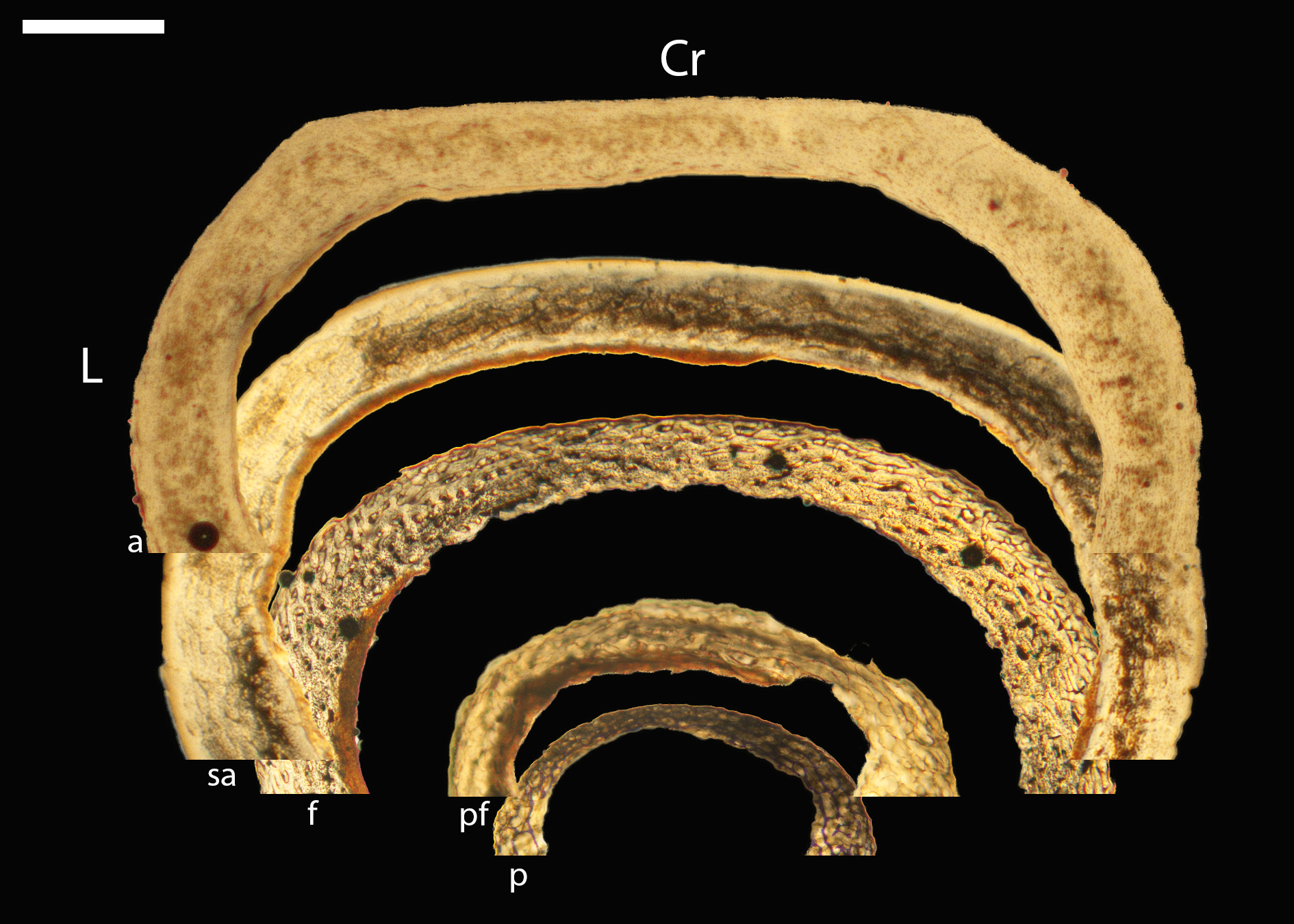

Supplement: Supplemental Information 21 — p = pin-feathered chick, MVZ190778; pf = pre-fledgling chick, MVZ190774; f = fledgling chick, MVZ190779; sa = sub-adult, MVZ190783; a = adult, MVZ190775. Cr, cranial; L, lateral. Scale bar equals 500 µm. [file peerj-09-12160-s021.jpg]

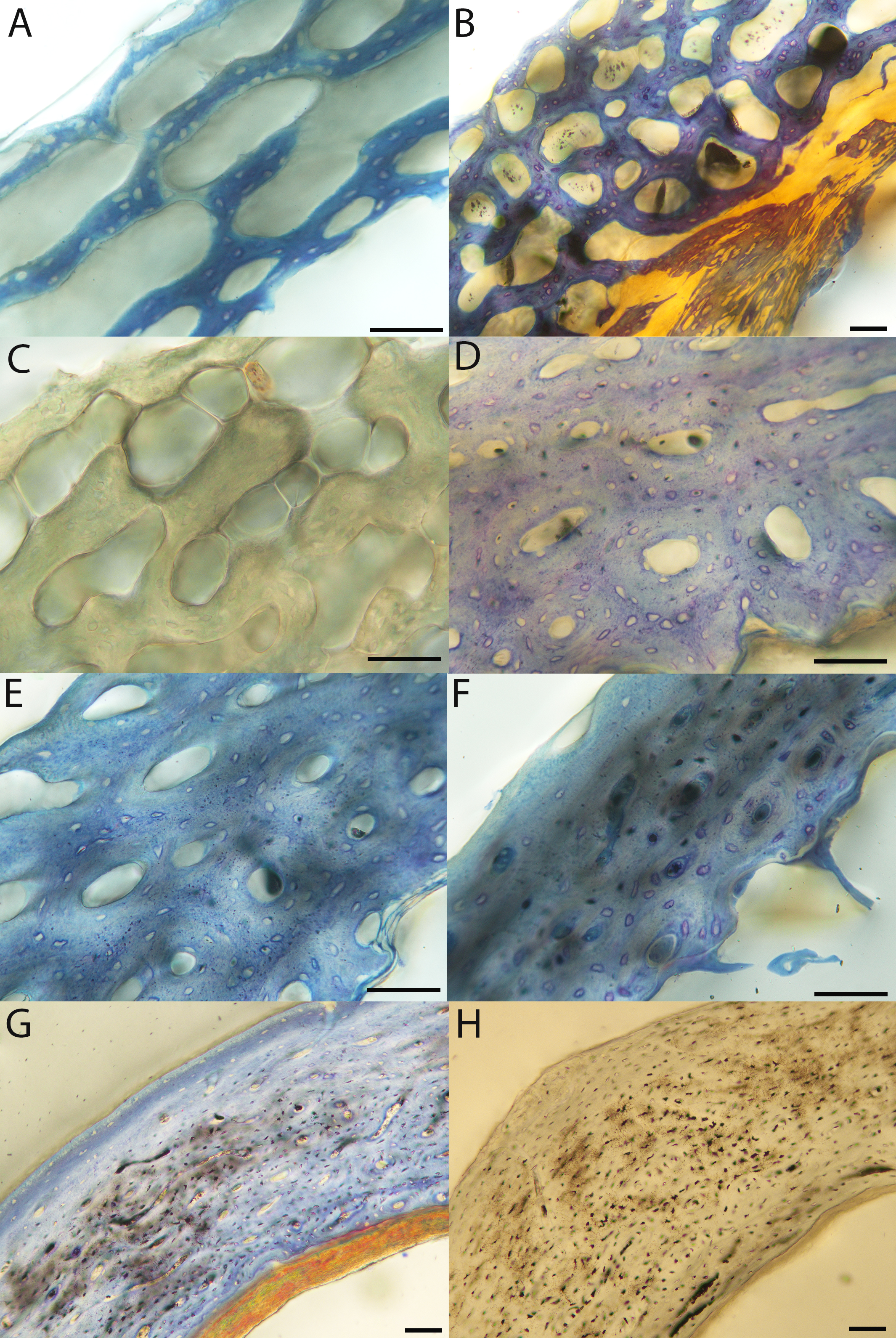

Supplement: Supplemental Information 22 — (A) Humerus of a pin-feathered chick (MVZ190778) with highly porous woven bone. Plain light, toluidine blue stain, 400×. (B) Humerus of a pre-fledgling chick (MVZ190784) showing a thick cortext of woven bone with large, irregular vascular canals and a thick layer of endosteum. Plain light, toluidine blue stain, 200×. (C) Humerus of a pre-fledgling chick (MVZ190774) showing woven bone with irregular vascular canals; these spaces are still large but are becoming smaller as the bone separating them expands. Plain light, 400×. (D) Humerus of a fledgling chick (MVZ190785) showing bone maturing into a fibrolamellar complex with many incipient osteons in a matrix of woven bone. Plane light, toluidine blue stain, 400×. (E) Humerus of a fledgling chick (MVZ190779) with characteristics similar to D. Plane light, toluidine blue stain, 400×. (F) Humerus of a sub-adult (MVZ190780), composed of mature fibrolamellar bone with an incipient an OCL. Plane light, toluidine blue stain, 200x. G) Humerus of a sub-adult (MVZ190783) showing fibrolamellar bone with an incipient OCL; there is also a layer of endosteum. Plane light, toluidine blue stain, 100×. (H) Humerus of an adult (MVZ190775) composed of fibrolamellar bone with very small vascular canals, within a parallel-fibered OCL. Plane light, 100×. In all images the periosteal surface is oriented to top/left and the endosteal to the bottom/right. Scale bare equals 50 µm. [file peerj-09-12160-s022.jpg]

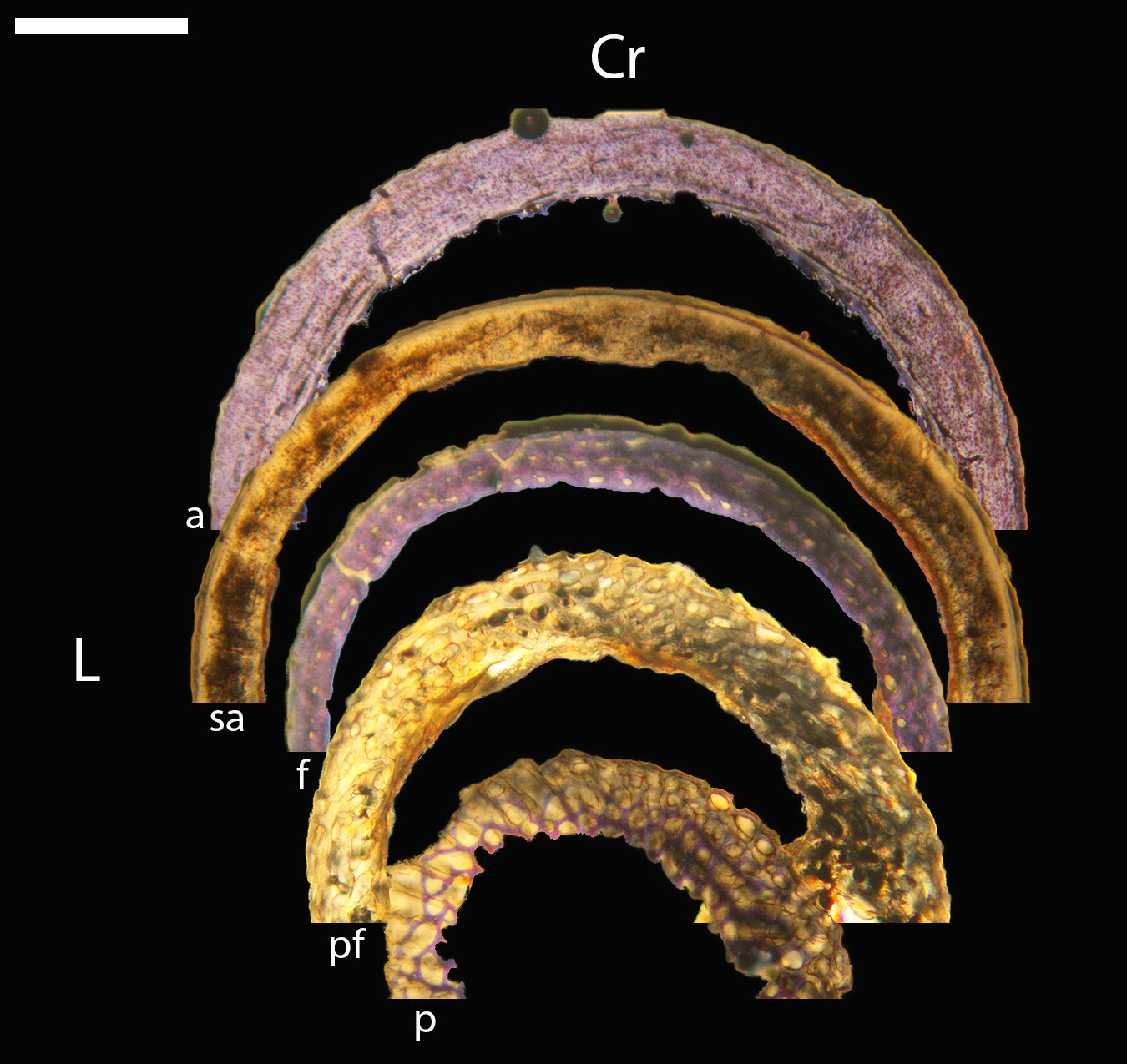

Supplement: Supplemental Information 23 — p = pin-feathered chick, MVZ190778; pf = pre-fledgling chick, MVZ190784; f = fledgling chick, MVZ190779; sa = sub-adult, MVZ190783; a = adult, MVZ190775. Cr, cranial; L, lateral. Scale bar equals 500 µm. [file peerj-09-12160-s023.jpg]

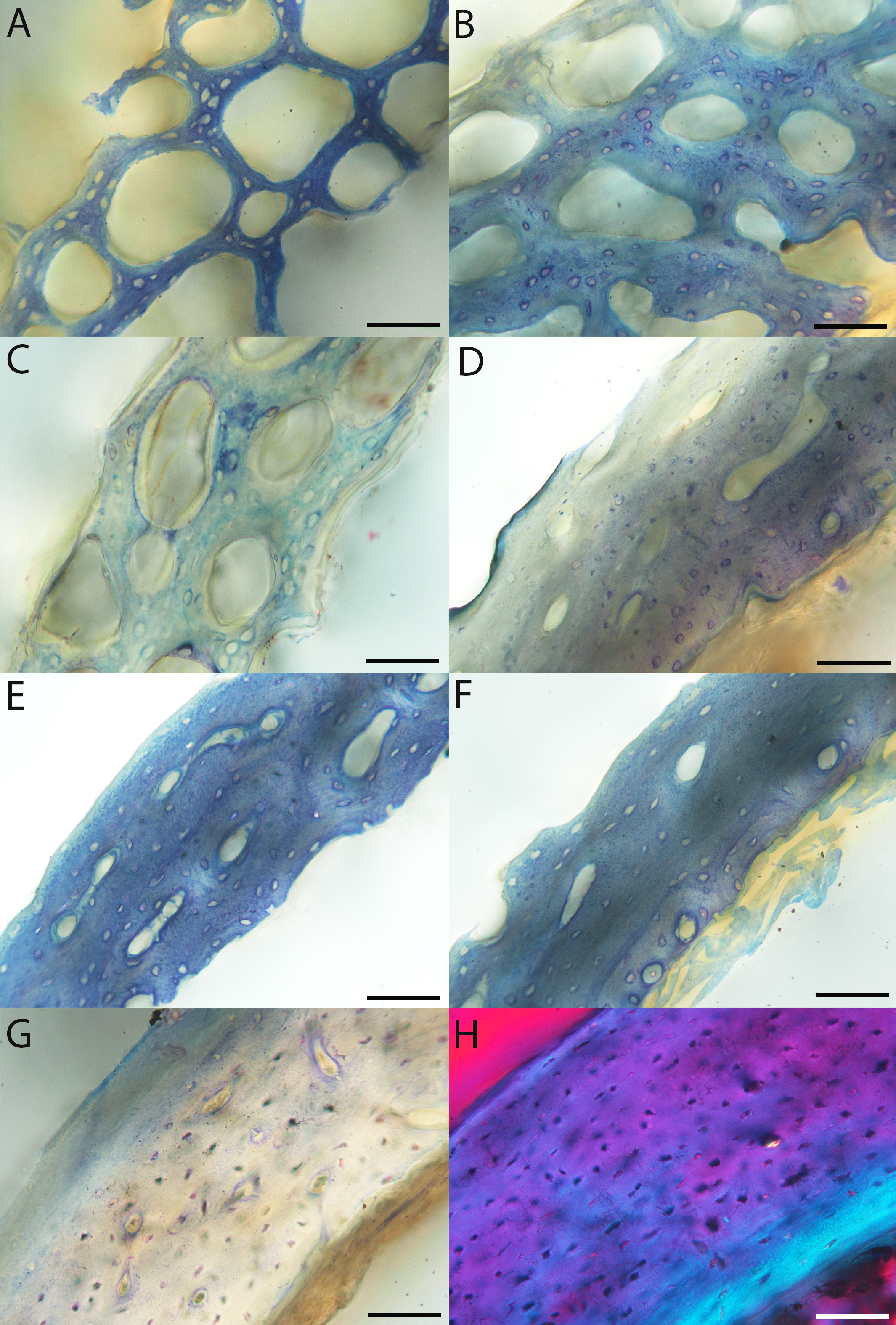

Supplement: Supplemental Information 24 — (A) Femur of a pin-feathered chick (MVZ190778), composed of very thin woven bone demarcating large, irregular vascular canals; most are longitudinal in orientation . Plane light, toluidine blue stain, 200×. (B) Femur of a pre-fledgling chick (MVZ190784), also composed of woven bone but with increased bone deposition and smaller vascular spaces as a consequence. Plane light, toluidine blue stain, 200×. (C) Femur of a pre-fledgling chick (MVZ190774) showing features similar to B. Plane light, toluidine blue stain, 200×. (D) Femur of a fledgling chick (MVZ190785) showing woven bone with incipient primary osteons and smaller vascular canals than the pre-fledgling stage. Plane light, toluidine blue, 200×. (E) Femur of a fledgling chick (MVZ190779) showing a region of fibrolamellar bone with weakly-formed primary osteons. Plane light, toluidine blue, 200×. (F) Femur of the less mature individual classified as “sub-adult” (MVZ190780) showing a thinning cortext composed of woven bone with weakly-formed primary osteons. Plane light, toluidine blue, 200×. (G) Femur of the more mature individual classified as “sub-adult” (MVZ190783) with vascular canals that are small but still numerus; a thin, outer layer resembling an OCL is visible. Plane light, toluidine blue stain, 200×. (H) Femur of an adult (MVZ190775) showing a cortex of parallel-fibered/weakly-woven bone with very few, small, primary vascular canals. An ICL is also visible, as well as a very thin layer possibly representing the OCL. Polarized light, 200×. In all images the periosteal surface is oriented to top/left and the endosteal to the bottom/right. Scale bare equals 50 µm. [file peerj-09-12160-s024.jpg]

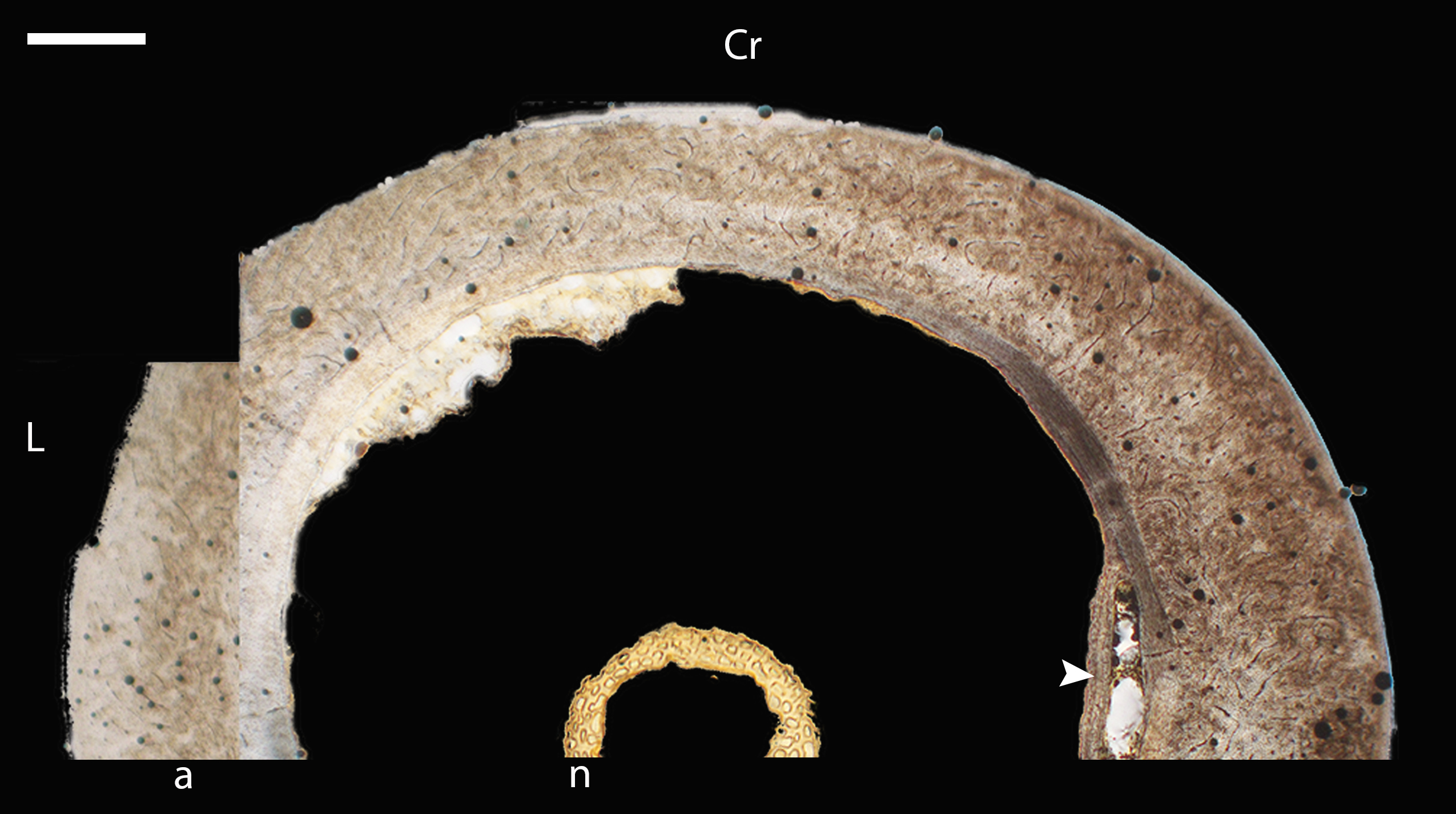

Supplement: Supplemental Information 25 — n = neonate, MVZ190822; a = adult, MVZ190831. Arrow indicates trabecular loop, which likely enclosed structures from a nutrient foramen. Cr, cranial; L, lateral. Scale bar equals 500 µm. [file peerj-09-12160-s025.jpg]

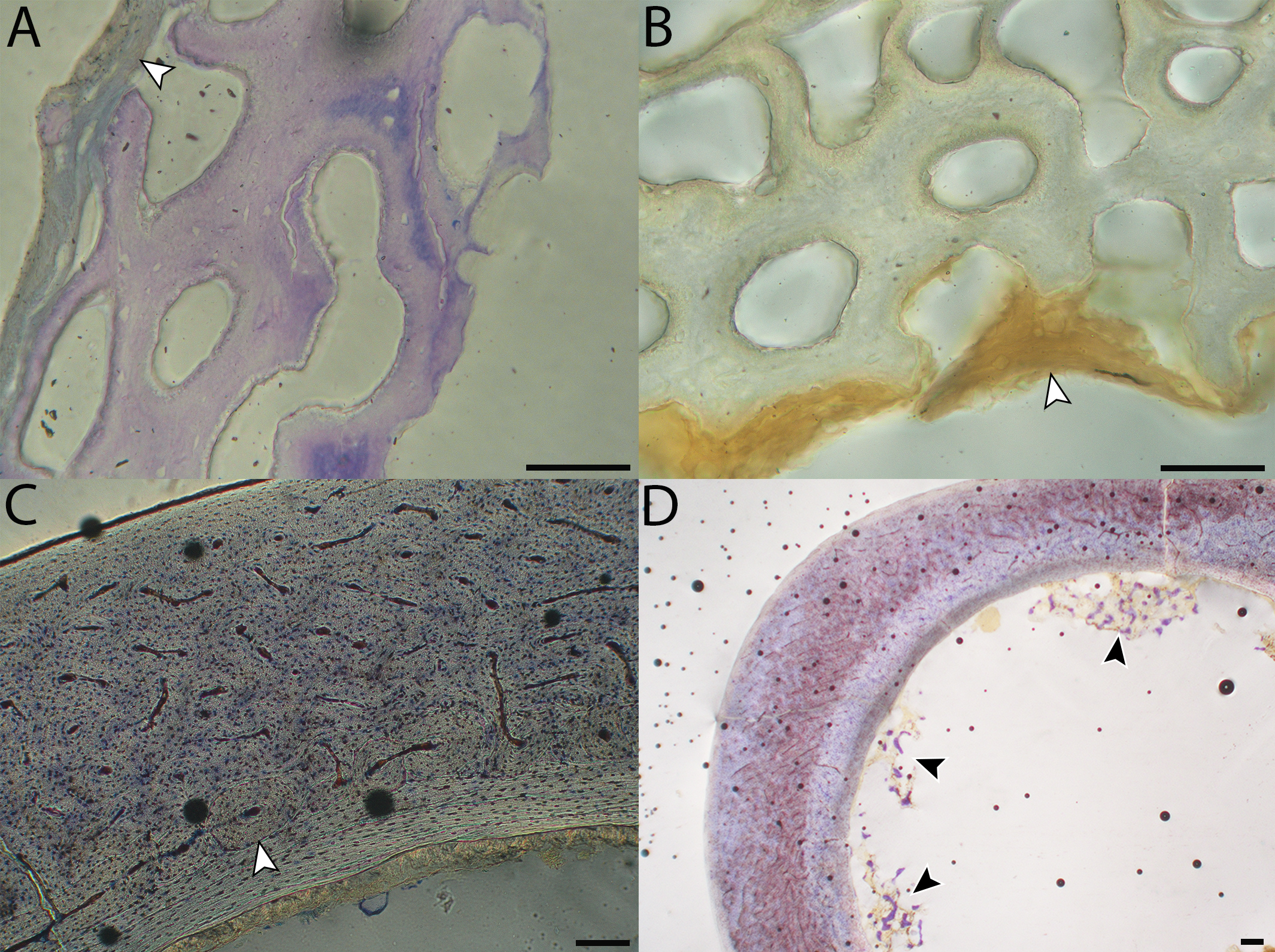

Supplement: Supplemental Information 26 — (A) Humerus of a neonate (MVZ190824), composed of woven bone with large, irregular vascular canals. Arrow indicates periosteum. Plane light, toluidine blue stain, 400×. (B) Humerus of a neonate (MVZ190822), also composed of woven bone with large, irregular vascular openings. Arrow indicates endosteum. Plane light, 400×. (C) Humerus of an adult (MVZ190829) showing a cortex of fibrolamellar bone between an OCL and ICL of avascular, parallel-fibered bone. Several secondary osteons are present near the ICL (one is indicated by an arrow). Plane light, toluidine blue stain, 100×. (D) Humerus of an adult (MVZ190831) with remnants of medullary bone (arrows). Plane light, toluidine blue stain, 40×. In all images the periosteal surface is oriented to top/left and the endosteal to the bottom/right. Scale bare equals 50 µm. [file peerj-09-12160-s026.jpg]

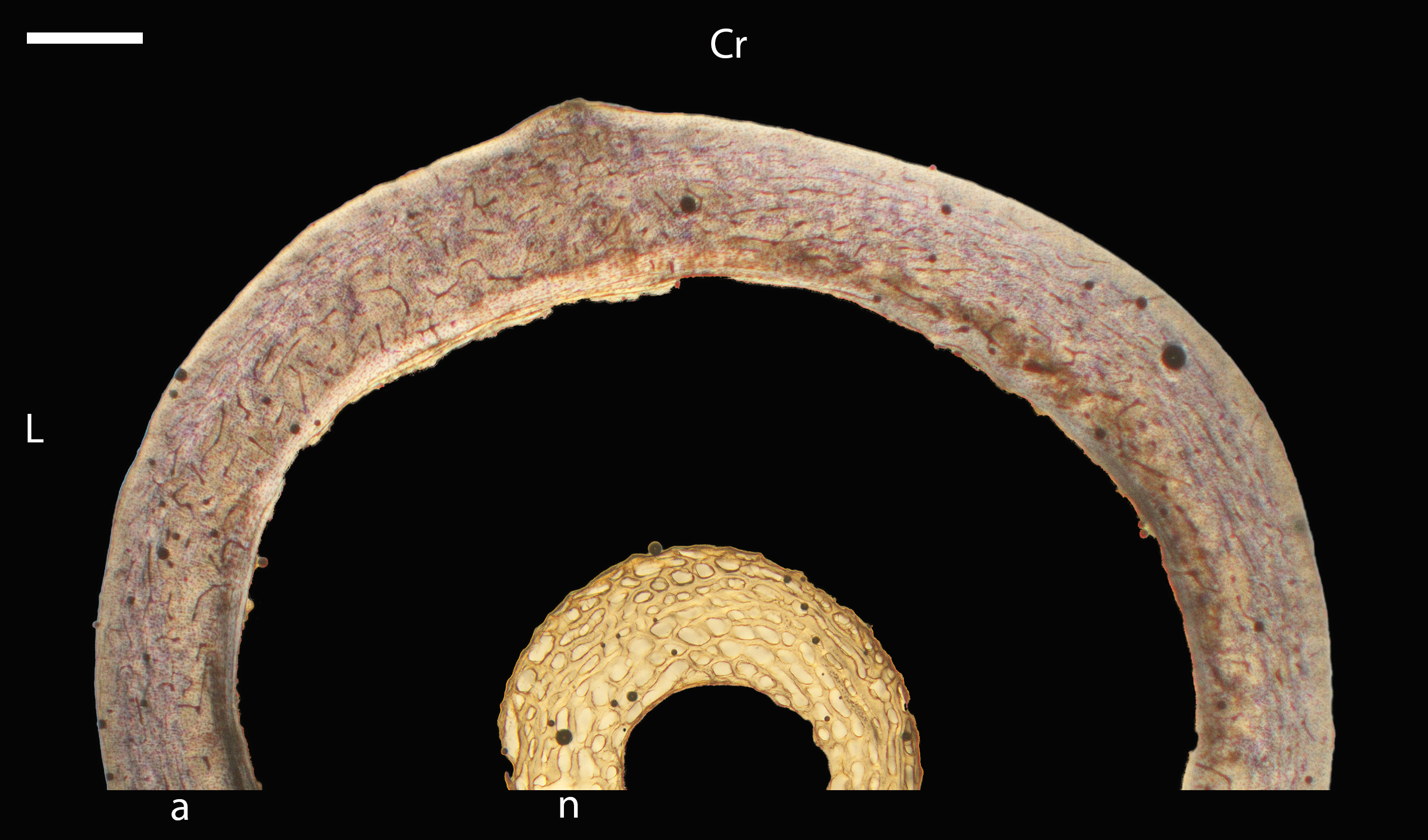

Supplement: Supplemental Information 27 — n = neonate, MVZ190824; a = adult, MVZ190829. Cr, cranial; L, lateral. Scale bar equals 500 µm. [file peerj-09-12160-s027.jpg]

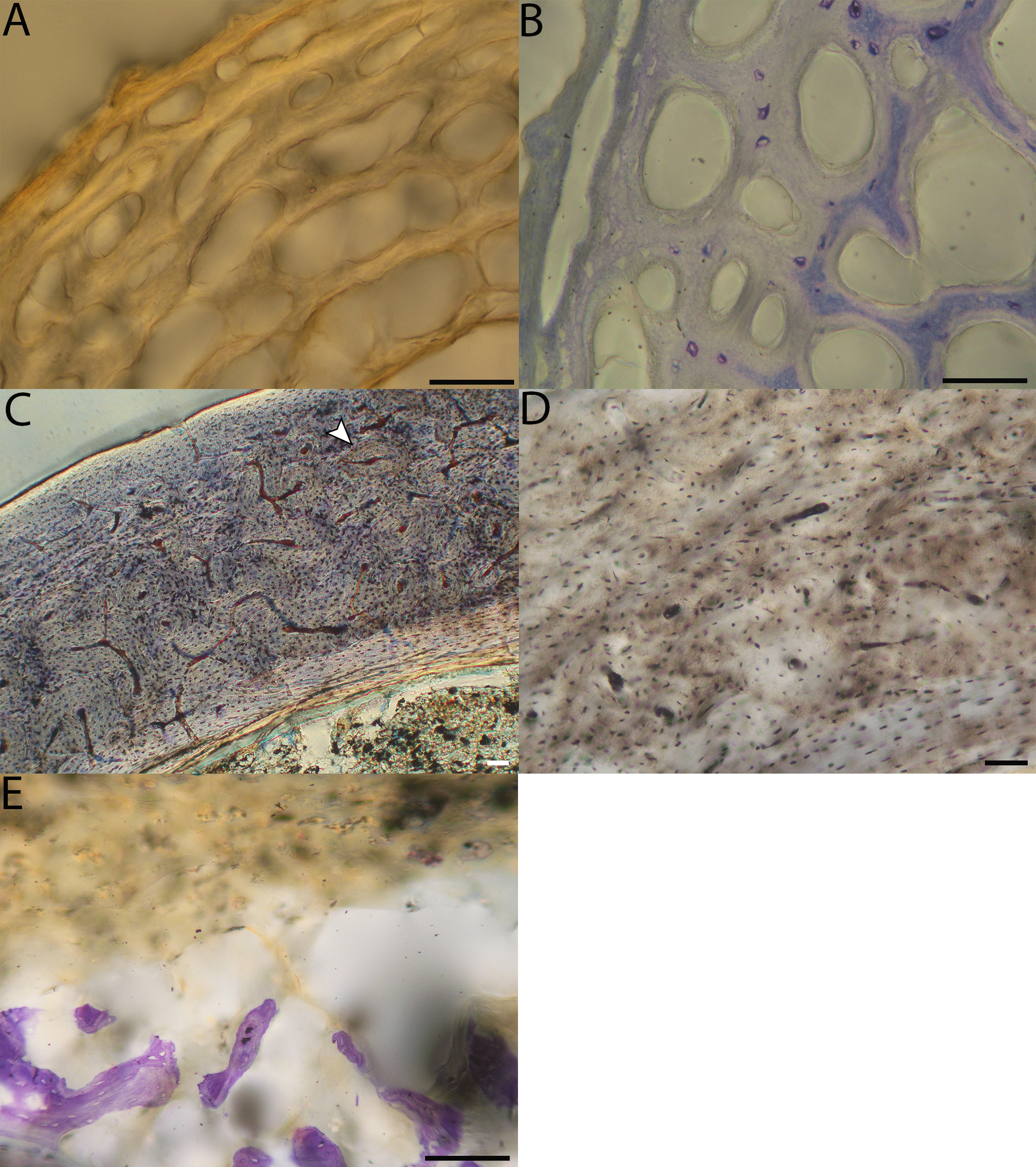

Supplement: Supplemental Information 28 — (A) Femur of a neonate (MVZ190824), composed of woven bone with very large vascular canals of variable shape. Plane light, 400×. (B) Femur of a neonate (MVZ190822) showing a relatively low number of osteocyte lacunae, which are rounded and lack cannaliculi. Plane light, toluidine blue stain, 400×. (C) Femur of an adult (MVZ190829) composed of fibrolamellar bone with numerous primary and secondary osteons (one example of the latter is indicated by an arrow). Plane light, toluidine blue stain, 100×. (D) Femur of an adult (MVZ190831). Plane light, toluidine blue stain, 200×. (E) Adult femur (MVZ190831), close-up of bone marrow (yellow tissue above) and delicate medullary bone (purple tissue below). Plane light, toluidine blue stain, 400×. In all images the periosteal surface is oriented to top/left and the endosteal to the bottom/right. Scale bare equals 50 µm. [file peerj-09-12160-s028.jpg]

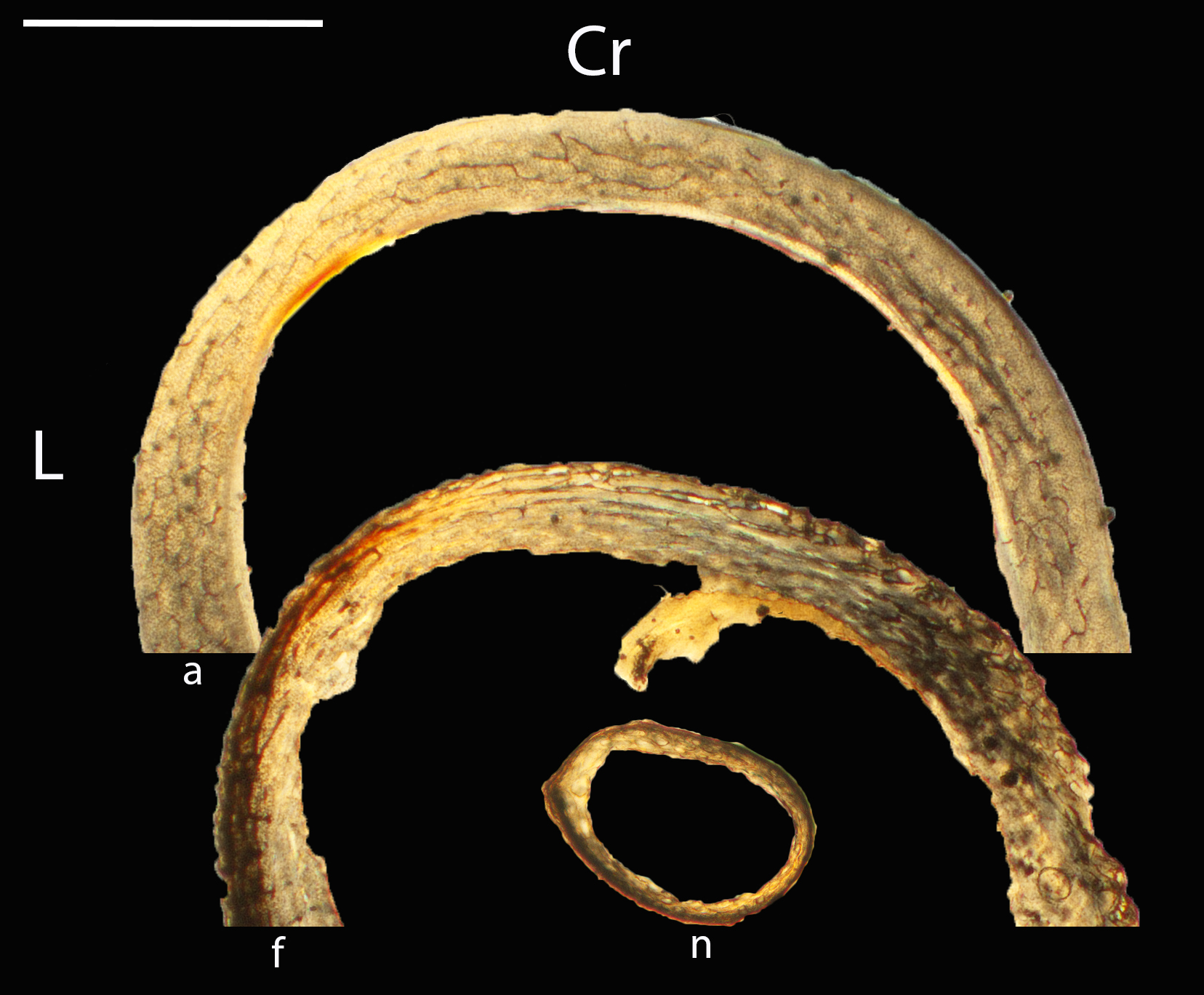

Supplement: Supplemental Information 29 — n = neonate, MVZ190890; f = fledgling, MVZ190888; a = adult, MVZ190892. Cr = cranial; L = lateral. Scale bar equals 1,000 µm. [file peerj-09-12160-s029.jpg]

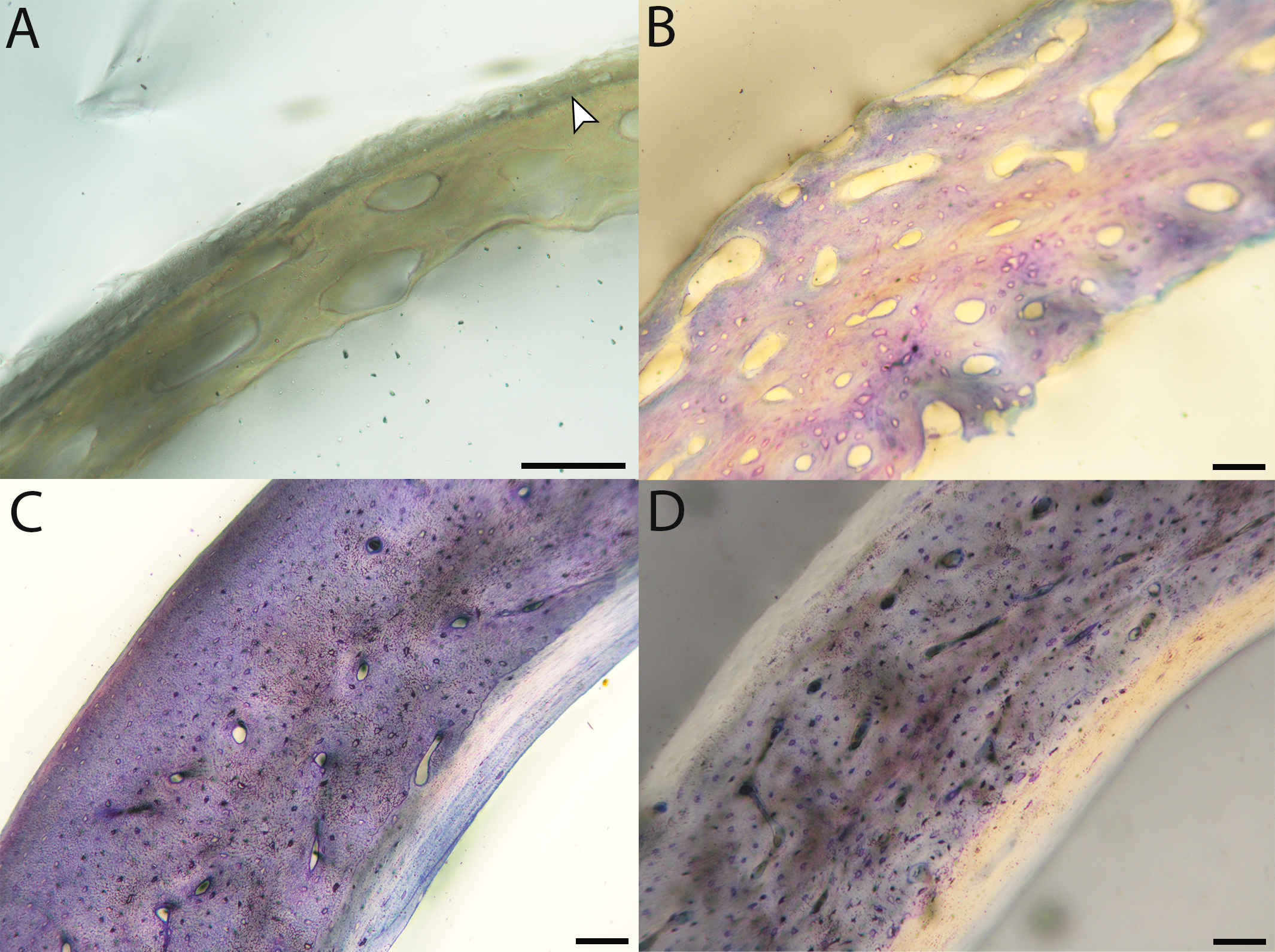

Supplement: Supplemental Information 30 — (A) Neonate humerus (MVZ190890) of woven bone with large vascular spaces and a thin layer of dense tissue surrounding it (arrow). Plane light, 400×. (B) Fledgling humerus (MVZ190888) showing a cortex composed of fibrolamellar bone endosteally (with longitdinal primary osteons) and incipient fibrolamellar bone periosteally (with incipient primary osteons in a variety of orientations). Plane light, toluidine blue stain, 200×. (C) Adult humerus (MVZ190892) of the more mature individual, showing an ICL and an OCL bordering a region of parallel-fibered bone with low levels of vascularity. Plane light, toluidine blue stain, 200×. (D) Adult humerus (MVZ190885) of the less mature individual, showing one of the larger regions of fibrolamellar bone; also notable is the incipient OCL, thinner than that seen in C. Plane light, 200×. In all images the periosteal surface is oriented to top/left and the endosteal to the bottom/right. Scale bare equals 50 µm. [file peerj-09-12160-s030.jpg]

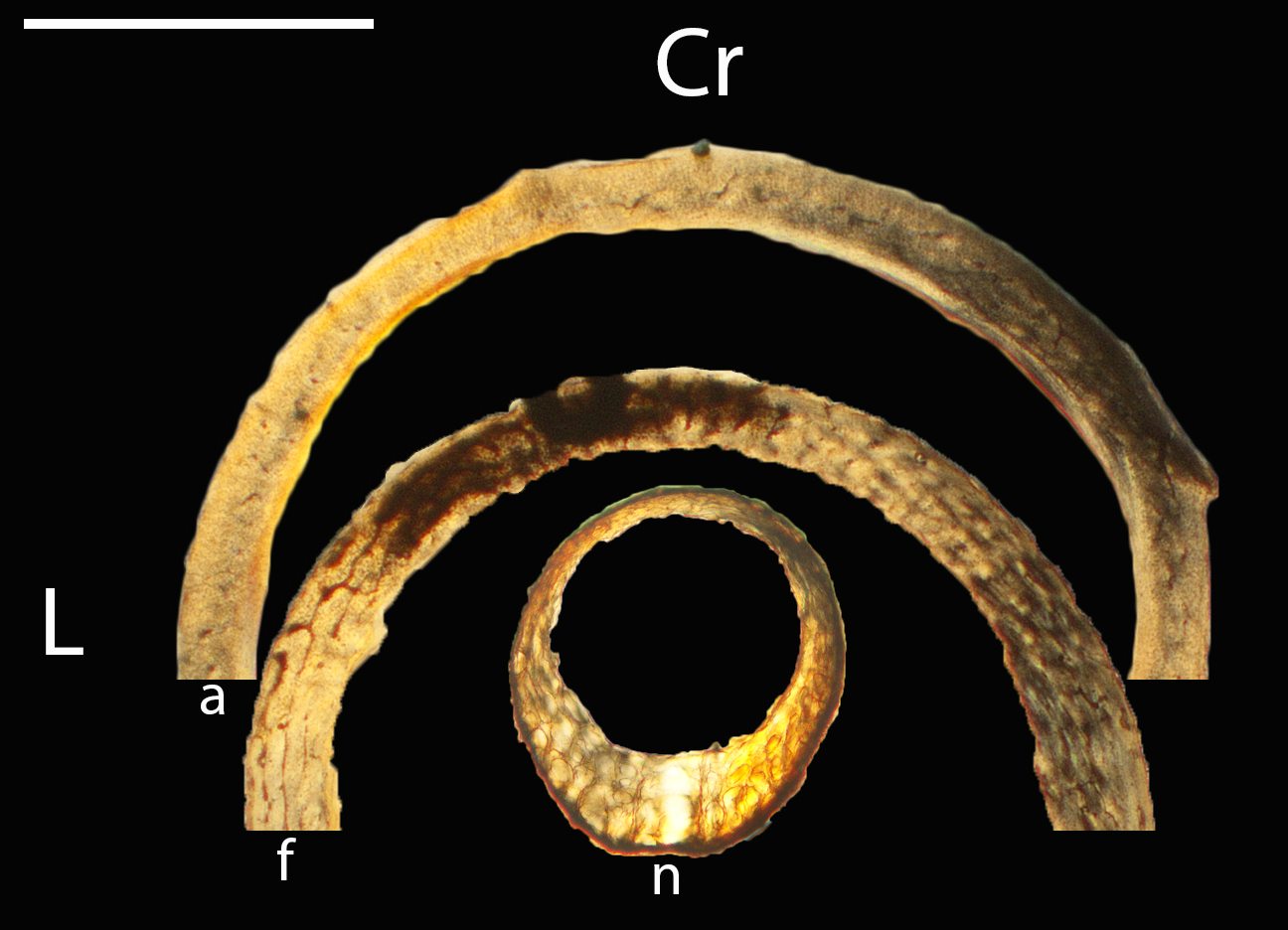

Supplement: Supplemental Information 31 — n = neonate, MVZ190890; f = fledgling, MVZ190888; a = adult, MVZ190892. Cr, cranial; L, lateral. Scale bar equals 1,000 µm. [file peerj-09-12160-s031.jpg]

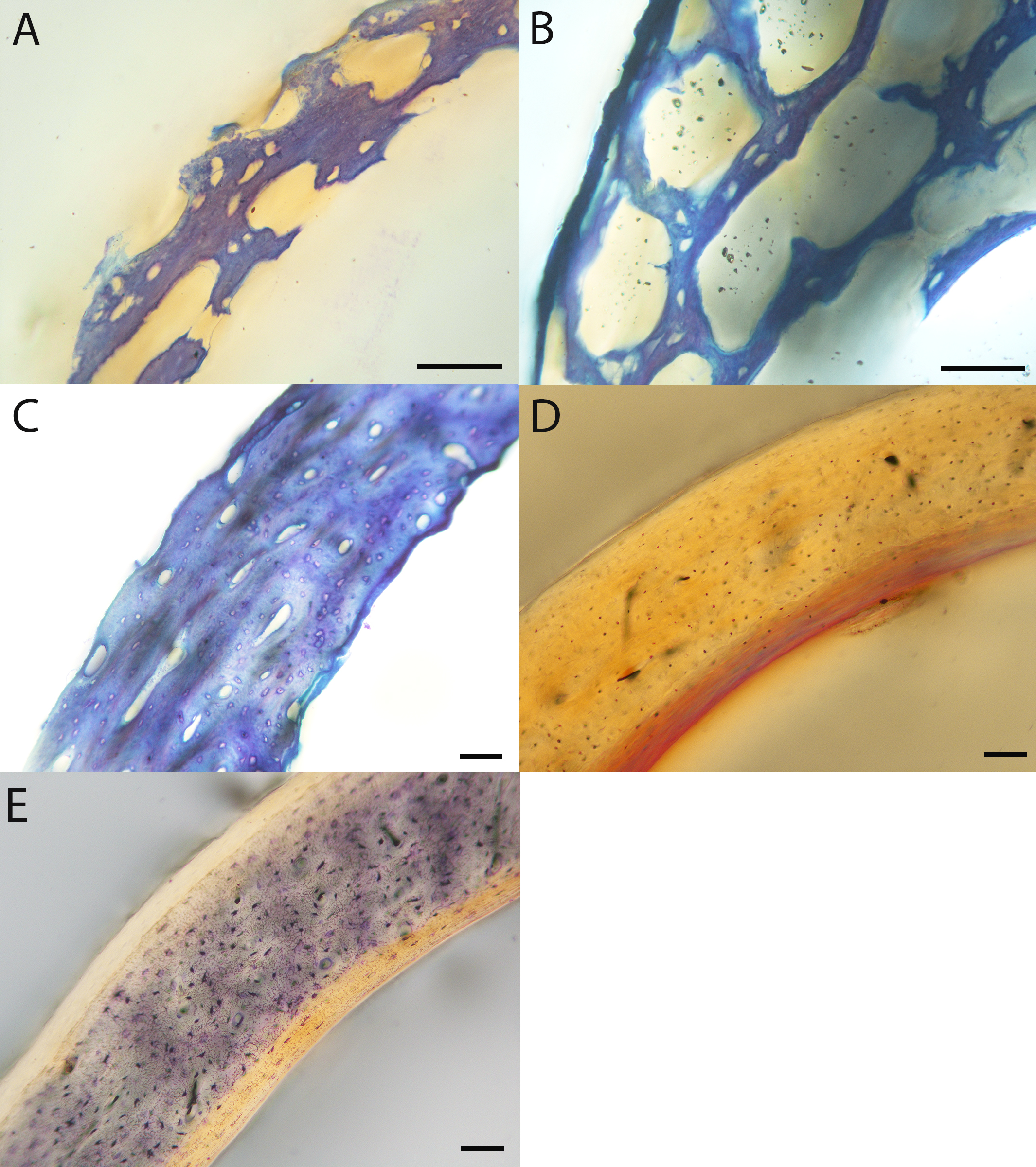

Supplement: Supplemental Information 32 — (A) Neonate femur (MVZ190887) showing the thinner region of the cortex, with relatively small, elongate vascular canals. Plane light, toluidine blue stain, 400×. (B) Neonate femur (MVZ190890) showing the thicker cortical bone with larger, more rounded vascular spaces and thinner struts of bone between them. Plane light, toluidine blue stain, 400×. (C) Fledgling femur (MVZ190888) showing an incipient fibrolamellar complex, with developing primary osteons and fairly large vascular spaces. Plane light, toluidine blue, 200×. D) Adult femur (MVZ190892) with few, small vascular canals; this part of the femur has fibrolamellar bone interspersed with parallel-fibered bone in the middle layer of the cortex. This individual appears to be a less-mature “adult,” with a more weakly-formed OCL. Plane light, 200×. (E) Adult femur (MVZ190885) mostly composed of parallel-fibered bone in this region of the cortex. This more mature adult has less fibrolamellar bone and a thicker OCL than the other “adult”. Plane light, toluidine blue stain, 200×. In all images the periosteal surface is oriented to top/left and the endosteal to the bottom/right. Scale bare equals 50 µm. [file peerj-09-12160-s032.jpg]

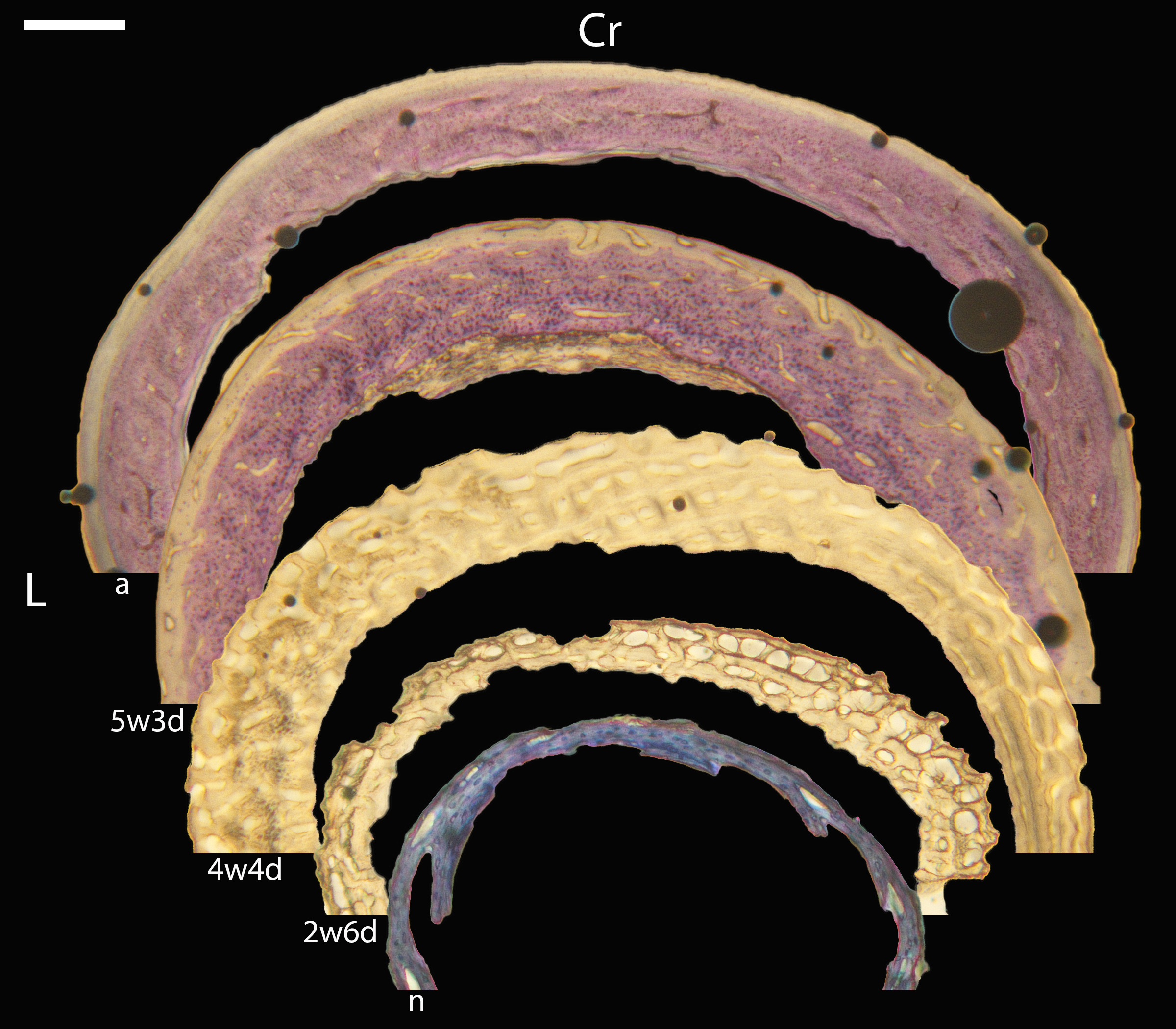

Supplement: Supplemental Information 33 — n = neonate, MVZ190895; 2w6d = 2 weeks and 6 days old, MVA190898; 4w4d = 4 weeks and 4 days old, MVZ190903; 5w3d = 5 weeks and 3 days old, MVZ190904; a = adult, MVZ190917. Cr, cranial; L, lateral. Scale bar equals 250 µm. [file peerj-09-12160-s033.jpg]

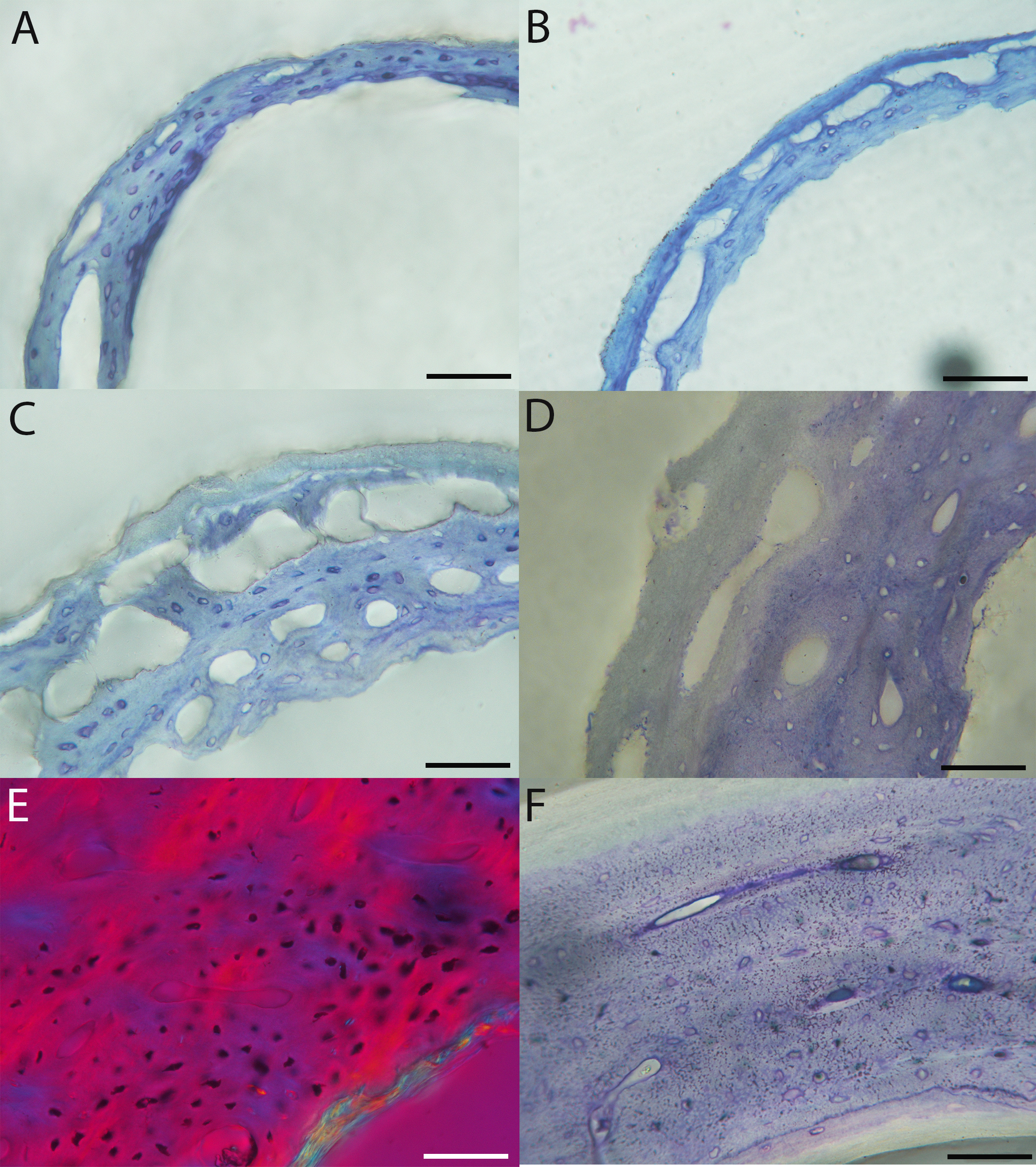

Supplement: Supplemental Information 34 — (A) Neonate humerus (MVZ190895) composed of woven bone with few large vascular chanells and moderate scalloping along the endosteal and periosteal margins. Plane light, toluidine blue stain, 400×. (B) Humerus of a 1-week-old chick (MVZ190896) showing characteristics similar to A but with increased vascular porosity. Plane light, toluidine blue stain, 200×. C) Humerus of a 2-week, 6-day-old chick (MVZ190898) with a thicker cortex still composed of woven bone; more endosteal canals are smaller and longitudinal, while channels in the periosteal region are larger and more immature. Plane light, toluidine blue stain, 400×. (D) Humerus of a 4-week, 4-day-old chick (MVZ190903) showing bone similar to C, but with smaller vascular channels and developing primary osteons. Plane light, toluidine blue stain, 400×. (E) Humerus of a 5-week, 3-day-old chick (MVZ190904) composed predominantly of fibrolamellar bone, but with several simple vascular canals; the endosteum lining the medullary cavity is also shown. Polarized light, 400×. (F) Adult humerus (MVZ190917), showing a prominent OCL and thin ICL surrounding a cortex of woven bone with primary vascular canals. Plane light, toluidine blue, 400×. In all images the periosteal surface is oriented to top/left and the endosteal to the bottom/right. Scale bare equals 50 µm. [file peerj-09-12160-s034.jpg]

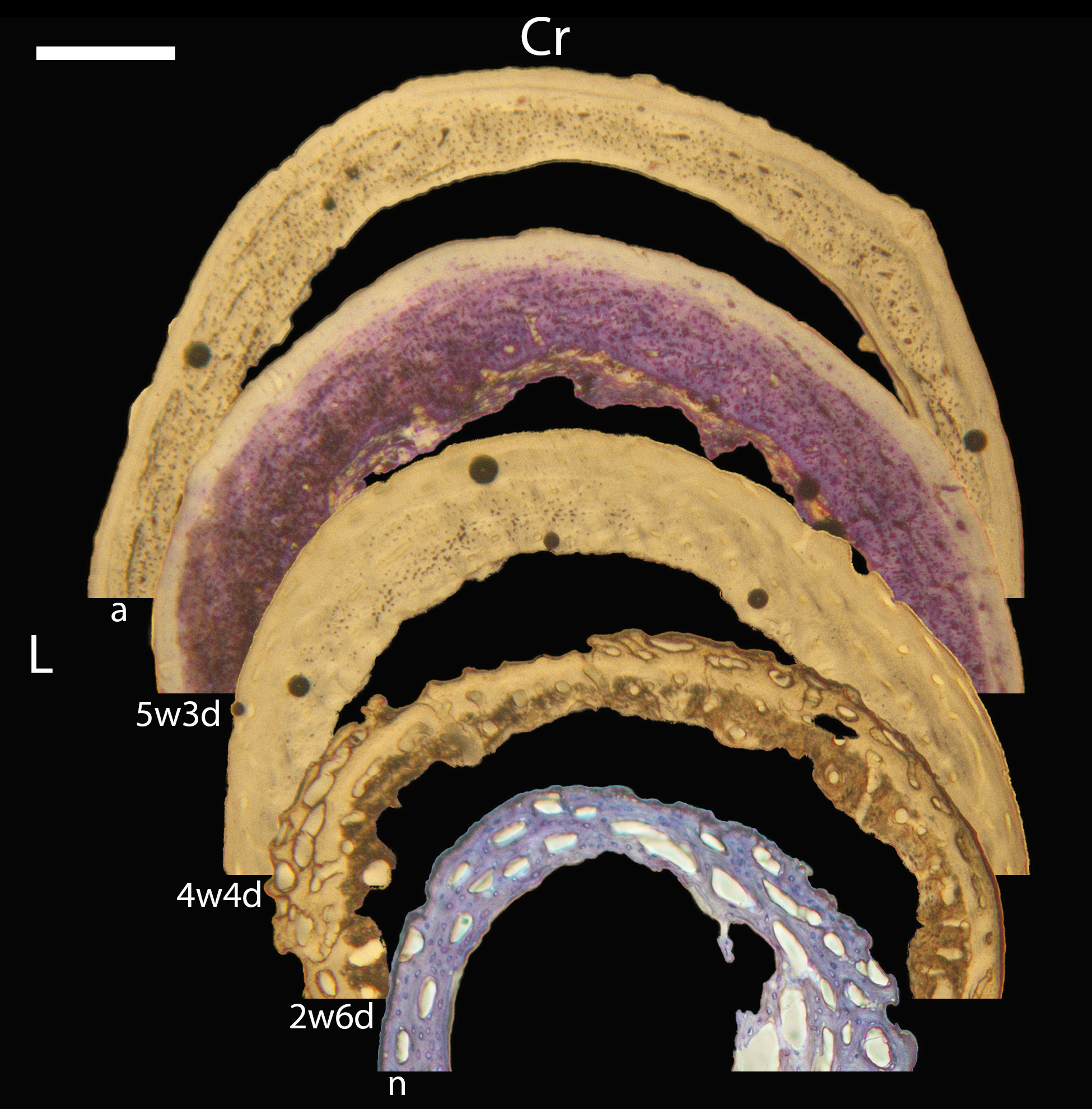

Supplement: Supplemental Information 35 — n = neonate, MVZ190895; 2w6d = 2 weeks and 6 days old, MVA190898; 4w4d = 4 weeks and 4 days old, MVZ190903; 5w3d = 5 weeks and 3 days old, MVZ190904; a = adult, MVZ190917. Cr, cranial; L, lateral. Scale bar equals 250 µm. [file peerj-09-12160-s035.jpg]

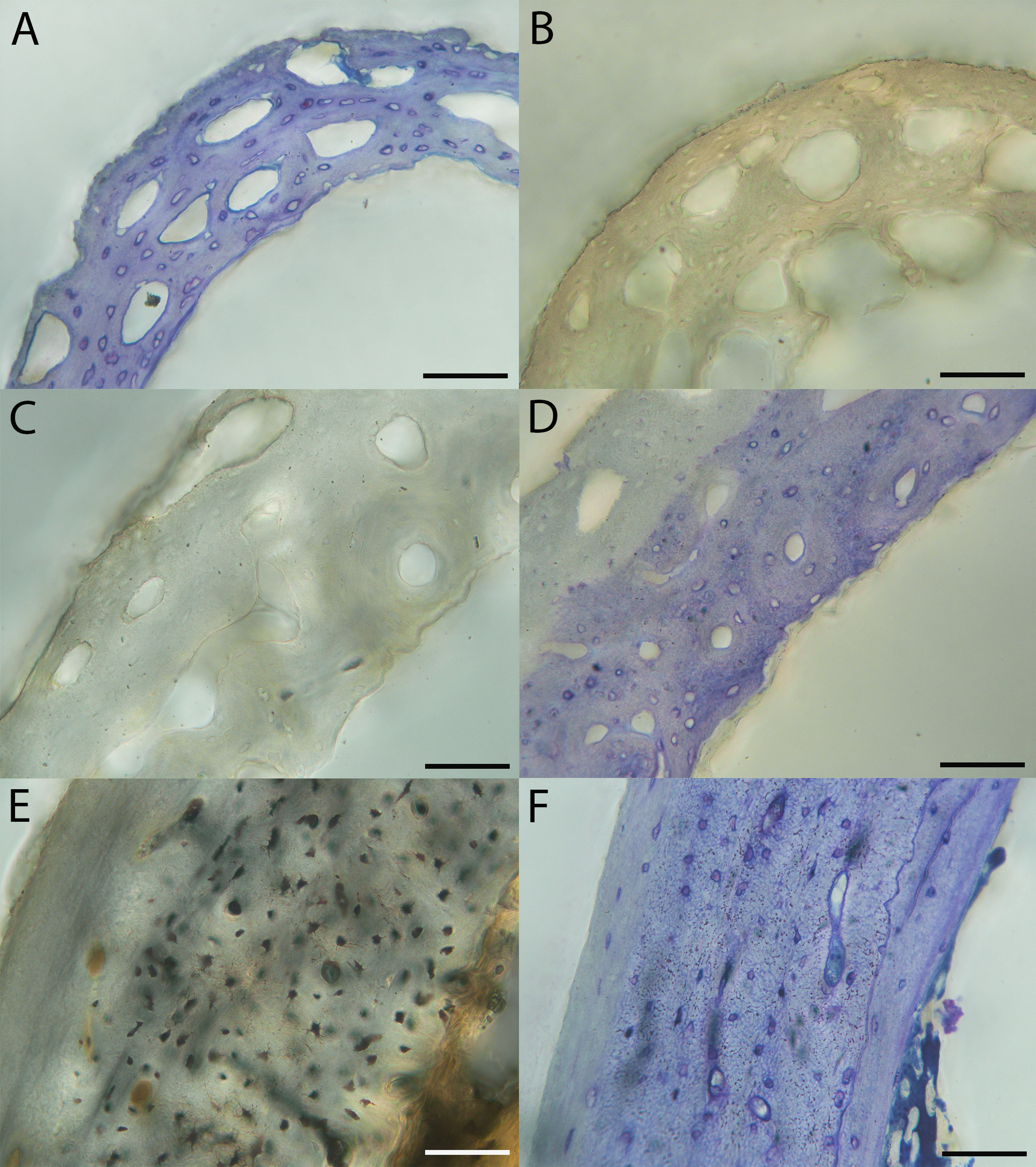

Supplement: Supplemental Information 36 — (A) Neonate femur (MVZ190895), showing a thick cortex of woven bone with large, irregular vascular channels with moderately-scalloped endosteal and periosteal margins. Plane light, toluidine blue stain, 400×. (B) Femur of a 1-week-old chick (MVZ190896), composed of woven bone with large vascular canals; this element shows a trend of larger, more immature vascular spaces in the endosteal half of the cortex. Plane light, 400×. (C) Femur of a 2-week, 6-day-old chick (MVZ190898), similar to B but with greater in-filling of vascular spaces. Plane light, 400×. (D) Femur of a 4-week, 4-day-old chick (MVZ190903) composed of fibrolamellar bone with mature and incipient primary osteons. Plane light, toluidine blue stain, 400×. (E) Femur of a 5-week, 3-day-old chick (MVZ190904) composed of fibrolamellar bone with an OCL and an actively-resorbing endosteal margin. Plane light, 400×. (F) Adult femur (MVZ190917) showing an OCL and ICL adjacent to a layer of weakly-woven bone with primary osteons and simple vascular canals. Plane light, toluidine blue stain, 400×. In all images the periosteal surface is oriented to top/left and the endosteal to the bottom/right. Scale bare equals 50 µm. [file peerj-09-12160-s036.jpg]

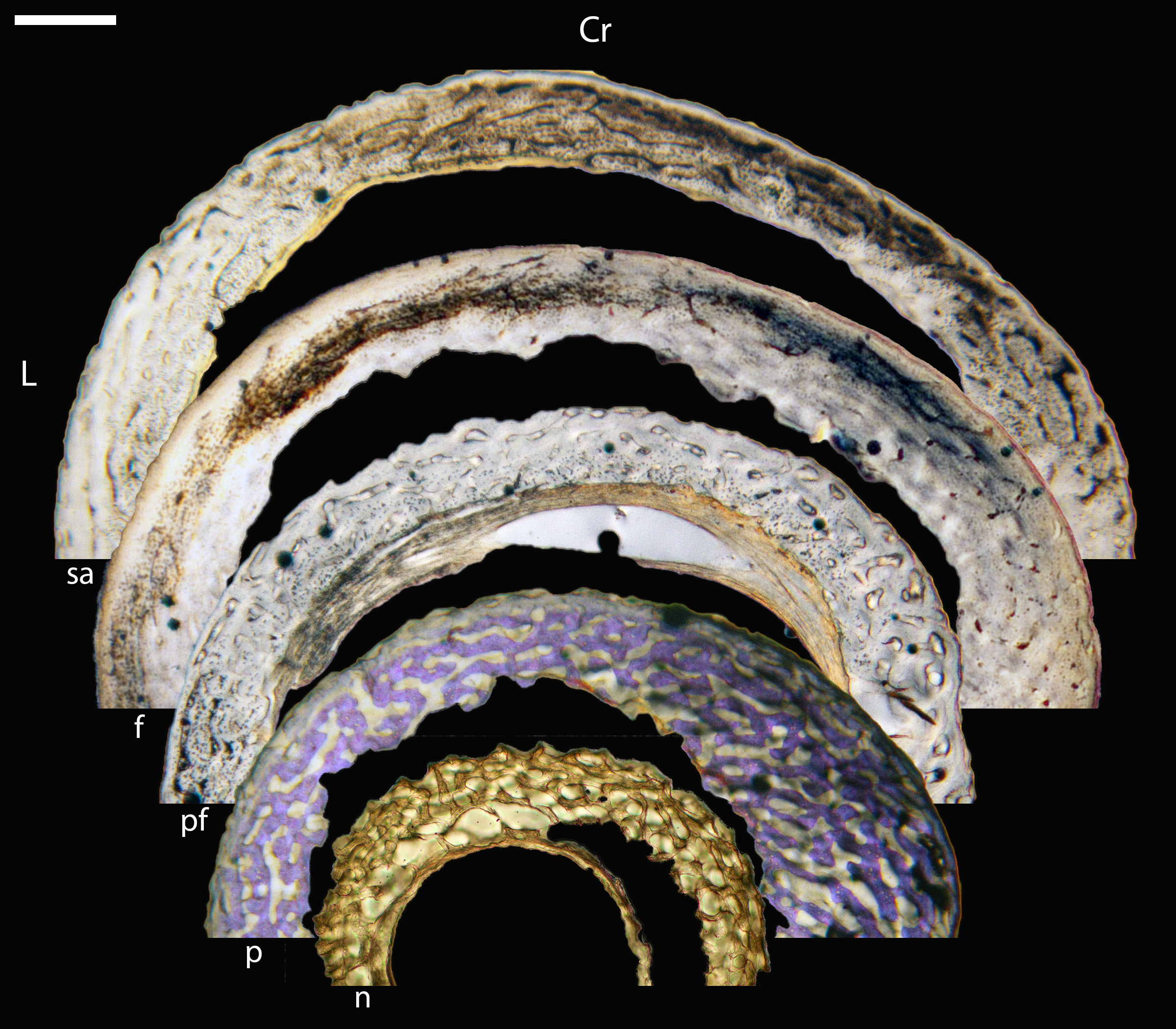

Supplement: Supplemental Information 37 — n = neonate, MVZ190927; p = pin-feathered chick, MVZ190929; pf = pre-fledgling, MVZ190921; f = fledgling, MVZ190931; sa = sub-adult, MVZ190923. Cr, cranial; L, lateral (anatomical directions cannot be applied to neonate). Scale bar equals 250 µm. [file peerj-09-12160-s037.jpg]

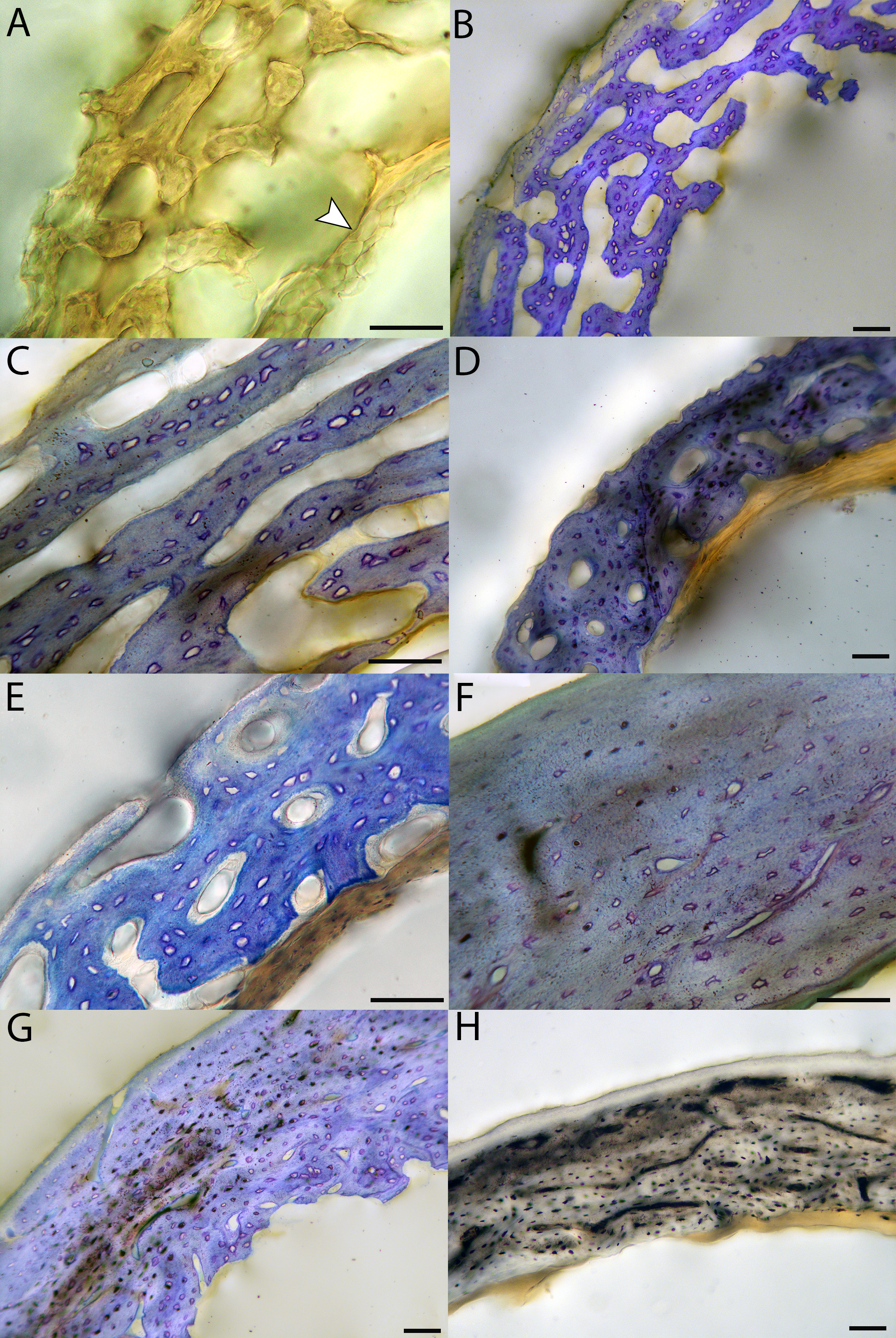

Supplement: Supplemental Information 38 — (A) Neonate humerus (MVZ190927) composed of woven bone with very large, irregular vascular spaces. Plane light, toluidine blue stain, 400×. (B) Humerus of a pin-feathered chick (MVZ190929) showing a cortex of woven bone largely similar to the neonate, but with smaller vascular canals and, as a consequence, thicker bone between vascular spaces. Plane light, toluidine blue stain, 200×. (C) Humerus of a pre-fledgling chick (MVZ190928), showing a region of circumferentially-oriented vascular channels, one example of the variation in vascular orientation observed at this growth stage. Plane light, toluidine blue stain, 400×. (D) Humerus of a pre-fledgling chick (MVZ190921) composed of woven bone, showing additional examples of variation in vascular channel orientation (here both longitudinal and reticular are seen). Plane light, toluidine blue stain, 200×. (E) Humerus of a pre-fledgling chick (MVZ190922) showing an incipient fibrolamellar complex, characteristic of this age group. Plane light, toluidine blue stain, 400×. (F) Humerus of a fledgling chick (MVZ190930) showing a region of parallel-fibered bone with low vascularity in the fibrolamellar cortex. Plane light, toluidine blue stain, 400×. (G) Humerus of a fledgling chick (MVZ190931) showing a cortex composed of fibrolamellar bone with small, longitudinal canals and an incipient OCL along the periosteal margin. Plane light, toluidine blue stain, 200×. (H) Humerus of a sub-adult (MVZ190923) showing microanatomical characteristics very similar to F and G. Plane light, 200×. In all images the periosteal surface is oriented to top/left and the endosteal to the bottom/right. Scale bare equals 50 µm. [file peerj-09-12160-s038.jpg]

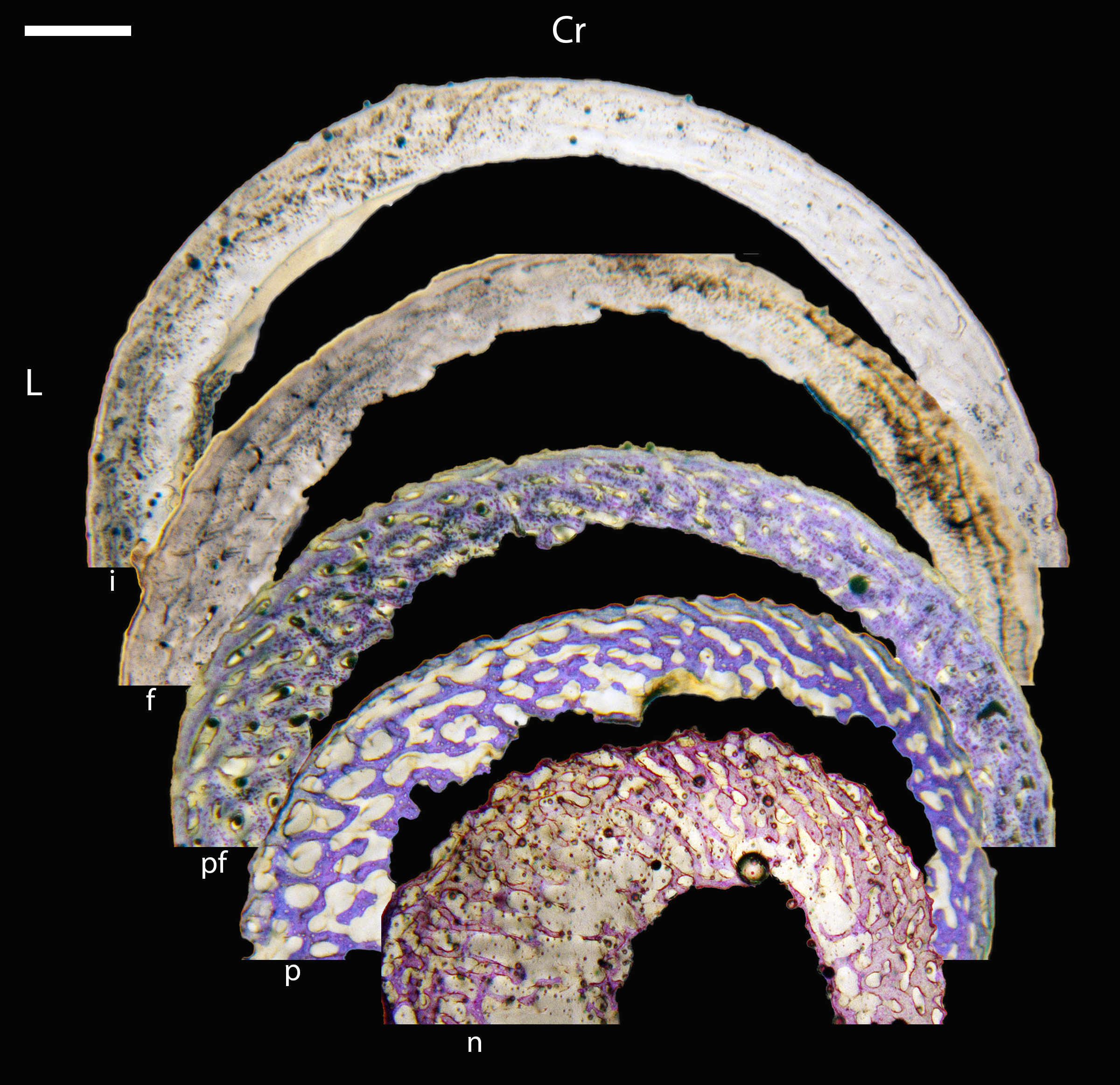

Supplement: Supplemental Information 39 — n = neonate, MVZ190927; p = pin-feathered chick, MVZ190929; pf = pre-fledgling, MVZ190921; f = fledgling, MVZ190931; sa = sub-adult, MVZ190923. Cr, cranial; L, lateral (anatomical directions cannot be applied to neonate). Scale bar equals 250 µm. [file peerj-09-12160-s039.jpg]

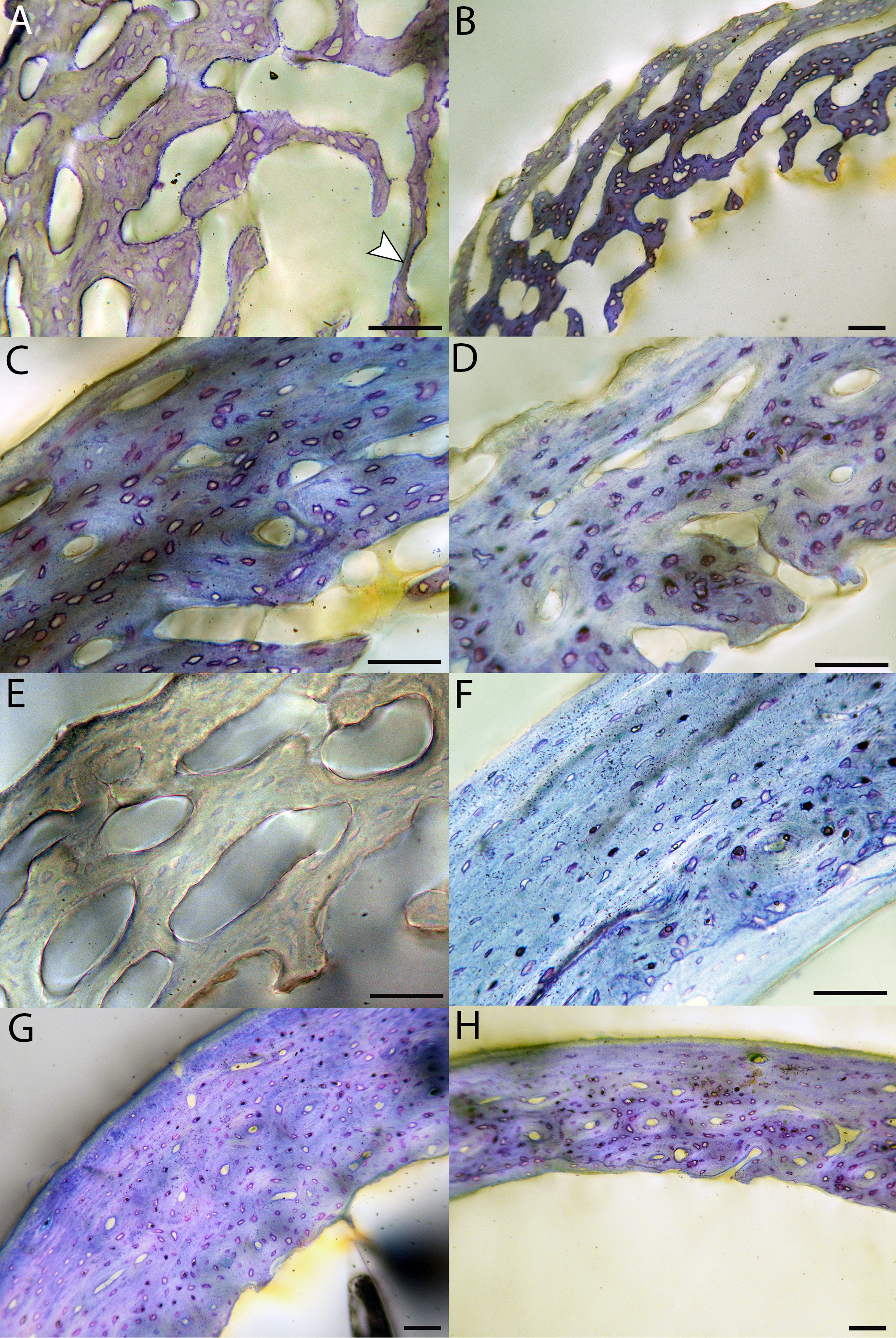

Supplement: Supplemental Information 40 — (A) Neonate femur (MVZ190927) showing a thick cortex of woven bone with high vascular porosity. Plane light, toluidine blue stain, 400×. (B) Femur of a pin-feathered chick (MVZ190929) showing immature bone generally similar to A but with a thinner cortical wall. Plane light, toluidine blue stain, 200×. (C) Femur of a pre-fledgling chick (MVZ190928) composed of woven bone with numerous incipient primary osteons, predominantly longitudinal in orientation in this cortical region of this individual. Plane light, toluidine blue stain, 400×. (D) Femur of a pre-fledgling chick (MVZ190921), showing diversity of orientation of vascular canals at this growth phase; here, there are anastomosing and circumferential channels. Plane light, toluidine blue stain, 400×. (E) Femur of a pre-fledgling chick (MVZ190922) showing a region of more immature bone (woven but without incipient primary osteons), highlighting osteohistological variation in and among individuals at this growth stage. Plane light, 400×. (F) Femur of a fledgling chick (MVZ190930) showing a patch of parallel-fibered bone, and primary osteons clustered near the endosteal margin. Plane light, toluidine blue stain, 400×. (G) Femur of a fledgling chick (MVZ190931) showing fibrolamellar bone . Plane light, toluidine blue stain, 200×. (H) Femur of a sub-adult (MVZ190923) showing characteristics broadly similar to F and G, with a marginally thinner cortex. Plane light, toluidine blue stain, 200×. In all images the periosteal surface is oriented to top/left and the endosteal to the bottom/right. Scale bare equals 50 µm. [file peerj-09-12160-s040.jpg]

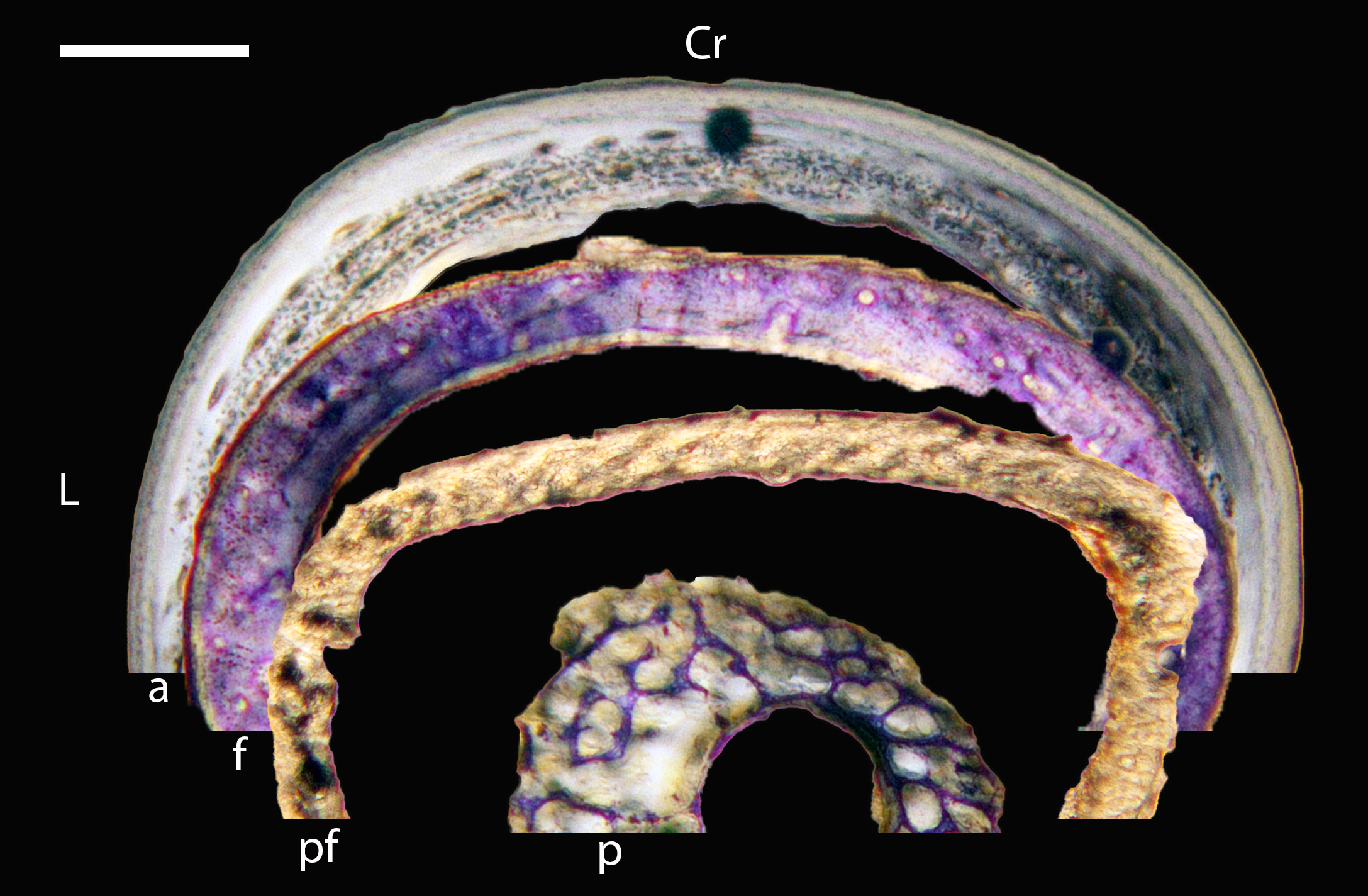

Supplement: Supplemental Information 41 — p = pin-feathered chick, MVZ190984; pf = pre-fledgling, MVZ190994; f = fledgling, MVZ190991; a = adult, MVZ190993. Cr, cranial; L, Lateral. Scale bar equals 250 µm. [file peerj-09-12160-s041.jpg]

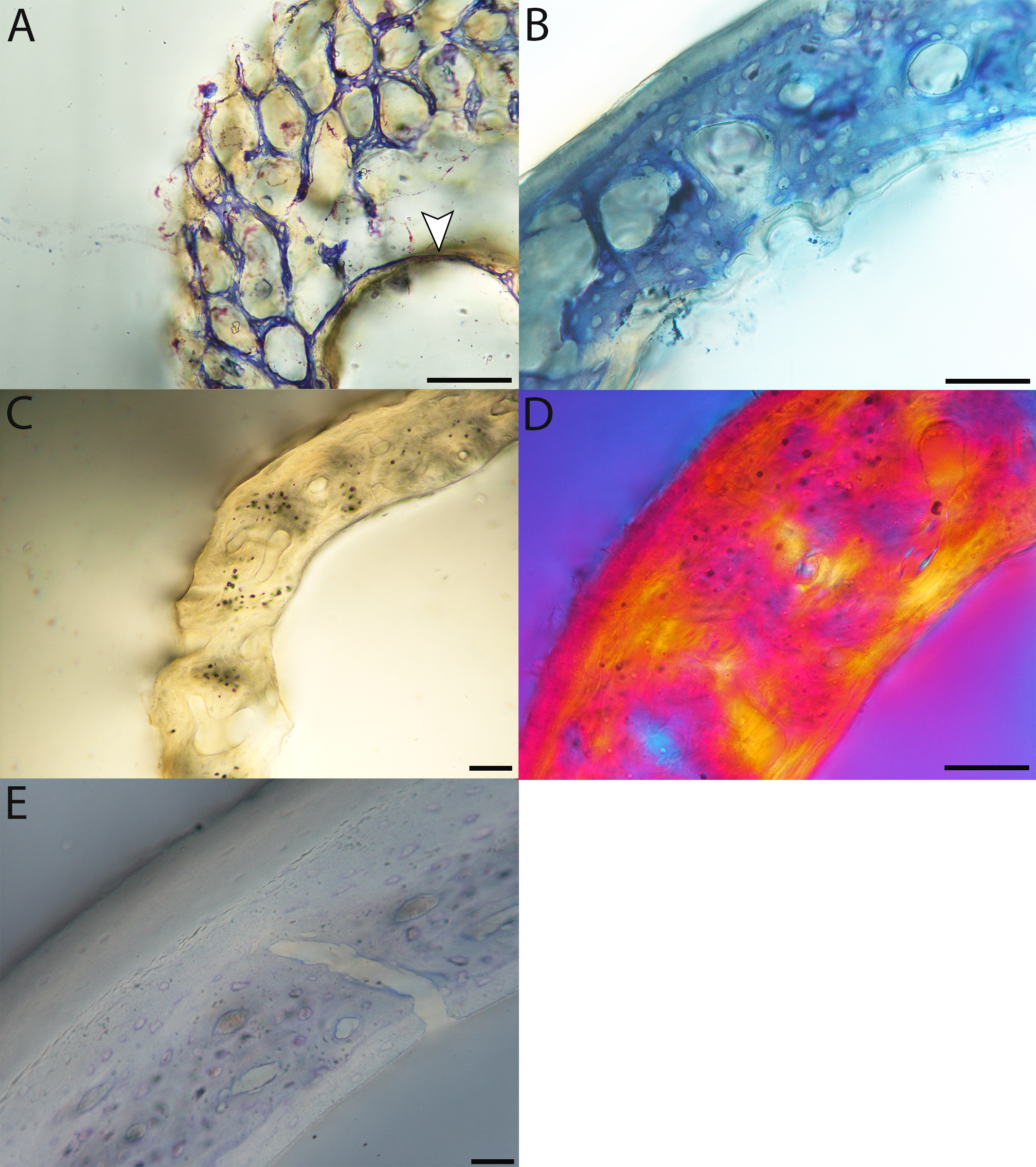

Supplement: Supplemental Information 42 — (A) Humerus of a pin-feathered chick (MVZ190984) composed of highly-porous woven bone, lined by a thin circlet of endosteal bone. Plane light, toluidine blue stain, 200×. (B) Humerus of a pre-fledgling chick (MVZ190967) showing a woven matrix with large vascular canals; these spaces are much smaller than in the pin-feathered stage. Plane light, toluidine blue stain, 400×. (C) Humerus of a pre-fledgling chick (MVZ190994) composed of woven bone with simple vascular canals and incipient primary osteons. Plane light, 200×. (D) Humerus of a fledgling chick (MVZ190991), composed of fibrolamellar bone and with a developing ICL. Polarized light, 400×. (E) Adult humerus (MVZ190993), showing a thick OCL, middle layer of fibrolamellar bone, and ICL. Plane light, toluidine blue, 400×. In all images the periosteal surface is oriented to top/left and the endosteal to the bottom/right. Scale bare equals 50 µm. [file peerj-09-12160-s042.jpg]

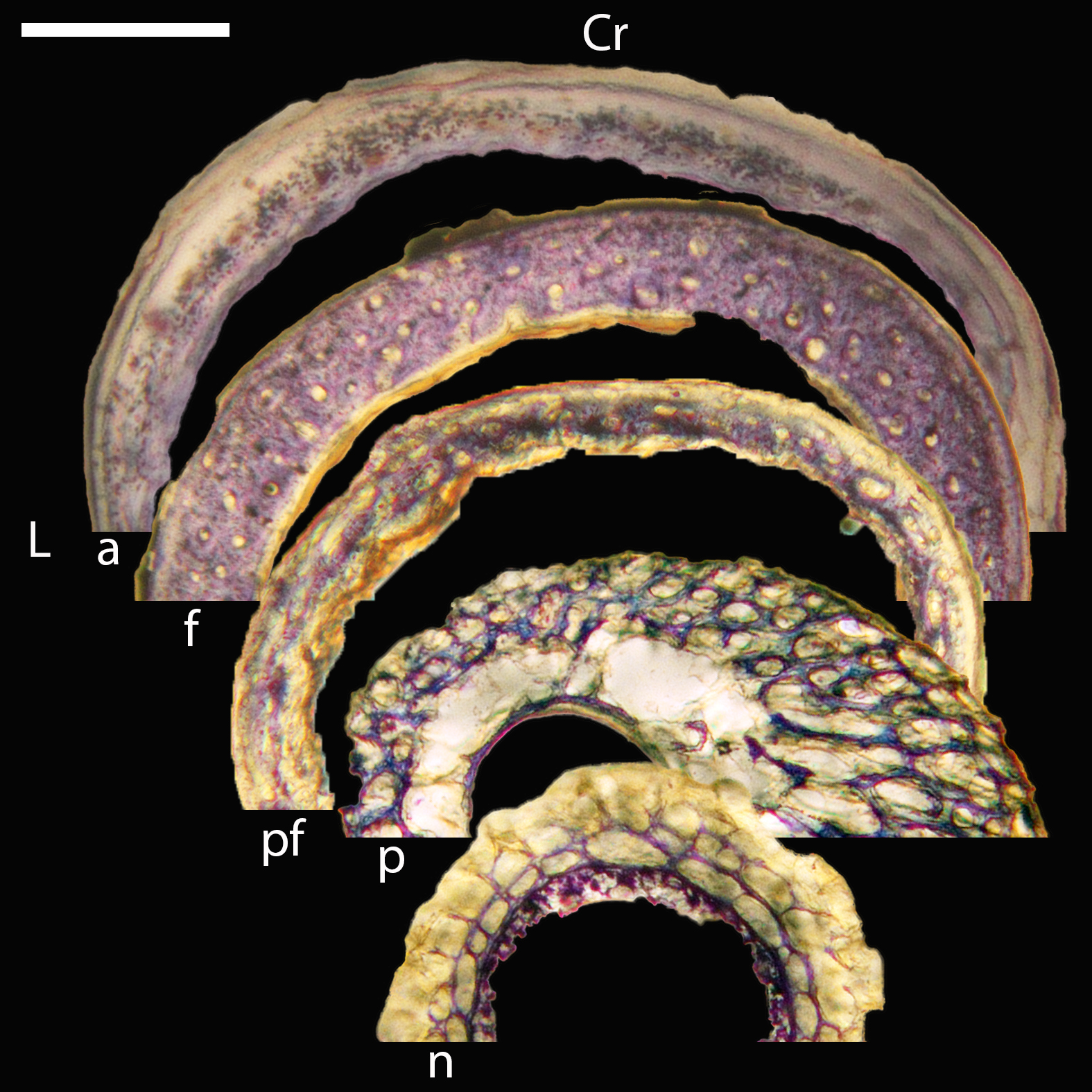

Supplement: Supplemental Information 43 — (n = neonate, MVZ190969; p = pin-feathered chick, MVZ190984; pf = pre-fledgling, MVZ190994; f = fledgling, MVZ190991; a = adult, MVZ190993. Cr, cranial; L, Lateral. Scale bar equals 250 µm. [file peerj-09-12160-s043.jpg]

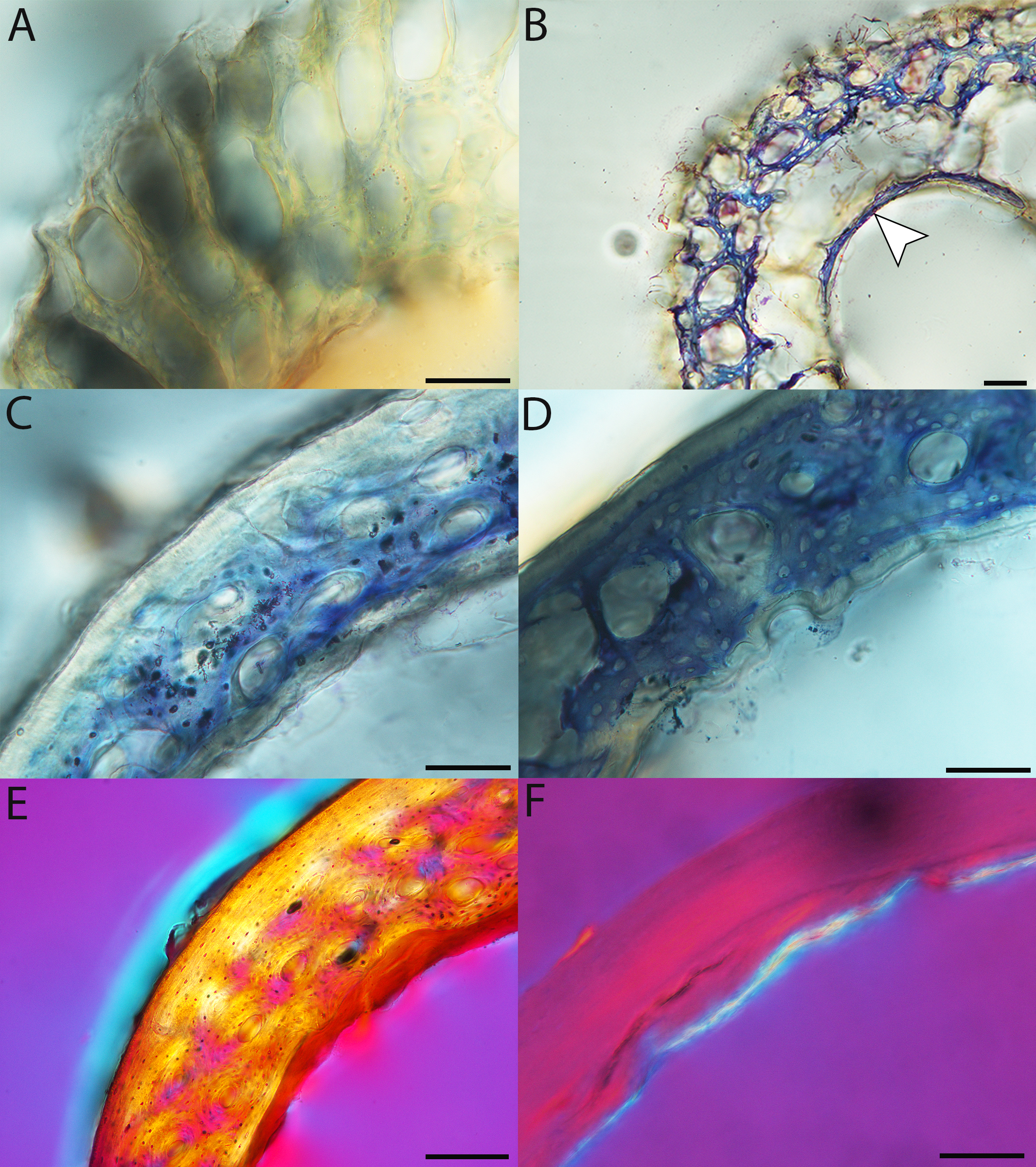

Supplement: Supplemental Information 44 — (A) Neonate femur (MVZ190969) showing a thick cortex of woven bone with large vascular canals and thin struts of bone separating them. Plane light, 400×. (B) Femur of a pin-feathered chick (MVZ190984) showing features similar to A, but with thicker bony struts separating smaller vascular spaces. Plane light, 200×. (C) Femur of a pre-fledgling chick (MVZ190994) composed of a developing fibrolamellar complex with an incipient OCL. Plane light, toluidine blue stain, 400×. (D) Femur of a pre-fledgling chick (MVZ190967) showing features similar to C; some incipient primary osteons are visible, though some simple vascular canals also remain. Plane light, toluidine blue stain, 400×. (E) Femur of a fledgling chick (MVZ190991) showing a fibrolamellar cortex that is more mature than in the pre-fledgling chicks, but still not finished developing. (F) Adult femur (MVZ190993) showing an avascular region of parallel-fibered bone; other regions of the cortex have woven bone and simple vascular canals. In all images the periosteal surface is oriented to top/left and the endosteal to the bottom/right. Scale bare equals 50 µm. [file peerj-09-12160-s044.jpg]

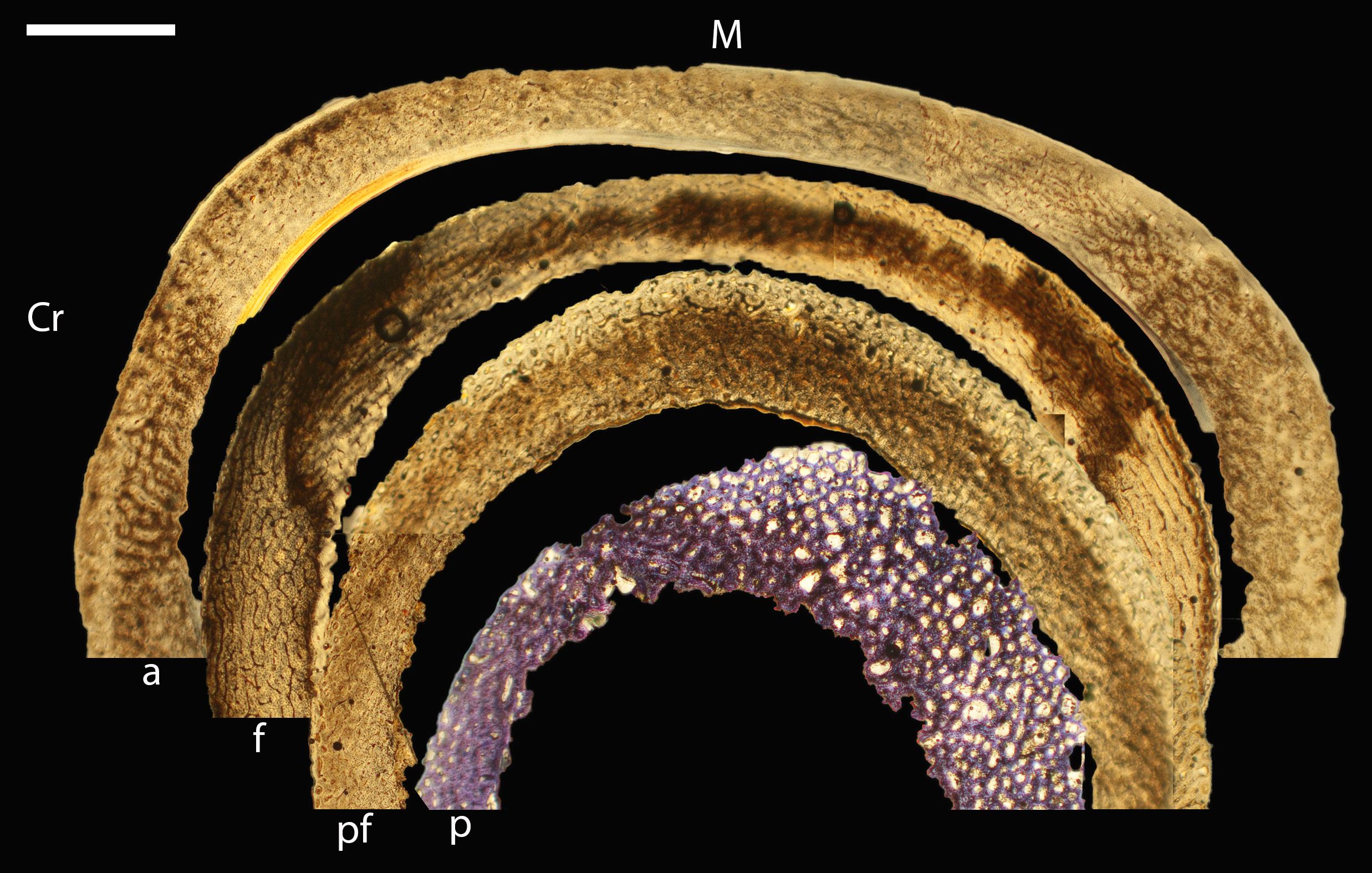

Supplement: Supplemental Information 45 — p = pin-feathered, MVZ190881; pf = pre-fledgling, MVZ190882; f = fledgling, MVZ190879; a = adult, MVZ190883. Cr, cranial; M, medial. Scale bar equals 1,000 µm. [file peerj-09-12160-s045.jpg]

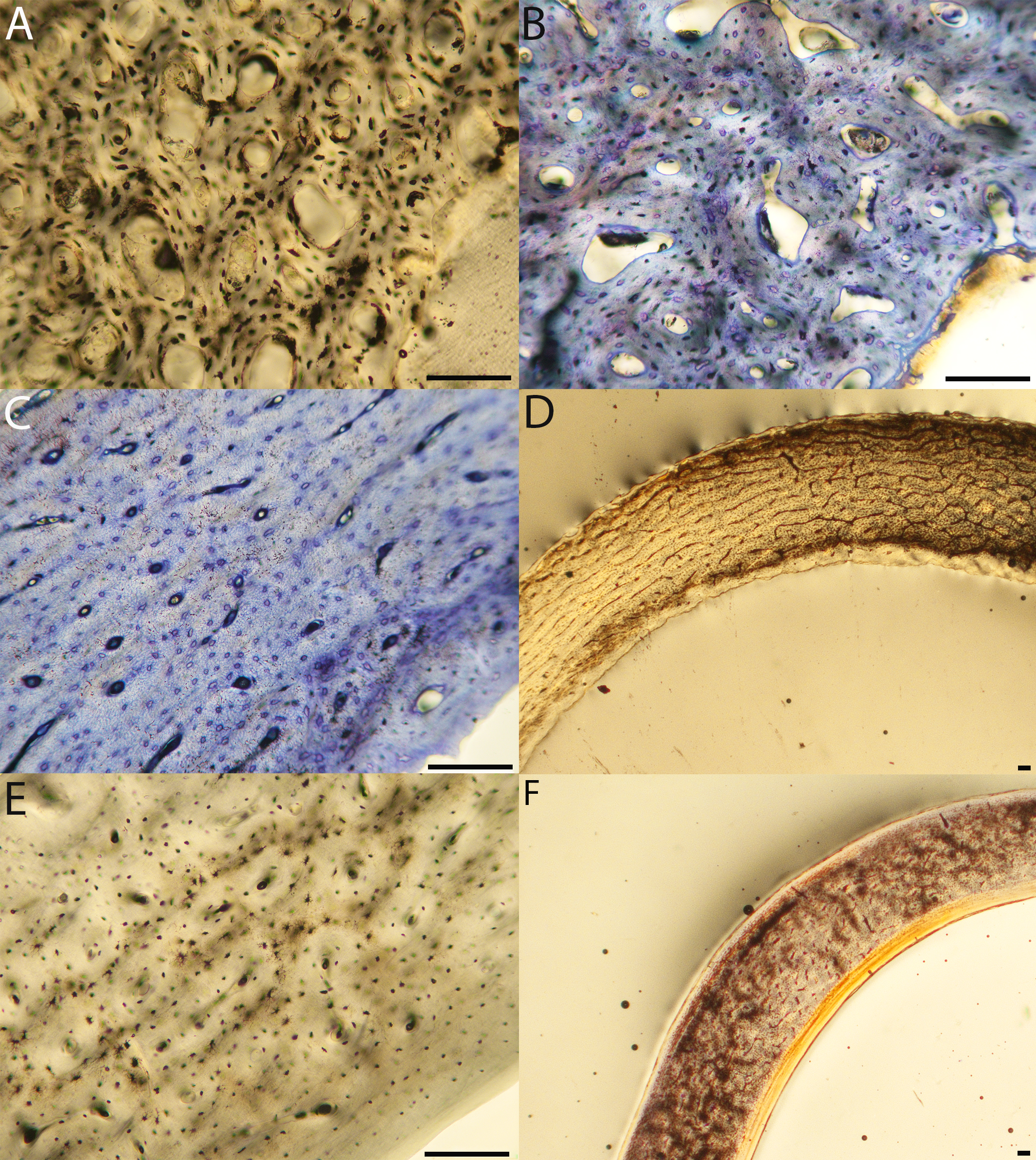

Supplement: Supplemental Information 46 — (A) Humerus of a pin-feathered chick (MVZ190881) showing a thick cortex of woven bone with high vascular porosity and some incipient primary osteons. Plane light, 400×. (B) Humerus of a pre-fledgling chick (MVZ190882). with charactiristics broadly similar to A, but with more matury primary osteons and smaller vascular canals. Plane light, toluidine blue, 400×. (C) Humerus of a fledgling chick (MVZ190879) showing mature fibrolamellar bone in the cortex. Plane light, toluidine blue stain, 400×. (D) Humerus of a fledgling chick (MVZ190879) showing variation in orientation of vascular canals, as well as an incipient OCL and ICL. Plane light, 40×. (E) Adult humerus (MVZ190883) of mature fibrolamellar bone. Plane light, 400×. (F) Adult humerus (MVZ190883) showing moderate level of porosity, strongly-developed ICL, and clear OCL. Plane light, toluidine blue stain, 40×. In all images the periosteal surface is oriented to top/left and the endosteal to the bottom/right. Scale bare equals 100 µm. [file peerj-09-12160-s046.jpg]

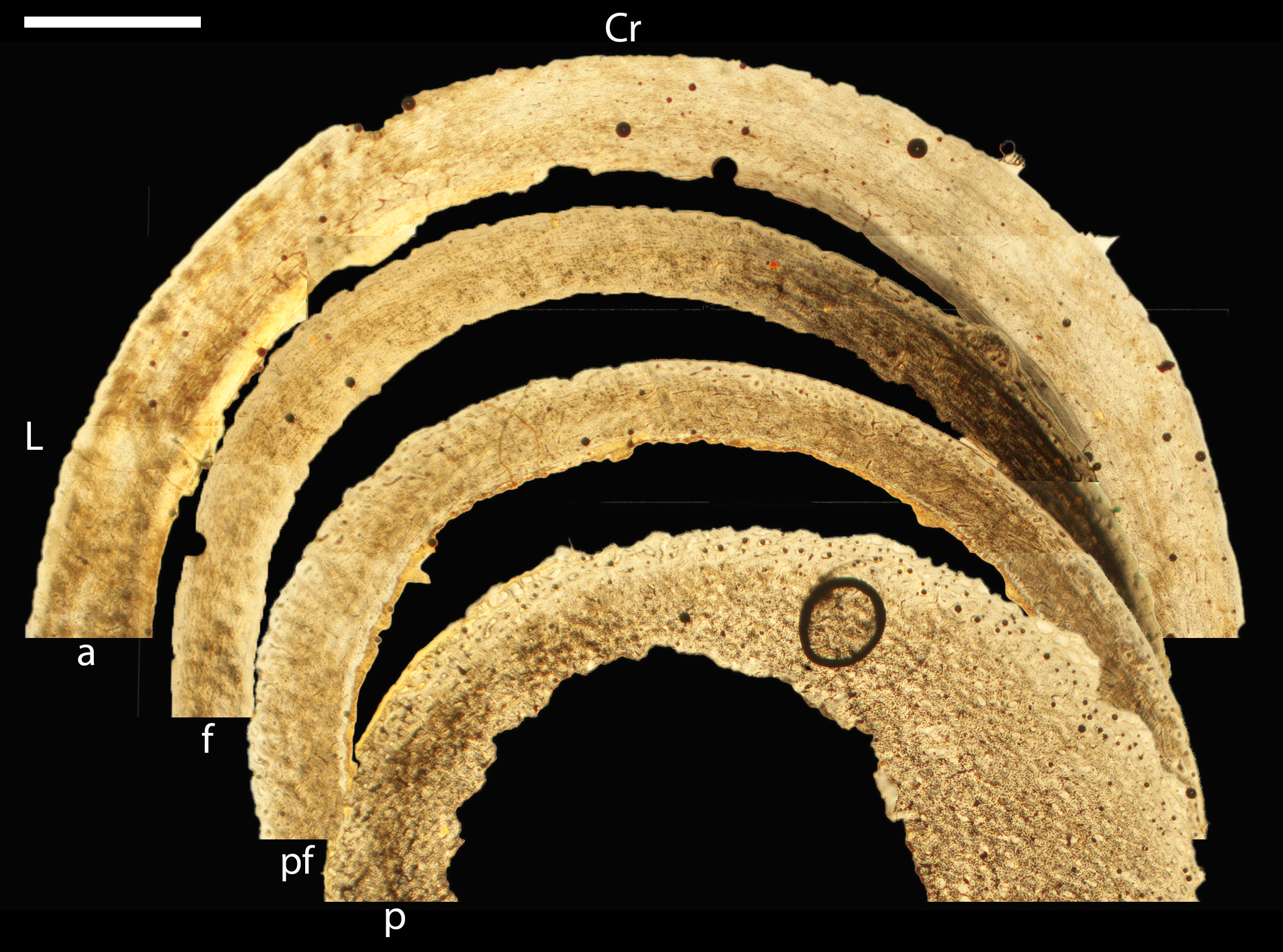

Supplement: Supplemental Information 47 — p = pin-feathered, MVZ190881; pf = pre-fledgling, MVZ190882; f = fledgling, MVZ190879; a = adult, MVZ190883. Cr, cranial; L, Lateral. Scale bar equals 1,000 µm. [file peerj-09-12160-s047.jpg]

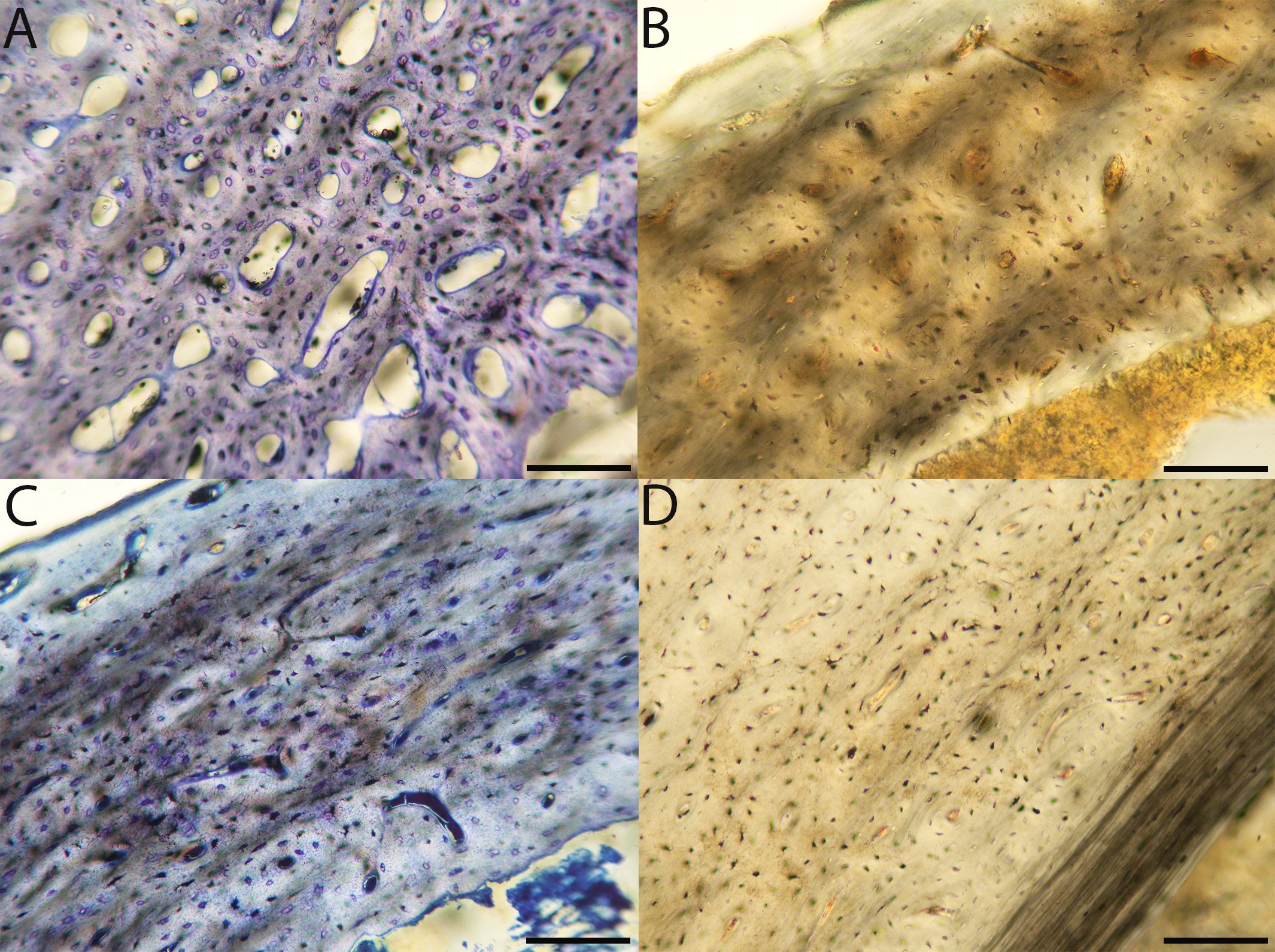

Supplement: Supplemental Information 48 — (A) Femur of pin-feathered chick (MVZ190881), composed of woven bone large vascular channels that are irregular in shape and size. Plane light, toluidine blue, 400×. (B) Pre-fledgling chick (MVZ190882). Plane light, 400×. (C) Fledgling femur (MVZ190879) composed of mature fibrolamellar bone. Plane light, toluidine blue stain, 400×. (D) Adult femur (MVZ190883) showing a mature fibrolamellar complex; a prominent ICL is also visible. Plane light, 400×. In all images the periosteal surface is oriented to top/left and the endosteal to the bottom/right. Scale bare equals 100 µm. [file peerj-09-12160-s048.jpg]

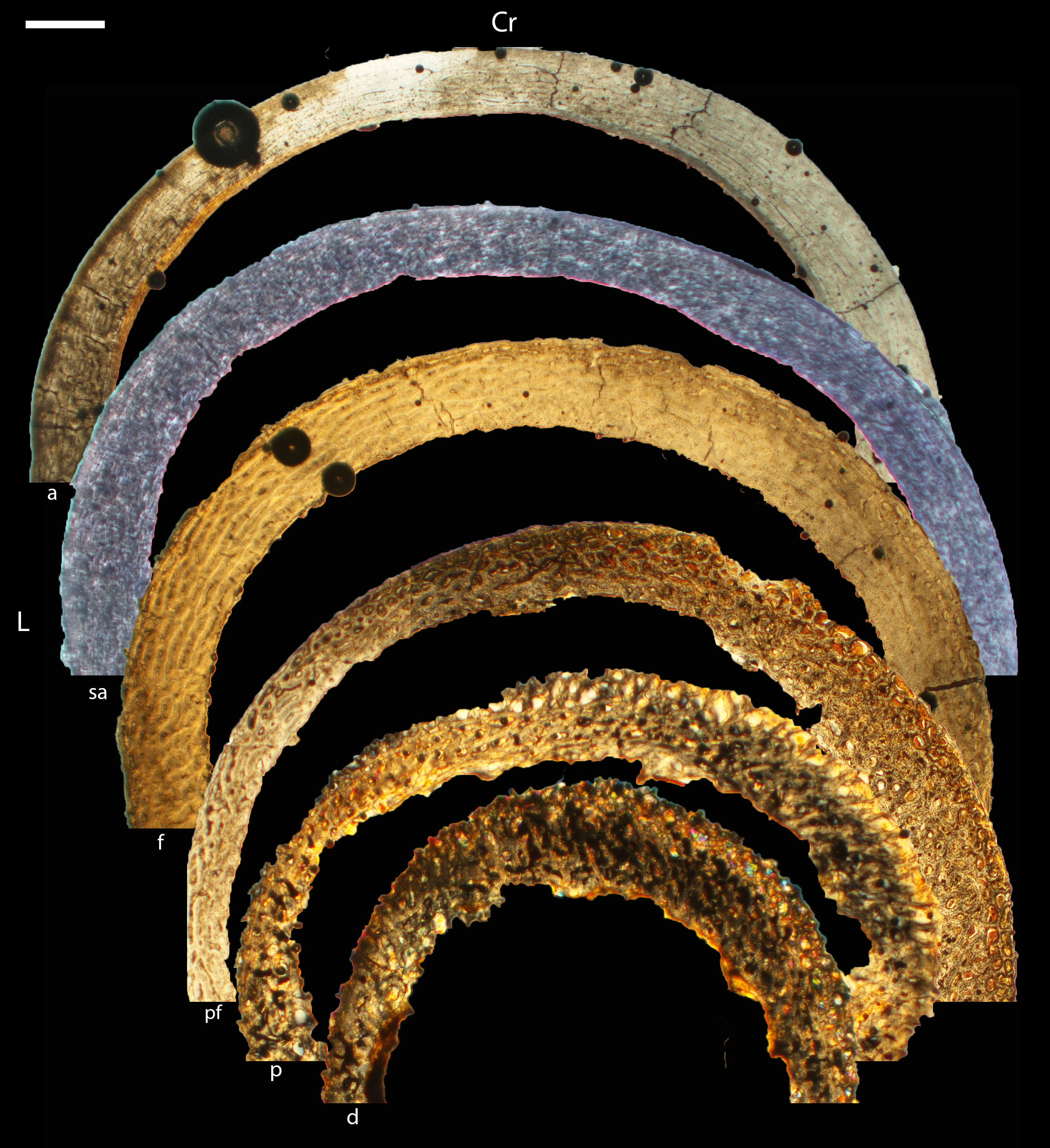

Supplement: Supplemental Information 49 — d = downy, MVZ190875; p = pin-feathered chick, MVZ190868; pf = pre-fledgling, MVZ190866, f = fledgling, MVZ190867; sa = sub-adult, MVZ190869; a = adult, MVZ190872. Cr, cranial; L, Lateral. Scale bar equals 500 µm. [file peerj-09-12160-s049.jpg]

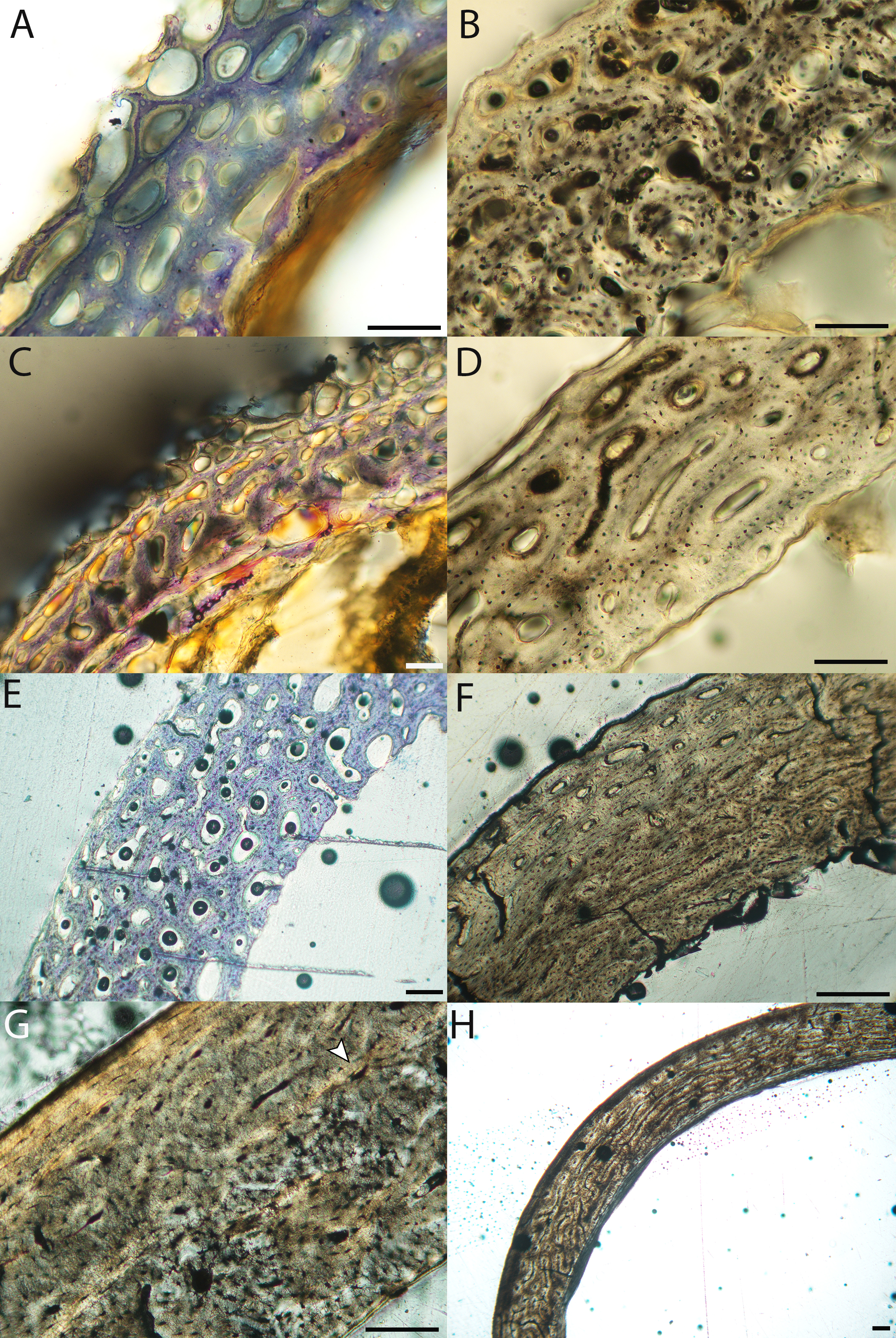

Supplement: Supplemental Information 50 — (A) Humerus of a downy chick (MVZ190875), composed of woven bone with large, irregular vascular canals separated by thick struts of bone. Plane light, toluidine blue stain, 200×. (B) Humerus of a pin-feathered chick (MVZ190876) showing disorganized, porous woven bone similar to the downy chick. Plane light, 200×. (C) Humerus of a pin-feathered chick (MVZ190868) with characteristics similar to B, and also showing a region with smaller vascular spaces in the periosteal region of the cortex combined with larger spaces in the endosteal portion. Plane light, toluidine blue stain, 100×. (D) Humerus of a pre-fledgling (MVZ190866) showing developing fibrolamellar bone; there are many incipient primary osteons in this region. Plane light, 200×. (E) Humerus of a pre-fledgling (MVZ190870) composed of incipient fibrolamellar bone. Plane light, toluidine blue stain, 100×. (F) Humerus of a fledgling (MVZ190867), showing a mature fibrolamellar complex in most of the cortical bone, and developing primary osteons with large vascular spaces in the periosteal region of the cortex. (G) Humerus of a sub-adult (MVZ190869) showing a cortex dominated by mature fibrolamellar bone with very small vascular openings; the thin, incipient OCL is also visible. Arrow indicates a structure resembling a growth line, which separates the more organized and less organized regions of the middle cortical layer. Plane light, 200×. Adult humerus (MVZ190872) composed of fibrolamellar bone with a dominant circumferential/anastomosing vascular pattern, between a thin OCL and ICL of parallel-fibered bone. Plane light, 40×. In all images the periosteal surface is oriented to top/left and the endosteal to the bottom/right. Scale bare equals 100 µm. [file peerj-09-12160-s050.jpg]

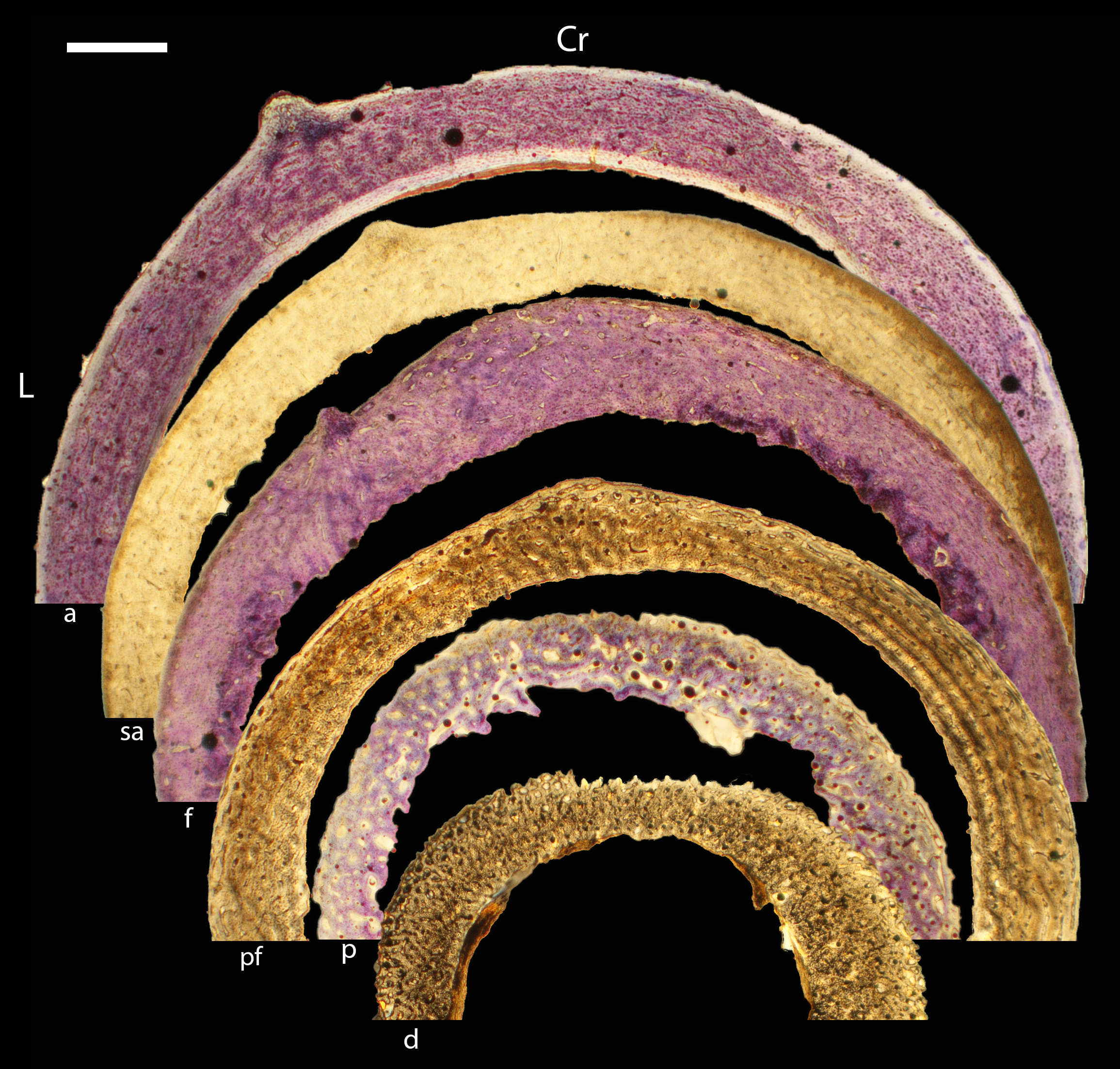

Supplement: Supplemental Information 51 — d = downy, MVZ190875; p = pin-feathered chick, MVZ190868; pf = pre-fledgling, MVZ190870, f = fledgling, MVZ190867; sa = sub-adult, MVZ190869; a = adult, MVZ190877. Cr, cranial; L, Lateral. Scale bar equals 500 µm. [file peerj-09-12160-s051.jpg]

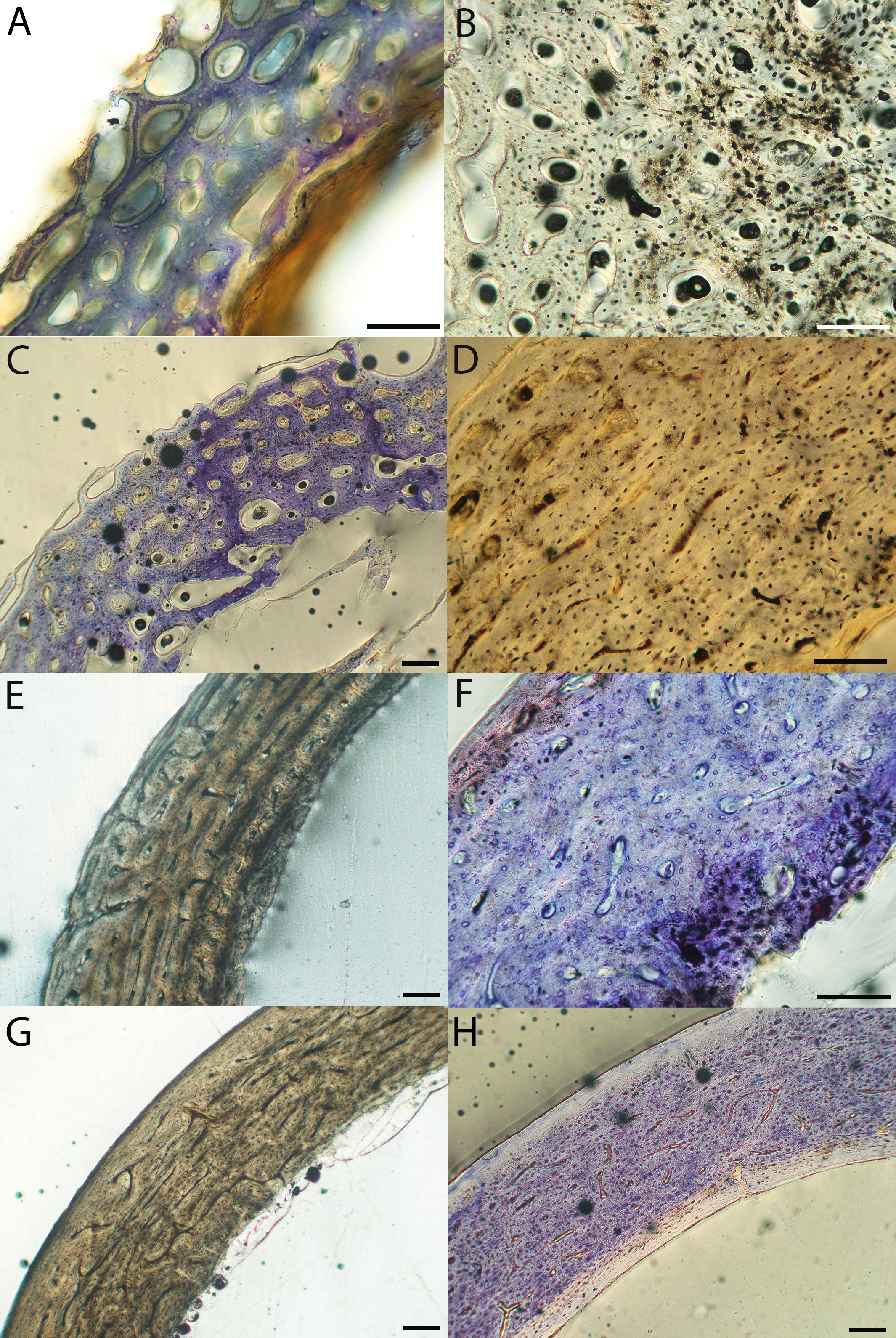

Supplement: Supplemental Information 52 — (A) Femur of a downy chick (MVZ190875) composed of woven bone with large vascular canals; the endosteal two-thirds of the cortex is more mature, with slightly smaller vascular spaces and thicker struts of bone separating them. Plane light, toluidine blue stain 200×. (B) Femur of the pin-feathered chick with a thicker cortex (MVZ190876), showing woven bone a concentration of more mature osteocyte lacunae in the endosteal two-thirds of the cortex. Plane light, 200×. (C) Femur of the pin-feathered chick with the thinner cortex (MVZ190868), composed of woven bone with several incipient primary osteons. Plane light, toluidine blue stain, 100×. (D) Femur of a pre-fledgling (MVZ190866) showing a fibrolamellar complex with larger, simple vascular channels near the periosteal edge. Plane light, 200×. (E) Femur of a pre-fledgling (MVZ190870) showing characteristics similar to D. Plane light, 100×. (F) Femur of a fledgling (MVZ190867) composed of fibrolamellar bone with lower vascular porosity than the pre-fledgling stage, with an incipient OCL of parallel-fibered bone developing along the periosteal margin. Plane light, toluidine blue stain, 200×. (G) Femur of a sub-adult (MVZ190869) showing fibrolamellar bone with many narrow circumferential and anastomosing vascular canals and a distinct OCL. Plane light, 100×. (H) Adult femur (MVZ190877), showing characteristics similar to G but with a mature ICL in addition to an OCL. Plane light, toluidine blue stain, 100×. In all images the periosteal surface is oriented to top/left and the endosteal to the bottom/right. Scale bare equals 100 µm. [file peerj-09-12160-s052.jpg]

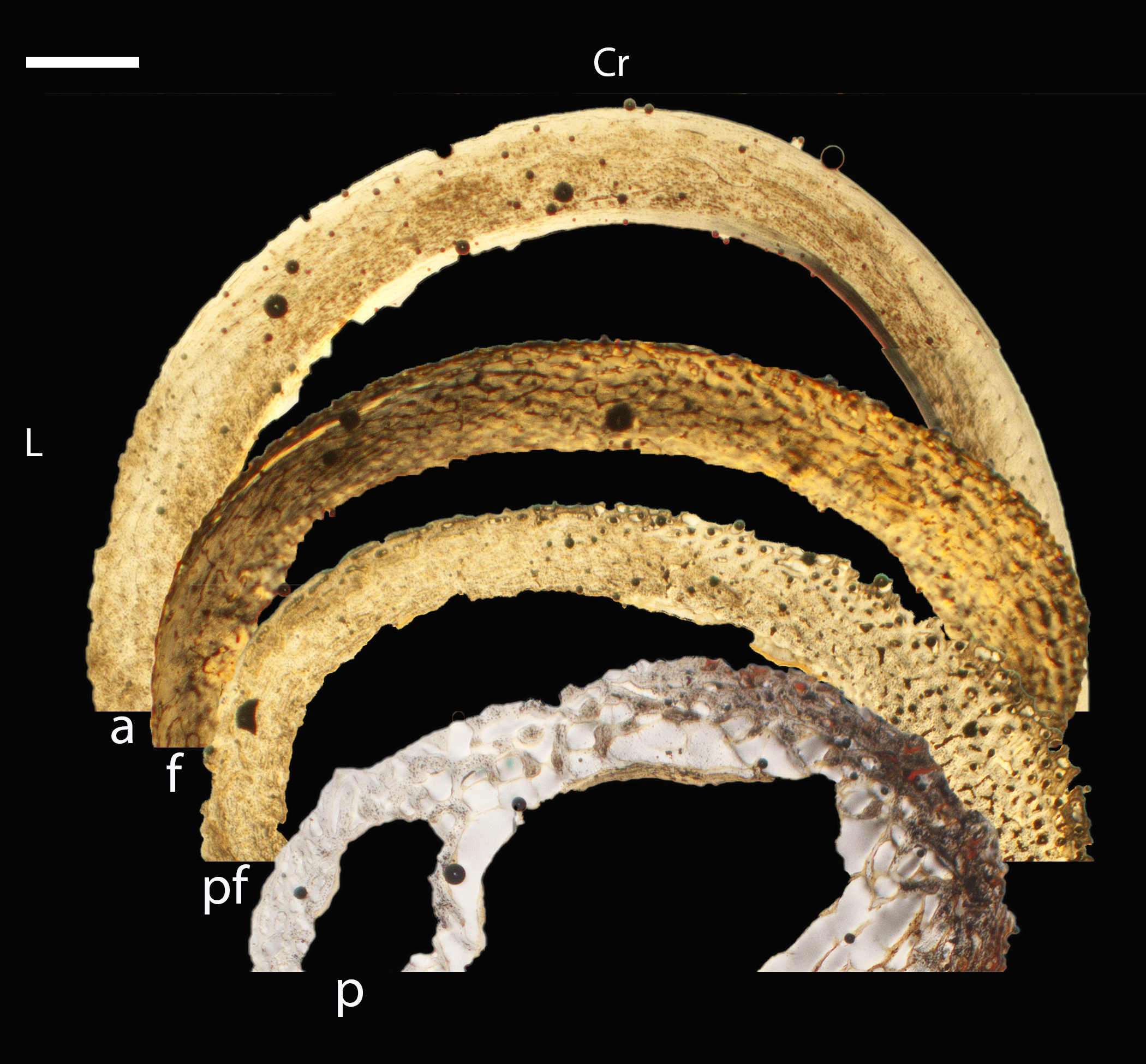

Supplement: Supplemental Information 53 — p = pin-feathered chick, MVZ190862; pf = pre-fledging, MVZ190858; f = fledgling, MVZ190860; a = adult, MVZ190861. Cr, cranial; L, Lateral. Scale bar equals 500 µm. [file peerj-09-12160-s053.jpg]

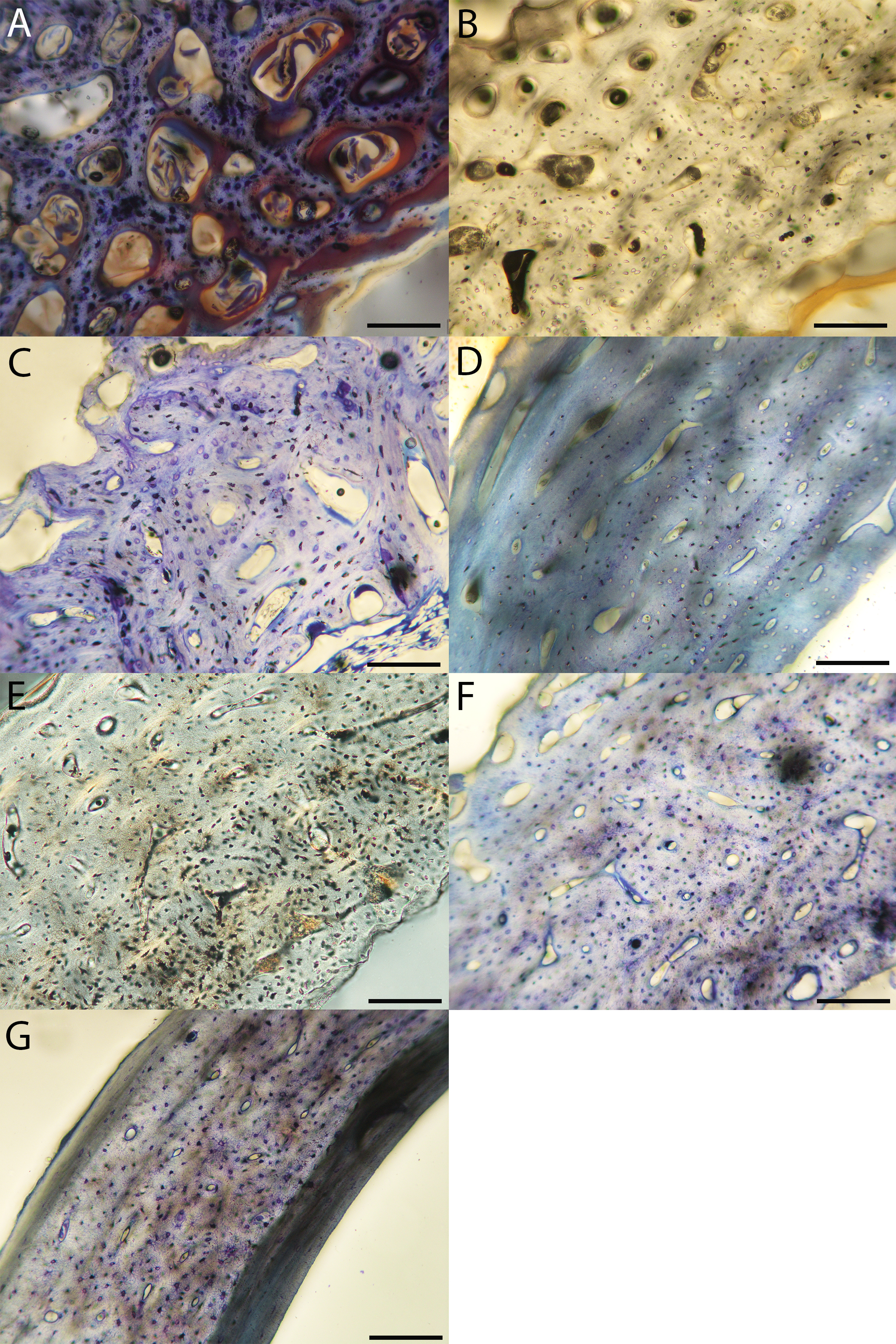

Supplement: Supplemental Information 54 — (A) Humerus of a pin-feathered chick (MVZ190862) showing a porous cortext of woven bone with a high density of osteocyte lacunae. Plane light, toluidine blue stain, 200×. (B) Humerus of a pre-fledgling chick (MVZ190858) showing a thin region of the cortex, with some incipient primary osteons (toward the endosteal surface) and many large, simple vascular canals. Plane light, 200×. (C) Humerus of a pre-fledling chick (MVZ190864) showing a thinner section of the cortex with a higher concentration of incipient primary osteons and lower vascular porosity. Plane light, toluidine blue stain, 200×. (D) Humerus of a fledgling chick (MVZ190860) showing a thicker region of the cortex still with fairly large vascular channel. Plane light, toluidine blue stain, 200×. (E) Humerus of a fledgling chick (MVZ190859) in a thin region of the cortex; the inner two-thirds are mature fibrolamellar bone with low vascular porosity, while the periosteal region is incipient fibrolamelar with larger vascular spaces. Plane light, 200×. (F) Humerus of a fledgling chick (MVZ191134) with characteristics similar to E. Plane light, 200×. (G) Adult humerus (MVZ190861) showing a region composed of fibrolamellar bone between a distinct OCL and ICL; in other parts of the cortex this middle layer consisted of woven bone with simple vascular canals. Plane light, toluidine blue stain, 200×. In all images the periosteal surface is oriented to top/left and the endosteal to the bottom/right. Scale bare equals 100 µm. [file peerj-09-12160-s054.jpg]

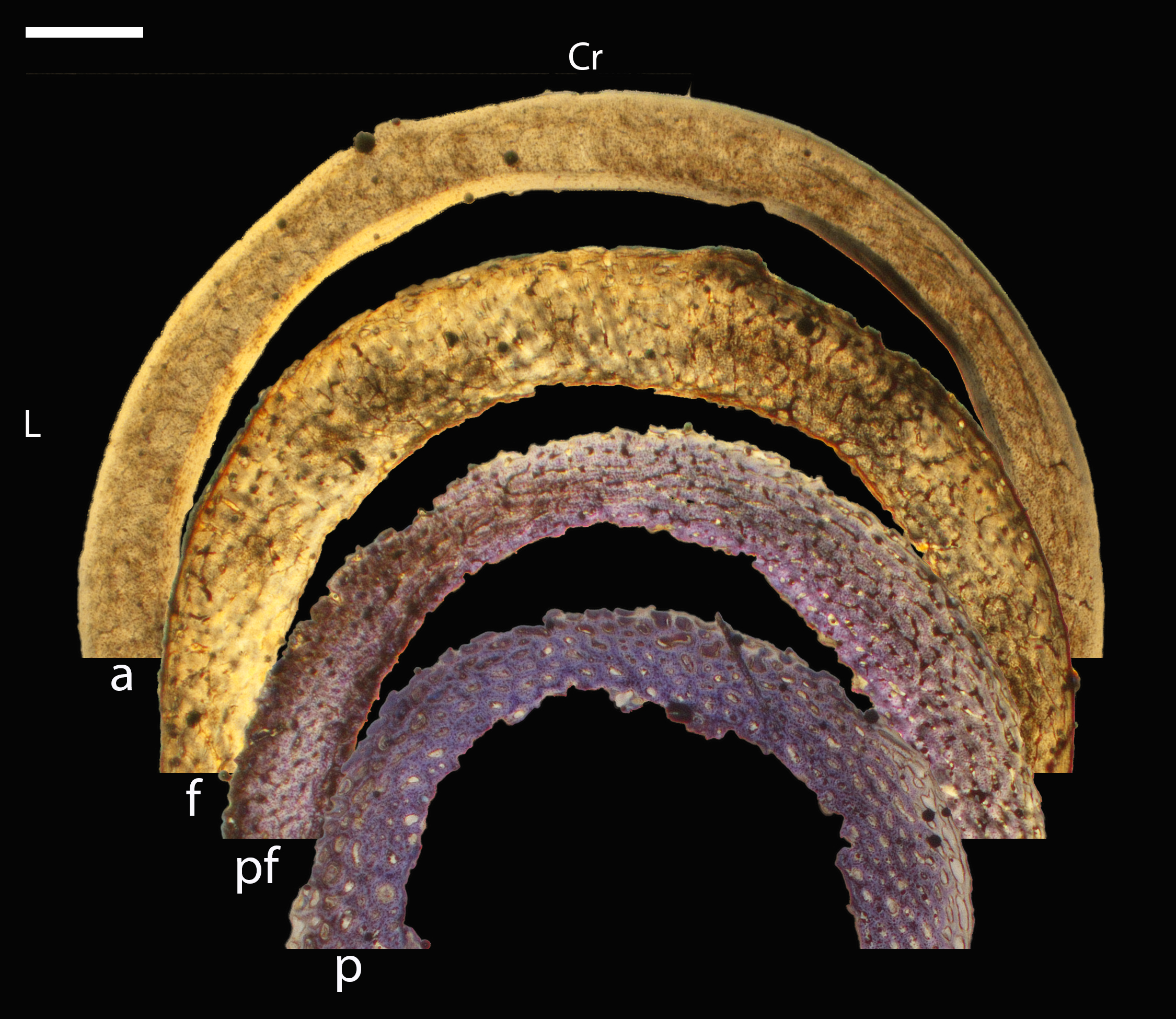

Supplement: Supplemental Information 55 — p = pin-feathered chick, MVZ190862; pf = pre-fledging, MVZ190858; f = fledgling, MVZ190860; a = adult, MVZ190861. Cr, cranial; L, Lateral. Scale bar equals 500 µm. [file peerj-09-12160-s055.jpg]

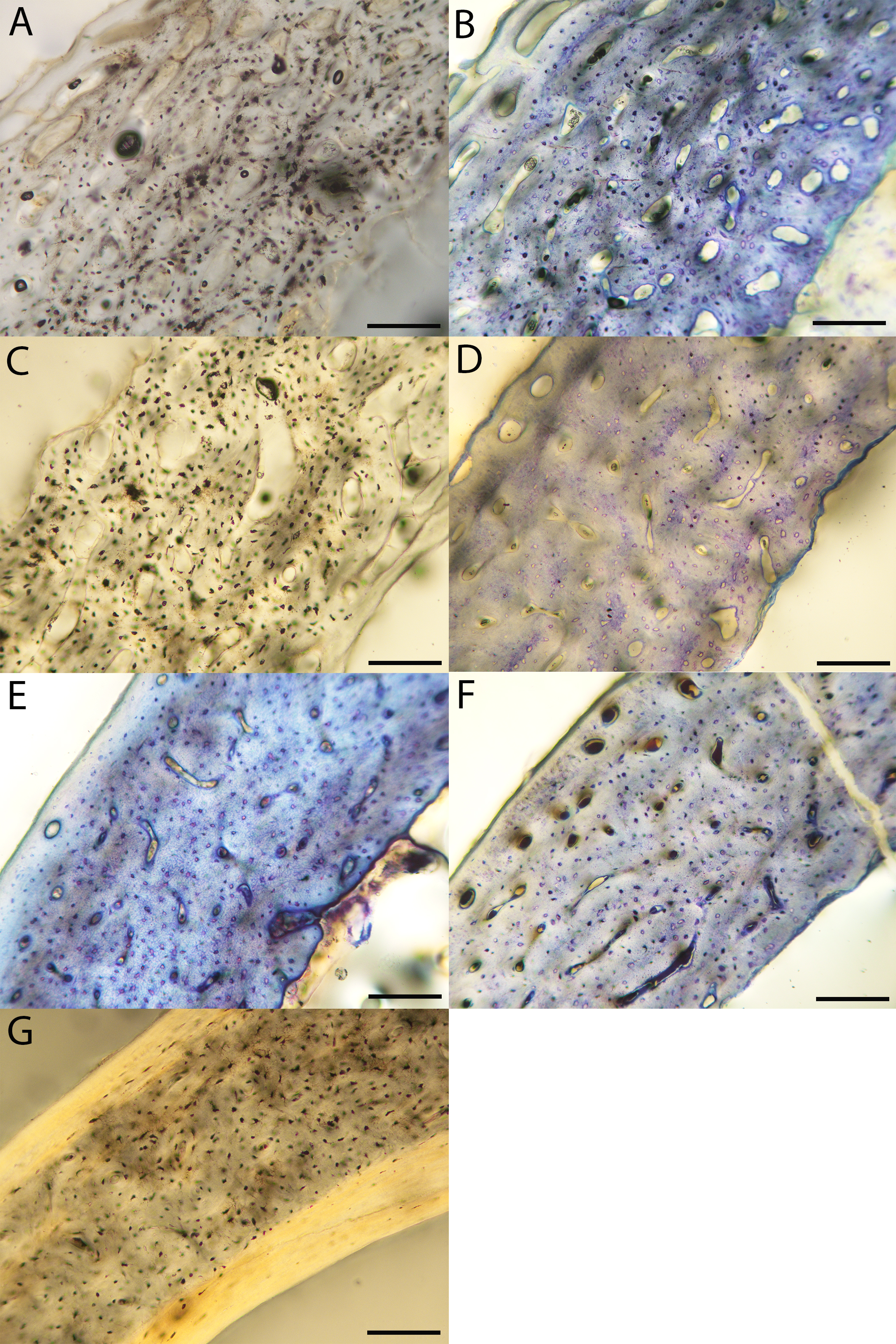

Supplement: Supplemental Information 56 — (A) Femur of a pin-feathered chick (MVZ190862) showing woven bone with high vascular porosity and a high density of osteocyte lacunae. Plane light, 200×. (B) Femur of a pre-fledgling chick (MVZ190858) composed of incipient fibrolamellar bone with smaller vascular openings than the pin-feathered stage. Plane light, toluidine blue stain, 200×. (C) Femur of a pre-fledgling chick (MVZ190864) with characteristics similar to B. Plane light, 200×. (D) Femur of a fledgling chick (MVZ190860) with an irregular endosteal border and thin periosteal layer of woven bone on either side of fibrolamellar bone. Plane light, toluidine blue stain, 200×. (E) Femur of a fledgling chick (MVZ190859) composed predominantly of fibrolamellar bone, with vascular porosity highly reduced relative to the pre-fledgling stage. Plane light, toluidine blue stain, 200×. (F) Femur of a fledgling chick (MVZ191134) showing characteristics similar to D and E. Plane light, toluidine blue stain, 200×. (G) Adult femur (MVZ190861) showing fibrolamellar bone between a prominent OCL and a thick ICL. Plane light, 200×. In all images the periosteal surface is oriented to top/left and the endosteal to the bottom/right. Scale bare equals 100 µm. [file peerj-09-12160-s056.jpg]

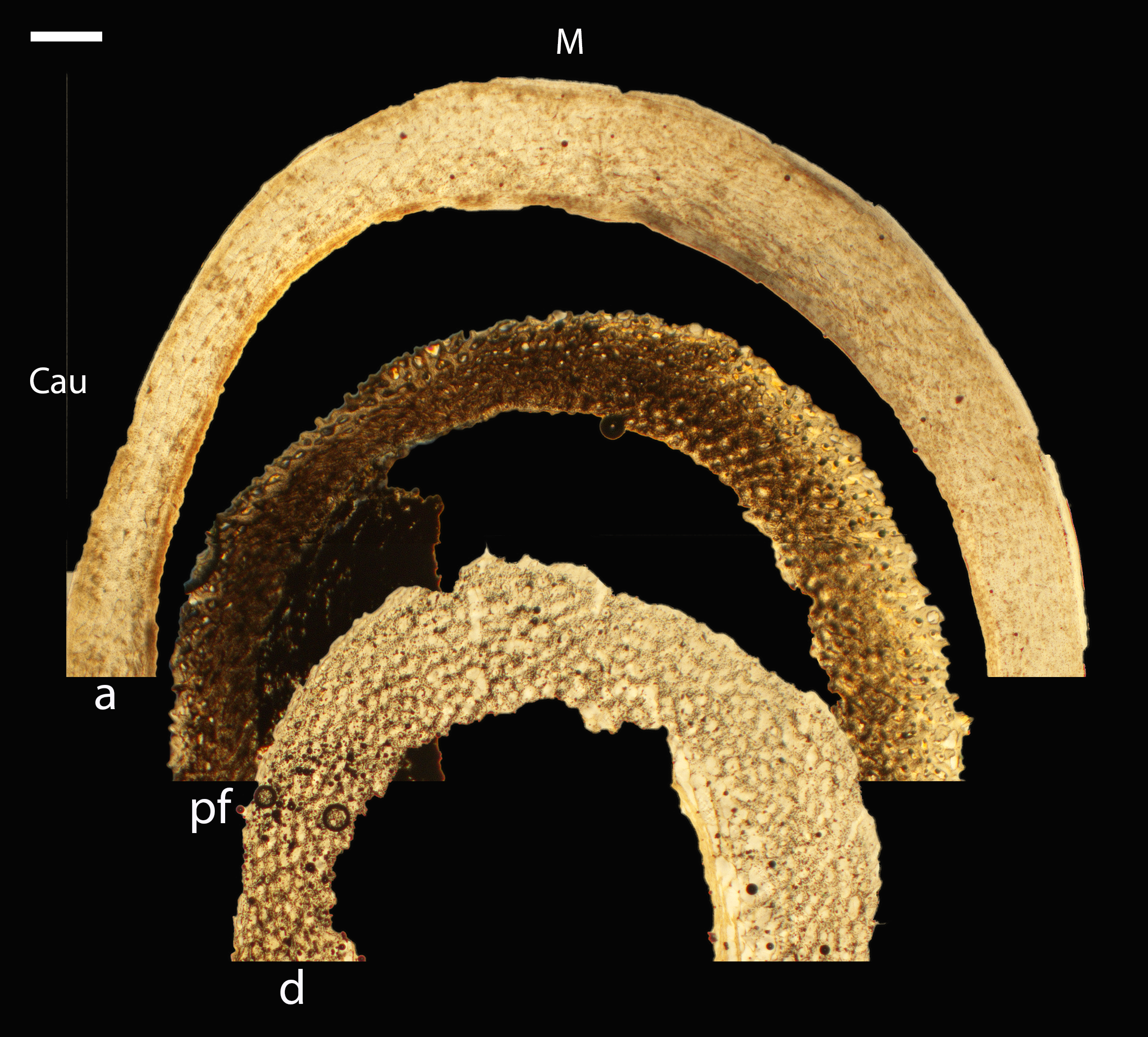

Supplement: Supplemental Information 57 — d = downy, MVZ190852; pf = pre-fledgling, MVZ190853; a = adult, MVZ190855. Cau, caudal; M, medial. Scale bar equals 500 µm. [file peerj-09-12160-s057.jpg]

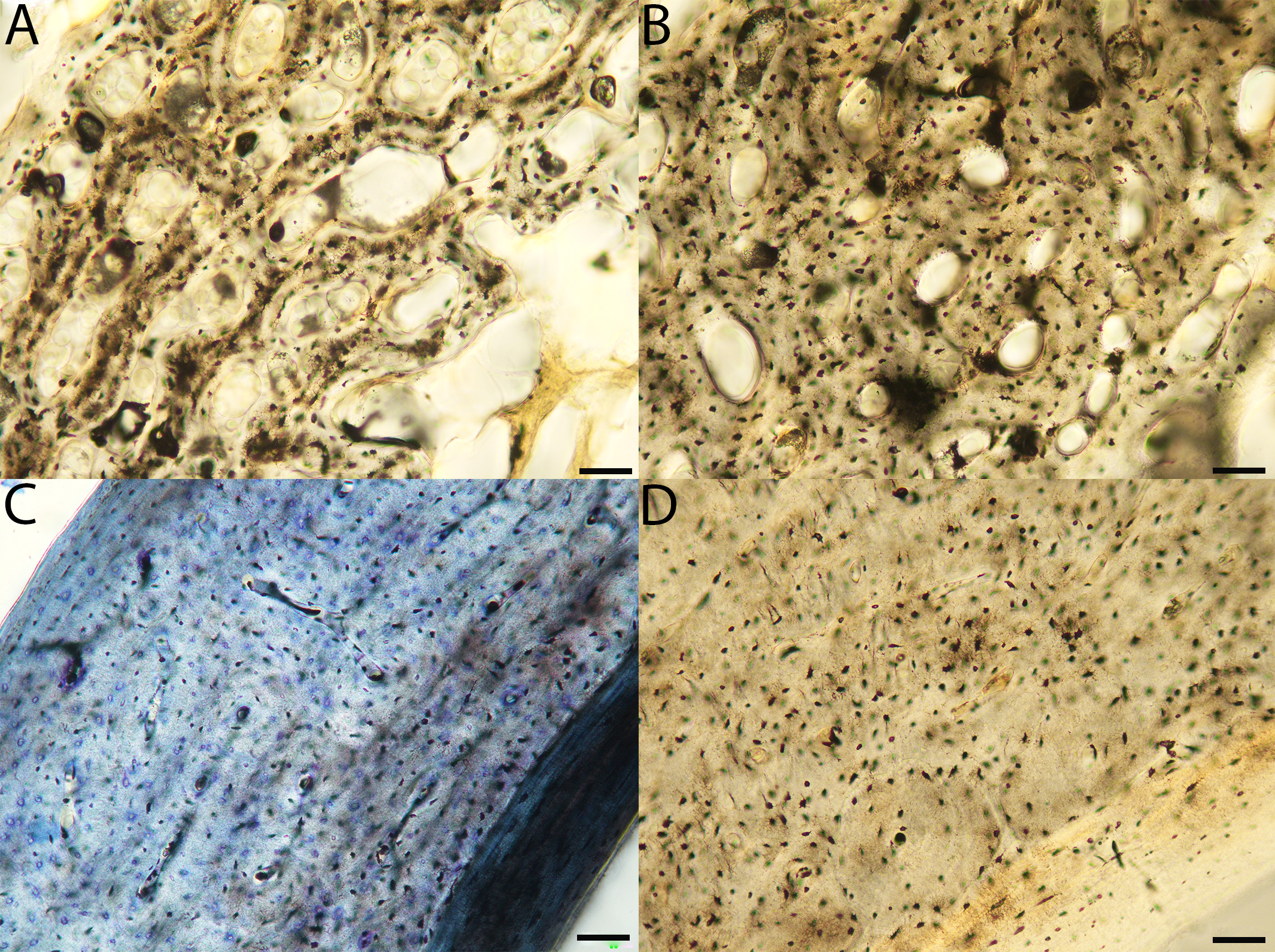

Supplement: Supplemental Information 58 — (A) Humerus of a downy chick (MVZ190852) composed of woven bone with large, irregularly shaped vascular channels. Plane light, 200×. (B) Humerus of a pre-fledgling chick (MVZ190853) with characteristics similar to A, but thicker struts of bone separate vascular channels (which, as a consequence, are smaller in comparison). Plane light, 200×. (C) Adult humerus (MVZ190855) showing the thick middle layer of fibrolamellar bone between and OCL and ICL that characterizes the adult red-tailed hawk. Plane light, toluidine blue stain, 200×. (D) Adult humerus (MVZ190854) showing a close-up on a region of fibrolamellar bone with secondary osteons. Plane light, 200×. In all images the periosteal surface is oriented to top/left and the endosteal to the bottom/right. Scale bare equals 50 µm. [file peerj-09-12160-s058.jpg]

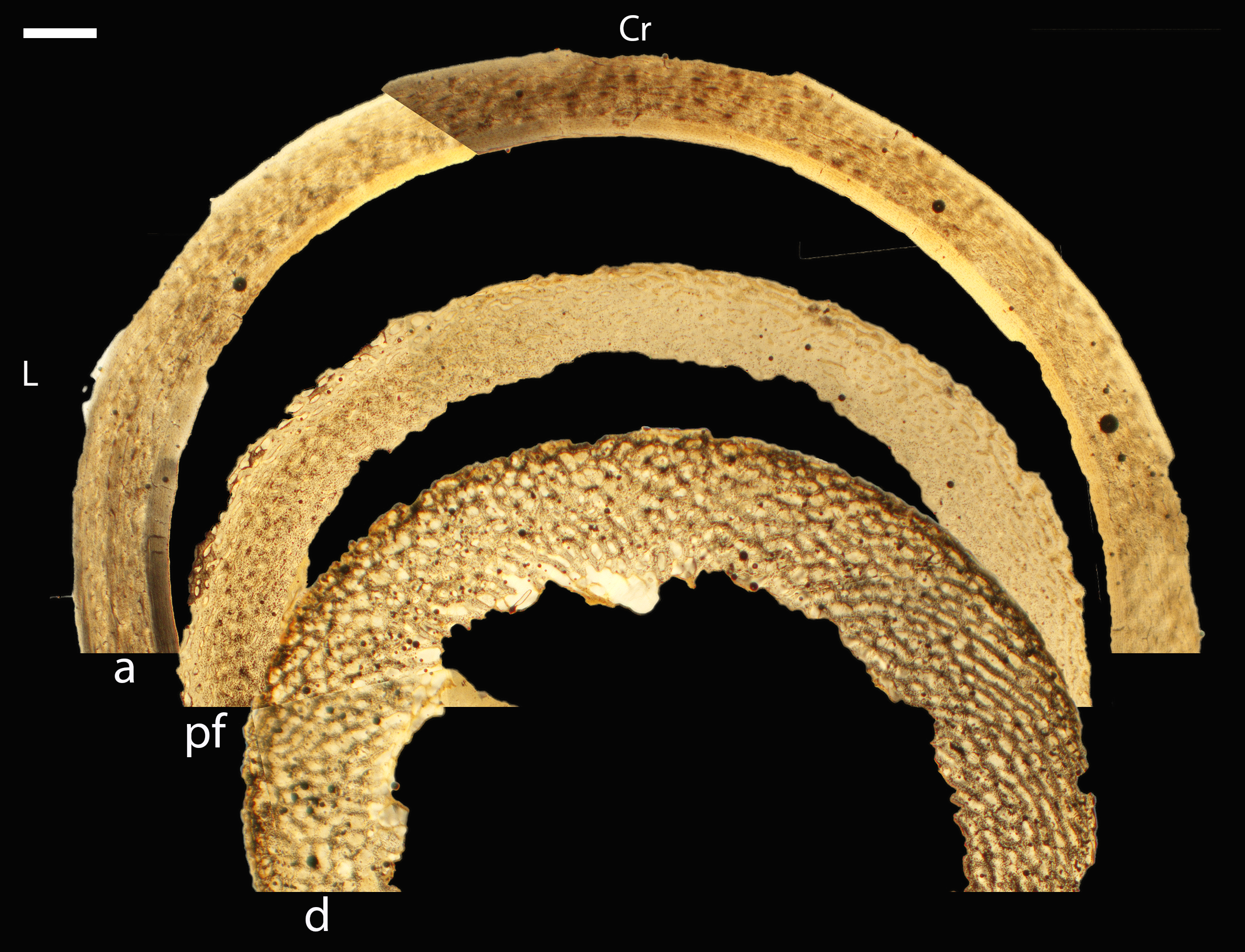

Supplement: Supplemental Information 59 — d = downy, MVZ190852; pf = pre-fledgling, MVZ190853; a = adult, MVZ190854. Scale bar equals 500 µm. [file peerj-09-12160-s059.jpg]

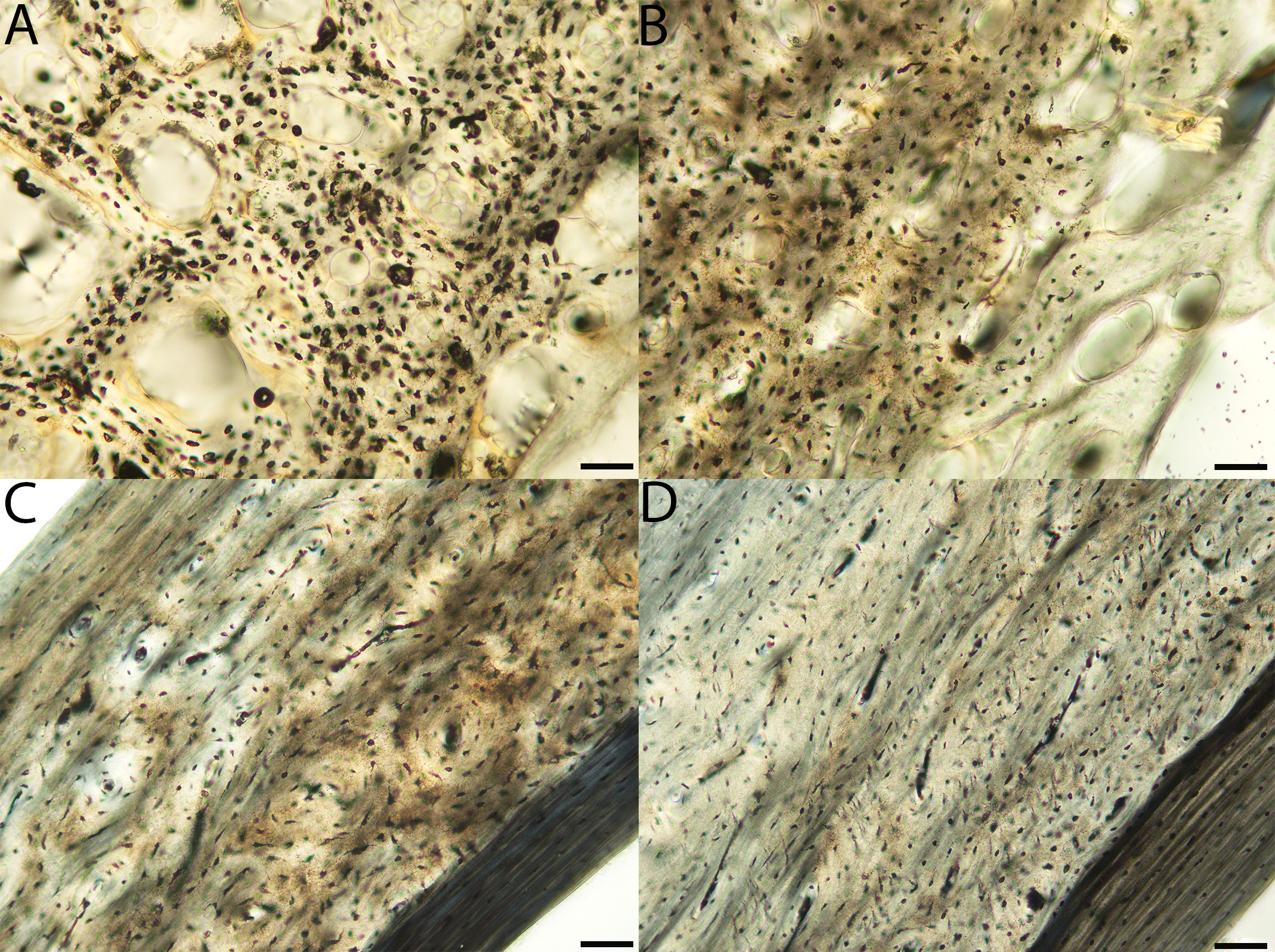

Supplement: Supplemental Information 60 — (A) Femur of a downy chick (MVZ190852) showing woven bone with large vascular channels and a high density of osteocyte lacunae. Plane light, 200×. (B) Femur of a pre-fledgling chick (MVZ190853) with a cortex of woven bone in which vascular canals are smaller than the downy stage; here they are seen oriented in many directions. Plane light, 200×. (C) Adult femur (MVZ190855) showing fibrolamellar bone between a thin OCL and a thicker, more fully developed ICL. Plane light, 200×. (D) Adult femur (MVZ190854) showing characteristics similar to C. Plane light, 200×. In all images the periosteal surface is oriented to top/left and the endosteal to the bottom/right. Scale bare equals 50 µm. [file peerj-09-12160-s060.jpg]
